# Structure-Based Phylogenetic Analysis of the Lipocalin Superfamily

**DOI:** 10.1371/journal.pone.0135507

**Published:** 2015-08-11

**Authors:** Balasubramanian Lakshmi, Madhulika Mishra, Narayanaswamy Srinivasan, Govindaraju Archunan

**Affiliations:** 1 Department of Animal Science, Bharathidasan University, Tiruchirappalli, 620024, India; 2 Molecular Biophysics Unit, Indian Institute of Science, Bangalore, 560012, India; 3 Department of Biochemistry, Indian Institute of Science, Bangalore, 560012, India; University of Rome Tor Vergata, ITALY

## Abstract

Lipocalins constitute a superfamily of extracellular proteins that are found in all three kingdoms of life. Although very divergent in their sequences and functions, they show remarkable similarity in 3-D structures. Lipocalins bind and transport small hydrophobic molecules. Earlier sequence-based phylogenetic studies of lipocalins highlighted that they have a long evolutionary history. However the molecular and structural basis of their functional diversity is not completely understood. The main objective of the present study is to understand functional diversity of the lipocalins using a structure-based phylogenetic approach. The present study with 39 protein domains from the lipocalin superfamily suggests that the clusters of lipocalins obtained by structure-based phylogeny correspond well with the functional diversity. The detailed analysis on each of the clusters and sub-clusters reveals that the 39 lipocalin domains cluster based on their mode of ligand binding though the clustering was performed on the basis of gross domain structure. The outliers in the phylogenetic tree are often from single member families. Also structure-based phylogenetic approach has provided pointers to assign putative function for the domains of unknown function in lipocalin family. The approach employed in the present study can be used in the future for the functional identification of new lipocalin proteins and may be extended to other protein families where members show poor sequence similarity but high structural similarity.

## Introduction

**Fig 1:**
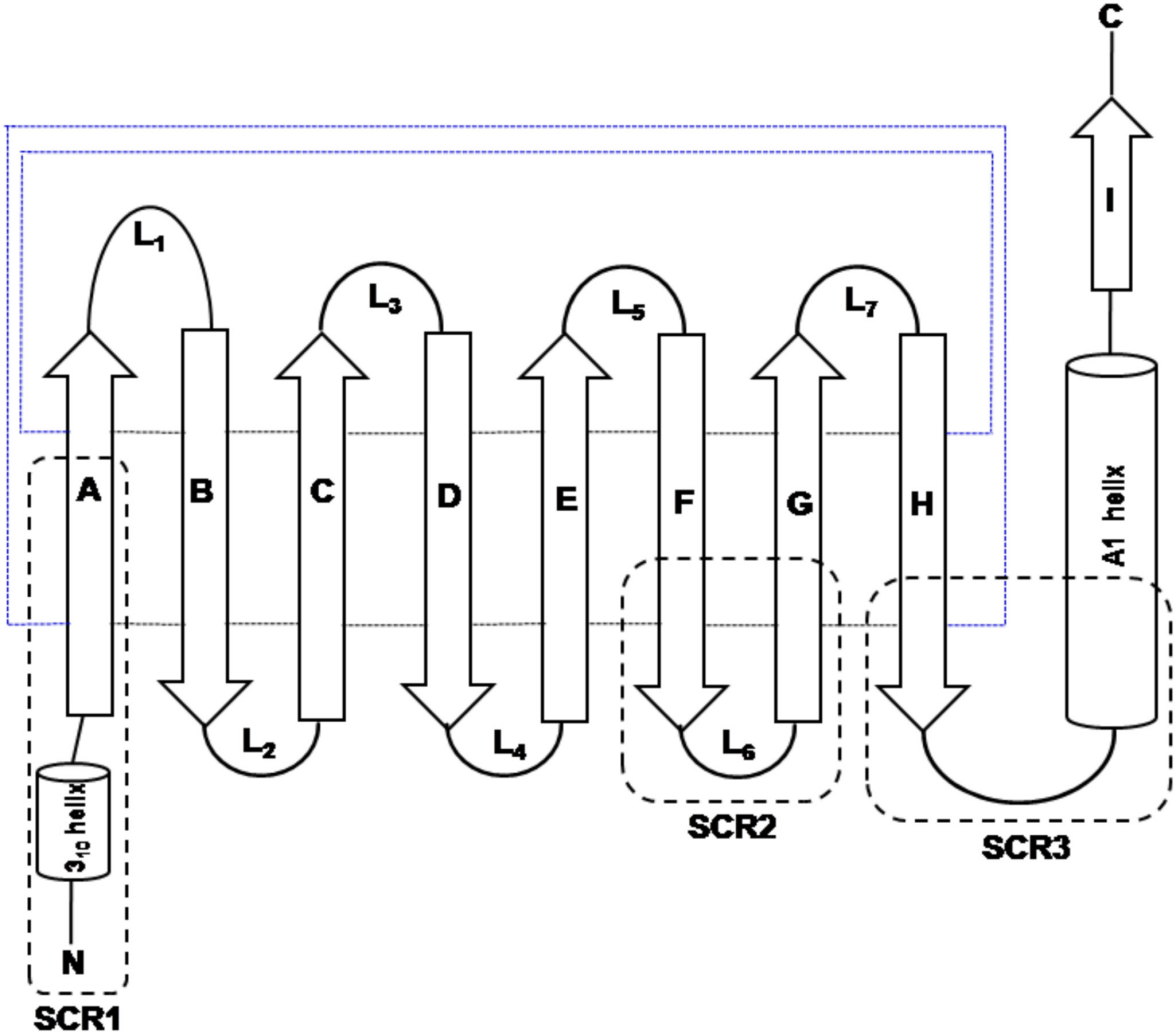
Topology diagram of the lipocalin fold. The strands [A-I] of the β-sheet and the helices are represented as arrows and cylinders respectively. The loops connecting the strands are marked as L1-L7. The hydrogen bond connections between the strands are shown in small dotted lines. The three structurally conserved regions (SCR) are marked as SCR1, SCR2 and SCR3 respectively. Blue lines indicates that β-strands A and H are in-register and thus forming a β-barrel structure. Black lines indicates that adjacently shown strands are in-register.

The lipocalin family of proteins arose early in the tree of life as suggested from the presence of extant proteins in prokaryotes and eukaryotes. Lipocalins constitute a heterogeneous group of secreted proteins that bind a wide variety of small hydrophobic ligands with high affinity [1]. Lipocalins vary in their lengths from 160 to 180 amino acid residues which correspond to a molecular mass of roughly 20 kDa [2, 3]. It is a well established fact that the lipocalins are quite diverse at the level of amino acid sequences with pair-wise sequence identity between many homologues below 30%. Lipocalins have three short conserved motifs that are characteristic features of the lipocalin protein family [4, 5]. However, it is interesting that the family has a well conserved overall three-dimensional structure. The topology of the lipocalin fold is shown in Fig 1. Lipocalin structures are made of a well conserved eight-stranded anti-parallel β-barrel (marked as A to H in Fig 1),a 3_10_ helix and an α-helix [1, 6]. The eight β-strands (A-H) closes back on itself to form continuous network of hydrogen bonds. The three short conserved regions (SCR) in lipocalins are also shown in Fig 1 and these regions often serve as diagnostic features of lipocalins. All the loops are β-hairpins except loop L_1_ which is larger and acts as a lid of the barrel in most of the lipocalins. The barrel is open at one end and encompasses the well buried ligand binding pocket in the other end of the structure [6, 7]. Lipocalin protein family is characterized by their functional diversity in terms of their molecular recognition properties that include the ability to bind various hydrophobic small molecules, cell surface receptors and other macromolecular complexes [1, 3, 6]. In the recent years, there is a significant increase in the number of known members in this family and a corresponding increase in its functional repertoire and molecular recognition properties. Therefore, it is important to understand the evolutionary history of the lipocalins.

There have been a number of studies in the past exploring various evolutionary aspects of lipocalins especially using sequence-based approaches [8–13]. These evolutionary studies suggested that the tree of lipocalins is rooted at the bacterial lipocalin genes and are probably evolved from a common ancestor and not by horizontal gene transfer [14]. Based on the earlier studies it is learnt that evolutionarily recent lipocalins are characterized by high evolutionary divergence (less than 30% sequence identity), optimization of flexibility of lipocalins and efficiency of ligand binding [13]. Though there is abundant information available on the functional and biochemical aspects of lipocalins, only few comprehensive phylogenetic studies have been reported for these proteins. However the results obtained from the previous studies mentioned above are of low confidence, as the phylogenetic trees were generated solely on the basis of sequences which are highly diverged. But in recent times, in addition to the increase in the knowledge of functional and biochemical aspects of lipocalins, extensive three dimensional structural information has also become available.

It is well known that the residues crucial for the structure and function of proteins are conserved better than other residues during the course of evolution. Traditionally, sequence-based phylogenetic approaches are used to study the evolutionary history of related proteins. However3D-structuresareknown to be conserved better than the amino acid sequences during the course of evolution. Earlier studies have shown that 3Dstructure-based phylogenetic studies help to understand the evolutionary relationship better as compared to the sequence-based phylogenetic trees [15, 16], particularly, when the sequence identity is low (less than about 30%) among the homologues. In the current work we have used a 3D structure based phylogenetic approach to understand the basis of functional diversity of lipocalins.

## Materials and Methods

### Dataset

**Fig 2:**
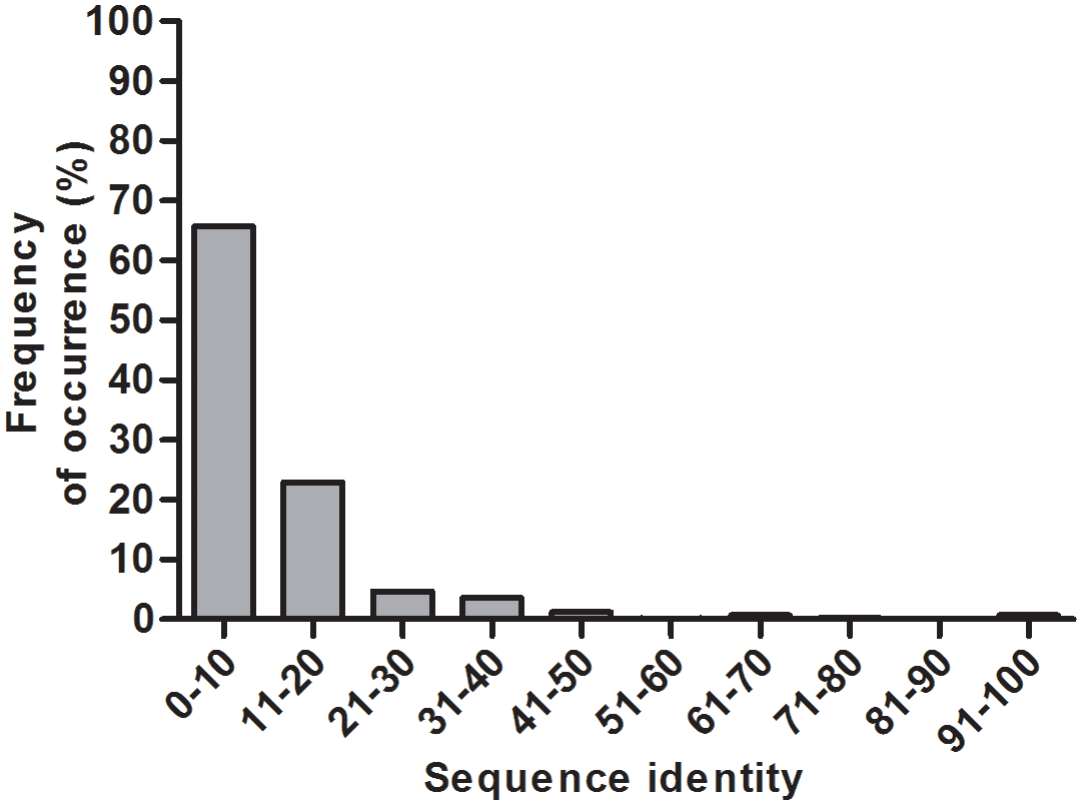
Distribution of sequence identity for 39 lipocalin domains.

According to the Structural Classification of Proteins (SCOP) [17] database lipocalin structures correspond to "lipocalin fold" which is categorized under all β-class of proteins. Lipocalin fold in SCOP (version 1.75)contains only one superfamily referred to as "Lipocalins" comprising of 9 families. In the lipocalin superfamily, crystal structures of 378 lipocalin domains comprising of ligand bound and ligand-free forms are available. Generally lipocalins have the tendency to bind to different types of ligands. Further ligand binding leads to structural changes. But in the current study our interest is to analyze the genuine structural characteristics without the influence of any external factors. So, for the current analysis 39 out of 378 protein domains available in the ligand-free forms have been considered. These 39 structures span 9 protein domain families in the lipocalin superfamily. S1 Table lists these 39 domains with their SCOP codes, organism names and SCOP family names. Pair-wise structural comparison was performed for all possible pairs from the dataset of 39 lipocalin domains resulting in 741 structural comparisons. Structural comparisons have been carried out using DaliLite pairwise alignment tool [18]. The structural alignments corresponding to these 741 pairs have been provided in S1 Text. The sequence identities corresponding to these alignments have been calculated and the frequencies of occurrence of pairs in various sequence identity ranges are shown in Fig 2. Over 80% of lipocalin domain pairs in the current dataset correspond to the sequence identity range of 1–20%. Therefore, sequence-based phylogeny studies may be less effective than the 3-D structure-based evolutionary analysis owing to low sequence similarity among the homologues.

### Phylogenetic tree and structural divergence measure

From the pairwise structural alignments obtained using DaliLite, a structural dissimilarity matrix was computed in order to generate phylogenetic tree. A measure referred to as structural dissimilarity metric (SDM) has been calculated for every pair using the formula [19] given below:

SDM= −100*log(w1*PFTE+w2*SRMS)

Where,

w1=(1−PFTE + 1−SRMS)/2 and w2 =(PFTE+SRMS)/2

PFTE= Numberoftopologicallyequivalentresidues/lengthofthesmallerprotein

SRMS= 1−(RMSD/3.0)

RMSD is the root mean square deviation of topologically equivalent C^α^ atoms in Å. Topological equivalence of C^α^ atoms is defined by ≤3Å cut-off distance between Cα atoms from the two structures after optimal superposition. The SDM values give the measure of the structural dissimilarity for a given pair of structures. Using the above mentioned formula, SDM values for all the 741 domain pairs were calculated. With these values 39 x 39 structure dissimilarity matrix has been generated. This matrix has been used to build the structure-based phylogenetic tree using Kitsch, a distance based algorithm from PHYLIP (version 3.573c) suite of programs [20]. The Kitsch program from the PHYLIP suite uses a Kitsch-Margoliash and Least Squares method with the evolutionary clock. Also, for the 741 pairs, distance matrices were generated on the basis of the structure-based sequence alignments [21]. Also for the 39 domains the traditional sequence-based phylogenetic tree has been obtained using clustalW2 (version 2.1) by considering the Neighbour-joining tree method [21–23]. The sequence alignments obtained using clustalW2 have been provided in S2 Text.

## Results and Discussion

### Phylogenetic analysis

It is known that lipocalin proteins originated in the early history of life. Earlier phylogenetic analysis on this versatile family has suggested that structural characteristics of lipocalins change during the course of evolution [13]. The various clades observed in earlier analysis generally correspond to the overall function of various lipocalins in terms of general nature of binding ligands. Previous analysis of phylogeny of lipocalins used only the sequences in clustering lipocalins. In sequence-based studies of lipocalins, with low sequence similarity, it has been difficult to pin point the functional properties of lipocalin in terms of ligand binding and receptor binding. The possible reasons could be their high sequence divergence and availability of a small number of 3-D structures. With the increased number of both the 3-D structures and the functional information of lipocalins, it is now possible to progress in our understanding of structure-function relationships of lipocalin protein family. In the previous studies from this laboratory the structure-based phylogenetic analysis has been established as an effective method to understand the structure-function relationships of homologues with low sequence identity among them [15]. Given the low-sequence similarity among lipocalins of known 3-D structure, in the current work, structure-based phylogenetic approach has been used to understand the functional diversity of lipocalins. This analysis has been carried out using 39 protein domains of known 3D structure which belong to 9 different protein families of the lipocalin superfamily.

**Fig 3:**
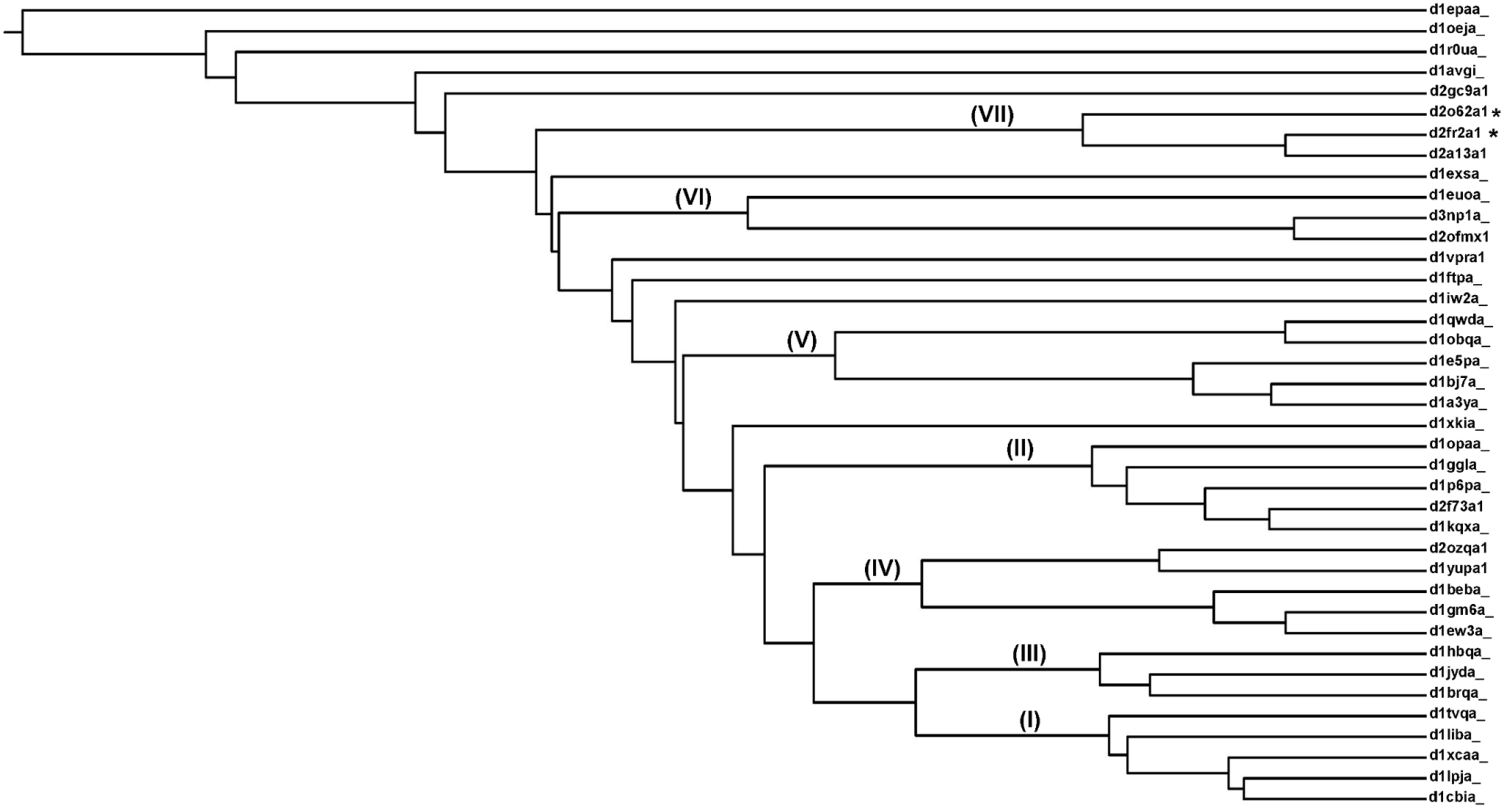
Structure-based phylogenetic tree generated for 39 lipocalin domains. Tree is generated based on the three dimensional structures of lipocalin domains. The clusters observed in the tree are numbered in Roman numerals. The DUFs are represented by asterisks.

Using pair-wise structural comparison for 39 lipocalin structures in the ligand-unbound form, a matrix of structural dissimilarity metric (SDM) (see Materials and Methods section) has been generated. The structure-based phylogenetic tree generated using such a SDM matrix is shown in Fig 3. Various clusters in the dendrogram are represented by Roman numerals (Fig 3) and the members of each of these cluster correspond to the same family. But it is interesting to note that members of a single family group into diverse clusters. The general structural and functional characteristics of each of these clusters are listed in Table 1. Detailed analysis of the 3-D structures suggest that various clusters within the individual families have distinct structural features in their ligand association such as movement of helices and diversity in the sizes of molecules binding at the topologically equivalent binding sites. All the single member families have "clustered" independently and are not part of any multi-lipocalin cluster. In addition two domains for which the function is not known (domain of unknown function (DUF) members) have been observed to cluster along with members of the known function. The detailed analysis of these clusters is provided in the later sections.

**Table 1:** List of clusters obtained based on structure based phylogeny for 9 lipocalin families.

S. No	Clusters	Number of members in each analysis	SCOP Family name	Structural and functional characteristics
1	Cluster I	5	Fatty acid binding protein like	Ligand binding is controlled by opening and closing of α-helix; Same ligand interacting residues; Water mediated ligand interaction
2	Cluster II	5	Fatty acid binding protein like	Ligand binds directly in the binding pocket; Same ligand interacting residue; No water mediated ligand interaction
3	Cluster III	3	Retinol binding protein like	Retinol transport; involves in macromolecular complexation
4	Cluster IV	5	Retinol binding protein like	Involved in dimerisation; ligand binds only by hydrophobic interaction; Binds to small ligand molecules
5	Cluster V	5	Retinol binding protein like	No dimerisation; ligand interaction is by hydrogen bond and hydrophobic interaction; Binds to larger ligand molecule; Glycosylation sites
6	Cluster VI	3	Retinol binding protein like	Transports NO
7	Cluster VII	3	Rv2717c -like	Transports heme; Putative heme binding region. The two DUFsoccur in this cluster.
			All 1756-like	
8	Single member	1	Thromin inhibitor	Lipocalin-like exosite-binding inhibitor
	Single member	1	Hypothetical protein YodA	NIL
	Single member	1	Hypothetical protein YwiB	NIL
	Single member	1	Phenolic acid decarboxylase (PAD)	Decarboxylation of p-coumaric acid that helps in regualtion of cell growth
	Single member	1	Dinoflagellate luciferase repeat	Biolluminescent dinoflagellate

**Fig 4:**
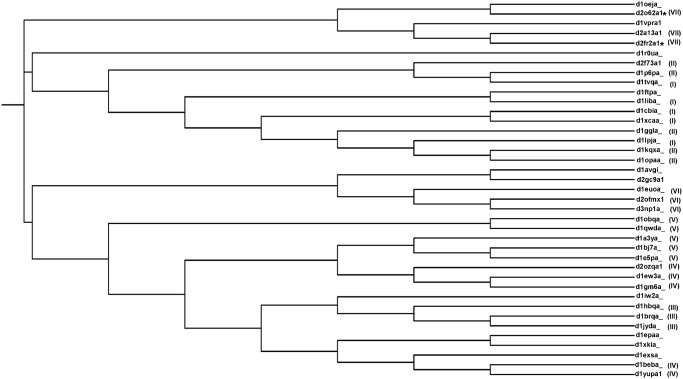
Sequence-based phylogenetic tree generated for 39 lipocalin domains. Tree generated based on the amino acid sequences of lipocalin domains. For each of the members the cluster numbering based on the structure-based tree is represented in brackets. The DUFs are represented by asterisks.

A traditional sequence-based phylogenetic tree has been generated for the 39 domains and is shown in Fig 4. The cluster numbering from the structure-based tree is mentioned for each of the members in sequence-based tree in brackets. Similar to structure-based tree, in sequence-based tree too lipocalin members tend to cluster based on their family. In both sequence and structure-based trees, the two major families Retinol binding protein-like and Fatty acid binding protein-like form many sub-clusters among their family members. But interestingly the clusters are generally different in the two trees. For example, a clear cluster is observed in the structure-based tree especially in the case of domains of unknown function, whereas in sequence-based tree it occurs in two different clusters.

### Structure-function relationship in lipocalin superfamily

In the structure-based phylogenetic tree many clusters are observed in each family (Fig 3). There are four and two clusters found for the major retinol binding protein-like (RBP-like) and fatty acid binding protein-like (FABP-like) families respectively and these are discussed in the subsequent sections. The overall functions of these clusters are listed in Table 1.

### Fatty acid binding protein-like

**Fig 5:**
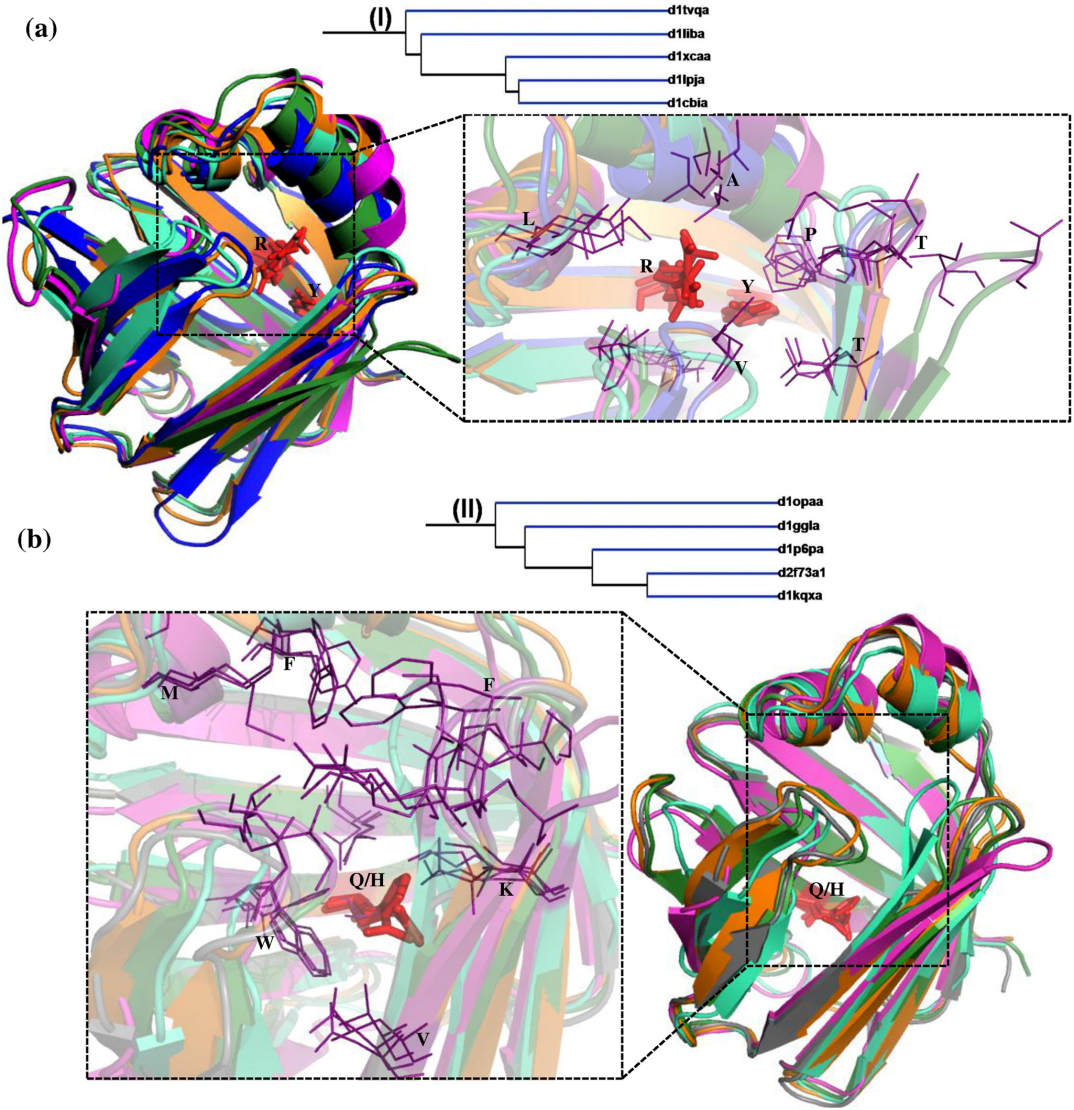
Structural superimpositions of lipocalin domains in cluster I (a) and cluster II (b)corresponding to Fatty acid binding protein-like family. **(a)** Structural superimposition of liver basic fatty acid binding protein (LB-FABP) (*Gallus gallus*) (d1tvqa_) (orange), Adipocyte lipid binding protein (d1liba_) (blue), Cellular retinoic acid binding protein (CRABP) (d1xcaa_) (pink), Cellular retinol binding protein (CRBP) IV (d1lpja_) (cyan) and CRABP I (d1cbia_) (green). **(b)** Structural superimposition of CRBP II (*Rattus rattus*) (d1opaa_) (orange), CRBP III (d1ggla_) (grey), LB-FABP (*Rhinella arenarum*) (d1p6pa_) (pink), Liver FABP (d2f73a1) (cyan), CRBP III (*Danio rerio*) (d1kqxa_) (green). The ligand binding region is represented as dotted line box. The residues in the binding regions are shown as purple lines in the closer view. The ligand interacting residues are represented as red sticks. This Fig and all other structural superimposition Figs have been generated using Pymol [31].

**Fig 6:**
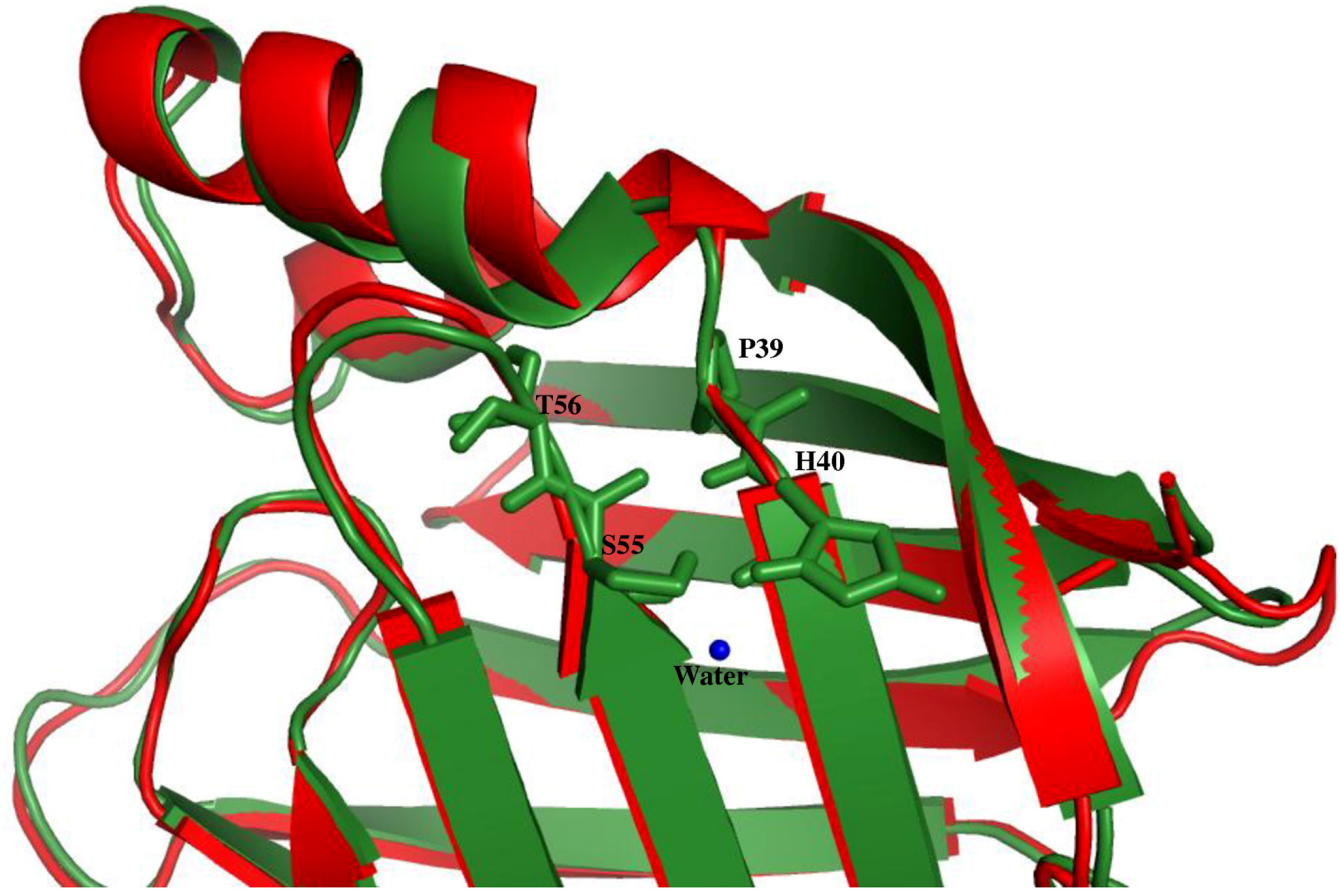
Structural superimpositions of cellular retinoic acid binding proteins in its ligand bound (2CBS) (red) and unbound (1CBI) (green) forms. Water molecule is shown as blue sphere.

The members of FABP-like family are carriers of fatty acids and other lipophilic substances like retinoid and facilitate the transfer of fatty acids between intra and extra cellular membranes [24, 25]. In the structure-based phylogenetic tree, two clusters of FABP-like family consisting of 5 members in each have been obtained. Cluster I comprise liver basic FABP (*Gallus gallus*), adipocyte lipid binding protein, cellular retinoic acid binding protein (CRABP), cellular retinol binding protein (CRBP) IV and CRABP I (Fig 3). Cluster II comprise CRBP II (*Rattus rattus*), CRBP III, Liver basic FABP (*Rhinella arenarum*), Liver FABP and CRBP II (*Danio rerio*). The members of both the clusters bind fatty acids, retinol and retinoic acid. Interestingly, the members are clustered based on their mode of interaction with the ligand. Branching of the members in the tree is consistent with the nature of the hydrophobic patch in the barrel. This has been illustrated in detail with the examples (Figs 5 and 6).

The lipid and fatty acid binding proteins in cluster I, branches out from the main node whereas retinol and retinoic acid binding protein cluster much closer within cluster I. Similarly within cluster II, CRBP II and III separates out from other three members. The structural superposition of each of the 5 members in the clusters I and II is shown in Fig 5a and 5b respectively. In the cartoon representation of the structural superposition the ligand interacting residues are shown as sticks (red). In all the 5 cases of cluster I (Fig 5a) the ligand interacting residues are Arg and Tyr. The structural superimposition of the hydrophobic pocket for all in cluster I is shown as purple lines. The common residues in all the members are marked and it clearly shows that there are slight differences in the features of hydrophobic residues. In cluster I the α-helix is involved in the ligand binding and the interaction is water mediated. For example, the structural superimposition of ligand bound (2CBS) (red) [26] and unbound (1CBI) (green) [27] forms of cellular retinoic acid binding protein is shown (Fig 6). The slight displacement of helix can be clearly noted. The residues S55, T56, P39 and H40 are involved in hydrogen bond interaction with the water molecule in its ligand unbound form. The water molecule is released and the helix is moved to the closed conformation when the ligand binds to the lipocalin. In cluster II (Fig 5b) the main ligand interacting residue is Gln (in 4 out of 5 cases, with the fifth being His). But in all these cases the nature of the interaction (hydrogen bond) is maintained. The hydrophobic pocket residues differ in most of the cases in cluster II but a few hydrophobic residues like Val and Phe are conserved. Though all the 10 members in cluster I and II belong to FABP-like family, they form different clusters corresponding to different modes of binding. In the single outlier corresponding to muscle fatty acid binding protein (d1ftpa_) [28], in addition to not having above mentioned structural features the ligand-binding site in this case is a helix-turn-helix motif and this feature is different from other members. So, this outlier lacks the structural features that other members have causing it to cluster away from the FABP-like clusters.

### Retinol binding protein-like

Retinol binding protein-like (RBP-like) family comprises mainly of retinol binding proteins, allergen and odorant binding proteins. As mentioned earlier, the RBP-like protein transport a variety of small molecules relevant in various biological processes. In the present structure-based phylogenetic tree the RBP-like proteins form four major clusters apart from four outliers. The four clusters are represented as III, IV, V and VI (Fig 3).

### Cluster IV and Cluster V

**Fig 7:**
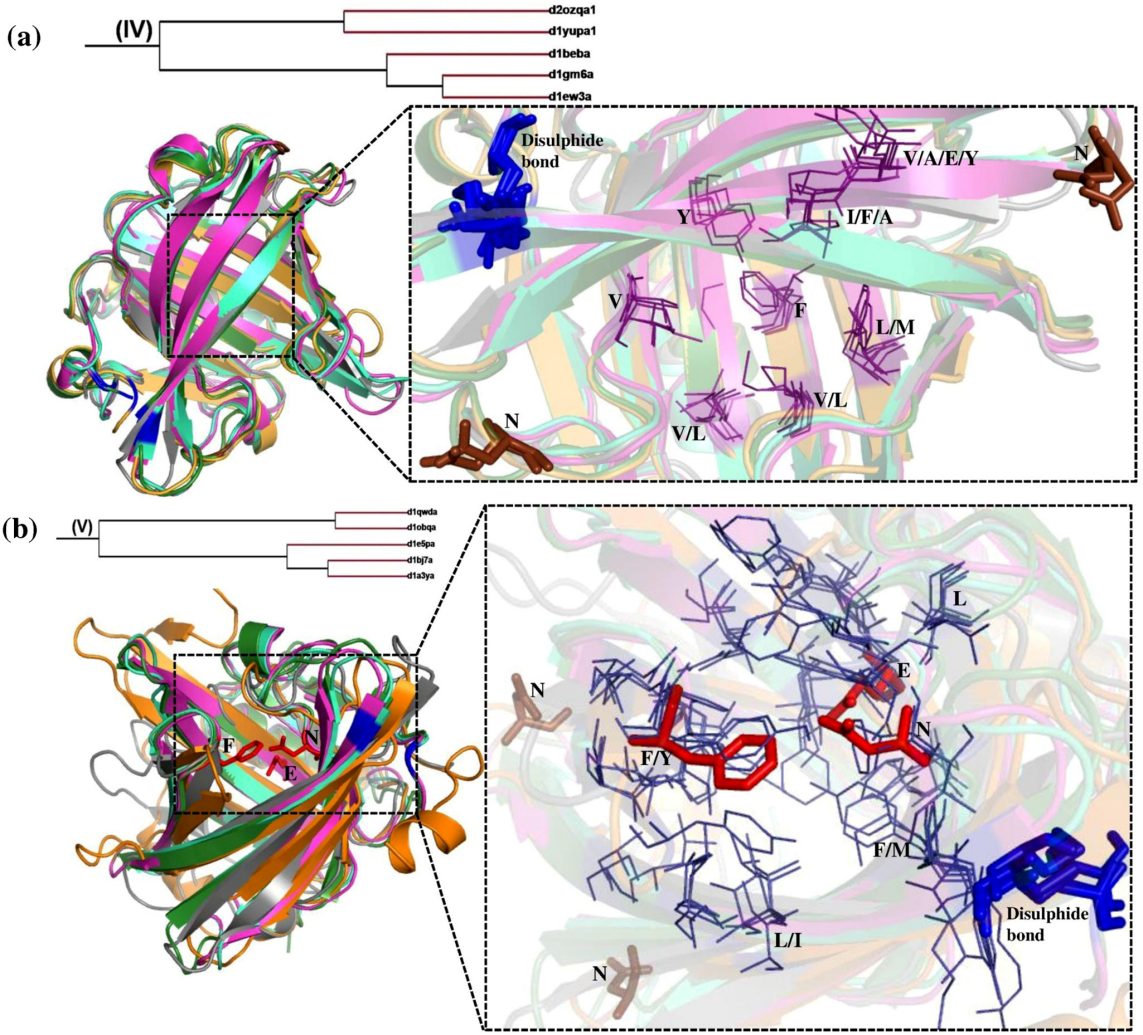
Structural superimposition of lipocalin domains in cluster IV (a) and cluster V (b) corresponding to Retinol protein binding protein-like family. **(a)** Structural superimposition of *Bovine* β-lacto globulin (d1beba_) (green), salivary lipocalin (d1gm6a_) (cyan), Major horse allergen (d1ew3a_) (pink), Major urinary protein (d2ozqa1) (grey) and *Reindeer* β-lacto globulin (d1yupa1) (orange). **(b)** Structural superimposition of Pheromone binding protein (d1e5pa_) (pink), Lipocalin allergen (d1bj7a_) (cyan), Odorant binding protein (d1a3ya_) (green), Lipoprotein Blc (d1qwda_) (grey) and α-crustacyanin (d1obqa_) (orange). The ligand binding region is represented as dotted line box. The residues in the binding regions are shown as purple lines in the closer view. The ligand interacting residues are represented as red sticks. The disulphide bond is represented as blue sticks. Asn that is involved in glycosylation are shown as brown sticks.

Cluster IV and V consists of five members each. The structural superimposition of the members of cluster IV and V are shown in Fig 7a and 7b respectively. The hydrophobic patch in the ligand binding pocket is shown in a close-up view for the two clusters. Cluster IV groups β-lacto globulins (Bovine and Reindeer), salivary lipocalin, horse allergen and Major urinary protein. Cluster V comprise pheromone and odorant binding proteins, allergens, lipoprotein Blc and α-crustacyanin. The lipocalin proteins in both the clusters bind hydrophobic molecules. The members in cluster IV bind smaller molecules like 2-(sec-butyl)thiazole and 6-hydroxy-6-methyl-hepta-3-one with molecular weight of 141and 144 g/mol respectively, whereas members in cluster V bind a larger molecule like astaxanthin with the molecular weight 596 g/mol. In both of these clusters, there are two Cys residues which are conserved in all the members and are involved in a disulphide bond, shown (Fig 7). The salivary lipocalin, allergen and pheromone binding proteins found in these clusters consist of the glycosylation sites (brown sticks) and putative IgE binding sites that are crucial for its function. In each of these clusters IV and V there are two sub-clusters with three and two members respectively. The reason for sub-clusters in cluster V is perhaps an insertion of 10 and 20 residues in the two cases respectively. But in cluster IV though the two members lack four hydrophobic interactions and two glycosylation sites, the type of ligand that it binds and their ligand binding pockets are similar which is consistent with them clustering together.

Though most of the characteristics of cluster IV and V are similar, there is a significant difference in their functions which separates them into two different clusters. It is evident (Fig 7) that in cluster IV the hydrophobic residues are conserved in most of the cases and orientation of these binding pocket residues are similar. Almost all the members in cluster IV have a dimerisation interface that is important for its function which is not found in cluster V. In cluster V there are three ligand interacting residues (Phe, Glu and Asn) whereas in cluster IV there is only hydrophobic interaction. It is interesting to note that features of ligand interaction are different in these clusters.

### Cluster III and Cluster VI

**Fig 8:**
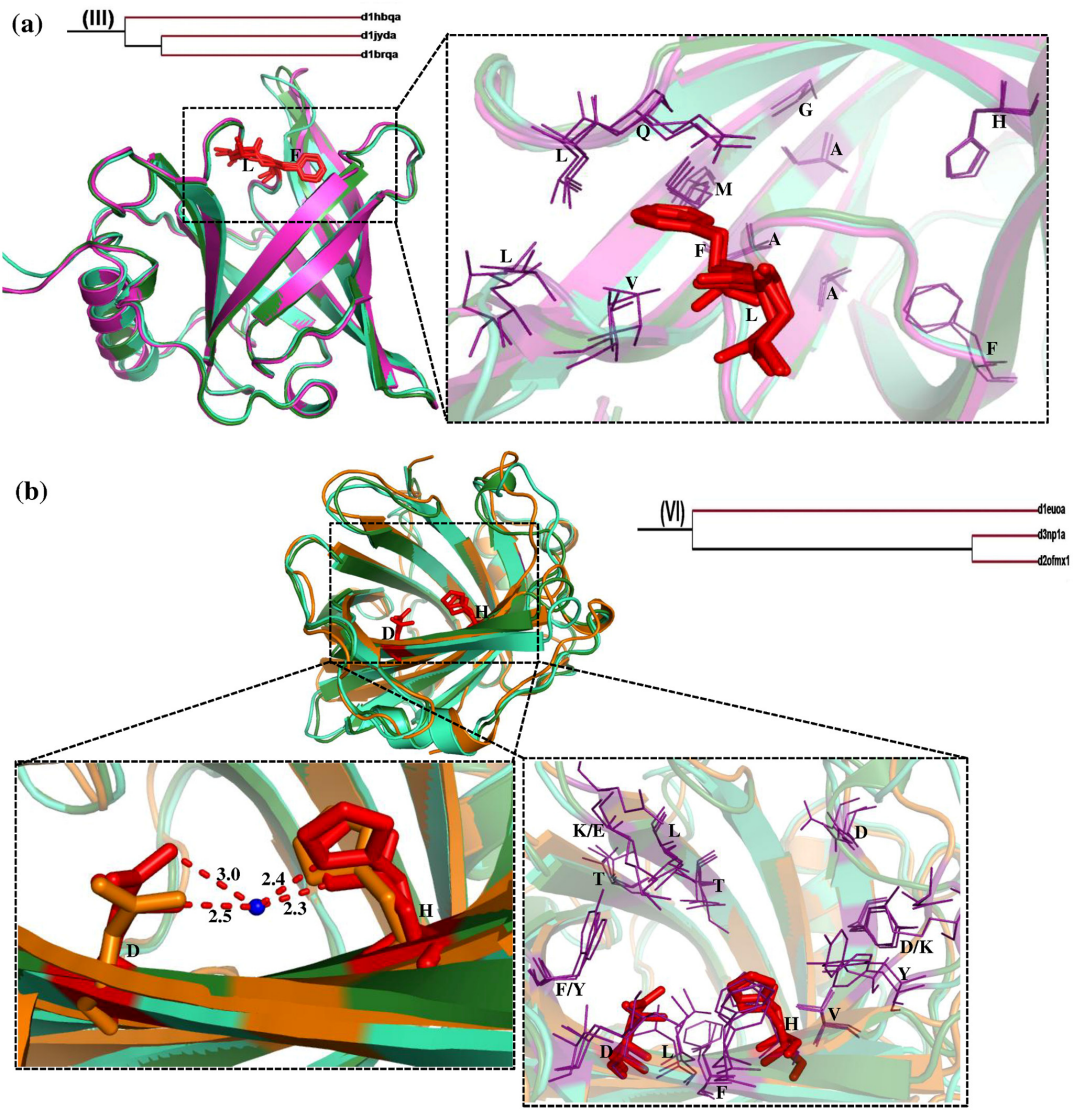
Structural superimposition of lipocalin domains in cluster III (a) and cluster VI (b) corresponding to Retinol protein binding protein-like. **(a)** Structural superposition of *Bovine* plasma RBP (d1hbqa_) (pink), *Human* serum RBP (d1jyda_) (cyan) and *Human* plasma RBP (d1brqa_) (green). **(b)** Structural superposition of Nitrophorin2 (d1euoa_) (orange), Nitrophorin1 (d3np1a_) (cyan) and Nitrophorin4 (d2ofmx1) (green). The water molecule is represented as blue sphere. The ligand binding region is represented as dotted line box. The residues in the binding regions are shown as purple lines in the closer view. The ligand interacting residues are represented as red sticks.

Cluster III and cluster VI contains three members each. The structural superimposition of members of each of these two clusters are shown in Fig 8a and 8b corresponding to cluster III and VI respectively. Cluster III comprise retinol binding proteins (human serum, human plasma and bovine plasma) in which Phe and Leu are the ligand interacting residues in all the cases. In spite of three retinol binding proteins being related in terms of sequence and structure, the source from which they are obtained is different. Also it is clear from the phylogenetic tree (Fig 3) that in cluster III both the human proteins cluster together whereas the bovine protein branches out separately. All three perform the same function (retinol transport) and are involved in macromolecular complexation. Also from Fig 8a it is evident that both ligand interacting residues and the hydrophobic patch are conserved.

Cluster VI groups three Nitrophorins (NP) (1, 2 and 4) together. The main function of these proteins is to transport of nitric oxide (NO), bind histamine and act as an anticoagulant. The NP-NO complex with histamine bound to it transports heme by forming a coordination bond with the heme moiety. The heme coordination is a water mediated interaction that is conserved among these members and the important interacting residues are His and Asp which are shown as red sticks (Fig 8b). The distances between the water molecule and the interacting residues are provided (Fig 8b). Most of the residues in the hydrophobic pocket are conserved while a few residues are substituted by chemically equivalent residues.

The outlier close to cluster VI is porcine β-lacto globulin (d1exsa_) that is different from other β-lacto globulins. It has been also shown in earlier studies that porcine β-lacto globulin unlike other β-lacto globulins does not bind any small hydrophobic ligand, but is involved in domain swapping [29]. Structure of porcine β-lacto globulin lacks the structural properties in the ligand binding site of other β-lacto globulins which is the likely reason for it to appear as an outlier. It is evident that most of the lipocalins in RBP-like family cluster based on their main function and their various modes of ligand binding.

### Inferences on the functions of domains of unknown function

**Fig 9:**
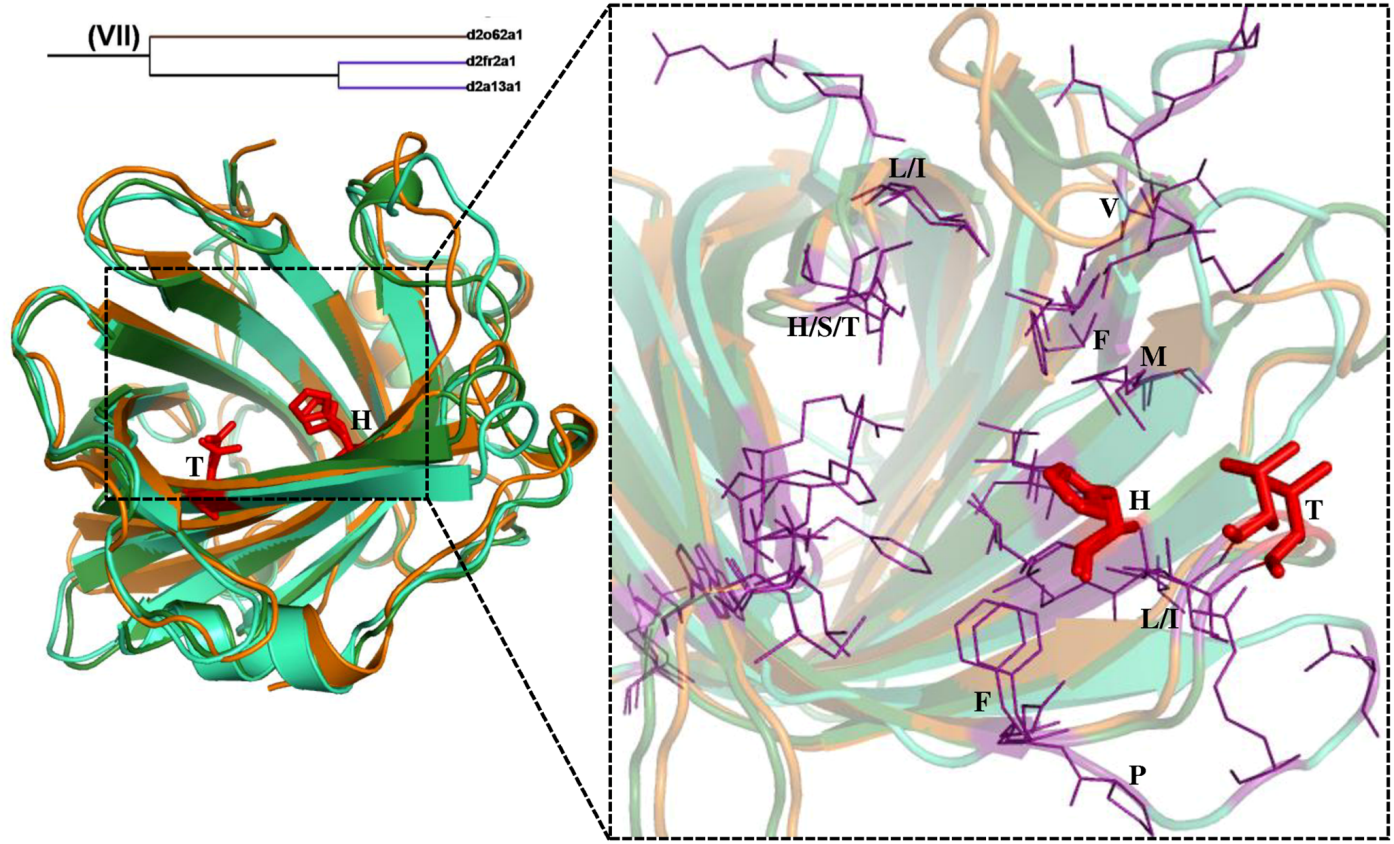
Structural superimposition of lipocalin domains in cluster VII corresponding to RV2717c-like and all 1756-like families. The ligand binding region is represented as dotted line box. The residues in the binding regions are shown as purple lines in the closer view. The ligand interacting residues are represented as red sticks. Shown are the superimposition of Uncharacterized protein DUF 3598 (*Nostoc punctiforme*) (d2o62a1) (cyan), Rv2717c DUF 1794 (*M*. *Tuberculosis*) (d2fr2a1) (orange) and Nitrophorin-like heme binding protein (*Arabidopsis Thaliana*) (d2a13a1) (green).

Cluster VII groups two different families "Rv2717c-like" and "All 1756-like" from the lipocalin superfamily. This cluster comprise an uncharacterized protein (DUF 3598), Rv2717c (DUF 1794) and a Nitrophorin-like protein. DUFs are the protein domains with their functions not identified yet. The structural superimposition of examples in this cluster is shown (Fig 9). It is clear from Fig 9. that both the DUFs are structurally quite similar to Nitrophorin-like protein. In fact the heme interacting residues His and Thr from Nitrophorin-like protein are conserved in the DUFs. Also most of the residues in the hydrophobic patch are conserved. This shows that similar hydrophobic patches are observed in DUFs as well.

**Fig 10:**
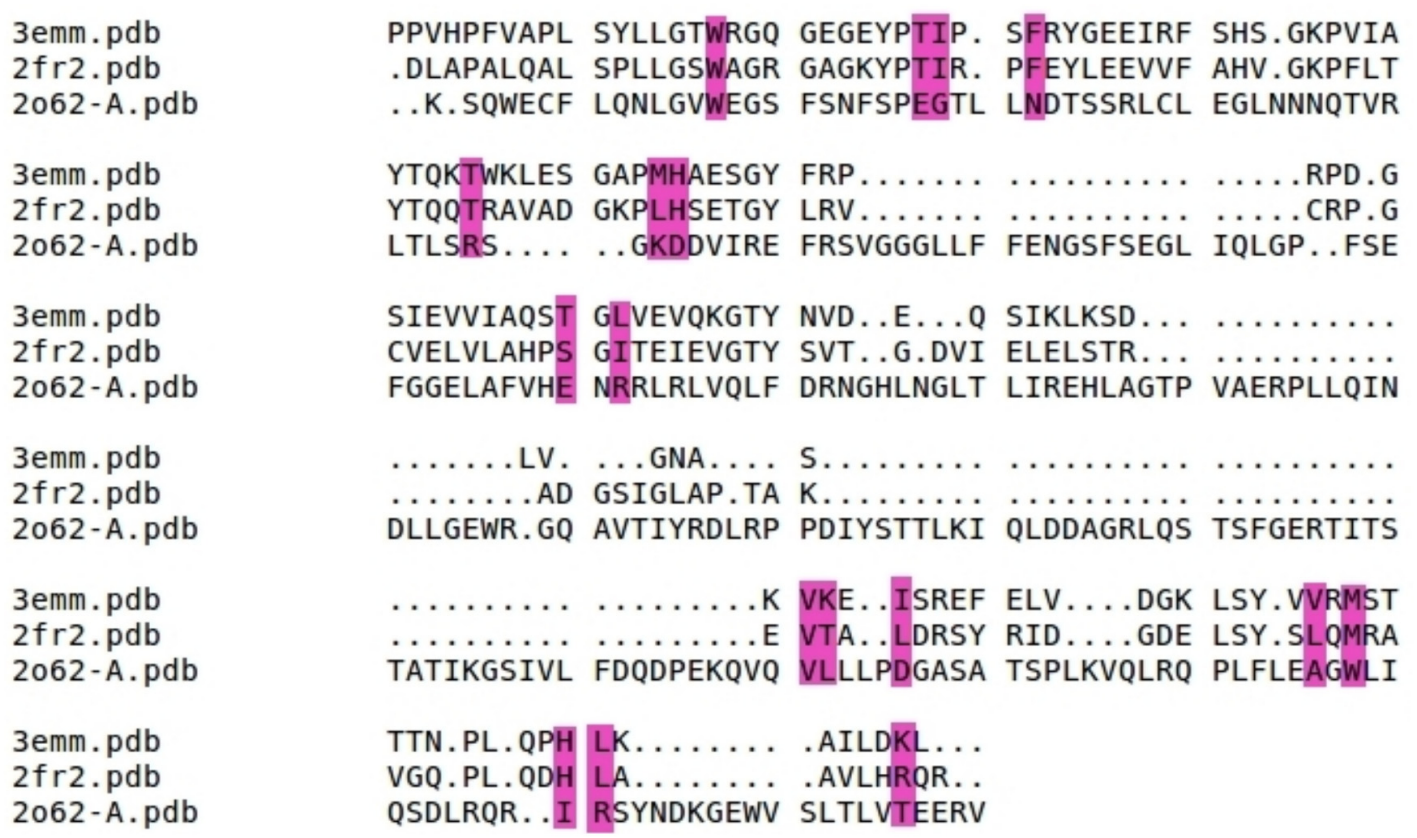
Structure-based sequence alignment between the DUF1794 (2FR2), DUF 3598 (2O62) and Nitrophorin-like heme binding protein (3EMM) in heme bound form. The residues that are important for heme binding are highlighted in pink color.

**Fig 11:**
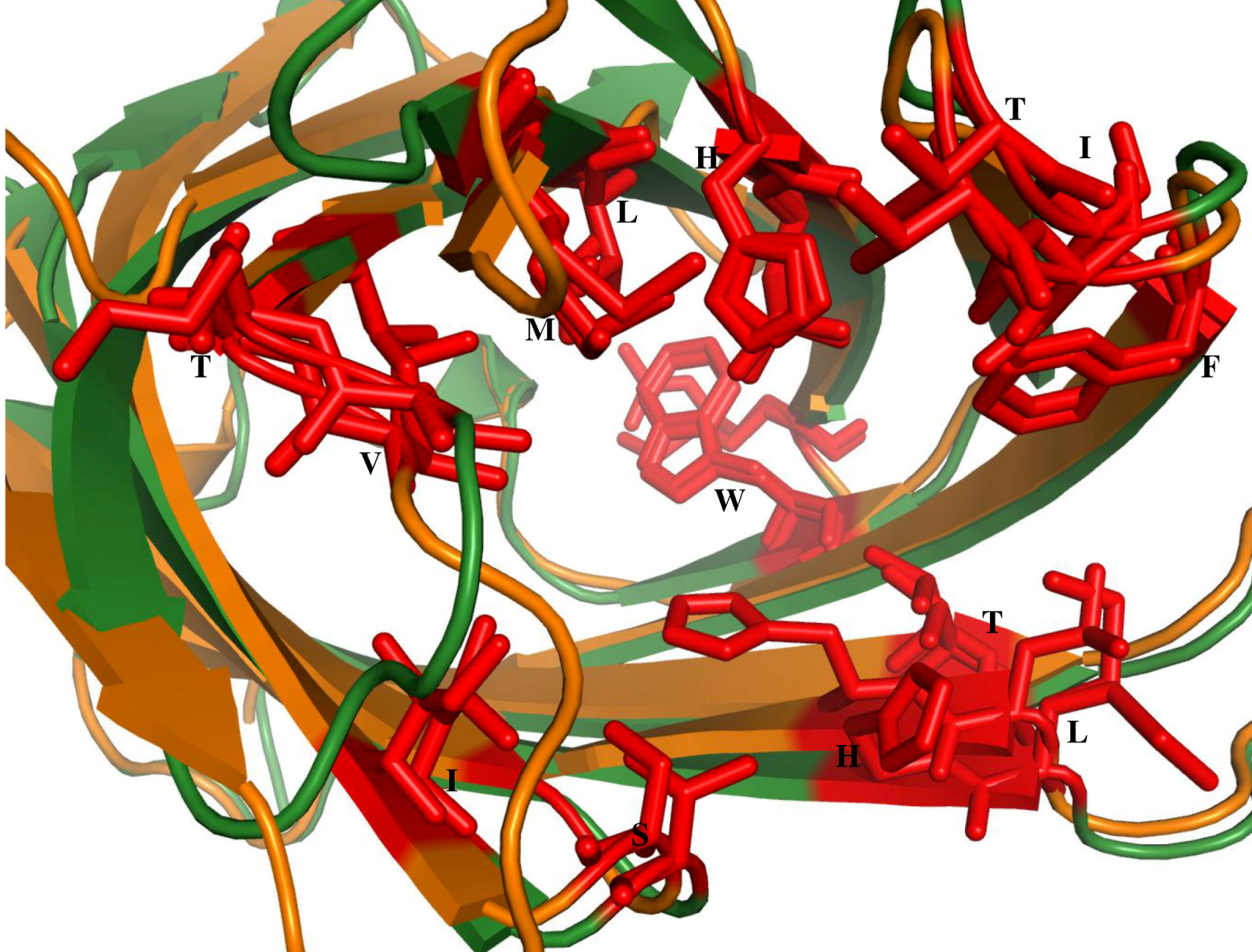
Structural superimposition of DUF1794 (2FR2) (orange) and its homologue Nitrophorin-like heme binding protein (2A13) in heme unbound form (green). The residues that are important for heme binding are represented as red sticks.

The two DUFs are compared to Nitrophorin-like protein in heme bound (3EMM) [30] form both in terms of their 3-D structures and sequences. It is interesting to note that DUF 1794 (2FR2) from *M*.*Tuberculosis* and DUF 3598 (2O62) from *Nostocpunctiforme* are highly similar in their 3-D structure to 3EMM but their corresponding sequence identity is only 34% and 12% respectively. The structure-based alignment among these three proteins is shown (Fig 10). The important residues are highlighted in pink color. It can be noted that these residues are identical between Nitrophorin-like protein (3EMM) [30] and DUF 1794 (2FR2). But in DUF 3598 (2O62), though the residues are not conserved their 3-D structures show remarkable similarity. The structural superimposition of DUF 1794 (2FR2)with the Nitrophorin-like protein (2A13) [30],in heme unbound form, is shown (Fig 11). It is evident from the Fig 11 that binding pocket and interacting residues (shown in red stick representation) are conserved in DUF 1794 as in Nitrophorin-like protein. It is clear from cluster VII that the members in this cluster have similar binding sites for heme. So it is tempting to conclude that the DUF 1794 (2FR2) from *M*.*Tuberculosis* have putative heme binding residues and DUF 3598 (2O62) from *Nostoc punctiforme* has binding sites similar to Nitrophorin-like protein.

## Conclusions

The superfamily of Lipocalins is known for its high sequence divergence and high structural similarity. Earlier phylogenetic analyses provided important insights into the history of changing functions of the lipocalins [13]. However sequence-based phylogeny studies are fraught with uncertainty in the case of lipocalins as they are highly diverged at the level of sequences. For example, in one of the previous studies [8] it was shown that highest sequence identity of prostaglandin D synthase is with α1 microglobulin and is less than 30% though these two proteins are most closely clustered in the phylogenetic tree. However these two proteins differ in terms of enzymatic properties and association with membranes. The close clustering in the dendrogram based on highest sequence identity is not consistent with biochemical and cellular localization properties. The ligand binding properties of lipocalins depend on chemical nature and spatial orientation of residues which bind to the ligand. However, sequence-based alignment may not correctly align the ligand binding residues from the homologous lipocalins as the sequence identity is low and it leads to uncertain or misleading conclusions on the functional similarity or differences between lipocalins.

Therefore, the current study has been made using the structure-based phylogenetic approach which led to a better understanding of the lipocalin functional similarity and diversity. The structure-based phylogenetic tree in the current study reveals that the lipocalin proteins cluster based on their functional similarity. Specifically, it is interesting to note that the clustering is related to the modes of their ligand binding. Though the overall functions of each cluster is similar, the sub-clusters in each of these clusters gives a clue on the residue level differences in the ligand binding region. Also, it is clear that most of the outliers correspond to single member families.

Previous studies [8–14] on the phylogeny of lipocalins based on sequence comparisons derived evolutionary models for lipocalins, discussed design of novel lipocalins with catalytic properties and suggested that the recently evolved lipocalins show high rates of amino acid substitutions, bind smaller hydrophobic ligands and evolve with improved binding. However, earlier studies did not discuss the structural equivalence of functional residues and their implications in identifying structurally or functionally close lipocalins and these aspects form the focus of the present study.

The present phylogenetic analysis not only revealed the structural basis for ligand association but also the functional diversity of lipocalins. The current analysis also sheds light on the functions of the hypothetical proteins for which the function is not been characterized. The clustering and structural similarity of DUF 1794 and DUF 3598 with the nitrophorins shows that the DUFs have possible similar binding sites. Particularly the comparison of DUF 1794 (*M*.*Tuberculosis*) with the Nitrophorin-like proteins suggests that the DUF 1794 has the putative heme binding sites due to which it could perform a similar function. So the current approach could help in the identification of functions of lipocalin proteins newly discovered from genome sequencing and structural genomics projects. It is evident from this study that the structure-based phylogenetic analysis could be a useful tool, even for other protein families/superfamilies, in understanding the functional similarity between the homologous proteins with poor sequence identity however with high structural similarity.

## Supporting Information

S1 Table List of 39 lipocalin domains considered for analysis. (DOCX)

S1 Text Structure-based pairwise alignment for 741 pairs of lipocalins using DALI. (DOCX)

S2 Text Multiple sequence alignment for 39 lipocalin domains using clustalW2. (DOC)
