# Supplementary material for: Structure-Based Phylogenetic Analysis of the Lipocalin Superfamily
Source: PLoS One. 2015 Aug 11;10(8):e0135507. doi: 10.1371/journal.pone.0135507 (PMC4532494; doi:10.1371/journal.pone.0135507)
Supplement: S1 Table — (DOCX) [file pone.0135507.s001.docx]

**S1 Table:** List of 39 lipocalin domains considered for analysis.

| **S. No** | **SCOP domain code** | **PDB code** | **Protein name** | **Organism** | **SCOP Family name** |
| --- | --- | --- | --- | --- | --- |
| 1 | d1brqa_ | 1RBQ | Human plasma retinol binding protein (RBP) | Homosapiens | Retinol binding protein like (b.60.1.1) |
| 2 | d1jyda_ | 1JYD | Human serum retinol binding protein (RBP) | Homosapiens |  |
| 3 | d1hbqa_ | 1HBQ | Bovine plasma retinol binding protein (RBP) | Bos taurus |  |
| 4 | d1ew3a_ | 1EW3 | Major horse allergen EQU C1 | Equus caballus |  |
| 5 | d1gm6a_ | 1GM6 | Salivary lipocalin | Sus scrofa |  |
| 6 | d1beba_ | 1BEB | Bovine beta- lactoglobulin | Bos taurus |  |
| 7 | d1yupa_ | 1YUP | Reindeer beta- lactoglobulin | Rangifer tarandus |  |
| 8 | d2ozqa1 | 2OZQ | Major urinary protein | Mus musculus |  |
| 9 | d1xkia_ | 1XKI | Von Ebner's gland protein (VEGP, tear lipocalin) | Homosapiens |  |
| 10 | d1a3ya_ | 1A3Y | Odorant binding protein | Sus scrofa |  |
| 11 | d1bj7a_ | 1BJ7 | Bovine Lipocalin allergen BOS D 2 | Bos taurus |  |
| 12 | d1e5pa_ | 1E5P | Aphrodosin, a sex pheromone | Golden hamster (Mesocricetus auratus) |  |
| 13 | d1obqa_ | 1OBQ | Alpha-crustacyanin | Homarus gammarus |  |
| 14 | d1qwda_ | 1QWD | Outer membrane lipoprotein Blc | Escherichia coli |  |
| 15 | d2ofmx1 | 2OFM | Nitrophorin4 | Rhodnius prolixus |  |
| 16 | d1epaa_ | 1EPA | Epididymal retinoic acid binding protein | Rattus norvegicus |  |
| 17 | d1iw2a_ | 1IW2 | Complement protein C8 gamma | Homosapiens |  |
| 18 | d1exsa_ | 1EXS | Procine beta- lactoglobulin | Sus scrofa |  |
| 19 | d1euoa_ | 1EUO | Nitrophorin 2 (prolixin-s) | Rhodnius prolixus |  |
| 20 | d3np1a_ | 3NP1 | Nitrophorin 1 | Rhodnius prolixus |  |
| 21 | d1cbia_ | 1CBI | Cellular retinoic acid binding protein (CRABP I) | Mus musculus | Fatty acid binding protein like (b.60.1.2) |
| 22 | d1lpja_ | 1LPJ | Cellular retinol binding protein (CRBP IV) | Homosapiens |  |
| 23 | d1xcaa_ | 1XCA | Cellular retinoic acid binding protein (CRABP) | Homosapiens |  |
| 24 | d1liba_ | 1LIB | Adipocyte lipid binding protein (ALPB) | Mus musculus |  |
| 25 | d1tvqa_ | 1TVQ | Liver basic fatty acid binding protein (LB FABP) | Gallus gallus |  |
| 26 | d1kqxa_ | 1KQX | Cellular retinoic acid binding protein (CRBP II) | Danio rerio |  |
| 27 | d2f73a1 | 2F73 | Liver fatty acid binding protein | Homosapiens |  |
| 28 | d1p6pa_ | 1P6P | Liver basic fatty acid binding protein (LB FABP) | Rhinella arenarum |  |
| 29 | d1ggla_ | 1GGL | Cellular retinol binding protein (CRBP III) | Homosapiens |  |
| 30 | d1opaa_ | 1OPA | Cellular retinol binding protein (CRBP II) | Rattus rattus |  |
| 31 | d1ftpa_ | 1FTP | The muscle fatty acid binding protein | Schistocerca gregaria |  |
| 32 | d1avgi_ | 1AVG | Thromin inhibitor | Triatoma pallidipennis | Thromin inhibitor (b.60.1.3) |
| 33 | d1oeja_ | 1OEJ | Hypothetical protein YodA | E.coli | Hypothetical protein YodA (b.60.1.4) |
| 34 | d1r0ua_ | 1R0U | Hypothetical protein YwiB | Bacillus subtilis | Hypothetical protein YwiB (b.60.1.5) |
| 35 | d2gc9a1 | 2GC9 | P-coumaric acid decarboxylase (PDC) | Lactobacillus plantarum | Phenolic acid decarboxylase (PAD) (b.60.1.6) |
| 36 | d1vpra1 | 1VPR | Dinoflagellate luciferase | Lingulodinium polyedrum (algae) | Dinoflagellate luciferase repeat (b.60.1.7) |
| 37 | d2a13a1 | 2A13 | Nitrobindin (heme-rim of the cavity) | Arabidopsis Thaliana | Rv2717c -like (b.60.1.8) |
| 38 | d2fr2a1 | 2FR2 | Rv2717c (DUF 1794) | Mycobacterium Tuberculosis |  |
| 39 | d2o62a1 | 2O62 | Uncharacterised protein (DUF 3598) | Nostoc punctiforme | All1756-like (b.60.1.9) |
