# Supplementary material for: Structure-Based Phylogenetic Analysis of the Lipocalin Superfamily
Source: PLoS One. 2015 Aug 11;10(8):e0135507. doi: 10.1371/journal.pone.0135507 (PMC4532494; doi:10.1371/journal.pone.0135507)
Supplement: S1 Text — (DOCX) [file pone.0135507.s002.docx]

**S1 Text:** Structure-based pairwise alignment for 741 pairs of lipocalins using DALI.

d1a3ya_ ----------------pfELSGKWITSYI---------------------gssdlekIGENApfqvfmrsiefddkeskvylnffskengICEEFSLIGTK-QEGNTYDVNY------AGNNKFVVSYAS-ETALIISNINVDEEGDKTIMTGLLGKGTDiedqdlekfkevtrengipeenivniiERDDCPA--

d1avgi_ aegddcsiekamgdfkpeEFFNGTWYLAHgpgvtspavcqkfttsgskgftqiveigYNKFE----------------------------SNVKFQCNQVDnKNGEQYSFKCkssdntEFEADFTFISVSyDNFALVCRSITFTSQPKEDRYLVFERTKS--------------------------dTDPDAKEic

d1a3ya_ -------pfELSGKWITSYIGSSDLEKIG-ENAPFQVFMRSIEFDdKESKVYLNFFSKENGICEEFSLIGTKQEG-NTYDVNYAGNNKFVVSYAS-ETALIISNINVdeeGDKT--IMTGLLGKGTDIEDQDLEKFKEVTRENGIPeeNIVNII--ERDDCPa

d1beba_ qtmkgldiqKVAGTWYSLAMAASDISLLDaQSAPLRVYVEELKPT-PEGDLEILLQKWENGECAQKKIIAEKTKIpAVFKIDALNENKVLVLDTDyKKYLLFCMENS---AEPEqsLVCQCLVRTPEVDDEALEKFDKALKALPMH--IRLSFNptQLEEQC-

d1a3ya_ --pFELSGKWITSYIGSSDLEKIGENAPFQVFMRSIEFDDKeSKVYLNFFSKENGICEEFSLIGTKQEGNTYDVNYAGNNKFVVSYASETALIISNINVDeEGDKTIMTGLLGKGTDIEDQDLEKFKEVTRENGIPEENIVNIIERDDCPA

d1bj7a_ idpSKIPGEWRIIYAAADNKDKIVEGGPLRNYYRRIECINDcESLSITFYLKDQGTCLLLTEVAKRQEGYVYVLEFYGTNTLEVIHVSENMLVTYVENYD-GERITKMTEGLAKGTSFTPEELEKYQQLNSERGVPNENIENLIKTDNCPP

d1a3ya_ ----------------pfELSGKWITSYIGSsDLEKigenaPFQV-FMRSIEFDdKESKVYLNFFSKE-----NGICEEFSLIGTKQEGN-TYDVNY--------AGNNKFVVSYAS-ETALIISNINV--deegdKTIMTGLLGKGTDI-EDQDLEKFKEVTRENGIPEeNIVNIieRDDCPA

d1brqa_ erdcrvssfrvkenfdkaRFSGTWYAMAKKD-PEGL-----FLQDnIVAEFSVD-ETGQMSATAKGRVrllnnWDVCADMVGTFTDTEDPaKFKMKYwgvasflqKGNDDHWIVDTDyDTYAVQYSCRLlnldgtcADSYSFVFSRDPNGlPPEAQKIVRQRQEELCLAR-QYRLI--VHNGYC

d1a3ya_ pfELSGKWITsYIGSSD--------------lekiGENApfQVFMrSIEFDDkeSKVYLNFFSKengiCEEFSLIGTK-------------------QEGN-TYDVN--------yaGNNKFVVSYasetaLIISNINVDeEGDKTIMTGLLGkgtdiedqdlekfkevtrengipeenivniierddcpa

d1cbia_ --PNFAGTWK-MRSSENfdellkalgvnamlrkvaVAAA-sKPHV-EIRQDG--DQFYIKTSTT----VRTTEINFKVgegfeeetvdgrkcrslptWENEnKIHCTqtllegdgpkTYWTRELAN-----DELILTFGA-DDVVCTRIYVRE--------------------------------------

d1a3ya_ pfELSGKWITSYIGSSDLEKIGENAPFQVFMRSIEFDDKeSKVYLNFFSKENGICEEFSLIGTKQEGNTYDVNYAGNNKFVVSYASETALIISNINVDEEGDKTIMTGLLGKGTDIEDQDLEKFKEVTRENGIPEENIVNIIERDDCPA

d1e5pa_ faELQGKWYTIVIAADNLEKIEEGGPLRFYFRHIDCYKNcSEXEITFYVITNNQCSKTTVIGYLKGNGTYETQFEGNNIFQPLYITSDKIFFTNKNXDRAGQETNXIVVAGKGNALTPEENEILVQFAHEKKIPVENILNILATDTCPE

d1a3ya_ -----pFELSGKWITSYIGSSdlekigenapfqvFMRSIEFDdkESKVYLNFFSKENGICEEFSLIGTKQEG-NTYDVNY-aGNNKFVVSYAS-ETALIISNINVDEeGDKTIMTGLLGKGTDIEDQDLEKFKEVTRENGIPEENIVNIIERDDCPA------

d1epaa_ vkdfdiSKFLGFWYEIAFASK---------eekmGAMVVELK--ENLLALTTTYYSEDHCVLEKVTATEGDGpAKFQVTRlsGKKEVVVEATDyLTYAIIDITSLVA-GAVHRTMKLYSRSLDDNGEALYNFRKITSDHGFSETDLYILKHDLTCVKvlqsaa

d1a3ya_ --------------pFELS-GKWITSYIGSSDLEkiGENApfQVFMrSIEFDDKESKVYLNFFSKENGICEEFSLIGTKQE--GNTYDVNY---------------aGNNKFVVSYASETALIISNINVDEEGDKTIMTGLLGK-GTDI-EDQDLEKFKEVTRengiPEENIVNIIER-DDCPA-----

d1euoa_ mdcstnispkqgldkAKYFsGKWYVTHFLDKDPQ--VTDQ--YCSS-FTPRESDGTVKEALYHYNANKKTSFYNIGEGKLEssGLQYTAKYktvdkkkavlkeadekNSYTLTVLEADDSSALVHICLREGSKDLGDLYTVLTHqKDAEpSAKVKSAVTQAGL----QLSQFVGTKDLgCQYDDqftsl

d1a3ya_ -------pFELSGKWITSYIGSSDLEKIGENAPFQVFMRSIEFDdKESKVYLNFFSKENGICEEFSLIGTKQ-EGNTYDVNYAGNNKFVVSYA-SETALIISNINVDeEGDKTIMTGLLGKGTDIEDQDLEKFKEVTRENGIPEENIVNIIER-DDCPA--

d1ew3a_ vairnfdiSKISGEWYSIFLASDVKEKIEENGSMRVFVDVIRAL-DNSSLYAEYQTKVNGECTEFPMVFDKTeEDGVYSLNYDGYNVFRISEFeNDEHIILYLVNFD-KDRPFQLFEFYAREPDVSPEIKEEFVKIVQKRGIVKENIIDLTKIdRCFQLrg

d1a3ya_ -----------pFELSGKWITSYIGSS-DLEKigenAPFQ-VFMRSIEFDdKESKVYLNFFSKENGICEEFSLIGTKQ-EGNTYDVNYAGNNKFVVSYAS-ETALIISNINVdeeGDKT--IMTGLLGKGTDIEDQDLEKFKEVTRENGIPEeNIVNiIERDDCPA

d1exsa_ vevtpimteldtQKVAGTWHTVAMAVSdVSLL-dakSSPLkAYVEGLKPT-PEGDLEILLQKRENDKCAQEVLLAKKTdIPAVFKINALDENQLFLLDTDyDSHLLLCMENS---ASPEhsLVCQSLARTLEVDDQIREKFEDALKTLSVPM-RILPaQLEEQCRV

d1a3ya_ -pFELSGKWITSYIGS--SDLE------kiGENApFQVFMRSIEFDDK-ESKVYLNFFskengiCEEFSLIGTKQE-----------------------gNTYDvnyaGNNKFVVsYASETALIISNINVdeegdKTIMTGLLGKGtdiedqdlekfkevtrengipeenivniierddcpa

d1ftpa_ vkEFAGIKYKLDSQTNfeEYMKaigvgaieRKAG-LALSPVIELEILDgDKFKLTSKT------AIKNTEFTFKLGeefdeetldgrkvkstitqdgpnkLVHEqkgdHPTIIIR-EFSKEQCVITIKLG-----DLVATRIYKAQ------------------------------------

d1a3ya_ pFELSGKWITSYIGS--SDLE------kiGENAPfQVFMRSIEFDDkeSKVYLNFFSKengiCEEFSLI--------------------gtKQEG---NTYD-vnyagNNKFVVsYASETALIISNINVdeegdKTIMTGLLGKGtdiedqdlekfkevtrengipeenivniierddcpa

d1ggla_ pPNLTGYYRFVSQKNmeDYLQalnislavRKIALlLKPDKEIEHQG--NHMTVRTLST----FRNYTVQfdvgvefeedlrsvdgrkcqtiVTWEeehLVCVqkgevpNRGWRH-WLEGEMLYLELTAR-----DAVCEQVFRKV-----------------------------------h

d1a3ya_ -------pfELSGKWITSYIGSSDLEKIGENAPFQVFMRSIEFDdKESKVYLNFFSKENGICEEFSLIGTKQEGNTYDVNYAGNNKFVVSYAS-ETALIISNINVdEEGDKTIMTGLLGKGTDIEDQDLEKFKEVTRENGIPEENIVNIIER-DDCPA--

d1gm6a_ vvtsnfdasKIAGEWYSILLASDAKENIEENGSMRVFVEHIRVL-DNSSLAFKFQRKVNGECTDFYAVCDKVGDGVYTVAYYGENKFRLLEVNySDYVILHLVDV-NGDKTFQLMEFYGRKPDVEPKLKDKFVEICQQYGIIKENIIDLTKIdRCFQLrg

d1a3ya_ ----------------pfELSGKWITSYIGSsDLEKigenaPFQV-FMRSIEFDdKESKVYLNFFSKEN-----GICEEFSLIGTKQEG-NTYDVNY--------AGNNKFVVSYAS-ETALIISNINV--deegdKTIMTGLLGKGTDIED-QDLEKFKEVTRENGIPEeNIVNIIERdDCPA

d1hbqa_ erdcrvssfrvkenfdkaRFAGTWYAMAKKD-PEGL-----FLQDnIVAEFSVD-ENGQMSATAKGRVRllnnwDVCADMVGTFTDTEDpAKFKMKYwgvasflqKGNDDHWIIDTDyETFAVQYSCRLlnldgtcADSYSFVFARDPSGFSpEVQKIVRQRQEELCLAR-QYRLIPHNgYCNG

d1a3ya_ --------------pfELSGKWITSYIGSSDlekigenapfqvFMRSIEFDdkESKVYLNFFSKENGICEEFSLIGTKQEG-NTYDVNY---AGNNKFVVSYAS-ETALIISNINVdeegdKTIMTGLLGKGTDIEDQDLEKFKEVTRENGIPEENIVNIIERdDCPA-----------

d1iw2a_ aspistiqpkanfdaqQFAGTWLLVAVGSAG--------raeaTTLHVAPQ--GTAMAVSTFRKLDGICWQVRQLYGDTGVlGRFLLQArgaRGAVHVVVAETDyQSFAVLYLERA-----GQLSVKLYARSLPVSDSVLSGFEQRVQEAHLTEDQIFYFPKY-GFCEaadqfhvldev

d1a3ya_ ----------------pfELSGKWITSYIGSsDLEKigenaPFQV-FMRSIEFDdKESKVYLNFFSKE-----NGICEEFSLIGTKQEGN-TYDVNY--------AGNNKFVVSYAS-ETALIISNINV--deegdKTIMTGLLGKGTDI-EDQDLEKFKEVTRENGIPEeNIVNIIeRDDCPa

d1jyda_ erdcrvssfrvkenfdkaRFSGTWYAMAKKD-PEGL-----FLQDnIVAEFSVD-ETGQMSATAKGRVrllnnWDVCADMVGTFTDTEDPaKFKMKYwgvasflqKGNDDHWIVDTDyDTYAVQYSCRLlnldgtcADSYSFVFSRDPNGlPPEAQKIVRQRQEELCLAR-QYRLIV-HNGYC-

d1a3ya_ pfELSGKWITSYIGS--SDLE------kiGENApfQVFMRSIEFDDkeSKVYLNFFSKengiCEEFSLIGT--------------------KQEG---NTYD-vnyagnNKFVVsYASETALIISNINVdeegdKTIMTGLLGKGtdiedqdlekfkevtrengipeenivniierddcpa

d1kqxa_ paDFNGTWEMLSNDNfeDVMKaldidfatRKIAvhLKQTKVIVQNG--DKFETKTLST----FRNYEVNFVigeefdeqtkgldnrtvktlVKWDgdkLVCVqkgekenRGWKQ-WIEGDLLHLEIHCQ-----DKVCHQVFKKK-----------------------------------n

d1a3ya_ pFELSGKWITSYIGS--SDLE------kiGENAPfQVFMrSIEFDDKESKVYLNFFskengiCEEFSLI-------------------gTKQEG---NTYD-vnyagnNKFVVSYASeTALIISNINVdeegdKTIMTGLLGKGtdiedqdlekfkevtrengipeenivniierddcpa

d1liba_ cDAFVGTWKLVSSENfdDYMKevgvgfatRKVAGmAKPN-MIISVNGDLVTIRSES------TFKNTEIsfklgvefdeitaddrkvksIITLDggaLVQVqkwdgksTTIKRKRDG-DKLVVECVMK-----GVTSTRVYERA------------------------------------

d1a3ya_ pfeLSGKWITSYIGS--SDLE------kiGENAPfQVFMRSIEFDDkeSKVYLNFFSKengiCEEFSLIGT--------------------KQEG---NTYD-vnyagnNKFVVsYASETALIISNINVdeegdKTIMTGLLGKGtdiedqdlekfkevtrengipeenivniierddcpa

d1lpja_ padLSGTWTLLSSDNfeGYMLalgidfatRKIAKlLKPQKVIEQNG--DSFTIHTNSS----LRNYFVKFKvgeefdednrgldnrkckslVIWDndrLTCIqkgekknRGWTH-WIEGDKLHLEMFCE-----GQVCKQTFQRA------------------------------------

d1a3ya_ ---------------------------pfELSGKWITSYIGSsdlekigenAPFQV---FMRSIEFDDkeSKVYLNFFS-KENGICEEFSLIGTKQ--EGNTYDVNY--AGNNKFVVSYAS-ETALIISNINVdeEGDKTIMTGLLGKGTDIEDQDLEKFKEVTRENGIPEENIVNIIErdDCPA------

d1obqa_ dkipdfvvpgkcasvdrnklwaeqtpnrnSYAGVWYQFALTN--------nPYQLIekcVRNEYSFDG--KQFVIESTGiAYDGNLLKRNGKLYPNpfGEPHLSIDYenSFAAPLVILETDySNYACLYSCIDynFGYHSDFSFIFSRSANLADQYVKKCEAAFKNINVDTTRFVKTVQgsSCPYdtqktl

d1a3ya_ -----------------------pfelsgKWITSYIG------------------------------SSDLekigenapfQVFMRSIEfddkeskvYLNFFSKEngICEEFSLI-GTKQEG---------NTYDVNY-------AGNNKFVVS-yasetaLIISNINVDE------egdkTIMTGLLGkgTDIEDQDLEKFkevtrengipeenivniierddcpa

d1oeja_ gkplteveqkaangvfddanvqnrtlsdwDGVWQSVYpllqsgkldpvfqkkadadktktfaeikdyYHKG-----yatdIEMIGIED--------GIVEFHRN--NETTSCKYdYDGYKIltyksgkkgVRYLFECkdpeskaPKYIQFSDHiiaprksSHFHIFMGNDsqqsllnemeNWPTYYPY--QLSSEEVVEEM----------------------msh

d1a3ya_ pfELSGKWITSYIGS--SDLE------kiGENAPFQvFMRSIEFDDkeSKVYLNFFSKengiCEEFSLI--------------------gtKQEG---NTYD-vnyagnNKFVVsYASETALIISNINVdeegdKTIMTGLLGKGtdiedqdlekfkevtrengipeenivniierddcpa

d1opaa_ tkDQNGTWEMESNENfeGYMKaldidfatRKIAVRLtQTKIIVQDG--DNFKTKTNST----FRNYDLDftvgvefdehtkgldgrnvktlVTWEgntLVCVqkgekenRGWKQ-WVEGDKLYLELTCG-----DQVCRQVFKKK------------------------------------

d1a3ya_ pfELSGKWITSYIGS--SDLE------kiGENApfQVFMRSIEFDDkeSKVYLNFFSKengiCEEFSLIGT------------------KQEG---NTYDvnyagNNKFVVsYASETALIISNINVdeegdKTIMTGLLGKGtdiedqdlekfkevtrengipeenivniierddcpa

d1p6pa_ --AFNGTWNVYAQENyeNFLRtvglpediIKVAkdVNPVIEIEQNG--NEFVVTSKTP----KQTHSNSFTvgkeseitsmdgkkikvtVQLEggkLICK---sdKFSHIQ-EVNGDEMVEKITIG-----SSTLTRKSKRV------------------------------------

d1a3ya_ --------------------------pfELSGKWITSYIGSsdlekigenAPFQ---vFMRSIEFDdKESKVYLNFFSKE--NGICEEFSLIGTKQE---GNTYDVNYA--GNNKFVVSYAS--ETALIISNInvdeegdkTIMTGLLGKGTDIEDQDLEKFKEVTRENGIPEENIVNIIErddcpa

d1qwda_ hlestslykkssstpprgvtvvnnfdakRYLGTWYEIARFD--------hRFERglekVTATYSLR-DDGGLNVINKGYNpdRGMWQQSEGKAYFTGaptRAALKVSFFgpFYGGYNVIALDreYRHALVCGP-------dRDYLWILSRTPTISDEVKQEMLAVATREGFDVSKFIWVQQ----pg

d1a3ya_ -----pfeLSGKWITsYIGSSD------LEKIgenapfQVFMRSIEFDDkeSKVYLNFFSKENGICEEFSLIGTkqegNTYDV------------------------------nyAGNN-KFVVsYASETALIISNINVDEEGDKTIMTGLLGKGTdiedqdlekfkevtrengipeenivniierddcpa

d1r0ua_ gfqsnamkQETPITL-HVKSVIeddgnqEVIE------FRTTGFYYVKQ--NKVYLSYYEEHDLGKVKTIVKVS----EGEVLvmrsgavkmnqrfvtgastiakykmsfgelelKTSTkSIQS-DLDEEKGRISIAYDMHVGHLHNMTITYEGGT-----------------------------------

d1a3ya_ pfELSGKWITSYIGS--SDLE------kiGENAPFQVFmRSIEFDDKESKVYLNFFskengiCEEFSLIGT-------------------KQEG---NTYDvnyagNNKFVVsYASETALIISNINVdeegdKTIMTGLLGKGtdiedqdlekfkevtrengipeenivniierddcpa

d1tvqa_ --AFSGTWQVYAQENyeEFLKalalpedlIKMARDIKP-IVEIQQKGDDFVVTSKT------PRQTVTNSFtlgkeadittmdgkklkctVHLAngkLVTK---seKFSHEQ-EVKGNEMVETITFG-----GVTLIRRSKRV------------------------------------

d1a3ya_ ---------------------------------------------------------------------------------------------------------------------------------------------------------pfelsgKWITSYIGSsdleKIGE-------------------------napFQVFMRSIEFDdkesKVYLNFFSKEngiCEEFSLI-------------------GTKQEGNTYDVNY-------aGNNKFVVSYASEtALIISNINVDeEGDKTIMTGLLGK---------gtdiedqdlekfkevtrengipeenivniierddcpa

d1vpra1 ekgfeagdnklggalnakhvekygdnfkngxhkpefhedglhkpxevggkkfesgfhyllechelggknasggyggplcedpygsevqaxtekllkeadsdrtlcfnnfqdpcpqltkeqvaxckgfdygdktlklpcgplpwpaglpepgyvpktnplHGRWITVSG----GQAAfikeaiksgmlgaaeankivadtdhhqtGGXYLRINQFG----DVCTVDASVA-kfARAKRTWksghyfyeplvsggnllgvWVLPEEYRKIGFFwexesgrcFRIERRAFPVGP-YTFXRQATEV-GGKISFVFYVKVSndpesdpiplqsrdytalagrdnaptnlgkpyptlakdldypkkrd

d1a3ya_ pfeLSGKWITsYIGSSDLE--------------kiGENApfQVFMrSIEFDDkeSKVYLNFFSKengiCEEFSLI--------------------gTKQEGNTYDVN--------yaGNNKFVVSYAsetaLIISNINVDEeGDKTIMTGLLGkgtdiedqdlekfkevtrengipeenivniierddcpa

d1xcaa_ --pNFSGNWK-IIRSENFEellkvlgvnvmlrkiaVAAA-sKPAV-EIKQEG--DTFYIKTSTT----VRTTEINfkvgeefeeqtvdgrpckslvKWESENKMVCEqkllkgegpkTSWTMELTND----GELILTMTAD-DVVCTRVYVRE--------------------------------------

d1a3ya_ pfELSGKWITSYIGSsdlekigenAPFQVFMRSIEFDdKESKVYLNFFSkeNGICEEFSLIGTKQEG-NTYDVNyagNNKFVVSYA-SETALIISNINVdeeGDKTIMTGLLGKGTDIEDQDLEKFKEVTRENGIPEENIVNiIERDdcpa

d1xkia_ --DVSGTWYLKAMTV---------NLESVTPMTLTTL-EGGNLEAKVTM--SGRCQEVKAVLEKTDEpGKYTAD-ggKHVAYIIRShVKDHYIFYSEGE---GKPVRGVKLVGRDPKNNLEALEDFEKAAGARGLSTESILI-PRQS----

d1a3ya_ -----------pfELSGKWITSYIGSSDLEKIGE-NAPFQVFMRSIEFDdKESKVYLNFFSKENGICEEFSLIGTKQEG-NTYDVNYAGNNKFVVSYAS-ETALIISNINVdeeGDKT--IMTGLLGKGTDIEDQDLEKFKEVTRENGIPeeNIVNI----IERDDCPa

d1yupa1 iivtqtmkdldvqKVAGTWYSLAMAASDISLLDAqSAPLRVYVEELKPT-PGGDLEILLQKWENGKCAQKKIIAEKTEIpAVFKIDALNENKVLVLDTDyKKYLLFCMENS---AEPEqsLACQCLVRTPEVDDEAMEKFDKALKALPMH--IRLSFnptqLEEQCRV-

d1a3ya_ ---------pFELSGKWITsYIGSSD--LEKIgenapfQVFMRsIEFDD---KESKVYLNFFSKEngICEE-FSLIGTK---------------------QEGN--------------tydvnyaGNNKFVVsYASETALIISNINVDEEGD-KTIMTGLLGKGtdiedqdlekfkevtrengipeenivniierddcpa

d2a13a1 ppvhpfvaplSYLLGTWRG-QGEGEYptIPSF------RYGEE-IRFSHsgkPVIAYTQKTWKLE--SGAPxHAESGYFrprpdgsievviaqstglvevQKGTynvdeqsiklksdlvgnaskvKEISREF-ELVDGKLSYVVRXSTTTNPlQPHLKAILDKL------------------------------------

d1a3ya_ pfELSGKWITSYIGS--SDLE------kiGENApfQVFMRSIEFDDkeSKVYLNFFSKengiCEEFSLI------------------gtKQEG----NTYDvnyaGNNKFVVsYASETALIISNINVdeegdKTIMTGLLGKGtdiedqdlekfkevtrengipeenivniierddcpa

d2f73a1 -mSFSGKYQLQSQENfeAFMKaiglpeelIQKGkdIKGVSEIVQNG--KHFKFTITAG----SKVIQNEftvgeeceletmtgekvktvVQLEgdnkLVTT---fKNIKSVT-ELNGDIITNTMTLG-----DIVFKRISKRI------------------------------------

d1a3ya_ --------pFELSGKWITsYIGSSD--LEKIgenapfQVFMRSIEFD-dKESKVYLNFFSKeNGICE-EFSLIGT---------------------kQEGN---------TYDV------------nyAGNNKFVVsYASETALIISNINVDEEGD-KTIMTGLLGKGtdiedqdlekfkevtrengipeenivniierddcpa

d2fr2a1 dlapalqalSPLLGSWAG-RGAGKYptIRPF------EYLEEVVFAHvgKPFLTYTQQTRAvADGKPlHSETGYLrvcrpgcvelvlahpsgiteieVGTYsvtgdvielELSTradgsiglaptakeVTALDRSY-RIDGDELSYSLQMRAVGQPlQDHLAAVLHRQ-----------------------------------r

d1a3ya_ -------pfELSGKWITsYIGSsdlekigenapFQVFMrSIEFDdkeskVYLNFFSK-engiCEEFS-LIGTKQEG----NTYDVNY--AGNNKFVVSYAsetaLIISNINVDEE-------------------------------GDKTIMTG-LLGKgtdiedqdlekfkevtrengipeenivniiERDD--------------cpa

d2gc9a1 xtktfktldDFLGTHFIyTYDN----------gWEYEW-YAKND-----HTVDYRIHggxvaGRWVTdQKADIVXLtegiYKISWTEptGTDVALDFXPN---eKKLHGTIFFPKwveehpeitvtyqnehidlxeqsrekyatypKLVVPEFAnITYX-------------gdagqnnedviseapykEXPNdirngkyfdqnyhrlnk

d1a3ya_ ------pFELSGKWITsYIGSSD---LEKIgenapfQVFMrSIEFDDK-eskvYLNFFSkengicEEFSLI-----------------gTKQEG-----NTYD----vnyaGNNKFVVSYASETALIISNINVDE-EGDKTIMTGLLGKGtdiedqdlekfkevtrengipeenivniierddcpa

d2o62a1 erpllqiNDLLGEWRG-QAVTIYrdlRPPD-----iYSTT-LKIQLDDagrlxQSTSFG------ERTITStatikgsivlfdqdpekqVQVLLlpdgaSATSplkvqlrqPLFLEAGWLIQSDLRQRXIRSYNDkGEWVSLTLVTEERV------------------------------------

d1a3ya_ -------------pFELS--GKWITSYIGSSDLEKIGENapfQVFMrSIEFDDKESKVYLNFFSKENGICEEFSLI-GTKQEGNTYDVNY---------------aGNNKFVVSYASETALIISNINVDEEGDKTIMTGLLGK-GTDI-EDQDLEKFKEVTRengiPEENIVNIIERDDCPA----------

d2ofmx1 actknaiaqtgfnkDKYFngDVWYVTDYLDLEPDDVPKR---YCAA-LAAGTASGKLKEALYHYDPKTQDTFYDVSeLQVESLGKYTANFkkvdkngnvkvavtagNYYTFTVMYADDSSALIHTCLHKGNKDLGDLYAVLNRnKDAAaGDKVKSAVSAATL----EFSKFISTKENNCAYDndslkslltk

d1a3ya_ -----------pfELSGKWITSYIGSSDLEKIGENAPFQVFMRSIEFDDkeSKVYLNFFSKENGICEEFSLIGTKQ-EGNTYDVNYAGNNKFVVSYAS-ETALIISNINVDeEGDKTIMTGLLGKGTDIEDQDLEKFKEVTRENGIPEENIVNIIERDDCpa

d2ozqa1 eeasstgrnfnveKINGEWHTIILASDKREKIEDNGNFRLFLEQIHVLE--KSLVLKFHTVRDEECSELSMVADKTeKAGEYSVTYDGFNTFTIPKTDyDNFLMAHLINEK-DGETFQLMGLYGREPDLSSDIKERFAQLCEEHGILRENIIDLSNANRC--

d1a3ya_ -------------pFELS--GKWITSYIGSSDLEkiGENApfQVFMrSIEFDDKESKVYLNFFSKENGICEEFSLI-GTKQEGNTYDVNY---------------aGNNKFVVSYASETALIISNINVDEEGDKTIMTGLLGK-GTDI-EDQDLEKFKEVTRengiPEENIVNIIERdDCPA----------

d3np1a_ kctknalaqtgfnkDKYFngDVWYVTDYLDLEPD-dVPKR--YCAA-LAAGTASGKLKEALYHYDPKTQDTFYDVSeLQEESPGKYTANFkkvekngnvkvdvtsgNYYTFTVMYADDSSALIHTCLHKGNKDLGDLYAVLNRnKDTNaGDKVKGAVTAASL----KFSDFISTKDNkCEYDnvslkslltk

d1avgi_ aegddcsiekamgDFKP----------eeffngTWYLA-HGPGV------------------------------------tspavCQKFttsgskgftqiveigynkfeSNVKFQCNQVdNKNG---------EQYSFKCKSSDNT-eFEADFTFIsvsydnfALVCRSITFT-----sqpkeDRYLVFERTKSDT-dpdakeic

d1oeja_ ---gkplteveqkAANGvfddanvqnrtlsdwdGVWQSvYPLLQsgkldpvfqkkadadktktfaeikdyyhkgyatdiemigieDGIV--------------efhrnnETTSCKYDYD-GYKIltyksgkkgVRYLFECKDPESKapKYIQFSDHiiaprksSHFHIFMGNDsqqsllnemeNWPTYYPYQLSSEevveemmsh

d1avgi_ ---------------------AEGDdcsiekamgdfkpeeffngtwylaHGPG---------------vTSPAvcqkfttsgskgFTQI--veigynkfesnVKFQCnqvdnkngeQYSFKCKSSD---NTEFEADFTFISVSY---DNFALVCRSITFTsqpkEDRYLVFERTksdtdpdakeic

d1r0ua_ gfqsnamkqetpitlhvksviEDDG--------------------nqevIEFRttgfyyvkqnkvylsyYEEH------dlgkvkTIVKvsegevlvmrsgaVKMNQ-----rfvtGASTIAKYKMsfgELELKTSTKSIQSDLdeeKGRISIAYDMHVG----HLHNMTITYE---------ggt

d1avgi_ ----------------------------------------------------------------------------------------------AEGD------------------------------------------------dcsiekamGDFKP---------------------------------------------------eefFNGTwylahgpgvTSPA--vcqkfTTSGskgftqiveigynkfESNVKFQCNQVdnKNGE-QYSFKCKSSD-NTEFEADFTFISvsydnFALVCRSITFtSQPKEDRYLVFER---------------------------------tksdtdpdakeic

d1vpra1 ekgfeagdnklggalnakhvekygdnfkngxhkpefhedglhkpxevggkkfesgfhyllechelggknasggyggplcedpygsevqaxteklLKEAdsdrtlcfnnfqdpcpqltkeqvaxckgfdygdktlklpcgplpwpaglpepgyvpKTNPLhgrwitvsggqaafikeaiksgmlgaaeankivadtdhhqtggxylrinqfgdvCTVD-------asVAKFarakrtwKSGH---------yfyeplVSGGNLLGVWV--LPEEyRKIGFFWEXEsGRCFRIERRAFP--vgpYTFXRQATEV-GGKISFVFYVKVSndpesdpiplqsrdytalagrdnaptnlgkpyptlakdldypkkrd

d1avgi_ aegddcsiekamgdfKPEEFF-------------------------------NGTWYLAHG----PGVTsPAVCqkfTTSGSkgftqiveigynkFESNVKFQCNQVDnknGEQYSFKC-KSSDN---TEFEADFTFISVsydnfaLVCRSITFT-----SQPKEDRYLVfertksdtdpdakeic

d2a13a1 -------ppvhpfvaPLSYLLgtwrgqgegeyptipsfrygeeirfshsgkpVIAYTQKTWklesGAPX-HAES-gyFRPRP----dgsievviaQSTGLVEVQKGTYnvdEQSIKLKSdLVGNAskvKEISREFELVDG------KLSYVVRXStttnpLQPHLKAILD--------------kl

d1avgi_ aegddcsiekamgdfKPEEF-------------------------------fNGTWYLAHG----PGVTsPAVCQKFTTSGSkgftqiveigynkFESNVKFQCNQVD--nkngEQYSFKC----KSSD------nTEFEADFTFISVsydnfaLVCRSITFT-----SQPKEDRYLVfertksdtdpdakeic

d2fr2a1 --------dlapalqALSPLlgswagrgagkyptirpfeyleevvfahvgkpFLTYTQQTRavadGKPL-HSETGYLRVCRP-----gcvelvlaHPSGITEIEVGTYsvtgdvIELELSTradgSIGLaptakevTALDRSYRIDGD------ELSYSLQMRavgqpLQDHLAAVLH-------------rqr

d1avgi_ aegddcsiekamgDFKPEEFFNGTWYLAHgpgvtspavcqkFTTSgskgftqiveIGYNKFESnVKFQCNQV---dnkngeQYSFKCKssdnTEFEADFTFISVSydnfALVCRSITFTS-------------------------------QPKEDRYLVFER---------------tksDTDP-DAKE----------ic

d2gc9a1 ---------xtktFKTLDDFLGTHFIYTY--dngweyewyaKNDH----tvdyriHGGXVAGR-WVTDQKADivxltegiyKISWTEP----TGTDVALDFXPNE----KKLHGTIFFPKwveehpeitvtyqnehidlxeqsrekyatypKLVVPEFANITYxgdagqnnedviseapykEXPNdIRNGkyfdqnyhrlnk

d1avgi_ aegddcsiekamGDFKPEEFFN-----------------gtwyLAHG------------pgvtSPAVCqkfttSGSKgftqiveigynkfesnVKFQCNQVdnkngeQYSFKC--kssDNTEFEADFTFISVsydnfALVCRSITFT-----SQPKEDRYLVFErtksdtdpdakeic

d2o62a1 -----------eRPLLQINDLLgewrgqavtiyrdlrppdiysTTLKiqlddagrlxqstsfgERTIT-statIKGS---------ivlfdqdPEKQVQVL-llpdgASATSPlkvqlRQPLFLEAGWLIQS-----DLRQRXIRSYndkgeWVSLTLVTEERV--------------

d1beba_ ---------QTMKGLDIQKV-AGTWYSLA----maasdislldaqsaplrvYVEElkptpegdleillqKWENgecAQKKIIAE--KTKIpaVFKIDA------LNENKVLVLDTDYKKYLLFCMENS-aepEQSLVCQCLVRTPEvddealekfdkalkalpmhirlsfnpTQLEEQ---c

d1avgi_ aegddcsieKAMGDFKPEEFfNGTWYLAHgpgvtspavcqkfttsgskgftQIVE---------igynkFESN---VKFQCNQVdnKNGE--QYSFKCkssdntEFEADFTFISVSYDNFALVCRSITftsqPKEDRYLVFERTKS--------------------------DTDPDAkeic

d1beba_ qtmkgLDIQKVAGTWYSLAMAASDISLLDaQSAPLRVYVEELKPTPE-GDLEILLQKWENGECAQKKIIAEKTkIPAVFKIDALNENKVLVLDTDyKKYLLFCMENSAEpEQSLVCQCLVRTPEVDDEALEKFDKALKALPMH--IRLSFnptQLEEQC-

d1bj7a_ -----IDPSKIPGEWRIIYAAADNKDKIV-EGGPLRNYYRRIECINDcESLSITFYLKDQGTCLLLTEVAKRQ-EGYVYVLEFYGTNTLEVIHVS-ENMLVTYVENYDGeRITKMTEGLAKGTSFTPEELEKYQQLNSERGVPneNIENL--iKTDNCPp

d1beba_ ---------QTMKGLDIQKVAGTWYSLAMAASDislldaqsAPLR--vYVEELKPTPEGDLEILLQKWE-----NGECAQKKIIAEKTKIPAVFKIDA--------LNENKVLVLDTDYKKYLLFCMENSA----EPEQsLVCQCLVRTPEVDD-EALEKFDKALKALPM-HIRLSFnptQLEEQC

d1brqa_ erdcrvssfRVKENFDKARFSGTWYAMAKKDPE--------GLFLqdnIVAEFSVDETGQMSATAKGRVrllnnWDVCADMVGTFTDTEDPAKFKMKYwgvasflqKGNDDHWIVDTDYDTYAVQYSCRLLnldgTCAD-SYSFVFSRDPNGLPpEAQKIVRQRQEELCLaRQYRLI---VHNGYC

d1beba_ qtmkgldiqKVAGTWYSLAMAA--SDIS------llDAQSAPLRV-YVEELKPTPeGDLEILLQkwengECAQKKIIAEK-----------------TKIP----AVFK----idalneNKVLVLDTDYkkYLLFCMEnsaePEQSLVCQCLVRTpevddealekfdkalkalpmhirlsfnptqleeqc

d1cbia_ --------pNFAGTWKMRSSENfdELLKalgvnamlRKVAVAAASkPHVEIRQDG-DQFYIKTS----tTVRTTEINFKVgegfeeetvdgrkcrslPTWEnenkIHCTqtllegdgpkTYWTRELAND--ELILTFG----ADDVVCTRIYVRE-----------------------------------

d1beba_ qtmkgldIQKVAGTWYSLAMAASDISLLDaQSAPLRVYVEELKPTPE-GDLEILLQKWENGECAQKKIIAEKTKIPaVFKIDALNENKVLVLDTDyKKYLLFCMENS-aepEQSLVCQCLVRtPEVDD-EALEKFDKALKALPMH--IRLSFnptQLEEQ-c

d1e5pa_ -------FAELQGKWYTIVIAADNLEKIE-EGGPLRFYFRHIDCYKNcSEXEITFYVITNNQCSKTTVIGYLKGNG-TYETQFEGNNIFQPLYIT-SDKIFFTNKNXdragQETNXIVVAGK-GNALTpEENEILVQFAHEKKIPveNILNI--lATDTCpe

d1beba_ qtMKGLDIQKVAGTWYSLAMAASdislldaqsaplrvYVEELKPTPEgDLEILLQKWENGECAQKKIIAEKTKIPAVFKIDA-lNENKVLVLDTDYKKYLLFCMENSAepeQSLVCQCLVRTPEVDDEALEKFDKALKALPMH--IRLSFNPTqlEEQC-------

d1epaa_ --VKDFDISKFLGFWYEIAFASK----------eekmGAMVVELKEN-LLALTTTYYSEDHCVLEKVTATEGDGPAKFQVTRlsGKKEVVVEATDYLTYAIIDITSLVagaVHRTMKLYSRSLDDNGEALYNFRKITSDHGFSetDLYILKHD--LTCVkvlqsaa

d1beba_ -------QTMKGLDIQKV-AGTWYSLAMAASDISllDAQSaplRVYVEELKPTpEGDLEILLQKW--eNGECAQKKIIAEKTKIPAVFKIDA---------------lNENKVLVLDTDyKKYLLFCMENS---AEPEqsLVCQCLVRTP-EVDDE-ALEKFDKALKalpmhIRLSFNptqlEEQC------

d1euoa_ mdcstniSPKQGLDKAKYfSGKWYVTHFLDKDPQ--VTDQ---YCSSFTPRES-DGTVKEALYHYnanKKTSFYNIGEGKLESSGLQYTAKYktvdkkkavlkeadekNSYTLTVLEAD-DSSALVHICLRegsKDLG--DLYTVLTHQKdAEPSAkVKSAVTQAGL--qlsQFVGTK-dlgCQYDdqftsl

d1beba_ qtMKGLDIQKVAGTWYSLAMAASDISLLDaQSAPLRVYVEELKPTPEGDLEILLQKWENGECAQKKIIAEKTKIPAVFKIDALNENKVLVLDTDYKKYLLFCMENSAEpEQSLVCQCLVRTPEVDDEALEKFDKALKALPMH--IRLSFNptQLEEQC----

d1ew3a_ vaIRNFDISKISGEWYSIFLASDVKEKIE-ENGSMRVFVDVIRALDNSSLYAEYQTKVNGECTEFPMVFDKTEEDGVYSLNYDGYNVFRISEFENDEHIILYLVNFDKdRPFQLFEFYAREPDVSPEIKEEFVKIVQKRGIVkeNIIDLT--KIDRCFqlrg

d1beba_ ----QTMKGLDIQKVAGTWYSLAMAAS-DISLldaQSAPLR-VYVEELKPTPEGDLEILLQKWENGECAQKKIIAEKTKIPAVFKIDALNENKVLVLDTDyKKYLLFCMENSAEPEQSLVCQCLVRTPEVDDEALEKFDKALKAL-PMHIRLSFNPTqlEEQC-

d1exsa_ vevtPIMTELDTQKVAGTWHTVAMAVSdVSLL--dAKSSPLkAYVEGLKPTPEGDLEILLQKRENDKCAQEVLLAKKTDIPAVFKINALDENQLFLLDTDyDSHLLLCMENSASPEHSLVCQSLARTLEVDDQIREKFEDALKTLsVPMRILPAQLE--EQCRv

d1beba_ qtmkgldIQKVAG-TWYSLAMAA--SDIS------llDAQSAplRVYVEELKPTPEGDLEILLQKWengeCAQKKII------------------aEKTKI---pAVFKidalNENKVLVlDTDYkKYLLFCMEnSAEPeqsLVCQCLVRTpevddealekfdkalkalpmhirlsfnptqleeqc

d1ftpa_ -------VKEFAGiKYKLDSQTNfeEYMKaigvgaieRKAGL-aLSPVIELEILDGDKFKLTSKTA----IKNTEFTfklgeefdeetldgrkvksTITQDgpnkLVHEqkgdHPTIIIR-EFSK-EQCVITIK-LGDL---VATRIYKAQ-----------------------------------

d1beba_ qtmkgldiQKVAGTWYSLAMAASDI--------sllDAQSAPlRVYVEELKPTPEGdLEILLQKWengeCAQKKII--------------------aeKTKI--PAVFK-idalneNKVLVlDTDYkKYLLFCMEnSAEPeqsLVCQCLVRTpevddealekfdkalkalpmhirlsfnptqleeqc

d1ggla_ -------pPNLTGYYRFVSQKNMEDylqalnislavRKIALL-LKPDKEIEHQGNH-MTVRTLST----FRNYTVQfdvgvefeedlrsvdgrkcqtiVTWEeeHLVCVqkgevpnRGWRH-WLEG-EMLYLELT-ARDA---VCEQVFRKV----------------------------------h

d1beba_ qtMKGLDIQKVAGTWYSLAMAASDISLLDaQSAPLRVYVEELKPTPEGDLEILLQKWENGECAQKKIIAEKTkIPAVFKIDALNENKVLVLDTDYKKYLLFCMENSAepeQSLVCQCLVRTPEVDDEALEKFDKALKALPMH--IRLSFNPtqlEEQC-----

d1gm6a_ vvTSNFDASKIAGEWYSILLASDAKENIE-ENGSMRVFVEHIRVLDNSSLAFKFQRKVNGECTDFYAVCDKV-GDGVYTVAYYGENKFRLLEVNYSDYVILHLVDVNgdkTFQLMEFYGRKPDVEPKLKDKFVEICQQYGIIkeNIIDLTK---IDRCfqlrg

d1beba_ ---------QTMKGLDIQKVAGTWYSLAMAASDislldaqsAPLR--vYVEELKPTPEGDLEILLQKWEN-----GECAQKKIIAEKTKIPAVFKIDA--------LNENKVLVLDTDYKKYLLFCMENSA----EPEQsLVCQCLVRTPEVDD-EALEKFDKALKALPM-HIRLSFnptQLEEQC--

d1hbqa_ erdcrvssfRVKENFDKARFAGTWYAMAKKDPE--------GLFLqdnIVAEFSVDENGQMSATAKGRVRllnnwDVCADMVGTFTDTEDPAKFKMKYwgvasflqKGNDDHWIIDTDYETFAVQYSCRLLnldgTCAD-SYSFVFARDPSGFSpEVQKIVRQRQEELCLaRQYRLI---PHNGYCng

d1beba_ -------QTMKGLDIQKVAGTWYSLAMAASDislldaqsaplrvYVEELKPTpEGDLEILLQKWENGECAQKKIIAEKTKIPAVFKIDA---lNENKVLVLDTDYKKYLLFCMENSAepeqSLVCQCLVRTPEVDDEALEKFDKALKALPMH--IRLSFnptQLEEQC------------

d1iw2a_ aspistiQPKANFDAQQFAGTWLLVAVGSAG---------raeaTTLHVAPQ-GTAMAVSTFRKLDGICWQVRQLYGDTGVLGRFLLQArgarGAVHVVVAETDYQSFAVLYLERAG----QLSVKLYARSLPVSDSVLSGFEQRVQEAHLTedQIFYF---PKYGFCeaadqfhvldev

d1beba_ ---------QTMKGLDIQKVAGTWYSLAMAASDislldaqsAPLR--vYVEELKPTPEGDLEILLQKWE-----NGECAQKKIIAEKTKIPAVFKIDA--------LNENKVLVLDTDYKKYLLFCMENSA----EPEQsLVCQCLVRTPEVDD-EALEKFDKALKALPM-HIRLSFnptQLEEQC

d1jyda_ erdcrvssfRVKENFDKARFSGTWYAMAKKDPE--------GLFLqdnIVAEFSVDETGQMSATAKGRVrllnnWDVCADMVGTFTDTEDPAKFKMKYwgvasflqKGNDDHWIVDTDYDTYAVQYSCRLLnldgTCAD-SYSFVFSRDPNGLPpEAQKIVRQRQEELCLaRQYRLI---VHNGYC

d1beba_ qtmkgldiQKVAGTWYSLAMA-------asdislldaqsaplrvYVEELKPTPeGDLEILLQKWengeCAQKKIIA--------------------EKTKI--PAVFKI-dalneNKVLVlDTDYkKYLLFCMEnSAEPeqsLVCQCLVRTPevddealekfdkalkalpmhirlsfnptqleeqc

d1kqxa_ -------pADFNGTWEMLSNDnfedvmkaldidfatrkiavhlkQTKVIVQNG-DKFETKTLST----FRNYEVNFvigeefdeqtkgldnrtvktLVKWDgdKLVCVQkgekenRGWKQ-WIEG-DLLHLEIH-CQDK---VCHQVFKKKN----------------------------------

d1beba_ qtmkgldIQKVAGTWYSLAMAA--SDIS------llDAQSAplRVYVEELKPTPEgdLEILLQKWengeCAQKKII------------------aEKTKI--PAVFKID-alneNKVLVLDTDykKYLLFCMEnSAEPeqsLVCQCLVRTpevddealekfdkalkalpmhirlsfnptqleeqc

d1liba_ -------CDAFVGTWKLVSSENfdDYMKevgvgfatRKVAG-mAKPNMIISVNGD-lVTIRSEST----FKNTEISfklgvefdeitaddrkvksIITLDggALVQVQKwdgksTTIKRKRDG--DKLVVECV-MKGV---TSTRVYERA-----------------------------------

d1beba_ qtmkgldiQKVAGTWYSLAMAA--SDIS------llDAQSAplrvYVEELKPTPeGDLEILLQKWengeCAQKKIIAEK------------------TKIP----AVFK-idalneNKVLVlDTDYkKYLLFCMENsaepEQSLVCQCLVRTpevddealekfdkalkalpmhirlsfnptqleeqc

d1lpja_ -------pADLSGTWTLLSSDNfeGYMLalgidfatRKIAK-llkPQKVIEQNG-DSFTIHTNSS----LRNYFVKFKVgeefdednrgldnrkcksLVIWdndrLTCIqkgekknRGWTH-WIEG-DKLHLEMFC----EGQVCKQTFQRA-----------------------------------

d1beba_ ------------------qTMKGL--DIQKVAGTWYSLAMAAsdislldaqsAPLRV---YVEELKPTPEgDLEILLQKWE-NGECAQKKIIAEKTK-ipaVFKIDA--LNENKVLVLDTDYKKYLLFCMENSA-epeQSLVCQCLVRTPEVDDEALEKFDKALK--ALPMHIRLSFnptQLEE--------qc

d1obqa_ dkipdfvvpgkcasvdrnkLWAEQtpNRNSYAGVWYQFALTN---------nPYQLIekcVRNEYSFDGK-QFVIESTGIAyDGNLLKRNGKLYPNPfgepHLSIDYenSFAAPLVILETDYSNYACLYSCIDYnfgyHSDFSFIFSRSANLADQYVKKCEAAFKniNVDTTRFVKT---VQGSscpydtqktl

d1beba_ ------------------qtmKGLDIQKVAGTWYSLA---------------------maasdislldaqsaplRVYVEELKPtpegdLEILLQKWengeCAQKKIIAEKTKI------------pAVFKIDA---lnENKVLVLD----tdYKKYLLFCMENSA--EPEQ-slVCQCLVR-TPEV--DDEALEkfdkalkalpmhirlsfnptqleeqc

d1oeja_ gkplteveqkaangvfddanvQNRTLSDWDGVWQSVYpllqsgkldpvfqkkadadktktfaeikdyyhkgyatDIEMIGIED-----GIVEFHRN----NETTSCKYDYDGYkiltyksgkkgvrYLFECKDpeskaPKYIQFSDhiiaprKSSHFHIFMGNDSqqSLLNemeNWPTYYPyQLSSeeVVEEMM------------------------sh

d1beba_ qtmkgldiQKVAGTWYSLAMAA--SDIS------llDAQSAplrvYVEELKPTPeGDLEILLQKWengeCAQKKIIAEK------------------TKIP----AVFK-idalneNKVLVlDTDYkKYLLFCMENsaepEQSLVCQCLVRTpevddealekfdkalkalpmhirlsfnptqleeqc

d1opaa_ -------tKDQNGTWEMESNENfeGYMKaldidfatRKIAV-rltQTKIIVQDG-DNFKTKTNST----FRNYDLDFTVgvefdehtkgldgrnvktLVTWegntLVCVqkgekenRGWKQ-WVEG-DKLYLELTC----GDQVCRQVFKKK-----------------------------------

d1beba_ qtmkgldiqKVAGTWYSLAMAA--SDIS------llDAQSAplRVYVEELKPTPeGDLEILLQKWengeCAQKKIIAEK----------------TKIP----AVFKidalneNKVLVlDTDYkKYLLFCMENSaepeQSLVCQCLVRTpevddealekfdkalkalpmhirlsfnptqleeqc

d1p6pa_ ---------AFNGTWNVYAQENyeNFLRtvglpediIKVAK-dVNPVIEIEQNG-NEFVVTSKTP----KQTHSNSFTVgkeseitsmdgkkikvTVQLeggkLICK---sdkFSHIQ-EVNG-DEMVEKITIG----SSTLTRKSKRV-----------------------------------

d1beba_ -------------------QTMKGLDIQKVAGTWYSLAMAAsdislldaqsAPLR---vYVEELKPTPEGDLEILLQKWE--NGECAQKKIIAEKTKI--PAVFKIDAL--NENKVLVLDTD-YKKYLLFCMEnsaepeqsLVCQCLVRTPEVDDEALEKFDKALK--ALPMHIRLSFnptQLEEqc

d1qwda_ hlestslykkssstpprgvTVVNNFDAKRYLGTWYEIARFD---------hRFERglekVTATYSLRDDGGLNVINKGYNpdRGMWQQSEGKAYFTGAptRAALKVSFFgpFYGGYNVIALDrEYRHALVCGP------drDYLWILSRTPTISDEVKQEMLAVATreGFDVSKFIWV---QQPG--

d1beba_ qtmkgldiqkvAGTWYSlAMAASD------ISLLdaqsaplRVYVEELKPTPeGDLEILLQKWENGECAQKKIIAEKtkipavFKIDA--------lNENK------------------------VLVLdtdYKKYLLFCMENSA-epEQSLVCQCLVRTPevddealekfdkalkalpmhirlsfnptqleeqc

d1r0ua_ --gfqsnamkqETPITL-HVKSVIeddgnqEVIE-------FRTTGFYYVKQ-NKVYLSYYEEHDLGKVKTIVKVSE------GEVLVmrsgavkmnQRFVtgastiakykmsfgelelktstksIQSD--lDEEKGRISIAYDMhvgHLHNMTITYEGGT----------------------------------

d1beba_ qtmkgldiqKVAGTWYSLAMAA--SDIS------llDAQSAplRVYVEELKPTPeGDLEILLQKWengeCAQKKIIAEK----------------TKIP----AVFKidalneNKVLVlDTDYkKYLLFCMENsaepEQSLVCQCLVRTpevddealekfdkalkalpmhirlsfnptqleeqc

d1tvqa_ ---------AFSGTWQVYAQENyeEFLKalalpedlIKMAR-dIKPIVEIQQKG-DDFVVTSKTP----RQTVTNSFTLgkeadittmdgkklkcTVHLangkLVTK---sekFSHEQ-EVKG-NEMVETITF----GGVTLIRRSKRV-----------------------------------

d1beba_ ----------------------------------------------------------------------------------------------------------------------------------------------------qtmkgldiQKVAGTWYSLAM-------------------aasDISLLdaqsAPLR--VYVEELKPTPeGDLEILLQKWengecAQKKII------------------aEKTKIP---AVFKID---alNENKVLVlDTDY---KKYLLFCMEnSAEPeqsLVCQCLVRT------------pevddealekfdkalkalpmhirlsfnptqleeqc

d1vpra1 ekgfeagdnklggalnakhvekygdnfkngxhkpefhedglhkpxevggkkfesgfhyllechelggknasggyggplcedpygsevqaxtekllkeadsdrtlcfnnfqdpcpqltkeqvaxckgfdygdktlklpcgplpwpaglpepgyvpktNPLHGRWITVSGgqaafikeaiksgmlgaaeankIVADT----DHHQtgGXYLRINQFG-DVCTVDASVA---kfARAKRTwksghyfyeplvsggnllgVWVLPEeyrKIGFFWexesgRCFRIER-RAFPvgpYTFXRQATE-VGGK---ISFVFYVKVsndpesdpiplqsrdytalagrdnaptnlgkpyptlakdldypkkrd

d1beba_ qtmkgldiQKVAGTWYSLAMAASDI--------sllDAQSAP-lRVYVEELKPTPeGDLEILLQKWengeCAQKKIIAEK----------------TKIP-----AVFK----idalneNKVLVlDTDYKKYLLFCMENSAepeqSLVCQCLVRTpevddealekfdkalkalpmhirlsfnptqleeqc

d1xcaa_ --------PNFSGNWKIIRSENFEEllkvlgvnvmlRKIAVAaaSKPAVEIKQEG-DTFYIKTSTT----VRTTEINFKVgeefeeqtvdgrpcksLVKWesenkMVCEqkllkgegpkTSWTM-ELTNDGELILTMTADD----VVCTRVYVRE-----------------------------------

d1beba_ qtmkgldiqKVAGTWYSLAMAAsdislldaqsAPLRVYVEELKPTPEGDLEILLQKweNGECAQKKIIAEKTKIPAVFKIDalnENKVLVLDTDYKKYLLFCMENSAEPeqSLVCQCLVRTPEVDDEALEKFDKALKALPM--HIRLSFNPtqleeqc

d1xkia_ ---------DVSGTWYLKAMTV----------NLESVTPMTLTTLEGGNLEAKVTM--SGRCQEVKAVLEKTDEPGKYTAD-ggKHVAYIIRSHVKDHYIFYSEGEGKP--VRGVKLVGRDPKNNLEALEDFEKAAGARGLstESILIPRQ------s

d1beba_ ----QTMKGLDIQKVAGTWYSLAMAASDISLLDAQSAPLRVYVEELKPTPEGDLEILLQKWENGECAQKKIIAEKTKIPAVFKIDALNENKVLVLDTDYKKYLLFCMENSAEPEQSLVCQCLVRTPEVDDEALEKFDKALKALPMHIRLSFNPTQLEEQC--

d1yupa1 iivtQTMKDLDVQKVAGTWYSLAMAASDISLLDAQSAPLRVYVEELKPTPGGDLEILLQKWENGKCAQKKIIAEKTEIPAVFKIDALNENKVLVLDTDYKKYLLFCMENSAEPEQSLACQCLVRTPEVDDEAMEKFDKALKALPMHIRLSFNPTQLEEQCrv

d1beba_ --qtmkglDIQKVAGTWYSlAMAASD--ISLLdaqsaplRVYVEELKptPEGDLEILLQKW--eNGEC-AQKKIIAE--------------------kTKIP------AVFK------idalneNKVLVLDTDykKYLLFCMENS----AEPEqsLVCQCLVRTpevddealekfdkalkalpmhirlsfnptqleeqc

d2a13a1 ppvhpfvaPLSYLLGTWRG-QGEGEYptIPSF------rYGEEIRFShsGKPVIAYTQKTWkleSGAPxHAESGYFRprpdgsievviaqstglvevqKGTYnvdeqsIKLKsdlvgnaskvkeISREFELVD--GKLSYVVRXStttnPLQP--HLKAILDKL-----------------------------------

d1beba_ qtmkgldiQKVAGTWYSLAMAASDI--------sllDAQSAplrvYVEELKPTPeGDLEILLQKWengeCAQKKIIAEK----------------TKIP-----AVFKIDalneNKVLVlDTDYkKYLLFCMENSaepeQSLVCQCLVRTpevddealekfdkalkalpmhirlsfnptqleeqc

d2f73a1 --------MSFSGKYQLQSQENFEAfmkaiglpeelIQKGK-dikGVSEIVQNG-KHFKFTITAG----SKVIQNEFTVgeeceletmtgekvktVVQLegdnkLVTTFK---nIKSVT-ELNG-DIITNTMTLG----DIVFKRISKRI-----------------------------------

d1beba_ -qtmkglDIQKVAGTWYSlAMAASD--ISLLdaqsaplRVYVEELKPTP---EGDLEILLQKWEngECAQ-KKIIAEK------TKIP------------------------AVFK------------idalneNKVLVlDTDYkKYLLFCMENS----AEPEqsLVCQCLVRTPevddealekfdkalkalpmhirlsfnptqleeqc

d2fr2a1 dlapalqALSPLLGSWAG-RGAGKYptIRPF-------EYLEEVVFAHVgkpFLTYTQQTRAVA--DGKPlHSETGYLrvcrpgCVELvlahpsgiteievgtysvtgdvieLELStradgsiglaptakevtaLDRSY-RIDG-DELSYSLQMRavgqPLQD--HLAAVLHRQR----------------------------------

d1beba_ qtmkGLDIQKV-AGTWYSlAMAAsdislldaqsaplRVYVEELKPTpegdLEILLQK-wengeCAQKK-IIAEKT---kipAVFKIDA--lNENKVLVLDTdykkyLLFCMENSA--------------------------------epEQSLVCQCLVRtpevddealekfdkalkalpmhirlsfNPTQLEE--------------qc

d2gc9a1 xtktFKTLDDFlGTHFIY-TYDN------------gWEYEWYAKND----HTVDYRIhggxvaGRWVTdQKADIVxltegiYKISWTEptgTDVALDFXPN----eKKLHGTIFFpkwveehpeitvtyqnehidlxeqsrekyatypkLVVPEFANITY-----------xgdagqnnedviseapYKEXPNDirngkyfdqnyhrlnk

d1beba_ qtmkGLDIQKVAGTWYSlAMAASD---ISLLdaqsaplRVYVEELKPTP--EGDLEILLQKwengecAQKKII---------------aekTKIP------AVFK----idalNENKVLVlDTDYKKYLLFCMENS---AEPEqSLVCQCLVRTpevddealekfdkalkalpmhirlsfnptqleeqc

d2o62a1 -erpLLQINDLLGEWRG-QAVTIYrdlRPPD-------IYSTTLKIQLDdaGRLXQSTSFG------ERTITStatikgsivlfdqdpekqVQVLllpdgaSATSplkvqlrqPLFLEAG-WLIQSDLRQRXIRSYndkGEWV-SLTLVTEERV-----------------------------------

d1beba_ ------QTMKGLDIQKVA--GTWYSLAMAASDISLLDAQsaplrvYVEELKPTPEgDLEILLQKW--eNGECAQKKIIAEKTkIPAVFKIDA---------------lNENKVLVLDTDyKKYLLFCMENS--AEPEqSLVCQCLVRTP-EVDDE-ALEKFDKALKalpmhIRLSFNpTQLEEQ----------c

d2ofmx1 actknaIAQTGFNKDKYFngDVWYVTDYLDLEPDDVPKR----ycAALAAGTASG-KLKEALYHYdpkTQDTFYDVSELQVE-SLGKYTANFkkvdkngnvkvavtagNYYTFTVMYAD-DSSALIHTCLHkgNKDL-GDLYAVLNRNKdAAAGDkVKSAVSAATL--efsKFISTK-ENNCAYdndslkslltk

d1beba_ --qTMKG--LDIQKVAGTWYSLAMAASDISLLDaQSAPLRVYVEELKPTpEGDLEILLQKWENGECAQKKIIAEKTKIPAVFKIDALNENKVLVLDTDYKKYLLFCMENS--AEPEqsLVCQCLVRTPEVDDEALEKFDKALKALPMH--IRLSFNPtQLEEqc

d2ozqa1 eeaSSTGrnFNVEKINGEWHTIILASDKREKIE-DNGNFRLFLEQIHVL-EKSLVLKFHTVRDEECSELSMVADKTEKAGEYSVTYDGFNTFTIPKTDYDNFLMAHLINEkdGETF--QLMGLYGREPDLSSDIKERFAQLCEEHGILreNIIDLSN-ANRC--

d1beba_ ------QTMKGLDIQKVA--GTWYSLAMAASDISllDAQSaplrvYVEELKPTPEgDLEILLQKWE--NGECAQKKIIAEKTkIPAVFKIDA---------------lNENKVLVLDTDyKKYLLFCMENS--AEPEqSLVCQCLVRTP-EVDDE-ALEKFDKALKAlpmhIRLSFNPtqlEEQC----------

d3np1a_ kctknaLAQTGFNKDKYFngDVWYVTDYLDLEPDdvPKRY----cAALAAGTASG-KLKEALYHYDpkTQDTFYDVSELQEE-SPGKYTANFkkvekngnvkvdvtsgNYYTFTVMYAD-DSSALIHTCLHkgNKDL-GDLYAVLNRNKdTNAGDkVKGAVTAASLK--fsDFISTKD-nkCEYDnvslkslltk

d1bj7a_ --------------IDPSKI-PGEWRIIY------------------aaadnkdkiVEGGPlrnyyrriecindceslsitfylkdqgTCLLLTEVAK--RQEGyVYVLEF------YGTNTLEVIHVS-ENMLVTYVENYDG-ERITKMTEGLAKGTSftpeelekyqqlnsergvpnenienliKTDNCPP--

d1avgi_ aegddcsiekamgdFKPEEFfNGTWYLAHgpgvtspavcqkfttsgskgftqiveiGYNKF--------------------------eSNVKFQCNQVdnKNGE-QYSFKCkssdntEFEADFTFISVSyDNFALVCRSITFTsQPKEDRYLVFERTKS--------------------------dTDPDAKEic

d1bj7a_ --------------IDPSKIPGEWRIIYAAAdNKDKiveggPLRN-YYRRIECINDcESLSITFYLKD-----QGTCLLLTEVAKRQ-EGYVYVLEF--------YGTNTLEVIHVS-ENMLVTYVENY---dgerITKMTEGLAKGT-SFTPEELEKYQQLNSERGVpNENIENLiKTDNCPp

d1brqa_ erdcrvssfrvkenFDKARFSGTWYAMAKKD-PEGL-----FLQDnIVAEFSVDET-GQMSATAKGRVrllnnWDVCADMVGTFTDTeDPAKFKMKYwgvasflqKGNDDHWIVDTDyDTYAVQYSCRLlnldgtcADSYSFVFSRDPnGLPPEAQKIVRQRQEELCL-ARQYRLI-VHNGYC-

d1bj7a_ idpSKIPGEWRIIYAA----------adnkdkiveggplRNYYRRIECINdcESLSITFYLKdqgtCLLLTEVAKRQ----------------------egYVYV----lefygtNTLEViHVSENMLVTYVENYdgerITKMTEGLAKGtsftpeelekyqqlnsergvpnenienliktdncpp

d1cbia_ ---PNFAGTWKMRSSEnfdellkalgvnamlrkvavaaaSKPHVEIRQDG--DQFYIKTSTT----VRTTEINFKVGegfeeetvdgrkcrslptwenenkIHCTqtllegdgpkTYWTR-ELANDELILTFGAD----DVVCTRIYVRE------------------------------------

d1bj7a_ idPSKIPGEWRIIYAAADNKDKIVEGGPLRNYYRRIECINDCESLSITFYLKDQGTCLLLTEVAKRQEGYVYVLEFYGTNTLEVIHVSENMLVTYVENYDG-ERITKMTEGLAKGTSFTPEELEKYQQLNSERGVPNENIENLIKTDNCPP

d1e5pa_ --FAELQGKWYTIVIAADNLEKIEEGGPLRFYFRHIDCYKNCSEXEITFYVITNNQCSKTTVIGYLKGNGTYETQFEGNNIFQPLYITSDKIFFTNKNXDRaGQETNXIVVAGKGNALTPEENEILVQFAHEKKIPVENILNILATDTCPE

d1bj7a_ ---IDPSKIPGEWRIIYAAADnkdkiveggplrnYYRRIECINdcESLSITFYLKDQGTCLLLTEVAKRQE-GYVYVLEFY-GTNTLEVIHV-SENMLVTYVENYDGERITKMTEGLAKGT-SFTPeELEKYQQLNSERGVPNENIENLIKTDNCPP------

d1epaa_ vkdFDISKFLGFWYEIAFASK---------eekmGAMVVELKE--NLLALTTTYYSEDHCVLEKVTATEGDgPAKFQVTRLsGKKEVVVEATdYLTYAIIDITSLVAGAVHRTMKLYSRSLdDNGE-ALYNFRKITSDHGFSETDLYILKHDLTCVKvlqsaa

d1bj7a_ ------------IDPSKIP-GEWRIIYAAADNKDkiVEGGplRNYYRRIECINdcESLSITFYLKD--QGTCLLLTEVAKRQ-EGYVYVLEF---------------yGTNTLEVIHVSENMLVTYVENYD-GERITKMTEGLAKG--TSFTPEELEKYQQLNSergvPNENIENLIKT-DNCPP-----

d1euoa_ mdcstnispkqgLDKAKYFsGKWYVTHFLDKDPQ--VTDQ--YCSSFTPRESD--GTVKEALYHYNanKKTSFYNIGEGKLEsSGLQYTAKYktvdkkkavlkeadekNSYTLTVLEADDSSALVHICLREgSKDLGDLYTVLTHQkdAEPSAKVKSAVTQAGL----QLSQFVGTKDLgCQYDDqftsl

d1bj7a_ -----IDPSKIPGEWRIIYAAADNKDKIVEGGPLRNYYRRIECINDcESLSITFYLKDQGTCLLLTEVAKRQ-EGYVYVLEFYGTNTLEVIHV-SENMLVTYVENYDGERITKMTEGLAKGTSFTPEELEKYQQLNSERGVPNENIENLIKT-DNCPP--

d1ew3a_ vairnFDISKISGEWYSIFLASDVKEKIEENGSMRVFVDVIRALDN-SSLYAEYQTKVNGECTEFPMVFDKTeEDGVYSLNYDGYNVFRISEFeNDEHIILYLVNFDKDRPFQLFEFYAREPDVSPEIKEEFVKIVQKRGIVKENIIDLTKIdRCFQLrg

d1bj7a_ ---------IDPSKIPGEWRIIYAAAD-NKDKiveGGPLR-NYYRRIECINDcESLSITFYLKDQGTCLLLTEVAKRQ-EGYVYVLEFYGTNTLEVIHV-sENMLVTYVENYdgERIT--KMTEGLAKGTSFTPEELEKYQQLNSERGVPNeNIENliKTDNCPP

d1exsa_ vevtpimteLDTQKVAGTWHTVAMAVSdVSLL-daKSSPLkAYVEGLKPTPE-GDLEILLQKRENDKCAQEVLLAKKTdIPAVFKINALDENQLFLLDTdyDSHLLLCMENS--ASPEhsLVCQSLARTLEVDDQIREKFEDALKTLSVPM-RILPaqLEEQCRV

d1bj7a_ idPSKIPG-EWRIIYAAA--DNKD------kiVEGGplrnYYRRIECINdcESLSITFYLKdqgtCLLLTEV------------------aKRQEG----YVYVLefyGTNTLEViHVSENMLVTYVENYdgerITKMTEGLAKGtsftpeelekyqqlnsergvpnenienliktdncpp

d1ftpa_ --VKEFAGiKYKLDSQTNfeEYMKaigvgaieRKAGlalsPVIELEILD-gDKFKLTSKTA----IKNTEFTfklgeefdeetldgrkvksTITQDgpnkLVHEQkgdHPTIIIR-EFSKEQCVITIKLG----DLVATRIYKAQ------------------------------------

d1bj7a_ idPSKIPGEWRIIYAAA--DNKD------kiVEGGPlRNYYRRIECINDCesLSITFYLKdqgtCLLLTEV--------------------aKRQEG---YVYV-lefyGTNTLEViHVSENMLVTYVENYdgerITKMTEGLAKGtsftpeelekyqqlnsergvpnenienliktdncpp

d1ggla_ --PPNLTGYYRFVSQKNmeDYLQalnislavRKIALlLKPDKEIEHQGNH--MTVRTLST----FRNYTVQfdvgvefeedlrsvdgrkcqtIVTWEeehLVCVqkgevPNRGWRH-WLEGEMLYLELTAR----DAVCEQVFRKV-----------------------------------h

d1bj7a_ -----IDPSKIPGEWRIIYAAADNKDKIVEGGPLRNYYRRIECINDcESLSITFYLKDQGTCLLLTEVAKRQEGYVYVLEFYGTNTLEVIHVS-ENMLVTYVENYDGERITKMTEGLAKGTSFTPEELEKYQQLNSERGVPNENIENLIKT-DNCPP--

d1gm6a_ vvtsnFDASKIAGEWYSILLASDAKENIEENGSMRVFVEHIRVLDN-SSLAFKFQRKVNGECTDFYAVCDKVGDGVYTVAYYGENKFRLLEVNySDYVILHLVDVNGDKTFQLMEFYGRKPDVEPKLKDKFVEICQQYGIIKENIIDLTKIdRCFQLrg

d1bj7a_ --------------IDPSKIPGEWRIIYAAAdNKDKiveggPLRN-YYRRIECINDCEsLSITFYLKD-----QGTCLLLTEVAKRQ-EGYVYVLEF--------YGTNTLEVIHVS-ENMLVTYVENYD---gerITKMTEGLAKGT-SFTPEELEKYQQLNSERGVPnENIENLIKTDNCPP

d1hbqa_ erdcrvssfrvkenFDKARFAGTWYAMAKKD-PEGL-----FLQDnIVAEFSVDENGQ-MSATAKGRVrllnnWDVCADMVGTFTDTeDPAKFKMKYwgvasflqKGNDDHWIIDTDyETFAVQYSCRLLnldgtcADSYSFVFARDPsGFSPEVQKIVRQRQEELCLA-RQYRLIPHNGYCNG

d1bj7a_ ----------IDPS--KIPGEWRIIYAAADNkdkiveggplrnYYRRIECINdcESLSITFYLKDQGTCLLLTEVAKRQ-EGYVYVLEF---YGTNTLEVIHVS-ENMLVTYVENYdgerITKMTEGLAKGTSFTPEELEKYQQLNSERGVPNENIENLIKTdNCPP-----------

d1iw2a_ aspistiqpkANFDaqQFAGTWLLVAVGSAG--------raeaTTLHVAPQG--TAMAVSTFRKLDGICWQVRQLYGDTgVLGRFLLQArgaRGAVHVVVAETDyQSFAVLYLERA----GQLSVKLYARSLPVSDSVLSGFEQRVQEAHLTEDQIFYFPKY-GFCEaadqfhvldev

d1bj7a_ --------------IDPSKIPGEWRIIYAAAdNKDKiveggPLRN-YYRRIECINDCeSLSITFYLKD-----QGTCLLLTEVAKRQ-EGYVYVLEF--------YGTNTLEVIHV-SENMLVTYVENY---dgerITKMTEGLAKGT-SFTPEELEKYQQLNSERGVpNENIENLIKTDNCpp

d1jyda_ erdcrvssfrvkenFDKARFSGTWYAMAKKD-PEGL-----FLQDnIVAEFSVDETG-QMSATAKGRVrllnnWDVCADMVGTFTDTeDPAKFKMKYwgvasflqKGNDDHWIVDTdYDTYAVQYSCRLlnldgtcADSYSFVFSRDPnGLPPEAQKIVRQRQEELCL-ARQYRLIVHNGYC--

d1bj7a_ idPSKIPGEWRIIYAAA--DNKD------kiVEGGPlRNYYRRIECINdcESLSITFYLKdqgtCLLLTEVAK--------------------RQEG---YVYV-lefygTNTLEViHVSENMLVTYVENYdgerITKMTEGLAKGtsftpeelekyqqlnsergvpnenienliktdncpp

d1kqxa_ --PADFNGTWEMLSNDNfeDVMKaldidfatRKIAVhLKQTKVIVQNG--DKFETKTLST----FRNYEVNFVigeefdeqtkgldnrtvktlVKWDgdkLVCVqkgekeNRGWKQ-WIEGDLLHLEIHCQ----DKVCHQVFKKK-----------------------------------n

d1bj7a_ idPSKIPGEWRIIYAAA--DNKD------kiVEGGplrNYYRRIECINDcesLSITFYLKdqgtCLLLTEV------------------aKRQEG---YVYVLE-fyGTNTLEViHVSENMLVTYVENYdgerITKMTEGLAKGtsftpeelekyqqlnsergvpnenienliktdncpp

d1liba_ --CDAFVGTWKLVSSENfdDYMKevgvgfatRKVAgmaKPNMIISVNGD--lVTIRSEST----FKNTEISfklgvefdeitaddrkvksIITLDggaLVQVQKwdgKSTTIKR-KRDGDKLVVECVMK----GVTSTRVYERA------------------------------------

d1bj7a_ idPSKIPGEWRIIYAAA--DNKD------kiVEGGPlRNYYRRIECINDCesLSITFYLKDqgtcLLLTEV--------------------aKRQEG---YVYV-lefyGTNTLEViHVSENMLVTYVENYdgerITKMTEGLAKGtsftpeelekyqqlnsergvpnenienliktdncpp

d1lpja_ --PADLSGTWTLLSSDNfeGYMLalgidfatRKIAKlLKPQKVIEQNGDS--FTIHTNSSL----RNYFVKfkvgeefdednrgldnrkcksLVIWDndrLTCIqkgekKNRGWTH-WIEGDKLHLEMFCE----GQVCKQTFQRA------------------------------------

d1bj7a_ -------------------------iDPSKIPGEWRIIYAAAdnkdkivegGPLRN---YYRRIECINdcESLSITFYLKD-QGTCLLLTEVAKRQ--EGYVYVLEF--YGTNTLEVIHV-SENMLVTYVENY-dgERITKMTEGLAKGTSFTPEELEKYQQLNSERGVPNENIENLIKT-dNCPP-----

d1obqa_ dkipdfvvpgkcasvdrnklwaeqtpNRNSYAGVWYQFALTN--------nPYQLIekcVRNEYSFDG--KQFVIESTGIAyDGNLLKRNGKLYPNpfGEPHLSIDYenSFAAPLVILETdYSNYACLYSCIDynfGYHSDFSFIFSRSANLADQYVKKCEAAFKNINVDTTRFVKTVQGssCPYDtqktl

d1bj7a_ -----------------------idPSKIPGEWRIIY----------------------aaadnkdkiveggpLRNYYRRIECindcesLSITFYLKdqgtCLLLTEVAKRQEG-------------YVYVLE---fygtNTLEVIH-----vSENMLVTYVENYD---geritkMTEGLAKGTS----FTPEELEKyqqlnsergvpnenienliktdncpp

d1oeja_ gkplteveqkaangvfddanvqnrtLSDWDGVWQSVYpllqsgkldpvfqkkadadktktfaeikdyyhkgyaTDIEMIGIED------GIVEFHRN----NETTSCKYDYDGYkiltyksgkkgvrYLFECKdpeskapKYIQFSDhiiaprKSSHFHIFMGNDSqqsllnemeNWPTYYPYQLsseeVVEEMMSH--------------------------

d1bj7a_ idPSKIPGEWRIIYAAA--DNKD------kiVEGGPlrnYYRRIECINdcESLSITFYLKDqgtcLLLTEVAK-------------------RQEG----YVYV-lefygTNTLEViHVSENMLVTYVENYdgerITKMTEGLAKGtsftpeelekyqqlnsergvpnenienliktdncpp

d1opaa_ --TKDQNGTWEMESNENfeGYMKaldidfatRKIAVrltQTKIIVQDG--DNFKTKTNSTF----RNYDLDFTvgvefdehtkgldgrnvktLVTWegntLVCVqkgekeNRGWKQ-WVEGDKLYLELTCG----DQVCRQVFKKK------------------------------------

d1bj7a_ idpsKIPGEWRIIYAAA--DNKD------kiVEGGplrnYYRRIECINdcESLSITFYLKdqgtCLLLTEVAK-----------------RQEG----YVYVlefygTNTLEViHVSENMLVTYVENYdgerITKMTEGLAKGtsftpeelekyqqlnsergvpnenienliktdncpp

d1p6pa_ ----AFNGTWNVYAQENyeNFLRtvglpediIKVAkdvnPVIEIEQNG--NEFVVTSKTP----KQTHSNSFTvgkeseitsmdgkkikvTVQLeggkLICK---sdKFSHIQ-EVNGDEMVEKITIG----SSTLTRKSKRV------------------------------------

d1bj7a_ ------------------------IDPSKIPGEWRIIYAAadnkdkivegGPLR---nYYRRIECINdcESLSITFYLKD--QGTCLLLTEVAKRQE---GYVYVLEFY--GTNTLEVIHVS--ENMLVTYVEnydgerITKMTEGLAKGTSFTPEELEKYQQLNSERGVPNENIENLIKTdncpp

d1qwda_ hlestslykkssstpprgvtvvnnFDAKRYLGTWYEIARF--------dhRFERglekVTATYSLRD-dGGLNVINKGYNpdRGMWQQSEGKAYFTGaptRAALKVSFFgpFYGGYNVIALDreYRHALVCGP------DRDYLWILSRTPTISDEVKQEMLAVATREGFDVSKFIWVQQP----g

d1bj7a_ ---idpskiPGEWRIiYAAADN------KDKIveggplRNYYRRIECINDCesLSITFYLKDQGTCLLLTEVAKRqegyVYVL------------------------------efYGTN-TLEVIhVSENMLVTYVENYDG-eRITKMTEGLAKGTsftpeelekyqqlnsergvpnenienliktdncpp

d1r0ua_ gfqsnamkqETPITL-HVKSVIeddgnqEVIE------FRTTGFYYVKQNK--VYLSYYEEHDLGKVKTIVKVSE----GEVLvmrsgavkmnqrfvtgastiakykmsfgelelKTSTkSIQSD-LDEEKGRISIAYDMHvgHLHNMTITYEGGT-----------------------------------

d1bj7a_ idpsKIPGEWRIIYAAA--DNKD------kiVEGGPlRNYYRRIECINDCesLSITFYLKdqgtCLLLTEV------------------aKRQEG---YVYVlefygTNTLEViHVSENMLVTYVENYdgerITKMTEGLAKGtsftpeelekyqqlnsergvpnenienliktdncpp

d1tvqa_ ----AFSGTWQVYAQENyeEFLKalalpedlIKMARdIKPIVEIQQKGDD--FVVTSKTP----RQTVTNSftlgkeadittmdgkklkcTVHLAngkLVTK---seKFSHEQ-EVKGNEMVETITFG----GVTLIRRSKRV------------------------------------

d1bj7a_ ---------------------------------------------------------------------------------------------------------------------------------------------------------idPSKIPgEWRIiYAAA------------------dnkdkiveGGPL--RNYYRRIECIndCESLSITFYLKDqgtcLLLTEV--------------------AKRQEGYVYVLEF-------yGTNTLEVIHvsenMLVTYVENYDGERITKMTEGLAK---------gtsftpeelekyqqlnsergvpnenienliktdncpp

d1vpra1 ekgfeagdnklggalnakhvekygdnfkngxhkpefhedglhkpxevggkkfesgfhyllechelggknasggyggplcedpygsevqaxtekllkeadsdrtlcfnnfqdpcpqltkeqvaxckgfdygdktlklpcgplpwpaglpepgyvpkTNPLH-GRWI-TVSGgqaafikeaiksgmlgaaeankivadTDHHqtGGXYLRINQF--GDVCTVDASVAK---fARAKRTwksghyfyeplvsggnllgvWVLPEEYRKIGFFwexesgrcFRIERRAFP-vgpYTFXRQATEVGGKISFVFYVKVSndpesdpiplqsrdytalagrdnaptnlgkpyptlakdldypkkrd

d1bj7a_ idpSKIPGEWRIIYAAA--DNKD-------kivEGGP-lRNYYRRIECINDCesLSITFYLKdqgtCLLLTEV------------------aKRQEG----YVYV----lefygTNTLEViHVSE-NMLVTYVENYdgerITKMTEGLAKGtsftpeelekyqqlnsergvpnenienliktdncpp

d1xcaa_ ---PNFSGNWKIIRSENfeELLKvlgvnvmlrkIAVAaaSKPAVEIKQEGDT--FYIKTSTT----VRTTEINfkvgeefeeqtvdgrpcksLVKWEsenkMVCEqkllkgegpKTSWTM-ELTNdGELILTMTAD----DVVCTRVYVRE------------------------------------

d1bj7a_ idpsKIPGEWRIIYAAAdnkdkivegGPLRNYYRRIECINDcESLSITFYLkdQGTCLLLTEVAKRQ-EGYVYVLEfygTNTLEVIHV-SENMLVTYVENYdgERITKMTEGLAKG--TSFTpeELEKYQQLNSERGVPNENIENlIKTDncpp

d1xkia_ ----DVSGTWYLKAMTV---------NLESVTPMTLTTLEG-GNLEAKVTM--SGRCQEVKAVLEKTdEPGKYTAD-ggKHVAYIIRShVKDHYIFYSEGE--GKPVRGVKLVGRDpkNNLE--ALEDFEKAAGARGLSTESILI-PRQS----

d1bj7a_ ---------IDPSKIPGEWRIIYAAADNKDKIV-EGGPLRNYYRRIECINDcESLSITFYLKDQGTCLLLTEVAKRQ-EGYVYVLEFYGTNTLEVIHVS-ENMLVTYVENYDGeRITKMTEGLAKGTSFTPEELEKYQQLNSERGVPneNIENL----IKTDNCpp

d1yupa1 iivtqtmkdLDVQKVAGTWYSLAMAASDISLLDaQSAPLRVYVEELKPTPG-GDLEILLQKWENGKCAQKKIIAEKTeIPAVFKIDALNENKVLVLDTDyKKYLLFCMENSAEpEQSLACQCLVRTPEVDDEAMEKFDKALKALPMH--IRLSFnptqLEEQCR-v

d1bj7a_ -------iDPSKIPGEWRIiYAAADN--KDKIveggplRNYYRRIE---ciNDCEsLSITFYLKDqgTCLL-LTEV-----------------------akRQEG------YVYVL------efyGTNTLEViHVSENMLVTYVENYDGER--ITKMTEGLAKGtsftpeelekyqqlnsergvpnenienliktdncpp

d2a13a1 ppvhpfvaPLSYLLGTWRG-QGEGEYptIPSF------RYGEEIRFshsgkPVIA-YTQKTWKLE--SGAPxHAESgyfrprpdgsievviaqstglvevqKGTYnvdeqsIKLKSdlvgnaskvKEISREF-ELVDGKLSYVVRXSTTTNplQPHLKAILDKL------------------------------------

d1bj7a_ idpSKIPGEWRIIYAAAdnKDKI---------VEGGplrnYYRRIECINDCesLSITFYLKdqgtCLLLTEVA------------------KRQEG----YVYVlefyGTNTLEViHVSENMLVTYVENYdgerITKMTEGLAKGtsftpeelekyqqlnsergvpnenienliktdncpp

d2f73a1 ---MSFSGKYQLQSQEN-fEAFMkaiglpeelIQKGkdikGVSEIVQNGKH--FKFTITAG----SKVIQNEFtvgeeceletmtgekvktVVQLEgdnkLVTT---fKNIKSVT-ELNGDIITNTMTLG----DIVFKRISKRI------------------------------------

d1bj7a_ ------IDPSKIPGEWriiyaaadnkdkiVEGG------plrnYYRRIECINDcESLSITFYLKD---QGTCLLLTEVAKRQEGYVYVLEF------YGTNTLEViHVSENMLVTYVEN----YDGE-------RITKMTEGLAKGTsftpeelekyqqlnsergvpneniENLIktdncpp

d2fr2a1 dlapalQALSPLLGSW----------agrGAGKyptirpfeylEEVVFAHVGK-PFLTYTQQTRAvadGKPLHSETGYLRVCRPGCVELVLahpsgiTEIEVGTY-SVTGDVIELELSTradgSIGLaptakevTALDRSYRIDGDE------lsyslqmravgqplqdhlAAVL---hrqr

d1bj7a_ -----IDPSKIPGEWRIIYAAAdnkdkiveggpLRNYYrRIECIndcesLSITFYLK-dqgtCLLLT-EVAKRQEG----YVYVLEF--YGTNTLEVIHVsenmLVTYVENYDG--------------------------------ERITKMTEG-LAKGtsftpeelekyqqlnsergvpnenienliKTDN--------------cpp

d2gc9a1 xtktfKTLDDFLGTHFIYTYDN----------gWEYEW-YAKND-----HTVDYRIHggxvaGRWVTdQKADIVXLtegiYKISWTEptGTDVALDFXPN---eKKLHGTIFFPkwveehpeitvtyqnehidlxeqsrekyatypKLVVPEFANiTYXG-------------dagqnnedviseapykEXPNdirngkyfdqnyhrlnk

d1bj7a_ ----IDPSKIPGEWRIiYAAADN----KDKIveggplRNYYRRIECINdcesLSITFYLkdqgtCLLLTEV----------------aKRQEG-----YVYV----lefyGTNTLEVIHVSENMLVTYVENYD--GERITKMTEGLAKGtsftpeelekyqqlnsergvpnenienliktdncpp

d2o62a1 erplLQINDLLGEWRG-QAVTIYrdlrPPDI------YSTTLKIQLDD-agrLXQSTSF----gERTITSTatikgsivlfdqdpekqVQVLLlpdgaSATSplkvqlrqPLFLEAGWLIQSDLRQRXIRSYNdkGEWVSLTLVTEERV------------------------------------

d1bj7a_ -----------IDPSKIP--GEWRIIYAAADNKDKIVEGgplRNYYRRIECINDcESLSITFYLKDQGTCLLLTEV-AKRQEGYVYVLEF---------------yGTNTLEVIHVSENMLVTYVENYDGERI-TKMTEGLAKGTSFTpeELEKYQQLNSERGVPNENIENLIKT-DNCPP---------

d2ofmx1 actknaiaqtgFNKDKYFngDVWYVTDYLDLEPDDVPKR---YCAALAAGTASG-KLKEALYHYDPKTQDTFYDVSeLQVESLGKYTANFkkvdkngnvkvavtagNYYTFTVMYADDSSALIHTCLHKGNKDlGDLYAVLNRNKDAA--AGDKVKSAVSAATLEFSKFISTKENnCAYDNdslkslltk

d1bj7a_ ---------IDPSKIPGEWRIIYAAADNKDKIVEGGPLRNYYRRIECINdcESLSITFYLKDQGTCLLLTEVAKRQ-EGYVYVLEFYGTNTLEVIHVS-ENMLVTYVENYDGERITKMTEGLAKGTSFTPEELEKYQQLNSERGVPNENIENLIKTDNCpp

d2ozqa1 eeasstgrnFNVEKINGEWHTIILASDKREKIEDNGNFRLFLEQIHVLE--KSLVLKFHTVRDEECSELSMVADKTeKAGEYSVTYDGFNTFTIPKTDyDNFLMAHLINEKDGETFQLMGLYGREPDLSSDIKERFAQLCEEHGILRENIIDLSNANRC--

d1bj7a_ -----------IDPSKIP--GEWRIIYAAADNKDkiVEGGplrnYYRRIECINDceSLSITFYLKD--QGTCLLLTEVAKRQEGYVYVLEF---------------yGTNTLEVIHVSENMLVTYVENYD-GERITKMTEGLAKGTSFTpeELEKYQQLNSERGVPNENIENLIKT-DNCPP---------

d3np1a_ kctknalaqtgFNKDKYFngDVWYVTDYLDLEPDdvPKRY---cAALAAGTASG--KLKEALYHYDpkTQDTFYDVSELQEESPGKYTANFkkvekngnvkvdvtsgNYYTFTVMYADDSSALIHTCLHKgNKDLGDLYAVLNRNKDTN--AGDKVKGAVTAASLKFSDFISTKDNkCEYDNvslkslltk

d1brqa_ erDCRV-SSFRVKENFDKARFS-GTWYAMAkkdpeglflQDNIvaefsVDETGQmsatakgrvrllnnwdVCADMVGTFTD-TEDPaKFKMKYWGVAsfLQKGNDDHWIVDTDYDTYAVQYSCRllnldgtcADSYSFVFSRDPNglppeaqkivrqrqeelclarqyrliVHNGYC--

d1avgi_ -aEGDDcSIEKAMGDFKPEEFFnGTWYLAH---gpgvtsPAVC-qkftTSGSKG---ftqiveigynkfeSNVKFQCNQVDnKNGE-QYSFKCKSSD--NTEFEADFTFISVSYDNFALVCRSI--tftsqpKEDRYLVFERTKS------------------------dtDPDAKEic

d1brqa_ erdcrvssfrvkenfdkaRFSGTWYAMAKK---------------dpeglflqDNIVAEFSVDEtGQMSATAKGRvrllnnwdvCADMVGTF------------------TDTEDP---AKFKmkywgvasflqkgnDDHWIvDTDYdTYAVQYSCRLlnldgtcADSYSFVFSRDpnglppeaqkivrqrqeelclarqyrlivhngyc

d1cbia_ -----------------pNFAGTWKMRSSEnfdellkalgvnamlrkvavaaaSKPHVEIRQDG-DQFYIKTSTT---------VRTTEINFkvgegfeeetvdgrkcrsLPTWENenkIHCT----qtllegdgpkTYWTR-ELAN-DELILTFGAD-------DVVCTRIYVRE----------------------------------

d1brqa_ erdcrvssfrvkenfdKARFSGTWYAMAKKD-PEGL-----FLQDnIVAEFSVDET-GQMSATAKGRVrllnnWDVCADMVGTFTDTEDPaKFKMKYwgvasflqKGNDDHWIVDTDyDTYAVQYSCRLlnldgtcADSYSFVFSRDpNGLPPEAQKIVRQRQEELCL-ARQYRLI-VHNGYC-

d1e5pa_ ----------------FAELQGKWYTIVIAAdNLEKieeggPLRF-YFRHIDCYKNcSEXEITFYVIT-----NNQCSKTTVIGYLKGNG-TYETQF--------EGNNIFQPLYIT-SDKIFFTNKNX--dragqETNXIVVAGKG-NALTPEENEILVQFAHEKKIpVENILNIlATDTCPe

d1brqa_ erdcrvssfrvKENFDKARFSGTWYAMAKKDPeglflQDNIVAEFSVDeTGQMSATAKGRVRllnnwDVCADMVGTFTDTEDPAKFKMKYwgvasflqkGNDDHWIVDTDYDTYAVQYSCRLlnldgtcADSYSFVFSRDPNGLPpEAQKIVRQRQEELCLAR-QYRLIVHNGYC--------

d1epaa_ -----------VKDFDISKFLGFWYEIAFASK----eEKMGAMVVELK-ENLLALTTTYYSE-----DHCVLEKVTATEGDGPAKFQVTR-------lsGKKEVVVEATDYLTYAIIDITSL---vagaVHRTMKLYSRSLDDNG-EALYNFRKITSDHGFSEtDLYILKHDLTCvkvlqsaa

d1brqa_ erdcrVSSFRVKENFDKARFS-GTWYAMAKKDPEG-lflQDNIVAEFSVDeTGQMSATAKGRVRLlnnWDVCADMVGTFTDTEDPAKFKMKYWGVA----SFLQK---GNDDHWIVDTDyDTYAVQYSCRLlnldgtcADSYSFVFSRDPNGLppeAQKIVRQRQEELCLA-RQYRLIV-HNGYC-------

d1euoa_ --mdcSTNISPKQGLDKAKYFsGKWYVTHFLDKDPqvtdQYCSSFTPRES-DGTVKEALYHYNAN---KKTSFYNIGEGKLESSGLQYTAKYKTVDkkkaVLKEAdekNSYTLTVLEAD-DSSALVHICLR--egskdLGDLYTVLTHQKDAE---PSAKVKSAVTQAGLQlSQFVGTKdLGCQYddqftsl

d1brqa_ erdcrvssfrvKENFDKARFSGTWYAMAKKD-PEGL-----FLQDnIVAEFSVDETGQMSATAKGRVrllnnWDVCADMVGTFTDTEDPAKFKMKYwgvasflqKGNDDHWIVDTDYDTYAVQYSCRLLNLdgtcADSYSFVFSRDPnGLPPEAQKIVRQRQEELCLAR-QYRLIV---HNGYC--

d1ew3a_ ---------vaIRNFDISKISGEWYSIFLASdVKEKieengSMRV-FVDVIRALDNSSLYAEYQTKV-----NGECTEFPMVFDKTEEDGVYSLNY--------DGYNVFRISEFENDEHIILYLVNFDKD---rPFQLFEFYAREP-DVSPEIKEEFVKIVQKRGIVKeNIIDLTkidRCFQLrg

d1brqa_ erdcrvssFRVKENFDKARFSGTWYAMAKKD----PEGL--FLQDNIVAEFSVDETGQMSATAKGRVRllnnwDVCADMVGTFTDTEDPAKFKMKYwgvasflqkgnDDHWIVDTDyDTYAVQYSCRLLNldgtCADSYSFVFSRDPNGlPPEAQKIVRQRQEELCLARQYRLiVHNGYC--

d1exsa_ -----vevTPIMTELDTQKVAGTWHTVAMAVsdvsLLDAksSPLKAYVEGLKPTPEGDLEILLQKREN-----DKCAQEVLLAKKTDIPAVFKINA--------ldeNQLFLLDTDyDSHLLLCMENSAS---pEHSLVCQSLARTLEV-DDQIREKFEDALKTLSVPMRILPaQLEEQCrv

d1brqa_ erdcrvssfrvkenfdKARFSG-TWYAMAKK-DPEGL------------FLQDNIVAEFSVDETGQMSATAKGRvrllnnwdvCADMVGTF------------------TDTEDP---AKFKmkywgvasflqkgnDDHWIvDTDYdTYAVQYSCRLlnldgtcADSYSFVFSRDpnglppeaqkivrqrqeelclarqyrlivhngyc

d1ftpa_ ----------------VKEFAGiKYKLDSQTnFEEYMkaigvgaierkaGLALSPVIELEILDGDKFKLTSKTA---------IKNTEFTFklgeefdeetldgrkvksTITQDGpnkLVHE--------qkgdhpTIIIR-EFSK-EQCVITIKLG-------DLVATRIYKAQ----------------------------------

d1brqa_ erdcrvssfrvkenfdkARFSGTWYAMAKKDPEG-------------lFLQDNIVAEFSVDEtGQMSATAKGRvrllnnwdvCADMVGT--------------------fTDTED--pAKFKmkywgvasflqkgnDDHWIvDTDYdTYAVQYSCRLlnldgtcADSYSFVFSRDpnglppeaqkivrqrqeelclarqyrlivhngyc

d1ggla_ ----------------pPNLTGYYRFVSQKNMEDylqalnislavrkiALLLKPDKEIEHQG-NHMTVRTLST---------FRNYTVQfdvgvefeedlrsvdgrkcqtIVTWEeehLVCV-------qkgevpnRGWRH-WLEG-EMLYLELTAR-------DAVCEQVFRKV---------------------------------h

d1brqa_ erdcrvssfrvKENFDKARFSGTWYAMAKKD-PEGL-----FLQDnIVAEFSVDETGQMSATAKGRVrllnnWDVCADMVGTFTDTeDPAKFKMKYwgvasflqKGNDDHWIVDTDYDTYAVQYSCRLlnldgtcADSYSFVFSRDPnGLPPEAQKIVRQRQEELCLAR-QYRLIV---HNGYC--

d1gm6a_ ---------vvTSNFDASKIAGEWYSILLASdAKENieengSMRV-FVEHIRVLDNSSLAFKFQRKV-----NGECTDFYAVCDKV-GDGVYTVAY--------YGENKFRLLEVNYSDYVILHLVDV---ngdkTFQLMEFYGRKP-DVEPKLKDKFVEICQQYGIIKeNIIDLTkidRCFQLrg

d1brqa_ ERDCRVSSFRVKENFDKARFSGTWYAMAKKDPEGLFLQDNIVAEFSVDETGQMSATAKGRVRLLNNWDVCADMVGTFTDTEDPAKFKMKYWGVASFLQKGNDDHWIVDTDYDTYAVQYSCRLLNLDGTCADSYSFVFSRDPNGLPPEAQKIVRQRQEELCLARQYRLIVHNGYC--

d1hbqa_ ERDCRVSSFRVKENFDKARFAGTWYAMAKKDPEGLFLQDNIVAEFSVDENGQMSATAKGRVRLLNNWDVCADMVGTFTDTEDPAKFKMKYWGVASFLQKGNDDHWIIDTDYETFAVQYSCRLLNLDGTCADSYSFVFARDPSGFSPEVQKIVRQRQEELCLARQYRLIPHNGYCng

d1brqa_ erdcRVSSFRVKENFDKARFSGTWYAMAKKDPEglflQDNIVAEFSVDeTGQMSATAKGRVRllnnwDVCADMVGTFTDTEDPAKFKMKywgvasflqKGNDDHWIVDTDYDTYAVQYSCRLLnldgtcaDSYSFVFSRDpNGLPPEAQKIVRQRQEELCLAR-QYRLIVHNGYC------------

d1iw2a_ --asPISTIQPKANFDAQQFAGTWLLVAVGSAG---rAEATTLHVAPQ-GTAMAVSTFRKLD-----GICWQVRQLYGDTGVLGRFLLQ-----argaRGAVHVVVAETDYQSFAVLYLERAG-------QLSVKLYARS-LPVSDSVLSGFEQRVQEAHLTEdQIFYFPKYGFCeaadqfhvldev

d1brqa_ ERDCRVSSFRVKENFDKARFSGTWYAMAKKDPEGLFLQDNIVAEFSVDETGQMSATAKGRVRLLNNWDVCADMVGTFTDTEDPAKFKMKYWGVASFLQKGNDDHWIVDTDYDTYAVQYSCRLLNLDGTCADSYSFVFSRDPNGLPPEAQKIVRQRQEELCLARQYRLIVHNGYC

d1jyda_ ERDCRVSSFRVKENFDKARFSGTWYAMAKKDPEGLFLQDNIVAEFSVDETGQMSATAKGRVRLLNNWDVCADMVGTFTDTEDPAKFKMKYWGVASFLQKGNDDHWIVDTDYDTYAVQYSCRLLNLDGTCADSYSFVFSRDPNGLPPEAQKIVRQRQEELCLARQYRLIVHNGYC

d1brqa_ erdcrvssfrvkenfdkARFSGTWYAMAKK-DPEGL------------FLQDNIVAEFSVDEtGQMSATAKGRvrllnnwdvCADMVGTF--------------------TDTEDP--AKFKmkywgvasflqkgnDDHWIvDTDYdTYAVQYSCRLlnldgtcADSYSFVFSRDpnglppeaqkivrqrqeelclarqyrlivhngyc

d1kqxa_ ----------------pADFNGTWEMLSNDnFEDVMkaldidfatrkiAVHLKQTKVIVQNG-DKFETKTLST---------FRNYEVNFvigeefdeqtkgldnrtvktLVKWDGdkLVCV-------qkgekenRGWKQ-WIEG-DLLHLEIHCQ-------DKVCHQVFKKK---------------------------------n

d1brqa_ erdcrvssfrvkenfdKARFSGTWYAMAKK-DPEGL------------FLQDNIVAEFSVDETgQMSATAKGRvrllnnwdvCADMVGT------------------fTDTEDP--AKFKmkywgvasflqkgNDDHWIVDTDydTYAVQYSCRLlnldgtcADSYSFVFSRDpnglppeaqkivrqrqeelclarqyrlivhngyc

d1liba_ ----------------CDAFVGTWKLVSSEnFDDYMkevgvgfatrkvAGMAKPNMIISVNGD-LVTIRSEST---------FKNTEISfklgvefdeitaddrkvksIITLDGgaLVQV-------qkwdgkSTTIKRKRDG--DKLVVECVMK-------GVTSTRVYERA----------------------------------

d1brqa_ erdcrvssfrvkenfdkaRFSGTWYAMAKK-DPEGL------------flqdNIVAEFSVDEtGQMSATAKGRvrllnnwdvCADMVGTF--------------------TDTEDP--AKFKmkywgvasflqkgnDDHWIvDTDYdTYAVQYSCRLlnldgtcADSYSFVFSRDpnglppeaqkivrqrqeelclarqyrlivhngyc

d1lpja_ ----------------paDLSGTWTLLSSDnFEGYMlalgidfatrkiakllKPQKVIEQNG-DSFTIHTNSS---------LRNYFVKFkvgeefdednrgldnrkcksLVIWDNdrLTCI-------qkgekknRGWTH-WIEG-DKLHLEMFCE-------GQVCKQTFQRA----------------------------------

d1brqa_ ------erdCRVSSFR---VKENF--DKARFSGTWYAMAKKDPEGLFLQDNIVAEFSVDeTGQMSATAKGRVRLlnnwDVCADMVGTFTDT--EDPAkFKMKYWgvasflQKGNDDHWIVDTDYDTYAVQYSCrLLNLDGTCaDSYSFVFSRDPnGLPPEAQKIVRQRQEELCL-ARQYRLIVH--NGYC------

d1obqa_ dkipdfvvpGKCASVDrnkLWAEQtpNRNSYAGVWYQFALTNNPYQLIEKCVRNEYSFD-GKQFVIESTGIAYD----GNLLKRNGKLYPNpfGEPH-LSIDYE------NSFAAPLVILETDYSNYACLYSC-IDYNFGYH-SDFSFIFSRSA-NLADQYVKKCEAAFKNINVdTTRFVKTVQgsSCPYdtqktl

d1brqa_ ---------erdcrvssfrvkENFDKARFSGTWYAMA---------------------------kkdpeglflqdnIVAEFSVdetgqMSATAKGRvrllnnwdvCADMVGTFTDTED------------pAKFKMkywgvasflQKGN-DDHWIVD----tdYDTYAVQYSCRLlnldgtcadsYSFVFSR-DPNG--LPPEAQKIvrqrqeelclarqyrlivhngyc

d1oeja_ gkplteveqkaangvfddanvQNRTLSDWDGVWQSVYpllqsgkldpvfqkkadadktktfaeikdyyhkgyatdiEMIGIED-----GIVEFHRN---------NETTSCKYDYDGYkiltyksgkkgvrYLFEC------kdpESKApKYIQFSDhiiaprKSSHFHIFMGNDsqqsllnemeNWPTYYPyQLSSeeVVEEMMSH-----------------------

d1brqa_ erdcrvssfrvkenfdkARFSGTWYAMAKK-DPEGL------------FLQDNIVAEFSVDEtGQMSATAKGRvrllnnwdvCADMVGTF--------------------TDTED--PAKFKmkywgvasflqkgnDDHWIvDTDYdTYAVQYSCRLlnldgtcADSYSFVFSRDpnglppeaqkivrqrqeelclarqyrlivhngyc

d1opaa_ ----------------tKDQNGTWEMESNEnFEGYMkaldidfatrkiAVRLTQTKIIVQDG-DNFKTKTNST---------FRNYDLDFtvgvefdehtkgldgrnvktLVTWEgnTLVCV-------qkgekenRGWKQ-WVEG-DKLYLELTCG-------DQVCRQVFKKK----------------------------------

d1brqa_ erdcrvssfrvkenfdkaRFSGTWYAMAKKD---PEGL----------FLQDNIVAEFSVDetGQMSATAKGRvrllnnwdvCADMVGTFTD----------------TEDP----AKFKmkywgvasflqkgnDDHWIvDTDYdTYAVQYSCRLlnldgtcADSYSFVFSRDpnglppeaqkivrqrqeelclarqyrlivhngyc

d1p6pa_ ------------------AFNGTWNVYAQENyenFLRTvglpediikvAKDVNPVIEIEQN-gNEFVVTSKTP---------KQTHSNSFTVgkeseitsmdgkkikvTVQLeggkLICK-----------sdkFSHIQ-EVNG-DEMVEKITIG-------SSTLTRKSKRV----------------------------------

d1brqa_ ----------erdcrVSSFRVKENFDKARFSGTWYAMAKKDPEGLFLQDNIVAEFSVDETGQMSATAKGRVRLlnnWDVCADMVGTFTDTED--PAKFKMKYWgvasflQKGNDDHWIVDTDY-DTYAVQYSCRllnldgtcadSYSFVFSRDPnGLPPEAQKIVRQRQEELCL-ARQYRLIVHNgyc

d1qwda_ hlestslykkssstpPRGVTVVNNFDAKRYLGTWYEIARFDHRFERGLEKVTATYSLRDDGGLNVINKGYNPD---RGMWQQSEGKAYFTGAptRAALKVSFF------GPFYGGYNVIALDReYRHALVCGPD---------rDYLWILSRTP-TISDEVKQEMLAVATREGFdVSKFIWVQQP--g

d1brqa_ erdcrvssfrvkenfdkARFSGTWYAmAKKDPE-----glflqDNIVAEFSVDEtGQMSATAKGRVRllnnwDVCADMVGTFTDtedpakFKMKYWGVAsfLQKGND------------------------dHWIVdtdYDTYAVQYSCRLLNLDgtCADSYSFVFSRDPnglppeaqkivrqrqeelclarqyrlivhngyc

d1r0ua_ -----------gfqsnaMKQETPITL-HVKSVIeddgnqevieFRTTGFYYVKQ-NKVYLSYYEEHD-----LGKVKTIVKVSE------GEVLVMRSGavKMNQRFvtgastiakykmsfgelelktstksIQSD--lDEEKGRISIAYDMHVG--HLHNMTITYEGGT---------------------------------

d1brqa_ erdcrvssfrvkenfdkaRFSGTWYAMAKK-DPEGL------------FLQDNIVAEFSVDEtGQMSATAKGRvrllnnwdvCADMVGTF------------------tDTEDP--AKFKmkywgvasflqkgnDDHWIvDTDYdTYAVQYSCRLlnldgtcADSYSFVFSRDpnglppeaqkivrqrqeelclarqyrlivhngyc

d1tvqa_ ------------------AFSGTWQVYAQEnYEEFLkalalpedlikmARDIKPIVEIQQKG-DDFVVTSKTP---------RQTVTNSFtlgkeadittmdgkklkctVHLANgkLVTK-----------sekFSHEQ-EVKG-NEMVETITFG-------GVTLIRRSKRV----------------------------------

d1brqa_ ----------------------------------------------------------------------------------------------erdcrvSSFRV---------------------------------------------kenfdkARFSGTWYAMAKKD---------------------pEGLFL--qdnIVAEFSVDEtGQMSATAKGRVrllnnwdvcADMVGT------------------fTDTEDP---AKFKMkywgvasflqkGNDDHWIvDTDY---DTYAVQYSCRLlnldgtcADSYSFVFSRD-------------pnglppeaqkivrqrqeelclarqyrlivhngyc

d1vpra1 ekgfeagdnklggalnakhvekygdnfkngxhkpefhedglhkpxevggkkfesgfhyllechelggknasggyggplcedpygsevqaxtekllkeadsDRTLCfnnfqdpcpqltkeqvaxckgfdygdktlklpcgplpwpaglpepgyvpktNPLHGRWITVSGGQaafikeaiksgmlgaaeankivADTDHhqtggXYLRINQFG-DVCTVDASVAK--------fARAKRTwksghyfyeplvsggnllgVWVLPEeyrKIGFF-----wexesgRCFRIER-RAFPvgpYTFXRQATEVG-------GKISFVFYVKVsndpesdpiplqsrdytalagrdnaptnlgkpyptlakdldypkkrd

d1brqa_ erdcrvssfrvkenfdkARFSGTWYAMAKKDPEG---------------lfLQDNIVAEFSVDEtGQMSATAKGRvrllnnwdvCADMVGTF------------------TDTEDP---AKFKmkywgvasflqkgndDHWIvDTDYDTYAVQYSCRLlnldgtcADSYSFVFSRDpnglppeaqkivrqrqeelclarqyrlivhngyc

d1xcaa_ -----------------PNFSGNWKIIRSENFEEllkvlgvnvmlrkiavaAASKPAVEIKQEG-DTFYIKTSTT---------VRTTEINFkvgeefeeqtvdgrpcksLVKWESenkMVCE----qkllkgegpktSWTM-ELTNDGELILTMTAD-------DVVCTRVYVRE----------------------------------

d1brqa_ erdcrvssfrvkenfdkaRFSGTWYAMAKKDpegLFLQdNIVAEFSVDETGQMSATAKGrvrllnnWDVCADMVGTFTDTEDPAKFKMKywgvasflqkgNDDHWIVDTDYDTYAVQYSCRLLNldgtcADSYSFVFSRDPNGLPPeAQKIVRQRQEELCLARQ-YRLIVHngyc

d1xkia_ ------------------DVSGTWYLKAMTV---NLES-VTPMTLTTLEGGNLEAKVTM-------SGRCQEVKAVLEKTDEPGKYTAD---------ggKHVAYIIRSHVKDHYIFYSEGEGK-----PVRGVKLVGRDPKNNLE-ALEDFEKAAGARGLSTEsILIPRQ---s

d1brqa_ erdcrvSSFRVKENFDKARFSGTWYAMAKKDPE--------GLFLqdnIVAEFSVDETGQMSATAKGRVrllnnWDVCADMVGTFTDTEDPAKFKMKYwgvasflqKGNDDHWIVDTDYDTYAVQYSCRLLNldgtCADSYSFVFSRDPNGLPpEAQKIVRQRQEELCLaRQYRLI---VHNGYC--

d1yupa1 -----iIVTQTMKDLDVQKVAGTWYSLAMAASDislldaqsAPLR--vYVEELKPTPGGDLEILLQKWE-----NGKCAQKKIIAEKTEIPAVFKIDA--------LNENKVLVLDTDYKKYLLFCMENSAE---pEQSLACQCLVRTPEVDD-EAMEKFDKALKALPM-HIRLSFnptQLEEQCrv

d1brqa_ erdcrvssfrvkenfDKARFSGTWYAmAKKDPE--glflqDNIVAEFSVDETGQMSATAKGRVrllnnwDVCADMVGTFTD-TEDPakFKMKYWGVAsfLQKGN---------------------------DDHWiVDTDydTYAVQYSCRlLNLDGTCADSYSFVFSRDpnglppeaqkivrqrqeelclarqyrlivhngyc

d2a13a1 -------ppvhpfvaPLSYLLGTWRG-QGEGEYptipsfrYGEEIRFSHSGKPVIAYTQKTWK--lesgAPXHAESGYFRPrPDGS--IEVVIAQST--GLVEVqkgtynvdeqsiklksdlvgnaskvkeISRE-FELV-dGKLSYVVRX-STTTNPLQPHLKAILDKL----------------------------------

d1brqa_ erdcrvssfrvkenfdkARFSGTWYAMAKK-DPEGL------------FLQDNIVAEFSVDEtGQMSATAKGRvrllnnwdvCADMVGTFTD----------------TEDP-----AKFKmkywgvasflqkgnDDHWIvDTDYdTYAVQYSCRLlnldgtcADSYSFVFSRDpnglppeaqkivrqrqeelclarqyrlivhngyc

d2f73a1 -----------------MSFSGKYQLQSQEnFEAFMkaiglpeeliqkGKDIKGVSEIVQNG-KHFKFTITAG---------SKVIQNEFTVgeeceletmtgekvktVVQLegdnkLVTT-----------fknIKSVT-ELNG-DIITNTMTLG-------DIVFKRISKRI----------------------------------

d1brqa_ erdcrvssfrvkenFDKARFSGTWY-amakkdpeglflqdNIVAEFSVDETGQMSATAKGRVRllnNWDVCADMVGTFTDTeDPAKFKMKYWGVASflQKGNDDHWiVDTDydTYAVQYSCRL--------lnldgtcadSYSFVFSRDPnglppeaqkivrqrqeelclaRQYRLIVhngyc

d2fr2a1 --------dlapalQALSPLLGSWAgrgagkyptirpfeyLEEVVFAHVGKPFLTYTQQTRAV--aDGKPLHSETGYLRVC-RPGCVELVLAHPSG--ITEIEVGT-YSVT-gDVIELELSTRadgsiglaptakevtalDRSYRIDGDE-------lsyslqmravgqplQDHLAAVlhrqr

d1brqa_ erdcrvssfrvkENFD--KARFS--------------------GTWYAmAKKDPEGL--flqdnIVAEFSVDETGQMSATAKGRvrllnnwdVCADMVGTFTDTedpaKFKMKYWGVA---------------------------------SFLQKGNDDH-wIVDTDYDTyavqyscrllnldgtcadsysfvfsrdpnglppeaqkivrqRQEELCLArqyrlivhngyc

d2gc9a1 -----------xTKTFktLDDFLgthfiytydngweyewyaknDHTVD-YRIHGGXVagrwvtdQKADIVXLTEGIYKISWTEP--------TGTDVALDFXPN----EKKLHGTIFFpkwveehpeitvtyqnehidlxeqsrekyatypKLVVPEFANItyXGDAGQNN-----------------------------edviseapykexPNDIRNGK-yfdqnyhrlnk

d1brqa_ erdcrvssfrvkENFDKARFSGTWYAmAKKDPE----GLFLQDnIVAEFSVDETGQMSATAKGRvrllnnwdvCADMVGTF---------------tdTEDP-----AKFKmkywgvasflqkgnDDHWIvDTDYDTYAVQYSCRLlNLDGTCADSYSFVFSRDpnglppeaqkivrqrqeelclarqyrlivhngyc

d2o62a1 ----------erPLLQINDLLGEWRG-QAVTIYrdlrPPDIYS-TTLKIQLDDAGRLXQSTSFG---------ERTITSTAtikgsivlfdqdpekqvQVLLlpdgaSATS----plkvqlrqplFLEAG-WLIQSDLRQRXIRSY-NDKGEWVSLTLVTEERV----------------------------------

d1brqa_ erdcrVSSFRVKENFDKARFS--GTWYAMAKKDP--EGLFlQDNIVAEFSVDETgQMSATAKGRVRLlnnWDVCADMVGTFTDTeDPAKFKMKYWGVA----SFLQK---GNDDHWIVDTDyDTYAVQYSCRllnldgtcaDSYSFVFSRDPNGLppeAQKIVRQRQEELCLA-RQYRLIV-HNGYC-----------

d2ofmx1 ---acTKNAIAQTGFNKDKYFngDVWYVTDYLDLepDDVPkRYCAALAAGTASG-KLKEALYHYDPK---TQDTFYDVSELQVE-SLGKYTANFKKVDkngnVKVAVtagNYYTFTVMYAD-DSSALIHTCL--hkgnkdlGDLYAVLNRNKDAA---AGDKVKSAVSAATLEfSKFISTKeNNCAYdndslkslltk

d1brqa_ erdcrvssfrVKEN--FDKARFSGTWYAMAKKD-PEGL-----FLQDnIVAEFSVDeTGQMSATAKGRVrllnnWDVCADMVGTFTDTEDPAKFKMKYwgvasflqKGNDDHWIVDTDYDTYAVQYSCRLLNLdgtcADSYSFVFSRDPnGLPPEAQKIVRQRQEELCLAR-QYRLI--VHNGyc

d2ozqa1 -------eeaSSTGrnFNVEKINGEWHTIILASdKREKiedngNFRL-FLEQIHVL-EKSLVLKFHTVR-----DEECSELSMVADKTEKAGEYSVTY--------DGFNTFTIPKTDYDNFLMAHLINEKDG---eTFQLMGLYGREP-DLSSDIKERFAQLCEEHGILReNIIDLsnANRC--

d1brqa_ erdcrVSSFRVKENFDKARFS--GTWYAMAKKDPEG--lflQDNIVAEFSVDETgQMSATAKGRVRllnnWDVCADMVGTFTDTeDPAKFKMKYWGVA----SFLQK---GNDDHWIVDTDyDTYAVQYSCRLlnldgtcADSYSFVFSRDPNGLppeAQKIVRQRQEELCLA-RQYRLIV-HNGYC-----------

d3np1a_ ---kcTKNALAQTGFNKDKYFngDVWYVTDYLDLEPddvpkRYCAALAAGTASG-KLKEALYHYDP---kTQDTFYDVSELQEE-SPGKYTANFKKVEkngnVKVDVtsgNYYTFTVMYAD-DSSALIHTCLH--kgnkdLGDLYAVLNRNKDTN---AGDKVKGAVTAASLKfSDFISTKdNKCEYdnvslkslltk

d1cbia_ pnfagtwKMRSsenfdellkalgvnamlrkvavaaaskphveirqDGDQfYIKTSTTVRT-teinFKVG---------egfeeetVDGRKCRSLPTWENE-----NKIHCTQTLlegdgpKTYWTRELAND----------ELILTFGaddVVCTRIYV----------------re

d1avgi_ -------AEGD-------------------dcsiekamgdfkpeeFFNGtWYLAHGPGVTspavcQKFTtsgskgftqiveigynKFESNVKFQCNQVDNkngeqYSFKCKSSD------NTEFEADFTFIsvsydnfalvCRSITFT---SQPKEDRYlvfertksdtdpdakeic

d1cbia_ --PNFAGTWKMRSSENFDELLKALGVNAMLRKVAVAAasKPHVEIRQD-GDQFYIKTSTTVRTTEINFKVGEGFEEETVDGRKCRSLPTWENENKIHCTQTLlegdGPKTYWTRELANDELILTFGADDVVCTRIYVRE

d1ftpa_ vkEFAGIKYKLDSQTNFEEYMKAIGVGAIERKAGLAL--SPVIELEILdGDKFKLTSKTAIKNTEFTFKLGEEFDEETLDGRKVKSTITQDGPNKLVHEQKG----DHPTIIIREFSKEQCVITIKLGDLVATRIYKAQ

d1cbia_ -PNFAGTWKMRSSENFDELLKALGVNAMLRKVAVAAasKPHVEIRQDGDQFYIKTSTTVRTTEINFKVGEGFEEET--VDGRKCRSLPTWENEnKIHCTQTLlegDGPKTYWTRELANDELILTFGADDVVCTRIYVRE-

d1ggla_ pPNLTGYYRFVSQKNMEDYLQALNISLAVRKIALLL--KPDKEIEHQGNHMTVRTLSTFRNYTVQFDVGVEFEEDLrsVDGRKCQTIVTWEEE-HLVCVQKG---EVPNRGWRHWLEGEMLYLELTARDAVCEQVFRKVh

d1cbia_ -PNFAGTWKMRSSENFDELLKALGVNAMLRKVAVAAasKPHVEIRQDGDQFYIKTSTTvRTTEINFKVGEGFEEET--VDGRKCRSLPTWENeNKIHCTQTLlegDGPKTYWTRELANDELILTFGADDVVCTRIYVRE-

d1kqxa_ pADFNGTWEMLSNDNFEDVMKALDIDFATRKIAVHL--KQTKVIVQNGDKFETKTLSTfRNYEVNFVIGEEFDEQTkgLDNRTVKTLVKWDG-DKLVCVQKG---EKENRGWKQWIEGDLLHLEIHCQDKVCHQVFKKKn

d1cbia_ -PNFAGTWKMRSSENFDELLKALGVNAMLRKVAVAAAskPHVEIRQDGDQFYIKTSTTvRTTEINFKVGEGFEEETVDGRKCRSLPTWENeNKIHCTQTLlegDGPKTYWTRELANDELILTFGADDVVCTRIYVRE

d1liba_ cDAFVGTWKLVSSENFDDYMKEVGVGFATRKVAGMAK--PNMIISVNGDLVTIRSESTfKNTEISFKLGVEFDEITADDRKVKSIITLDG-GALVQVQKW---DGKSTTIKRKRDGDKLVVECVMKGVTSTRVYERA

d1cbia_ -PNFAGTWKMRSSENFDELLKALGVNAMLRKVAVAAasKPHVEIRQDGDQFYIKTSTTvRTTEINFKVGEGFEEET--VDGRKCRSLPTWENeNKIHCTQTLlegDGPKTYWTRELANDELILTFGADDVVCTRIYVRE

d1lpja_ pADLSGTWTLLSSDNFEGYMLALGIDFATRKIAKLL--KPQKVIEQNGDSFTIHTNSSlRNYFVKFKVGEEFDEDNrgLDNRKCKSLVIWDN-DRLTCIQKG---EKKNRGWTHWIEGDKLHLEMFCEGQVCKQTFQRA

d1cbia_ --------------------------pNFAGTWKMR---------ssenfdellkalgVNAMLRKVAVAA-ASKP-HVEIRQDgdQFYIKTSttvrTTEINFkvgegfeeetvdgrkcrsLPTWENE---------NKIHCTQTllegdgpkTYWTRELA------NDELILTFGAD-------dvvcTRIYVRE--------------

d1oeja_ gkplteveqkaangvfddanvqnrtlsDWDGVWQSVypllqsgkldpvfqkkadadktKTFAEIKDYYHKgYATDiEMIGIED--GIVEFHR--nnETTSCK-----------------yDYDGYKIltyksgkkgVRYLFECK-dpeskapKYIQFSDHiiaprkSSHFHIFMGNDsqqsllnemenWPTYYPYqlsseevveemmsh

d1cbia_ -PNFAGTWKMRSSENFDELLKALGVNAMLRKVAVaaASKPHVEIRQDGDQFYIKTSTTVRTTEINFKVGEGFEEET--VDGRKCRSLPTWENeNKIHCTQTLlegDGPKTYWTRELANDELILTFGADDVVCTRIYVRE

d1opaa_ tKDQNGTWEMESNENFEGYMKALDIDFATRKIAV--RLTQTKIIVQDGDNFKTKTNSTFRNYDLDFTVGVEFDEHTkgLDGRNVKTLVTWEG-NTLVCVQKG---EKENRGWKQWVEGDKLYLELTCGDQVCRQVFKKK

d1cbia_ pNFAGTWKMRSSENFDELLKALGVNAMLRKVAVAAasKPHVEIRQDGDQFYIKTSTTVRTTEINFKVGEGFEEETVDGRKCRSLPTWeNENKIHCTQTllegdgpKTYWTRELANDELILTFGADDVVCTRIYVRE

d1p6pa_ -AFNGTWNVYAQENYENFLRTVGLPEDIIKVAKDV--NPVIEIEQNGNEFVVTSKTPKQTHSNSFTVGKESEITSMDGKKIKVTVQL-EGGKLICKSD-------KFSHIQEVNGDEMVEKITIGSSTLTRKSKRV

d1cbia_ pnfagtwkmrssenfdellkalgvnamlrkvavaaaskphveirqdgdqfyiktsttvrtteinfkvgegfeeetvdgrkcrsLPTWENE---NKIHCTQTllegDGPKTYWTRELANDELILTFGADdVVCTRIYVRE--------------------------------------------------------

d1r0ua_ -------------------------------------------------gfqsnamkqetpitlhvksvieddgnqeviefrtTGFYYVKqnkVYLSYYEE---hDLGKVKTIVKVSEGEVLVMRSGA-VKMNQRFVTGastiakykmsfgelelktstksiqsdldeekgrisiaydmhvghlhnmtityeggt

d1cbia_ pNFAGTWKMRSSENFDELLKALGVNAMLRKVAVAAAskPHVEIRQDGDQFYIKTSTTVRTTEINFKVGEGFEEETVDGRKCRSLPTWENeNKIHCTQTllegdgpKTYWTRELANDELILTFGADDVVCTRIYVRE

d1tvqa_ -AFSGTWQVYAQENYEEFLKALALPEDLIKMARDIK--PIVEIQQKGDDFVVTSKTPRQTVTNSFTLGKEADITTMDGKKLKCTVHLAN-GKLVTKSE-------KFSHEQEVKGNEMVETITFGGVTLIRRSKRV

d1cbia_ ------------------------------------------------------------------------------------------------------------------------------------------------------------pNFAGTWKMRSSEnfdellkalgvnamlrkvavaaaSK-------------------------------PHVEIRQDGDQFYIKTS-ttvrtTEINFKVGEGFEEETVDGRKCRSLPTWENENKIHCTQTLLEgDGPKTYWTRELAN----DELILTFGADDVVCTRIYVRE-----------------------------------------------

d1vpra1 ekgfeagdnklggalnakhvekygdnfkngxhkpefhedglhkpxevggkkfesgfhyllechelggknasggyggplcedpygsevqaxtekllkeadsdrtlcfnnfqdpcpqltkeqvaxckgfdygdktlklpcgplpwpaglpepgyvpktnPLHGRWITVSGG-----------------------QAafikeaiksgmlgaaeankivadtdhhqtggXYLRINQFGDVCTVDASvakfarAKRTWKSGHYFYEPLVSGGNLLGVWVLPEEYRKIGFFWEXE-SGRCFRIERRAFPvgpyTFXRQATEVGGKISFVFYVKVsndpesdpiplqsrdytalagrdnaptnlgkpyptlakdldypkkrd

d1cbia_ PNFAGTWKMRSSENFDELLKALGVNAMLRKVAVAAASKPHVEIRQDGDQFYIKTSTTVRTTEINFKVGEGFEEETVDGRKCRSLPTWENENKIHCTQTLLEGDGPKTYWTRELA-NDELILTFGADDVVCTRIYVRE

d1xcaa_ PNFSGNWKIIRSENFEELLKVLGVNVMLRKIAVAAASKPAVEIKQEGDTFYIKTSTTVRTTEINFKVGEEFEEQTVDGRPCKSLVKWESENKMVCEQKLLKGEGPKTSWTMELTnDGELILTMTADDVVCTRVYVRE

d1cbia_ ----------PNFAGTWKMrSSENFDellkalgvnaMLRKvaVAAASkPHVEIRQD-GDQFYIKTST-------TVRTTEINFKV----GEGFEEEtvdGRKCRSLPTWEN-ENKIHCTQ-TLLEGD-GPKTYWTRELANDELILTFGA------DDVVCTRIYVRE

d2a13a1 ppvhpfvaplSYLLGTWRG-QGEGEY----------PTIP--SFRYG-EEIRFSHSgKPVIAYTQKTwklesgaPXHAESGYFRPrpdgSIEVVIAqstGLVEVQKGTYNVdEQSIKLKSdLVGNASkVKEISREFELVDGKLSYVVRXstttnpLQPHLKAILDKL

d1cbia_ PNFAGTWKMRSSENFDELLKALGVNAMLRKVAVAAasKPHVEIRQDGDQFYIKTSttvrTTEINFKVGEGFEEETVDGRKCRSLPTWENENKIHCTQTllegdgpKTYWTRELANDELILTFGADDVVCTRIYVRE

d2f73a1 MSFSGKYQLQSQENFEAFMKAIGLPEELIQKGKDI--KGVSEIVQNGKHFKFTITagskVIQNEFTVGEECELETMTGEKVKTVVQLEGDNKLVTTFK-------NIKSVTELNGDIITNTMTLGDIVFKRISKRI

d1cbia_ ---------PNFAGTWKMrSSENFDellkalgvnamLRKVavAAASkPHVEIRQD-GDQFYIKTS-------ttvrTTEINFKV----GEGFEEEtvDGRKCRSLPTWEN---ENKIHCTQ----TLLE----gDGPKTYWTRELANDELILTFGA------DDVVCTRIYVRE-

d2fr2a1 dlapalqalSPLLGSWAG-RGAGKY----------pTIRP--FEYL-EEVVFAHVgKPFLTYTQQtravadgkplhSETGYLRVcrpgCVELVLAhpSGITEIEVGTYSVtgdVIELELSTradgSIGLaptakEVTALDRSYRIDGDELSYSLQMravgqpLQDHLAAVLHRQr

d1cbia_ -----------pnfAGTWKMRSsenfdellkalgvnamlrkvavaaaskpHVEIRQDGD-QFYIKTST-tvrTTEINFKVgegfeeetvdgrkcrslPTWENE----NKIHCTQTllegdgpKTYWTRELA--NDELILTFGAD--------------------------------DVVCTRIYVRE----------------------------------------

d2gc9a1 xtktfktlddflgtHFIYTYDN--------------------------gwEYEWYAKNDhTVDYRIHGgxvaGRWVTDQK-----------------ADIVXLtegiYKISWTEP------tGTDVALDFXpnEKKLHGTIFFPkwveehpeitvtyqnehidlxeqsrekyatypKLVVPEFANITyxgdagqnnedviseapykexpndirngkyfdqnyhrlnk

d1cbia_ -------PNFAGTWKMrSSENFDEllkalgvnamlRKVAVaaaSKPHVEIRQD-gdQFYIKTSttVRTTEINFKV---GEGFEEEtvdgrkcRSLPTWENENKIHCTQTLLEGDGPKTYWTRELAND-ELILTFGAD------DVVCTRIYVRE

d2o62a1 erpllqiNDLLGEWRG-QAVTIYR----------dLRPPD--iYSTTLKIQLDdagRLXQSTSfgERTITSTATIkgsIVLFDQD----pekQVQVLLLPDGASATSPLKVQLRQPLFLEAGWLIQSdLRQRXIRSYndkgewVSLTLVTEERV

d1e5pa_ ----------------FAEL-QGKWYTIViaadnlekieeggplrfyfrhidcYKNCsexeitfyvitnnqCSKTTVIGYlKGNG--TYETQF------EGNNIFQPLYIT-SDKIFFTNKNXDRAGQETNXIVVAGKGNAltpeeneilvqfahekkipvenilnilATDT--cpe

d1avgi_ aegddcsiekamgdfkPEEFfNGTWYLAH--------gpgvtspavcqkfttsGSKG-ftqiveigynkfeSNVKFQCNQvDNKNgeQYSFKCkssdntEFEADFTFISVSyDNFALVCRSITFTSQPKEDRYLVFERTKS--------------------------dTDPDakeic

d1e5pa_ fAELQgKWYTiVIAADNLE--------------kiEEGGpLRFYFrHIDCYKncSEXEITFYVItnnqCSKTTVI-------------------gYLKGNG-TYETQ--------feGNNIFQPLYItsdkiFFTNKNXDRaGQETNXIVVAGkgnaltpeeneilvqfahekkipvenilnilatdtcpe

d1cbia_ -PNFA-GTWK-MRSSENFDellkalgvnamlrkvaVAAA-SKPHV-EIRQDG--DQFYIKTSTT----VRTTEINfkvgegfeeetvdgrkcrslPTWENEnKIHCTqtllegdgpkTYWTRELAND-----ELILTFGAD-DVVCTRIYVRE--------------------------------------

d1e5pa_ -----FAELQGKWYTIVIAADnlekieeggplrfYFRHIDCYKNcsEXEITFYVITNNQCSKTTVIGYLKGNG-TYETQF-eGNNIFQPLYIT-SDKIFFTNKNXDRaGQETNXIVVAGKG-NALTpEENEILVQFAHEKKIPVENILNILATDTCPE------

d1epaa_ vkdfdISKFLGFWYEIAFASK---------eekmGAMVVELKEN--LLALTTTYYSEDHCVLEKVTATEGDGPaKFQVTRlsGKKEVVVEATDyLTYAIIDITSLVA-GAVHRTMKLYSRSlDDNG-EALYNFRKITSDHGFSETDLYILKHDLTCVKvlqsaa

d1e5pa_ --------------FAELQ-GKWYTIVIAADNLEkiEEGGplRFYFRHIDCYKncSEXEITFYVIT--NNQCSKTTVIGYLKGNG-TYETQF---------------eGNNIFQPLYITSDKIFFTNKNXDRAGQETNXIVVAGKG--NALTPEENEILVQFAHekkiPVENILNILAT-DTCPE-----

d1euoa_ mdcstnispkqgldKAKYFsGKWYVTHFLDKDPQ--VTDQ--YCSSFTPRESD--GTVKEALYHYNanKKTSFYNIGEGKLESSGlQYTAKYktvdkkkavlkeadekNSYTLTVLEADDSSALVHICLREGSKDLGDLYTVLTHQkdAEPSAKVKSAVTQAGL----QLSQFVGTKDLgCQYDDqftsl

d1e5pa_ -------FAELQGKWYTIVIAADNLEKIEEGGPLRFYFRHIDCYKNcSEXEITFYVITNNQCSKTTVIGYLKGNG-TYETQFEGNNIFQPLYI-TSDKIFFTNKNXDrAGQETNXIVVAGKGNALTPEENEILVQFAHEKKIPVENILNILAT-DTCPE--

d1ew3a_ vairnfdISKISGEWYSIFLASDVKEKIEENGSMRVFVDVIRALDN-SSLYAEYQTKVNGECTEFPMVFDKTEEDgVYSLNYDGYNVFRISEFeNDEHIILYLVNFD-KDRPFQLFEFYAREPDVSPEIKEEFVKIVQKRGIVKENIIDLTKIdRCFQLrg

d1e5pa_ -----------FAELQGKWYTIVIAAD-NLEKieeGGPLR-FYFRHIDCYKNcSEXEITFYVITNNQCSKTTVIGYLKGNG-TYETQFEGNNIFQPLYIT-SDKIFFTNKNXdraGQET--NXIVVAGKGNALTPEENEILVQFAHEKKIPVeNILNilATDTCPE

d1exsa_ vevtpimteldTQKVAGTWHTVAMAVSdVSLL-daKSSPLkAYVEGLKPTPE-GDLEILLQKRENDKCAQEVLLAKKTDIPaVFKINALDENQLFLLDTDyDSHLLLCMENS---ASPEhsLVCQSLARTLEVDDQIREKFEDALKTLSVPM-RILPaqLEEQCRV

d1e5pa_ FAELQGKWYTIVIAADNLEK---------iEEGGplRFYFRHIDCYKncSEXEITFYVItnnqCSKTTVIGY------------------LKGN---gTYETQ--feGNNIFQpLYITsdKIFFTNKNXDragQETNXIVVAGKgnaltpeeneilvqfahekkipvenilnilatdtcpe

d1ftpa_ VKEFAGIKYKLDSQTNFEEYmkaigvgaieRKAGlaLSPVIELEILD-gDKFKLTSKTA----IKNTEFTFKlgeefdeetldgrkvkstITQDgpnkLVHEQkgdhPTIIIR-EFSK--EQCVITIKLG---DLVATRIYKAQ-------------------------------------

d1e5pa_ FAELQGKWYTIVIAA--DNLE------kiEEGGPlRFYFRHIDCYKNcsEXEITFYVItnnqCSKTTVI--------------------gYLKGN---GTYE-tqfegNNIFQPlYITSDKIFFTNKNXdragqETNXIVVAGKgnaltpeeneilvqfahekkipvenilnilatdtcpe

d1ggla_ PPNLTGYYRFVSQKNmeDYLQalnislavRKIALlLKPDKEIEHQGN--HMTVRTLST----FRNYTVQfdvgvefeedlrsvdgrkcqtIVTWEeehLVCVqkgevpNRGWRH-WLEGEMLYLELTAR-----DAVCEQVFRK-----------------------------------vh

d1e5pa_ -------FAELQGKWYTIVIAADNLEKIEEGGPLRFYFRHIDCYKNcSEXEITFYVITNNQCSKTTVIGYLKGNGTYETQFEGNNIFQPLYIT-SDKIFFTNKNXdRAGQETNXIVVAGKGNALTPEENEILVQFAHEKKIPVENILNILAT-DTCPE--

d1gm6a_ vvtsnfdASKIAGEWYSILLASDAKENIEENGSMRVFVEHIRVLDN-SSLAFKFQRKVNGECTDFYAVCDKVGDGVYTVAYYGENKFRLLEVNySDYVILHLVDV-NGDKTFQLMEFYGRKPDVEPKLKDKFVEICQQYGIIKENIIDLTKIdRCFQLrg

d1e5pa_ ----------------FAELQGKWYTIVIAAdNLEKieeggPLRF-YFRHIDCYKNcSEXEITFYVIT-----NNQCSKTTVIGYLKGNG-TYETQF--------EGNNIFQPLYIT-SDKIFFTNKNX--dragqETNXIVVAGK-GNALTPEENEILVQFAHEKKIPVeNILNILATdTCPE

d1hbqa_ erdcrvssfrvkenfdKARFAGTWYAMAKKD-PEGL-----FLQDnIVAEFSVDEN-GQMSATAKGRVrllnnWDVCADMVGTFTDTEDPaKFKMKYwgvasflqKGNDDHWIIDTDyETFAVQYSCRLlnldgtcADSYSFVFARdPSGFSPEVQKIVRQRQEELCLAR-QYRLIPHNgYCNG

d1e5pa_ --------------FAELQGKWYTIVIAADNlekieeggplrfYFRHIDCYKncSEXEITFYVITNNQCSKTTVIGYLKGNG-TYETQF---EGNNIFQPLYIT-SDKIFFTNKNXdragqETNXIVVAGKGNALTPEENEILVQFAHEKKIPVENILNIlATDTCPE-----------

d1iw2a_ aspistiqpkanfdAQQFAGTWLLVAVGSAG--------raeaTTLHVAPQG--TAMAVSTFRKLDGICWQVRQLYGDTGVLgRFLLQArgaRGAVHVVVAETDyQSFAVLYLERA-----GQLSVKLYARSLPVSDSVLSGFEQRVQEAHLTEDQIFYF-PKYGFCEaadqfhvldev

d1e5pa_ ----------------FAELQGKWYTIVIAAdNLEKieeggPLRF-YFRHIDCYKNcSEXEITFYVIT-----NNQCSKTTVIGYLKGNG-TYETQF--------EGNNIFQPLYIT-SDKIFFTNKNX--dragqETNXIVVAGK-GNALTPEENEILVQFAHEKKIpVENILNIlaTDTCPe

d1jyda_ erdcrvssfrvkenfdKARFSGTWYAMAKKD-PEGL-----FLQDnIVAEFSVDET-GQMSATAKGRVrllnnWDVCADMVGTFTDTEDPaKFKMKYwgvasflqKGNDDHWIVDTDyDTYAVQYSCRLlnldgtcADSYSFVFSRdPNGLPPEAQKIVRQRQEELCL-ARQYRLI--VHNGYc

d1e5pa_ fAELQgKWYTivIAAD-----------nlekieeggplRFYFrHIDCYKncSEXEITFYVItnnqCSKTTVI---------------------gYLKGNGTYETQ-----feGNNIFQPLYitsdkIFFTNKNXDRaGQETNXIVVAGKgnaltpeeneilvqfahekkipvenilnilatdtcpe

d1kqxa_ pADFN-GTWE-mLSNDnfedvmkaldidfatrkiavhlKQTK-VIVQNG--DKFETKTLST----FRNYEVNfvigeefdeqtkgldnrtvktlVKWDGDKLVCVqkgekenRGWKQWIEG-----DLLHLEIHCQ-DKVCHQVFKKKN-------------------------------------

d1e5pa_ FAELQGKWYTIVIAA--DNLE------kiEEGGPlRFYFRHIDCYKNcsEXEITFYVItnnqCSKTTVI------------------gYLKGN---gTYET-qfegNNIFQPlYITSDKIFFTNKNXdragqETNXIVVAGKGnaltpeeneilvqfahekkipvenilnilatdtcpe

d1liba_ CDAFVGTWKLVSSENfdDYMKevgvgfatRKVAGmAKPNMIISVNGD--LVTIRSEST----FKNTEISfklgvefdeitaddrkvksIITLDggalVQVQkwdgkSTTIKR-KRDGDKLVVECVMK-----GVTSTRVYERA------------------------------------

d1e5pa_ fAELQGKWYTIVIAA-DNLEK-------ieeggplrFYFRHIDCYKNcsEXEITFYVItnnqCSKTTVI--------------------gyLKGN---gTYET-qfegNNIFQPlYITSDKIFFTNKNXdragqETNXIVVAGKGnaltpeeneilvqfahekkipvenilnilatdtcpe

d1lpja_ pADLSGTWTLLSSDNfEGYMLalgidfatrkiakllKPQKVIEQNGD--SFTIHTNSS----LRNYFVKfkvgeefdednrgldnrkckslVIWDndrlTCIQkgekkNRGWTH-WIEGDKLHLEMFCE-----GQVCKQTFQRA------------------------------------

d1e5pa_ ---------------------------FAELQGKWYTIVIAAdnlekieegGPLRF---YFRHIDCYKncSEXEITFYVIT-NNQCSKTTVIGYLK--GNGTYETQF--EGNNIFQPLYIT-SDKIFFTNKNxdrAGQETNXIVVAGKGNALTPEENEILVQFAHEKKIPVENILNILAT-dTCPE-----

d1obqa_ dkipdfvvpgkcasvdrnklwaeqtpnRNSYAGVWYQFALTN--------nPYQLIekcVRNEYSFDG--KQFVIESTGIAyDGNLLKRNGKLYPNpfGEPHLSIDYenSFAAPLVILETDySNYACLYSCIdynFGYHSDFSFIFSRSANLADQYVKKCEAAFKNINVDTTRFVKTVQGssCPYDtqktl

d1e5pa_ -------------------------FAELQGKWYTIV----------------------iaadnlekieeggpLRFYFRHIDCykncseXEITFYVItnnqCSKTTVIGYLKGN-------------gTYET---qfegnNIFQPLY-----itSDKIFFTNKNXD--ragqetnXIVVAGKGNA----LTPEENEIlvqfahekkipvenilnilatdtcpe

d1oeja_ gkplteveqkaangvfddanvqnrtLSDWDGVWQSVYpllqsgkldpvfqkkadadktktfaeikdyyhkgyaTDIEMIGIED------GIVEFHRN----NETTSCKYDYDGYkiltyksgkkgvryLFECkdpeskapKYIQFSDhiiaprkSSHFHIFMGNDSqqsllnemeNWPTYYPYQLsseeVVEEMMSH--------------------------

d1e5pa_ fAELQGKWYTIVIAA--DNLE------kiEEGGPLRFYFRHIDCYKncSEXEITFYVItnnqCSKTTVIGY--------------------LKGN---GTYE-tqfegnnIFQPlYITSDKIFFTNKNXdragqETNXIVVAGKGnaltpeeneilvqfahekkipvenilnilatdtcpe

d1opaa_ tKDQNGTWEMESNENfeGYMKaldidfatRKIAVRLTQTKIIVQDG--DNFKTKTNST----FRNYDLDFTvgvefdehtkgldgrnvktlVTWEgntLVCVqkgekenrGWKQ-WVEGDKLYLELTCG-----DQVCRQVFKKK------------------------------------

d1e5pa_ faELQGkWYTIvIAADNLE-----------kieeggplRFYFrHIDCYknCSEXEITFYVItnnqCSKTTVIGYL------------------KGNG-TYETQ-feGNNIFQPLYitsdkIFFTNKNXDRaGQETNXIVVAGkgnaltpeeneilvqfahekkipvenilnilatdtcpe

d1p6pa_ --AFNG-TWNV-YAQENYEnflrtvglpediikvakdvNPVI-EIEQN--GNEFVVTSKTP----KQTHSNSFTVgkeseitsmdgkkikvtvQLEGgKLICKsdkFSHIQEVNG-----DEMVEKITIG-SSTLTRKSKRV--------------------------------------

d1e5pa_ --------------------------FAELQGKWYTIVIAAdnlekieegGPLR---fYFRHIDCYKNcSEXEITFYVIT--NNQCSKTTVIGYLKGNG---TYETQFE--GNNIFQPLYIT--SDKIFFTNKnxdragqeTNXIVVAGKGNALTPEENEILVQFAHEKKIPVENILNIlATDTcpe

d1qwda_ hlestslykkssstpprgvtvvnnfdAKRYLGTWYEIARFD--------hRFERglekVTATYSLRDD-GGLNVINKGYNpdRGMWQQSEGKAYFTGAPtraALKVSFFgpFYGGYNVIALDreYRHALVCGP-------dRDYLWILSRTPTISDEVKQEMLAVATREGFDVSKFIWV-QQPG---

d1e5pa_ -----faelQGKWYTiVIAADN------LEKIeeggplRFYFRHIDCYKncSEXEITFYVITNNQCSKTTVIGYlkgnGTYET-------qfEGNN------------------------IFQPlYITSDKIFFTNKNXDRAgQETNXIVVAGKGnaltpeeneilvqfahekkipvenilnilatdtcpe

d1r0ua_ gfqsnamkqETPITL-HVKSVIeddgnqEVIE------FRTTGFYYVKQ--NKVYLSYYEEHDLGKVKTIVKVS----EGEVLvmrsgavkmNQRFvtgastiakykmsfgelelktstkSIQS-DLDEEKGRISIAYDMHVgHLHNMTITYEGG-----------------------------------t

d1e5pa_ faELQGKWYTIVIAA--DNLE------kiEEGGPlRFYFRHIDCYKncSEXEITFYVItnnqCSKTTVIGY------------------LKGN---GTYEtqfegNNIFQPlYITSDKIFFTNKNXdragqETNXIVVAGKGnaltpeeneilvqfahekkipvenilnilatdtcpe

d1tvqa_ --AFSGTWQVYAQENyeEFLKalalpedlIKMARdIKPIVEIQQKG--DDFVVTSKTP----RQTVTNSFTlgkeadittmdgkklkctVHLAngkLVTK---seKFSHEQ-EVKGNEMVETITFG-----GVTLIRRSKRV------------------------------------

d1e5pa_ -----------------------------------------------------------------------------------------------------------------------------------------------------------fAELQgKWYTiVIAAdnlekIEEG------------------------gpLRFYFRHIDCYKncSEXEITFYVITnnqcSKTTVI--------------------GYLKGNGTYETQF-------eGNNIFQPLYITsdKIFFTNKNXDrAGQETNXIVVAGK---------gnaltpeeneilvqfahekkipvenilnilatdtcpe

d1vpra1 ekgfeagdnklggalnakhvekygdnfkngxhkpefhedglhkpxevggkkfesgfhyllechelggknasggyggplcedpygsevqaxtekllkeadsdrtlcfnnfqdpcpqltkeqvaxckgfdygdktlklpcgplpwpaglpepgyvpktNPLH-GRWI-TVSG----gQAAFikeaiksgmlgaaeankivadtdhhqTGGXYLRINQFG--DVCTVDASVAK---fARAKRTwksghyfyeplvsggnllgvWVLPEEYRKIGFFwexesgrcFRIERRAFPVG-pYTFXRQATEV-GGKISFVFYVKVSndpesdpiplqsrdytalagrdnaptnlgkpyptlakdldypkkrd

d1e5pa_ fAELQGkWYTIVIAADNLE-------------kiEEGGplRFYFrHIDCYKncSEXEITFYVITnnqcSKTTVI------------------gyLKGN---GTYETQ------feGNNIFQPLYITsdKIFFTNKNXDragQETNXIVVAGKgnaltpeeneilvqfahekkipvenilnilatdtcpe

d1xcaa_ -PNFSG-NWKIIRSENFEEllkvlgvnvmlrkiaVAAA-sKPAV-EIKQEG--DTFYIKTSTTV----RTTEINfkvgeefeeqtvdgrpckslVKWEsenKMVCEQkllkgegpKTSWTMELTND--GELILTMTAD---DVVCTRVYVRE-------------------------------------

d1e5pa_ faELQGKWYTIVIAAdnlekieegGPLRFYFRHIDCYKNcSEXEITFYVitNNQCSKTTVIGYLKGNG-TYETQfegNNIFQPLYI-TSDKIFFTNKNXdraGQETNXIVVAGKG--NALTpeENEILVQFAHEKKIPVENILNiLATDtcpe

d1xkia_ --DVSGTWYLKAMTV---------NLESVTPMTLTTLEG-GNLEAKVTM--SGRCQEVKAVLEKTDEPgKYTAD-ggKHVAYIIRShVKDHYIFYSEGE---GKPVRGVKLVGRDpkNNLE--ALEDFEKAAGARGLSTESILI-PRQS----

d1e5pa_ -----------FAELQGKWYTIVIAADNLEKIEE-GGPLRFYFRHIDCYKNcSEXEITFYVITNNQCSKTTVIGYLKGNG-TYETQFEGNNIFQPLYIT-SDKIFFTNKNXdraGQET--NXIVVAGK-GNALTpEENEILVQFAHEKKIPveNILNI----LATDTCpe

d1yupa1 iivtqtmkdldVQKVAGTWYSLAMAASDISLLDAqSAPLRVYVEELKPTPG-GDLEILLQKWENGKCAQKKIIAEKTEIPaVFKIDALNENKVLVLDTDyKKYLLFCMENS---AEPEqsLACQCLVRtPEVDD-EAMEKFDKALKALPMH--IRLSFnptqLEEQCR-v

d1e5pa_ ---------FAELQGKWytiviaadnlekieeggplrfyfRHIDCYKNcSEXEITFYVI---tNNQCSKTTVIGYLKGNGTYETQF------EGNNIFQPLYitsdkIFFTNKN-XDRA---gQETNXIVVAGKGnaltpeeneilvqfahekKIPVeNILNILatdtcpe

d2a13a1 ppvhpfvapLSYLLGTW----rgqgegeyptipsfrygeeIRFSHSGK-PVIAYTQKTWklesGAPXHAESGYFRPRPDGSIEVVIaqstglVEVQKGTYNV---deQSIKLKSdLVGNaskvKEISREFELVDG-----klsyvvrxstttnPLQP-HLKAIL----dkl

d1e5pa_ fAELQGKWYTIVIAAdnLEKI---------EEGGplrfYFRHIDCYKNcsEXEITFYVItnnqCSKTTVI------------------gYLKGN----GTYETQfegNNIFQPlYITSDKIFFTNKNXdragqETNXIVVAGKGnaltpeeneilvqfahekkipvenilnilatdtcpe

d2f73a1 -MSFSGKYQLQSQEN-fEAFMkaiglpeelIQKGkdikGVSEIVQNGK--HFKFTITAG----SKVIQNEftvgeeceletmtgekvktVVQLEgdnkLVTTFK---NIKSVT-ELNGDIITNTMTLG-----DIVFKRISKRI------------------------------------

d1e5pa_ --------FAELQGKWYTiVIAADN---LEKIeeggplRFYFRHIDCYKncsEXEITFYVI--tNNQC-SKTTVIG--------------------ylKGNG---------TYET------------qfeGNNIFQPlYITSDKIFFTNKNXDRAGQ-ETNXIVVAGKGNaltpeeneilvqfahekkipvenilnilatdtcpe

d2fr2a1 dlapalqaLSPLLGSWAG-RGAGKYptiRPFE------YLEEVVFAHVG-kpFLTYTQQTRavaDGKPlHSETGYLrvcrpgcvelvlahpsgiteieVGTYsvtgdvielELSTradgsiglaptakevTALDRSY-RIDGDELSYSLQMRAVGQPlQDHLAAVLHRQR-----------------------------------

d1e5pa_ -------FAELQGKWYTIVIAAdnlekieeggpLRFYFrHIDCykncsEXEITFYVI-tnnqCSKTT-VIGYLKGN----gTYETQF--EGNNIFQPLYItsdkIFFTNKNXDRA-------------------------------GQETNXIVVAGKGnaltpeeneilvqfahekkipvenilnilATDT--------------cpe

d2gc9a1 xtktfktLDDFLGTHFIYTYDN----------gWEYEW-YAKN-----DHTVDYRIHggxvaGRWVTdQKADIVXLtegiyKISWTEptGTDVALDFXPN---eKKLHGTIFFPKwveehpeitvtyqnehidlxeqsrekyatypKLVVPEFANITYX------------gdagqnnedviseapykEXPNdirngkyfdqnyhrlnk

d1e5pa_ ------FAELQGKWYtiviaadnlekieeggplrfyFRHIDCYKncSEXEITFYVItnnqCSKTTVIGYLKgNGTYET-QFEG-NNIFQPLYitsdkIFFTNKNXdragqetnxivvagkgnaltpeeneilvqfahekkipvenilnilatdtcpe

d2o62a1 erpllqINDLLGEWR---gqavtiyrdlrppdiystTLKIQLDD-aGRLXQSTSFG----ERTITSTATIK-GSIVLFdQDPEkQVQVLLLP----dGASATSPL-------kvqlrqplfleagwliqsdlrqrxirsyndkgewvsltlvteerv

d1e5pa_ -------------FAELQ--GKWYTIVIAADNLEKIEEGgplRFYFRHIDCYKNcSEXEITFYVITNNQCSKTTVIGYLKGNG-TYETQF---------------eGNNIFQPLYITSDKIFFTNKNXDRAGQETNXIVVAGKG--NALTPEENEILVQFAHekkiPVENILNILAT-DTCPE---------

d2ofmx1 actknaiaqtgfnKDKYFngDVWYVTDYLDLEPDDVPKR---YCAALAAGTASG-KLKEALYHYDPKTQDTFYDVSELQVESLgKYTANFkkvdkngnvkvavtagNYYTFTVMYADDSSALIHTCLHKGNKDLGDLYAVLNRNkdAAAGDKVKSAVSAATL----EFSKFISTKENnCAYDNdslkslltk

d1e5pa_ -----------FAELQGKWYTIVIAADNLEKIEEGGPLRFYFRHIDCYKncSEXEITFYVITNNQCSKTTVIGYLKGN-GTYETQFEGNNIFQPLYIT-SDKIFFTNKNXDrAGQETNXIVVAGKGNALTPEENEILVQFAHEKKIPVENILNILATDTCpe

d2ozqa1 eeasstgrnfnVEKINGEWHTIILASDKREKIEDNGNFRLFLEQIHVLE--KSLVLKFHTVRDEECSELSMVADKTEKaGEYSVTYDGFNTFTIPKTDyDNFLMAHLINEK-DGETFQLMGLYGREPDLSSDIKERFAQLCEEHGILRENIIDLSNANRC--

d1e5pa_ -------------FAELQ--GKWYTIVIAADNLEkiEEGGplRFYFRHIDCYKNcSEXEITFYVITNNQCSKTTVIGYLKGNG-TYETQF---------------eGNNIFQPLYITSDKIFFTNKNXDRAGQETNXIVVAGKG--NALTPEENEILVQFAHekkiPVENILNILAT-DTCPE---------

d3np1a_ kctknalaqtgfnKDKYFngDVWYVTDYLDLEPD-dVPKR--YCAALAAGTASG-KLKEALYHYDPKTQDTFYDVSELQEESPgKYTANFkkvekngnvkvdvtsgNYYTFTVMYADDSSALIHTCLHKGNKDLGDLYAVLNRNkdTNAGDKVKGAVTAASL----KFSDFISTKDNkCEYDNvslkslltk

d1epaa_ -----------VKDFDISKF-LGFWYeIAFA-skeekmgamvvELKEN---llaltttyysedHCVLEKVTATE-GDGPaKFQVTR-----LSGKKEVVVEATDYLTYAIIDITSLVA-GAVHRTMKLYSRSLDdngealynfrkitsdhgfsetdlyilKHDLtCVKVLQsaa

d1avgi_ aegddcsiekaMGDFKPEEFfNGTWY-LAHGpgvtspavcqkfTTSGSkgftqiveigynkfeSNVKFQCNQVDnKNGE-QYSFKCkssdnTEFEADFTFISVSYDNFALVCRSITFTsQPKEDRYLVFERTKS--------------------------DTDP-DAKEIC---

d1epaa_ vkdfdiskFLGFWYEIAFASK-----------------------eEKMGAMVVELkenlLALTTTYYSEDHCVLEKVT------------------ATEGDgPAKFQVTRL-------SGKKEVVVEAtdyltyAIIDITSLVAGAVHRTMKLYSrslddngealynfrkitsdhgfsetdlyilkhdltcvkvlqsaa

d1cbia_ --------PNFAGTWKMRSSEnfdellkalgvnamlrkvavaaasKPHVEIRQDG----DQFYIKTSTTVRTTEINFKvgegfeeetvdgrkcrslPTWEN-ENKIHCTQTllegdgpKTYWTRELAN------DELILTFGADDVVCTRIYVRE--------------------------------------------

d1epaa_ ---------VKDFDISKF-LGFWYEIAFASK-----EEKMGAMVVELKENLLALTTTYYS--EDHCVLEKVTATEGDGPAKFQVTR--------------LSGKKEVVVEATdYLTYAIIDITSLVAGAV-HRTMKLYSRSL-DDNGE-ALYNFRKITSdhgfSETDLYILK-HDLTCvkvLQSAA--

d1euoa_ mdcstnispKQGLDKAKYfSGKWYVTHFLDKdpqvtDQYCSSFTPRESDGTVKEALYHYNanKKTSFYNIGEGKLESSGLQYTAKYktvdkkkavlkeadEKNSYTLTVLEA-DDSSALVHICLREGSKDlGDLYTVLTHQKdAEPSAkVKSAVTQAGL----QLSQFVGTKdLGCQY---DDQFTsl

d1epaa_ --VKDFDISKFLGFWYEIAFASK---------eekmGAMVVELKEN-LLALTTTYYSEDHCVLEKVTATEGDGPAKFQVTRLsGKKEVVVEATDYLTYAIIDITSLVAGAVHRTMKLYSRSLDDNGEALYNFRKITSDHGFSETDLYIL-KHDLtCVKVLQsaa

d1ew3a_ vaIRNFDISKISGEWYSIFLASDvkekieengsmrvFVDVIRALDNsSLYAEYQTKVNGECTEFPMVFDKTEEDGVYSLNYD-GYNVFRISEFENDEHIILYLVNFDKDRPFQLFEFYAREPDVSPEIKEEFVKIVQKRGIVKENIIDLtKIDR-CFQLRG---

d1epaa_ ------VKDFDISKFLGFWYEIAFASK----------eekmGAMVVELKEN-LLALTTTYYSEDHCVLEKVTATEGDGPAKFQVTRlsGKKEVVVEATDyLTYAIIDITSLVagaVHRTMKLYSRSLDDNGEALYNFRKITSDHGFSEtDLYILKHDLTCVKvlqsaa

d1exsa_ vevtpiMTELDTQKVAGTWHTVAMAVSdvslldakssplkaYVEGLKPTPEgDLEILLQKRENDKCAQEVLLAKKTDIPAVFKINA-lDENQLFLLDTDyDSHLLLCMENSAspeHSLVCQSLARTLEVDDQIREKFEDALKTLSVPM-RILPAQLEEQCRV------

d1epaa_ vkdfdISKFLG-FWYEIAFAS-----------------keEKMGAMVVELKE-NLLALTTTYYsedhCVLEKVTA------------------TEGDGP---AKFQVTrLSGKKEVVVEatdYLTYAIIDITSLvagaVHRTMKLYSRSlddngealynfrkitsdhgfsetdlyilkhdltcvkvlqsaa

d1ftpa_ -----VKEFAGiKYKLDSQTNfeeymkaigvgaierkaglALSPVIELEILDgDKFKLTSKTA----IKNTEFTFklgeefdeetldgrkvksTITQDGpnkLVHEQK-GDHPTIIIRE--fSKEQCVITIKLG----DLVATRIYKAQ------------------------------------------

d1epaa_ vkdfdISKFLGFWYEIAFAS-----------------keEKMGAMVVELKENLLALTTTYYsedhCVLEKVT--------------------aTEGDG--PAKFQVTRLSGKKEVVVeATDYlTYAIIDITSLvagaVHRTMKLYSRSlddngealynfrkitsdhgfsetdlyilkhdltcvkvlqsaa

d1ggla_ -----PPNLTGYYRFVSQKNmedylqalnislavrkialLLKPDKEIEHQGNHMTVRTLST----FRNYTVQfdvgvefeedlrsvdgrkcqtIVTWEeeHLVCVQKGEVPNRGWRH-WLEG-EMLYLELTAR----DAVCEQVFRKV-----------------------------------------h

d1epaa_ --VKDFDISKFLGFWYEIAFASK---------eekmGAMVVELKEN-LLALTTTYYSEDHCVLEKVTATEGdGPAKFQVTRLsGKKEVVVEATDYLTYAIIDITSLVAGAVHRTMKLYSRSLDDNGEALYNFRKITSDHGFSETDLYIL-KHDLtCVKVLQsaa

d1gm6a_ vvTSNFDASKIAGEWYSILLASDakenieengsmrvFVEHIRVLDNsSLAFKFQRKVNGECTDFYAVCDKV-GDGVYTVAYY-GENKFRLLEVNYSDYVILHLVDVNGDKTFQLMEFYGRKPDVEPKLKDKFVEICQQYGIIKENIIDLtKIDR-CFQLRG---

d1epaa_ -----------VKDFDISKFLGFWYEIAFASK----eEKMGAMVVELK-ENLLALTTTYYSE-----DHCVLEKVTATEGDGPAKFQVTR-------lsGKKEVVVEATDYLTYAIIDITSL---vagaVHRTMKLYSRSLDDNG-EALYNFRKITSDHGFSEtDLYILKHDLTCVKvlqsaa

d1hbqa_ erdcrvssfrvKENFDKARFAGTWYAMAKKDPeglflQDNIVAEFSVDeNGQMSATAKGRVRllnnwDVCADMVGTFTDTEDPAKFKMKYwgvasflqkGNDDHWIIDTDYETFAVQYSCRLlnldgtcADSYSFVFARDPSGFSpEVQKIVRQRQEELCLAR-QYRLIPHNGYCNG------

d1epaa_ ---------VKDFDISKFLGFWYEIAFASK-EEKMGAMVVELKENLLALTTTYYSEDHCVLEKVTATEGDGPAKFQVTRLS--GKKEVVVEATDYLTYAIIDITSLvagaVHRTMKLYSRSLDDNGEALYNFRKITSDHGFSETDLYILKHDLTCvkvLQSA-------a

d1iw2a_ aspistiqpKANFDAQQFAGTWLLVAVGSAgRAEATTLHVAPQGTAMAVSTFRKLDGICWQVRQLYGDTGVLGRFLLQARGarGAVHVVVAETDYQSFAVLYLERA----GQLSVKLYARSLPVSDSVLSGFEQRVQEAHLTEDQIFYFPKYGFC---EAADqfhvldev

d1epaa_ -----------VKDFDISKFLGFWYEIAFASK----eEKMGAMVVELK-ENLLALTTTYYSE-----DHCVLEKVTATEGDGPAKFQVT-------rlsGKKEVVVEATDYLTYAIIDITSL---vagaVHRTMKLYSRSLDDNG-EALYNFRKITSDHGFSEtDLYILKHDLTCvkvlqsaa

d1jyda_ erdcrvssfrvKENFDKARFSGTWYAMAKKDPeglflQDNIVAEFSVDeTGQMSATAKGRVRllnnwDVCADMVGTFTDTEDPAKFKMKywgvasflqkGNDDHWIVDTDYDTYAVQYSCRLlnldgtcADSYSFVFSRDPNGLPpEAQKIVRQRQEELCLAR-QYRLIVHNGYC--------

d1epaa_ vkdfdiSKFLGFWYEIAFAS-----------------keEKMGAMVVELKENLLALTTTYYsedhCVLEKVTA--------------------TEGDGP--AKFQVTRLSGKKEVVVeATDYlTYAIIDITSLvagaVHRTMKLYSRSlddngealynfrkitsdhgfsetdlyilkhdltcvkvlqsaa

d1kqxa_ -----pADFNGTWEMLSNDNfedvmkaldidfatrkiavHLKQTKVIVQNGDKFETKTLST----FRNYEVNFvigeefdeqtkgldnrtvktLVKWDGdkLVCVQKGEKENRGWKQ-WIEG-DLLHLEIHCQ----DKVCHQVFKKK-----------------------------------------n

d1epaa_ vkdfdISKFLGFWYEIAFAS-----------------keEKMGAMVVELKENLLALTTTYYsedhCVLEKVT------------------aTEGDGP--AKFQVTRLSGKKEVVVEATDylTYAIIDITSLvagaVHRTMKLYSRSlddngealynfrkitsdhgfsetdlyilkhdltcvkvlqsaa

d1liba_ -----CDAFVGTWKLVSSENfddymkevgvgfatrkvagMAKPNMIISVNGDLVTIRSEST----FKNTEISfklgvefdeitaddrkvksIITLDGgaLVQVQKWDGKSTTIKRKRDG--DKLVVECVMK----GVTSTRVYERA------------------------------------------

d1epaa_ vkdfdiSKFLGFWYEIAFAS-----------------keEKMGAMVVELKENLLALTTTYYsedhCVLEKVTA--------------------TEGDGP--AKFQVTRLSGKKEVVVeATDYlTYAIIDITSLvagaVHRTMKLYSRSlddngealynfrkitsdhgfsetdlyilkhdltcvkvlqsaa

d1lpja_ -----pADLSGTWTLLSSDNfegymlalgidfatrkiakLLKPQKVIEQNGDSFTIHTNSS----LRNYFVKFkvgeefdednrgldnrkcksLVIWDNdrLTCIQKGEKKNRGWTH-WIEG-DKLHLEMFCE----GQVCKQTFQRA------------------------------------------

d1epaa_ --------------------VKDF--DISKFLGFWYEIAFAS----keEKMGAMVVELKENLLALTTTYYSED-HCVLEKVTATEG--DGPAkFQVT-rlsGKKEVVVEATDYLTYAIIDITSL-vaGAVHRTMKLYSRSLDDNGEALYNFRKITSDHGFSETDLYILKH--DLTCvKVLQSAa

d1obqa_ dkipdfvvpgkcasvdrnklWAEQtpNRNSYAGVWYQFALTNnpyqliEKCVRNEYSFDGKQFVIESTGIAYDgNLLKRNGKLYPNpfGEPH-LSIDyensFAAPLVILETDYSNYACLYSCIDynfGYHSDFSFIFSRSANLADQYVKKCEAAFKNINVDTTRFVKTVQgsSCPY-DTQKTL-

d1epaa_ --------------------vKDFDISKFLGFWYEIA-------------------------------faskeekmGAMVVELkenlLALTTTYYsedhCVLEKVTATEGDGP------------AKFQVTRLS--gkKEVVVeATDY-----LTYAIIDITSL---vagavhrTMKLYSR-SLDD--NGEALYnfrkitsdhgfsetdlyilkhdltcvkvlqsaa

d1oeja_ gkplteveqkaangvfddanvQNRTLSDWDGVWQSVYpllqsgkldpvfqkkadadktktfaeikdyyhkgyatdiEMIGIED----GIVEFHRN----NETTSCKYDYDGYKiltyksgkkgvrYLFECKDPEskapKYIQF-SDHIiaprkSSHFHIFMGNDsqqsllnemeNWPTYYPyQLSSeeVVEEMM-------------------------------sh

d1epaa_ vkdfdiSKFLGFWYEIAFAS-----------------keEKMGAMVVELKENLLALTTTYYsedhCVLEKVTA--------------------TEGDGP--AKFQvTRLSG-KKEVVVeATDYlTYAIIDITSLvagaVHRTMKLYSRSlddngealynfrkitsdhgfsetdlyilkhdltcvkvlqsaa

d1opaa_ -----tKDQNGTWEMESNENfegymkaldidfatrkiavRLTQTKIIVQDGDNFKTKTNST----FRNYDLDFtvgvefdehtkgldgrnvktLVTWEGntLVCV-QKGEKeNRGWKQ-WVEG-DKLYLELTCG----DQVCRQVFKKK------------------------------------------

d1epaa_ vkdfdiskflgFWYEIAFASK---------------------eekMGAMVVELkenlLALTTTYYSEDHCVLEKVT------------------ATEGdgPAKFQVTRLSGKKEVVVEAtdyltyAIIDITSLVAGAVHRTMKLYSrslddngealynfrkitsdhgfsetdlyilkhdltcvkvlqsaa

d1p6pa_ ---------afNGTWNVYAQEnyenflrtvglpediikvakdvnpVIEIEQNG----NEFVVTSKTPKQTHSNSFTvgkeseitsmdgkkikvtVQLE--GGKLICKSDKFSHIQEVNG------DEMVEKITIGSSTLTRKSKRV--------------------------------------------

d1epaa_ ---------------------VKDFDISKFLGFWYEIAFAS----kEEKMGAMVVELKE-NLLALTTTYYSE--DHCVLEKVTATEGDG--PAKFQVT-RLSGKKEVVVEATDYL-TYAIIDITslvagavhRTMKLYSRSLDDNGEALYNFRKITSDHGFSETDLYILKHDltcvkvlqsaa

d1qwda_ hlestslykkssstpprgvtvVNNFDAKRYLGTWYEIARFDhrferGLEKVTATYSLRDdGGLNVINKGYNPdrGMWQQSEGKAYFTGAptRAALKVSfFGPFYGGYNVIALDREyRHALVCGP------drDYLWILSRTPTISDEVKQEMLAVATREGFDVSKFIWVQQP----------g

d1epaa_ vkdfdiskfLGFWYEIAFASK----------eekMGAMVVELKenLLALTTTYYSE--DHCVLEKVTAtegdgpaKFQV------------------------------trLSGKK-EVVVEatdylTYAIIDITSLVA-gAVHRTMKLYSRSLddngealynfrkitsdhgfsetdlyilkhdltcvkvlqsaa

d1r0ua_ -gfqsnamkQETPITLHVKSVieddgnqeviefrTTGFYYVKQ--NKVYLSYYEEHdlGKVKTIVKVS-------EGEVlvmrsgavkmnqrfvtgastiakykmsfgeleLKTSTkSIQSD--ldeEKGRISIAYDMHvgHLHNMTITYEGGT-----------------------------------------

d1epaa_ vkdfdiskflgFWYEIAFASK---------------------eekMGAMVVELkenlLALTTTYYSEDHCVLEKVT------------------ATEGDgpAKFQVTRLSGKKEVVVEAtdyltyAIIDITSLVAGAVHRTMKLYSrslddngealynfrkitsdhgfsetdlyilkhdltcvkvlqsaa

d1tvqa_ ---------afSGTWQVYAQEnyeeflkalalpedlikmardikpIVEIQQKG----DDFVVTSKTPRQTVTNSFTlgkeadittmdgkklkctVHLAN--GKLVTKSEKFSHEQEVKG------NEMVETITFGGVTLIRRSKRV--------------------------------------------

d1epaa_ ----------------------------------------------------------------------------------------------------------------------------------------------------vkdfdISKFlgFWYEIAFAS-------------------------------keekMGAMVVELkenlLALTTTYYSE-dHCVLEKVT------------------ATEGdGPAKFQVTRL------SGKKEVVVEATDyltYAIIDITSLVAGAVHRTMKLYSRSlddngealynfrkitsdhgfsetdlyilkhdltcvkvlQSAA--------------------------------------

d1vpra1 ekgfeagdnklggalnakhvekygdnfkngxhkpefhedglhkpxevggkkfesgfhyllechelggknasggyggplcedpygsevqaxtekllkeadsdrtlcfnnfqdpcpqltkeqvaxckgfdygdktlklpcgplpwpaglpepgyvPKTNplHGRWITVSGgqaafikeaiksgmlgaaeankivadtdhhqtggxYLRINQFG----DVCTVDASVAkfARAKRTWKsghyfyeplvsggnllgvWVLP-EEYRKIGFFWexesgrCFRIERRAFPVG--pYTFXRQATEVGGKISFVFYVKVSN-----------------------------------dpeSDPIplqsrdytalagrdnaptnlgkpyptlakdldypkkrd

d1epaa_ vkdfdiSKFLGFWYEIAFAS-----------------kEEKM--GAMVVELKENLLALTTTYYsedhCVLEKVTA-----------------TEGDGP----AKFQ---vtrLSGKKEVVVeATDYLTYAIIDITSLvagaVHRTMKLYSRSlddngealynfrkitsdhgfsetdlyilkhdltcvkvlqsaa

d1xcaa_ ------PNFSGNWKIIRSENfeellkvlgvnvmlrkiaVAAAskPAVEIKQEGDTFYIKTSTT----VRTTEINFkvgeefeeqtvdgrpckSLVKWEsenkMVCEqkllkgEGPKTSWTM-ELTNDGELILTMTAD----DVVCTRVYVRE------------------------------------------

d1epaa_ vkdfdisKFLGFWYEIAFASkeekMGAMVVELKEN-LLALTTTyysEDHCVLEKVTATEGDGPAKFQVTRlsgKKEVVVEATDYLTYAIIDITSLvaGAVHRTMKLYSRS-LDDNGeALYNFRKITSDHGFSETDLYILKHDltcvkvlqsaa

d1xkia_ -------DVSGTWYLKAMTVnlesVTPMTLTTLEGgNLEAKVT--mSGRCQEVKAVLEKTDEPGKYTADG--gKHVAYIIRSHVKDHYIFYSEGE--GKPVRGVKLVGRDpKNNLE-ALEDFEKAAGARGLSTESILIPRQS-----------

d1epaa_ ------VKDFDISKFLGFWYEIAFASK----------eekmGAMVVELKEN-LLALTTTYYSEDHCVLEKVTATEGDGPAKFQVTRlsGKKEVVVEATDYLTYAIIDITSLVagaVHRTMKLYSRSLDDNGEALYNFRKITSDHGFSetDLYILKHD--LTCVKvlqsaa

d1yupa1 iivtqtMKDLDVQKVAGTWYSLAMAASdislldaqsaplrvYVEELKPTPGgDLEILLQKWENGKCAQKKIIAEKTEIPAVFKIDA-lNENKVLVLDTDYKKYLLFCMENSAepeQSLACQCLVRTPEVDDEAMEKFDKALKALPMH--IRLSFNPTqlEEQCR-----v

d1epaa_ ----vkdfDISKFLGFWYEiAFASK------eeKMGAMVVELK-ENLLALTTTYYS---EDHCVLEKVTAT--------------------eGDGP------AKFQ-vTRLS----GKKEVVVEATDylTYAIIDITSLVA-GAVH-RTMKLYSRSlddngealynfrkitsdhgfsetdlyilkhdltcvkvlqsaa

d2a13a1 ppvhpfvaPLSYLLGTWRG-QGEGEyptipsfrYGEEIRFSHSgKPVIAYTQKTWKlesGAPXHAESGYFRprpdgsievviaqstglvevqKGTYnvdeqsIKLKsdLVGNaskvKEISREFELVD--GKLSYVVRXSTTtNPLQpHLKAILDKL------------------------------------------

d1epaa_ vkdfdiskFLGFWYEIAFASK---------------------eeKMGAMVVELkenlLALTTTYYSEDHCVLEKVT------------------ATEGdGPAKFQVTRLSGKKEVVVEAtdyltyAIIDITSLVAGAVHRTMKLYSrslddngealynfrkitsdhgfsetdlyilkhdltcvkvlqsaa

d2f73a1 --------MSFSGKYQLQSQEnfeafmkaiglpeeliqkgkdikGVSEIVQNG----KHFKFTITAGSKVIQNEFTvgeeceletmtgekvktvVQLE-GDNKLVTTFKNIKSVTELNG------DIITNTMTLGDIVFKRISKRI--------------------------------------------

d1epaa_ ---vkdfDISKFLGFWYEiAFASK------eekMGAMVVELK-ENLLALTTTYYS---EDHCVLEKVTAT--------------------eGDGP--------AKFQ----vTRLS-------GKKEVVVeATDYlTYAIIDITSLVA-GAVH-RTMKLYSRSLddngealynfrkitsdhgfsetdlyilkhdltcvkvlqsaa

d2fr2a1 dlapalqALSPLLGSWAG-RGAGKyptirpfeyLEEVVFAHVgKPFLTYTQQTRAvadGKPLHSETGYLRvcrpgcvelvlahpsgiteieVGTYsvtgdvieLELStradgSIGLaptakevTALDRSY-RIDG-DELSYSLQMRAVgQPLQdHLAAVLHRQR-----------------------------------------

d1epaa_ --vkdFDISKF-LGFWYEiAFASkeekmGAMVVELkenLLALTTTYYS-edhCVLEK-VTATEGDGP---AKFQVTR-LSGKKEVVVEATDyltyAIIDITSLVA--------------------------------GAVHRTMKLYSRslddngealynfrkitsdhgfsetdlyilKHDLT--------cvkvlqsaa

d2gc9a1 xtktfKTLDDFlGTHFIY-TYDN--gweYEWYAKN---DHTVDYRIHGgxvaGRWVTdQKADIVXLTegiYKISWTEpTGTDVALDFXPNE----KKLHGTIFFPkwveehpeitvtyqnehidlxeqsrekyatypKLVVPEFANITY------------xgdagqnnedviseapyKEXPNdirngkyfdqnyhrlnk

d1epaa_ -vKDFDISKFLGFWYEiAFASK-------eekMGAMVVELKE-NLLALTTTYYsedhCVLEKVTA----------------TEGDGP----AKFQ---vtrLSGKKEVVVeATDYLTYAIIDITSLV--AGAVHRTMKLYSRSlddngealynfrkitsdhgfsetdlyilkhdltcvkvlqsaa

d2o62a1 erPLLQINDLLGEWRG-QAVTIyrdlrppdiySTTLKIQLDDaGRLXQSTSFG----ERTITSTAtikgsivlfdqdpekqVQVLLLpdgaSATSplkvqlRQPLFLEAG-WLIQSDLRQRXIRSYNdkGEWVSLTLVTEERV------------------------------------------

d1epaa_ --------VKDFDISKFL--GFWYEIAFASK------EEKMGAMVVELKENLLALTTTYYS--EDHCVLEKVTATEGdGPAKFQVTR--------------LSGKKEVVVEATdYLTYAIIDITSLVAGAV-HRTMKLYSRS-LDDNGE-ALYNFRKITSdhgfSETDLYILK-HDLTCvkvLQSAA------

d2ofmx1 actknaiaQTGFNKDKYFngDVWYVTDYLDLepddvpKRYCAALAAGTASGKLKEALYHYDpkTQDTFYDVSELQVE-SLGKYTANFkkvdkngnvkvavtAGNYYTFTVMYA-DDSSALIHTCLHKGNKDlGDLYAVLNRNkDAAAGDkVKSAVSAATL----EFSKFISTKeNNCAY---DNDSLkslltk

d1epaa_ ------VKDFDISKFLGFWYEIAFASK---------eekmGAMVVELKENLLALTTTYYSEDHCVLEKVTATEGDGPAKFQVTRLsGKKEVVVEATDYLTYAIIDITSLVAGAVHRTMKLYSRSLDDNGEALYNFRKITSDHGFSETDLYIL-KHDLTcvkvlqsaa

d2ozqa1 eeasstGRNFNVEKINGEWHTIILASDkrekiedngnfrlFLEQIHVLEKSLVLKFHTVRDEECSELSMVADKTEKAGEYSVTYD-GFNTFTIPKTDYDNFLMAHLINEKDGETFQLMGLYGREPDLSSDIKERFAQLCEEHGILRENIIDLsNANRC---------

d1epaa_ --------VKDFDISKFL--GFWYEIAFASK------EEKMGAMVVELKENLLALTTTYYS--EDHCVLEKVTATEGdGPAKFQVTR--------------LSGKKEVVVEATDyLTYAIIDITSLV-AGAVHRTMKLYSRS-LDDNGEALYNFRKITSDhgfSETDLYILK-HDLTCvkvLQSAA------

d3np1a_ kctknalaQTGFNKDKYFngDVWYVTDYLDLepddvpKRYCAALAAGTASGKLKEALYHYDpkTQDTFYDVSELQEE-SPGKYTANFkkvekngnvkvdvtSGNYYTFTVMYAD-DSSALIHTCLHKgNKDLGDLYAVLNRNkDTNAGDKVKGAVTAASL---KFSDFISTKdNKCEY---DNVSLkslltk

d1euoa_ -MDCS-TNISPKQGLDKAKYFSGKWYVTHfldkdpqvtdQYCSsftPRESD-gtvkealyhynankkTSFYNIGEGKL-ESSGlQYTAKYKTVdkkkavLKEAdekNSYTLTVLEAD-DSSALVHICLREGSKDLGDLYTVLTHQKDaepsakvksavtqaglqlsqfvgtkdlGCQYDDQFTsl

d1avgi_ aEGDDcSIEKAMGDFKPEEFFNGTWYLAH-----gpgvtSPAVcqkFTTSGskgftqiveigynkfeSNVKFQCNQVDnKNGE-QYSFKCKSS------DNTE---FEADFTFISVSyDNFALVCRSITFTSQPKEDRYLVFERTKS--------------------------dTDPDAKEIC--

d1euoa_ mdcstnispkqgldkaKYFSgKWYVthFLDKD----------------pqVTDQ-YCSSFTPRESdgTVKEALYHYnankKTSFYNIGEGK------------------LESSGLQYTAkyktvdkkkavlkeadeKNSYTLTVLEaddssALVHICLREGSKDlGDLYTVLThqkdaepsakvksavtqaglqlsqfvgtkdlgcqyddqftsl

d1cbia_ ----------------PNFA-GTWK-mRSSENfdellkalgvnamlrkvaVAAAsKPHVEIRQDG--DQFYIKTST----TVRTTEINFKVgegfeeetvdgrkcrslpTWENENKIHC-------tqtllegdgpKTYWTRELAN-----DELILTFGADDVV-CTRIYVRE------------------------------------------

d1euoa_ mdcstnispKQGLDKAKYfSGKWYVTHFLDKDPQ--VTDQ--YCSSFTPRES-DGTVKEALYHYNanKKTSFYNIGEGKLESSGLQYTAKYktvdkkkavlkeadekNSYTLTVLEA-DDSSALVHICLREgSKDLGDLYTVLTHQKDAEpSAKVKSAVTQAGL---QLSQFVGTKDLgcQYDDQFTSl

d1ew3a_ -------vaIRNFDISKI-SGEWYSIFLASDVKEkiEENGsmRVFVDVIRALdNSSLYAEYQTKV--NGECTEFPMVFDKTEEDGVYSLNY---------------dGYNVFRISEFeNDEHIILYLVNFD-KDRPFQLFEFYAREPDVS-PEIKEEFVKIVQKrgiVKENIIDLTKI--DRCFQLRG-

d1euoa_ mdcstnispKQGLD-KAKYFsGKWYVTHFLD---KDPQ--vtdQYCSSFTPRES-DGTVKEALYHYNanKKTSFYNIGEGKLESSGLQYTAKYktvdkkkavlkeadeknSYTLTVLEAD-DSSALVHICLRegsKDLG--DLYTVLTHQKDAE--PSAKVKSAVTQAGLQLsQFVGtKDLGCQYDDqftsl

d1exsa_ ----vevtpIMTELdTQKVA-GTWHTVAMAVsdvSLLDaksspLKAYVEGLKPTpEGDLEILLQKRE--NDKCAQEVLLAKKTDIPAVFKINA---------------ldENQLFLLDTDyDSHLLLCMENS---ASPEhsLVCQSLARTLEVDdqIREKFEDALKTLSVPM-RILP-AQLEEQCRV-----

d1euoa_ mdcstnispkqgldKAKYFSGKWYVthFLDKD------------pqVTDQ--YCSSFTPRESD-gtVKEALYHYnankktSFYNI--------------------geGKLEsSGLQYTAKyktvdkkkavlkeadEKNSYTLTVLEaddssALVHICLREGSKDlGDLYTVLThqkdaepsakvksavtqaglqlsqfvgtkdlgcqyddqftsl

d1ftpa_ --------------VKEFAGIKYKL--DSQTNfeeymkaigvgaieRKAGlaLSPVIELEILDgdkFKLTSKTA------IKNTEftfklgeefdeetldgrkvkstITQD-GPNKLVHE-----------qkgdHPTIIIREFSK-----EQCVITIKLGDLV-ATRIYKAQ------------------------------------------

d1euoa_ mdcstnispkqglDKAKyfSGKWYVTHFLDKD----------pQVTD--QYCSSFTPRESDGTVKEALYHYnankktSFYNIGEGK--------------------LESS--GLQYTakyktvdkkkavlkeadeknsYTLTVlEADDSSALVHICLregskDLGDLYTVLTHQkdaepsakvksavtqaglqlsqfvgtkdlgcqyddqftsl

d1ggla_ -------------PPNL--TGYYRFVSQKNMEdylqalnislaVRKIalLLKPDKEIEHQGNHMTVRTLST------FRNYTVQFDvgvefeedlrsvdgrkcqtiVTWEeeHLVCV--------------qkgevpnRGWRH-WLEGEMLYLELTA-----RDAVCEQVFRKV---------------------------------------h

d1euoa_ mdcstnispKQGLDKAKYFsGKWYVTHFLDKDPQ--VTDQ--YCSSFTPRES-DGTVKEALYHYNanKKTSFYNIGEGKLEsSGLQYTAKYktvdkkkavlkeadekNSYTLTVLEAD-DSSALVHICLREGSKDlGDLYTVLTHQKdAEPSAkVKSAVTQAGL----QLSQFVGTKDLgcQYDDQFTSl

d1gm6a_ -------vvTSNFDASKIA-GEWYSILLASDAKEniEENGsmRVFVEHIRVLdNSSLAFKFQRKV--NGECTDFYAVCDKV-GDGVYTVAY---------------yGENKFRLLEVNySDYVILHLVDVNGDKT-FQLMEFYGRKP-DVEPK-LKDKFVEICQqygiIKENIIDLTKI--DRCFQLRG-

d1euoa_ --mdcSTNISPKQGLDKAKYFsGKWYVTHFLDKDPqvtdQYCSSFTPRES-DGTVKEALYHYNA---nKKTSFYNIGEGKLESSGLQYTAKYKTVDkkkaVLKEAdekNSYTLTVLEAD-DSSALVHICLR--egskdLGDLYTVLTHQKDAE---PSAKVKSAVTQAGLQlSQFVGTKdlGCQYDDqftsl

d1hbqa_ erdcrVSSFRVKENFDKARFA-GTWYAMAKKDPEG-lflQDNIVAEFSVDeNGQMSATAKGRVRllnnWDVCADMVGTFTDTEDPAKFKMKYWGVA----SFLQK---GNDDHWIIDTDyETFAVQYSCRLlnldgtcADSYSFVFARDPSGFspeVQKIVRQRQEELCLA-RQYRLIP-hNGYCNG-----

d1euoa_ mDCSTNISPKQGLDKAKYFsGKWYVTHFLDKDpqvtDQYCSSFTPRESDGTVKEALYHYNanKKTSFYNIGEGKLESSGLQYTAKyktvdkkkavlkeADEKNSYTLTVLEAD-DSSALVHICLregskDLGDLYTVLTHQkdAEPSAKVKSAVTQAGL----QLSQFVGTKdlgCQYDDQ------ftsl

d1iw2a_ aSPISTIQPKANFDAQQFA-GTWLLVAVGSAG----RAEATTLHVAPQGTAMAVSTFRKL--DGICWQVRQLYGDTGVLGRFLLQ------------aRGARGAVHVVVAETDyQSFAVLYLER-----AGQLSVKLYARS--LPVSDSVLSGFEQRVQeahlTEDQIFYFP--kYGFCEAadqfhvldev

d1euoa_ --mdcSTNISPKQGLDKAKYFsGKWYVTHFLDKDPqvtdQYCSSFTPRES-DGTVKEALYHYNA---nKKTSFYNIGEGKLESSGLQYTAKYKTVDkkkaVLKEAdekNSYTLTVLEAD-DSSALVHICLR--egskdLGDLYTVLTHQKDAE---PSAKVKSAVTQAGLQlSQFVGTKdlgCQYDdqftsl

d1jyda_ erdcrVSSFRVKENFDKARFS-GTWYAMAKKDPEG-lflQDNIVAEFSVDeTGQMSATAKGRVRllnnWDVCADMVGTFTDTEDPAKFKMKYWGVA----SFLQK---GNDDHWIVDTDyDTYAVQYSCRLlnldgtcADSYSFVFSRDPNGLppeAQKIVRQRQEELCLA-RQYRLIV--hNGYC------

d1euoa_ mdcstnispkqglDKAKyfSGKWYVTHFLDKD----------pQVTD--QYCSSFTPRESDGTVKEALYHYnankktSFYNIGEGK--------------------LESS--GLQYTAkyktvdkkkavlkeadeknsYTLTVlEADDSSALVHICLregskDLGDLYTVLTHQkdaepsakvksavtqaglqlsqfvgtkdlgcqyddqftsl

d1kqxa_ -------------PADF--NGTWEMLSNDNFEdvmkaldidfaTRKIavHLKQTKVIVQNGDKFETKTLST------FRNYEVNFVigeefdeqtkgldnrtvktlVKWDgdKLVCVQ--------------kgekenRGWKQ-WIEGDLLHLEIHC-----QDKVCHQVFKKK---------------------------------------n

d1euoa_ mdcstnispkqgldKAKYFsGKWYVTHFL------------dkdpqvtdqyCSSFTPRESDGTVKEALYHYnankktSFYNIGEGK------lessglqytakyktvdkkkavlkeadeknsytlTVLEADdSSALVHIClregskDLGDLYTVLTHQkdaepsakvksavtqaglqlsqfvgtkdlgcqyddqftsl

d1liba_ --------------CDAFV-GTWKLVSSEnfddymkevgvgfatrkvagmaKPNMIISVNGDLVTIRSEST------FKNTEISFKlgvefdeitaddrkvksiitldggalvqvqkwdgksttiKRKRDG-DKLVVECV-----mKGVTSTRVYERA----------------------------------------

d1euoa_ mdcstnispkqgldkakyfsgKWYVTHFLDKD----------------pqVTDQYCSSFTPREsdgtVKEALYHYNAnkKTSFYNIGE--------------------GKLEssGLQYTAKyktvdkkkavlkeaDEKNSYTLTVLEaddssALVHICLREGSKDlGDLYTVLThqkdaepsakvksavtqaglqlsqfvgtkdlgcqyddqftsl

d1lpja_ -----------------padlSGTWTLLSSDNfegymlalgidfatrkiaKLLKPQKVIEQNG----DSFTIHTNSS--LRNYFVKFKvgeefdednrgldnrkckslVIWD--NDRLTCI----------qkgeKKNRGWTHWIEG-----DKLHLEMFCEGQV-CKQTFQRA------------------------------------------

d1euoa_ --------mdcSTNIS---PKQGL--DKAKYFsGKWYVTHFLDKDpqvtdQYCSSFTPRESDGTVKEALYHYNaNKKTSFYNIGEGKLE--SSGLqYTAKYKTvdkkkavlkeadekNSYTLTVLEAD-DSSALVHIClregskdlGDLYTVLTHQKDAE--PSAKVKSAVTQAGLQLSQFVGTK-dlGCQY--DDQFtsl

d1obqa_ dkipdfvvpgkCASVDrnkLWAEQtpNRNSYA-GVWYQFALTNNP-yqliEKCVRNEYSFDGKQFVIESTGIA-YDGNLLKRNGKLYPNpfGEPH-LSIDYEN-------------sFAAPLVILETDySNYACLYSCidynfgyhSDFSFIFSRSANLAdqYVKKCEAAFKNINVDTTRFVKTVqgsSCPYdtQKTL---

d1euoa_ mdcstnispkQGLD---------kakyfsgKWYVTHFL------------------------------dkdpqvtdqYCSSFTpresdgtvkEALYHYNAnkKTSFYNIGEG-KLESS-------GLQYTAKYKtvdkkkaVLKEadekNSYTLTVL-eaddssALVHICLREGS------kdlGDLYTVLthqKDAE---PSAKVKSavtqaglqlsqfvgtkdlgcqyddqftsl

d1oeja_ -gkplteveqKAANgvfddanvqnrtlsdwDGVWQSVYpllqsgkldpvfqkkadadktktfaeikdyyhkgyatdiEMIGIE--------dGIVEFHRN--NETTSCKYDYdGYKILtyksgkkGVRYLFECK-------DPES-kapKYIQFSDHiiaprksSHFHIFMGNDSqqsllnemeNWPTYYP---YQLSseeVVEEMMS----------------------------h

d1euoa_ mdcstnispkqglDKAKyfSGKWYVTHFLD--KDPQ---------vTDQY-CSSFTPRESDGTVKEALYHYnankktSFYNIGEGK--------------------LESS--GLQYTakyktvdkkkavlkeadeknsYTLTVlEADDSSALVHICLregskDLGDLYTVLTHQkdaepsakvksavtqaglqlsqfvgtkdlgcqyddqftsl

d1opaa_ -------------TKDQ--NGTWEMESNENfeGYMKaldidfatrkIAVRlTQTKIIVQDGDNFKTKTNST------FRNYDLDFTvgvefdehtkgldgrnvktlVTWEgnTLVCV--------------qkgekenRGWKQ-WVEGDKLYLELTC-----GDQVCRQVFKKK----------------------------------------

d1euoa_ mdcstnispkqgldkaKYFSgKWYVTHFLD---KDPQ-------VTDQ--YCSSFTPRESDGTVKEALYHYnankktSFYNIGEGK------------------LESS--GLQYTakyktvdkkkavlkeadeknsYTLTVlEADDSSALVHICLregskDLGDLYTVLTHQkdaepsakvksavtqaglqlsqfvgtkdlgcqyddqftsl

d1p6pa_ ----------------AFNG-TWNVYAQENyenFLRTvglpediIKVAkdVNPVIEIEQNGNEFVVTSKTP------KQTHSNSFTvgkeseitsmdgkkikvtVQLEggKLICK------------------sdkFSHIQ-EVNGDEMVEKITI-----GSSTLTRKSKRV----------------------------------------

d1euoa_ ------------mdCSTNISPKQGLDKAKYFsGKWYVTHFLDkdpqvtDQYC-SSFTPRES-DGTVKEALYHYNANKKTSFYNIGEGKLESS--GLQYTAKYKTvdkkkavlkeadekNSYTLTVLEAD--DSSALVHIClregskdlgDLYTVLTHQKDAE--PSAKVKSAVTQAGLQLSQFVGTKDlgcqyddqftsl

d1qwda_ hlestslykkssstPPRGVTVVNNFDAKRYL-GTWYEIARFD--hrfeRGLEkVTATYSLRdDGGLNVINKGYNPDRGMWQQSEGKAYFTGAptRAALKVSFFG-------------pFYGGYNVIALDreYRHALVCGP-------drDYLWILSRTPTISdeVKQEMLAVATREGFDVSKFIWVQQ----------pg

d1euoa_ mdcstnispkqgldkakyFSGKWYVthFLDKD------pQVTDQYCSSFTPRESdgTVKEALYHYNankKTSFYNIGEGKlessglqyTAKYKTVDKkkavLKEADE---------------------knsYTLTVleaddssALVHICLREGS----KDLGDLYTVLTHqkdaepsakvksavtqaglqlsqfvgtkdlgcqyddqftsl

d1r0ua_ -----------gfqsnamKQETPIT-lHVKSVieddgnqEVIEFRTTGFYYVKQ--NKVYLSYYEEhdlGKVKTIVKVSE--------GEVLVMRSG-avkMNQRFVtgastiakykmsfgelelktstksIQSDL-----deEKGRISIAYDMhvghLHNMTITYEGGT-----------------------------------------

d1euoa_ mdcstnispkqgldkaKYFSgKWYVTHFLD--KDPQ--------VTDQ--YCSSFTPRESDGTVKEALYHYnankktSFYNIGEGK------------------LESS--GLQYTakyktvdkkkavlkeadeknsYTLTVlEADDSSALVHICLregskDLGDLYTVLTHQkdaepsakvksavtqaglqlsqfvgtkdlgcqyddqftsl

d1tvqa_ ----------------AFSG-TWQVYAQENyeEFLKalalpedlIKMArdIKPIVEIQQKGDDFVVTSKTP------RQTVTNSFTlgkeadittmdgkklkctVHLAngKLVTK------------------sekFSHEQ-EVKGNEMVETITF-----GGVTLIRRSKRV----------------------------------------

d1euoa_ -------------------------------------------------------------------------------------------------mdcSTNI-------------------------------------------spkqgldKAKYFSgKWYVtHFLDkdpQVTD-------------------------qYCSSFTPRESdgTVKEALYHYNankktSFYNI-------------------gEGKLESsGLQYTAKYktvdkkkavlkeadEKNSYTLTVLEaddsSALVHICLREGSKDLgDLYTVLTHqkdaepsakvksavtqaglqlsqfvgtkdlgcqyddqFTSL---------------------------------------

d1vpra1 ekgfeagdnklggalnakhvekygdnfkngxhkpefhedglhkpxevggkkfesgfhyllechelggknasggyggplcedpygsevqaxtekllkeadsDRTLcfnnfqdpcpqltkeqvaxckgfdygdktlklpcgplpwpaglpepgyvpKTNPLH-GRWI-TVSGgqaAFIKeaiksgmlgaaeankivadtdhhqtgGXYLRINQFG--DVCTVDASVA---kfARAKRtwksghyfyeplvsggnllgVWVLPE-EYRKIGFF--------wexesgRCFRIERRAFP-vgpYTFXRQATEVGGKIS-FVFYVKVS----------------------------------ndpESDPiplqsrdytalagrdnaptnlgkpyptlakdldypkkrd

d1euoa_ mdcstnispkqgldkAKYFsGKWYVTHFLD--KDPQ---------VTDQ---YCSSFTPRESDGTVKEALYHYnankktSFYNIGEGK------------------LESS---GLQYtakyktvdkkkavlkeadeknsyTLTVLEADDSSALVHICLregskDLGDLYTVLTHQkdaepsakvksavtqaglqlsqfvgtkdlgcqyddqftsl

d1xcaa_ ---------------PNFS-GNWKIIRSENfeELLKvlgvnvmlrKIAVaaaSKPAVEIKQEGDTFYIKTSTT------VRTTEINFKvgeefeeqtvdgrpckslVKWEsenKMVC-----------eqkllkgegpktSWTMELTNDGELILTMTA-----DDVVCTRVYVRE----------------------------------------

d1euoa_ mdcstnispkqgldkaKYFSgKWYVTHFLDkdpqvtdqYCSSFTPRES-DGTVKEALYHynanKKTSFYNIGEGKLESSGLQYTAKyktvdkkkavlkeadeKNSYTLTVLEA-DDSSALVHICLRegSKDLgDLYTVLTHQKDAEPSakVKSAVTQAGL----qlSQFVGTKDlgcqyddqftsl

d1xkia_ ----------------DVSG-TWYLKAMTV-----nleSVTPMTLTTLeGGNLEAKVTM----SGRCQEVKAVLEKTDEPGKYTAD----------------GGKHVAYIIRShVKDHYIFYSEGE--GKPV-RGVKLVGRDPKNNLE--ALEDFEKAAGarglstESILIPRQ-----------s

d1euoa_ mdcsTNISPKQGLDKAKYfSGKWYVTHFLDKDPQ--VTDQ---YCSSFTPRES-DGTVKEALYHYNanKKTSFYNIGEGKLESSGLQYTAKYktvdkkkavlkeadekNSYTLTVLEAD-DSSALVHICLRegsKDLG--DLYTVLTHQKdAEPSAkVKSAVTQAGL--qlsQFVGTKD-lgCQYDdqftsl

d1yupa1 ---iIVTQTMKDLDVQKV-AGTWYSLAMAASDISllDAQSaplRVYVEELKPTpGGDLEILLQKWE--NGKCAQKKIIAEKTEIPAVFKIDA---------------lNENKVLVLDTDyKKYLLFCMENS---AEPEqsLACQCLVRTP-EVDDE-AMEKFDKALKalpmhIRLSFNPtqlEEQC----rv

d1euoa_ mdcstnispkqglDKAKyFSGKWYVtHFLDKD-PQVTDQYCSSFTPRES-DGTVKEALYHYNANKKT-SFYNIGEGKLESSglqytAKYKTVDkkkavLKEAD-----------------------eknsYTLTVlEADDSSALVHICLRE-gSKDLGDLYTVLTHQkdaepsakvksavtqaglqlsqfvgtkdlgcqyddqftsl

d2a13a1 -----ppvhpfvaPLSY-LLGTWRG-QGEGEYpTIPSFRYGEEIRFSHSgKPVIAYTQKTWKLESGApXHAESGYFRPRPD-gsieVVIAQST-----GLVEVqkgtynvdeqsiklksdlvgnaskvkeISREF-ELVDGKLSYVVRXSTttNPLQPHLKAILDKL----------------------------------------

d1euoa_ mdcstnispkqgldkAKYFsGKWYVTHFLDKDP----------qVTDQ--YCSSFTPRESDGTVKEALYHYnankktSFYNIGEGK------------------LESS---GLQYTakyktvdkkkavlkeadekNSYTLTVlEADDSSALVHICLregskDLGDLYTVLTHQkdaepsakvksavtqaglqlsqfvgtkdlgcqyddqftsl

d2f73a1 ---------------MSFS-GKYQLQSQENFEAfmkaiglpeelIQKGkdIKGVSEIVQNGKHFKFTITAG------SKVIQNEFTvgeeceletmtgekvktvVQLEgdnKLVTT------------------fKNIKSVT-ELNGDIITNTMTL-----GDIVFKRISKRI----------------------------------------

d1euoa_ mdcstnispkqglDKAKYFsGKWYVThfldkdPQVTDQYCSSFTPRES-DGTVKEALYHYNANKKTS-FYNIGEGKLESSGlqytAKYKTVdkkkaVLKEADE---------------------------------KNSYTLTVLEaddssALVHICLREG-----SKDLgdLYTVLTHQkdaepsakvksavtqaglqlsqfvgtkdlgcqyddqftsl

d2fr2a1 ------dlapalqALSPLL-GSWAGRgagkypTIRPFEYLEEVVFAHVgKPFLTYTQQTRAVADGKPlHSETGYLRVCRPG-cveLVLAHP-----SGITEIEvgtysvtgdvielelstradgsiglaptakevtALDRSYRIDG-----DELSYSLQMRavgqpLQDH-lAAVLHRQR----------------------------------------

d1euoa_ mdcstnispkqgLDKAKYFSGKWYVtHFLDkdpqvtdQYCSSFTPREsdgTVKEALYHYNankktSFYNI-GEGKLESSG---LQYTAKYktvdkkkavlkeaDEKNSYTLTVLEAddssALVHICLREGS-------------------------------KDLGDLYTV-LTHQkdaepsakvksavtqaglqlsqfvgtkdlgcqyddQFTS-------------------------l

d2gc9a1 -------xtktfKTLDDFLGTHFIY-TYDN-------GWEYEWYAKN---DHTVDYRIHG-gxvaGRWVTdQKADIVXLTegiYKISWTE-------------PTGTDVALDFXPN---eKKLHGTIFFPKwveehpeitvtyqnehidlxeqsrekyatypKLVVPEFANiTYXG----------------------------dagqnneDVISeapykexpndirngkyfdqnyhrlnk

d1euoa_ mdcstnispkQGLDKAKYfSGKWYVtHFLDKD--PQVTDQYCSSFTPRE-sdgtvKEALYHYnankktSFYNI-----------------GEGKLE---ssgLQYTAkyktvdkkkavlkeADEKNSYTLTVLEADDSSALVHICLREG-SKDLGDLYTVLTHQkdaepsakvksavtqaglqlsqfvgtkdlgcqyddqftsl

d2o62a1 --------erPLLQINDL-LGEWRG-QAVTIYrdLRPPDIYSTTLKIQLddagrlXQSTSFG------ERTITstatikgsivlfdqdpeKQVQVLllpdgaSATSP-----------lkvQLRQPLFLEAGWLIQSDLRQRXIRSYNDkGEWVSLTLVTEERV----------------------------------------

d1euoa_ mDCSTNISPKQGLDKAKYFS-GKWYVTHFLDKDPQV-TDQYCSSFTPRESDGTVKEALYHYNANKKTSFYNIGEGKLEsSGLQYTAKYKTVDKKKAVLKEADEKNSYTLTVLEADDSSALVHICLREGSKDLGDLYTVLTHQKDAEPSAKVKSAVTQAGLQLSQFVGTKDLGCQYDDQFTSL----

d2ofmx1 -ACTKNAIAQTGFNKDKYFNgDVWYVTDYLDLEPDDvPKRYCAALAAGTASGKLKEALYHYDPKTQDTFYDVSELQVE-SLGKYTANFKKVDKNGNVKVAVTAGNYYTFTVMYADDSSALIHTCLHKGNKDLGDLYAVLNRNKDAAAGDKVKSAVSAATLEFSKFISTKENNCAYDNDSLKSlltk

d1euoa_ mdcstnisPKQG-LDKAKYfSGKWYVTHFLDKDPQ--VTDQ--YCSSFTPRESDGTVKEALYHYNanKKTSFYNIGEGKLESSGLQYTAKYktvdkkkavlkeadekNSYTLTVLEAD-DSSALVHICLREGSKDlGDLYTVLTHQKDAEpSAKVKSAVTQAGL---QLSQFVGTKDlgCQYDdqftsl

d2ozqa1 ----eeasSTGRnFNVEKI-NGEWHTIILASDKREkiEDNGnfRLFLEQIHVLEKSLVLKFHTVR--DEECSELSMVADKTEKAGEYSVTY---------------dGFNTFTIPKTDyDNFLMAHLINEKDGET-FQLMGLYGREPDLS-SDIKERFAQLCEEhgiLRENIIDLSN--ANRC------

d1euoa_ mDCSTNISPKQGLDKAKYFS-GKWYVTHFLDKD-PQVTDQYCSSFTPRESDGTVKEALYHYNANKKTSFYNIGEGKLEsSGLQYTAKYKTVDKKKAVLKEADEKNSYTLTVLEADDSSALVHICLREGSKDLGDLYTVLTHQKDAEPSAKVKSAVTQAGLQLSQFVGTKDLGCQYDDQFTSL----

d3np1a_ -KCTKNALAQTGFNKDKYFNgDVWYVTDYLDLEpDDVPKRYCAALAAGTASGKLKEALYHYDPKTQDTFYDVSELQEE-SPGKYTANFKKVEKNGNVKVDVTSGNYYTFTVMYADDSSALIHTCLHKGNKDLGDLYAVLNRNKDTNAGDKVKGAVTAASLKFSDFISTKDNKCEYDNVSLKSlltk

d1ew3a_ ---------vaIRNFDISKI-SGEWYSIF------------------lasdvkekiEENGSmrvfvdviraldnsslyaeyqtkvngECTEFPMVFDK-TEEDgVYSLNY------DGYNVFRISEFENDEHIILYLVNFD-KDRPFQLFEFYAREPDvspeikeefvkivqkrgivkeniidlTKIDRCFQLRg

d1avgi_ aegddcsiekaMGDFKPEEFfNGTWYLAHgpgvtspavcqkfttsgskgftqiveiGYNKF-------------------------eSNVKFQCNQVDnKNGE-QYSFKCkssdntEFEADFTFISVSYDNFALVCRSITFtSQPKEDRYLVFERTKS--------------------------DTDPDAKEIC-

d1ew3a_ vairnfdiSKISGEWYSIFLASDV-------------kEKIEengSMRVFVDVIRALDnSSLYAEYQTKvngeCTEFPMVFDK-----------------TEED----GVYS----lnydGYNVFRISEFEndEHIILYLVNFdkdrPFQLFEFYAREpdvspeikeefvkivqkrgivkeniidltkidrcfqlrg

d1cbia_ --------PNFAGTWKMRSSENFDellkalgvnamlrkVAVA---AASKPHVEIRQDG-DQFYIKTSTT----VRTTEINFKVgegfeeetvdgrkcrslPTWEnenkIHCTqtllegdgPKTYWTRELAN--DELILTFGAD----DVVCTRIYVRE---------------------------------------

d1ew3a_ --VAIRN--fDISKISGEWYSIFLASD-VKEKieeNGSMR-VFVDVIRALDNSSLYAEYQTKVNGECTEFPMVFDKTEEDGVYSLNYDGYNVFRISEFENDEHIILYLVNFDKdrPFQLFEFYAREPDVSPEIKEEFVKIVQKRGIVKeNIIDLTKIDRCFqlrg

d1exsa_ veVTPIMtelDTQKVAGTWHTVAMAVSdVSLL-daKSSPLkAYVEGLKPTPEGDLEILLQKRENDKCAQEVLLAKKTDIPAVFKINALDENQLFLLDTDYDSHLLLCMENSASpeHSLVCQSLARTLEVDDQIREKFEDALKTLSVPM-RILPAQLEEQCR---v

d1ew3a_ vairnfdISKISG-EWYSIFLAS--DVKE------kiEENGsmRVFVDVIRALDNSSLYAEYQTKvngeCTEFPMV------------------fDKTEED---GVYSLnydGYNVFRISEFenDEHIILYLVNFdkdrPFQLFEFYAREpdvspeikeefvkivqkrgivkeniidltkidrcfqlrg

d1ftpa_ -------VKEFAGiKYKLDSQTNfeEYMKaigvgaieRKAGlaLSPVIELEILDGDKFKLTSKTA----IKNTEFTfklgeefdeetldgrkvksTITQDGpnkLVHEQkgdHPTIIIREFS--KEQCVITIKLG----DLVATRIYKAQ---------------------------------------

d1ew3a_ vairnfdISKISGEWYSIFLAS--DVKE------kiEENGSmRVFVDVIRALDNsSLYAEYQTKvngeCTEFPMV--------------------fdKTEED--GVYSL-nydGYNVFRISEfeNDEHIILYLVNFdkdrPFQLFEFYAREpdvspeikeefvkivqkrgivkeniidltkidrcfqlrg

d1ggla_ -------PPNLTGYYRFVSQKNmeDYLQalnislavRKIALlLKPDKEIEHQGN-HMTVRTLST----FRNYTVQfdvgvefeedlrsvdgrkcqtiVTWEEehLVCVQkgevPNRGWRHWL--EGEMLYLELTAR----DAVCEQVFRKV--------------------------------------h

d1ew3a_ vAIRN-FDISKISGEWYSIFLASDVKEKIEENGSMRVFVDVIRALDNSSLYAEYQTKVNGECTEFPMVFDKTeEDGVYSLNYDGYNVFRISEFENDEHIILYLVNFDKDRPFQLFEFYAREPDVSPEIKEEFVKIVQKRGIVKENIIDLTKIDRCFQLRG

d1gm6a_ -VVTSnFDASKIAGEWYSILLASDAKENIEENGSMRVFVEHIRVLDNSSLAFKFQRKVNGECTDFYAVCDKV-GDGVYTVAYYGENKFRLLEVNYSDYVILHLVDVNGDKTFQLMEFYGRKPDVEPKLKDKFVEICQQYGIIKENIIDLTKIDRCFQLRG

d1ew3a_ ---------vaIRNFDISKISGEWYSIFLASdVKEKieengSMRV-FVDVIRALDNSSLYAEYQTKVN-----GECTEFPMVFDKTEEDGVYSLNY--------DGYNVFRISEFENDEHIILYLVNFDKD---rPFQLFEFYAREP-DVSPEIKEEFVKIVQKRGIVKeNIIDLtKIDRCFQLrg

d1hbqa_ erdcrvssfrvKENFDKARFAGTWYAMAKKD-PEGL-----FLQDnIVAEFSVDENGQMSATAKGRVRllnnwDVCADMVGTFTDTEDPAKFKMKYwgvasflqKGNDDHWIIDTDYETFAVQYSCRLLNLdgtcADSYSFVFARDPsGFSPEVQKIVRQRQEELCLAR-QYRLI-PHNGYCNG--

d1ew3a_ -------vaiRNFDISKISGEWYSIFLASDVkekieengsmrvFVDVIRALdNSSLYAEYQTKVNGECTEFPMVFDKTEEDGVYSLNY---DGYNVFRISEFENDEHIILYLVNFdkdrPFQLFEFYAREPDVSPEIKEEFVKIVQKRGIVKENIIDLTKIDRCFQL-------rg

d1iw2a_ aspistiqpkANFDAQQFAGTWLLVAVGSAG--------raeaTTLHVAPQ-GTAMAVSTFRKLDGICWQVRQLYGDTGVLGRFLLQArgaRGAVHVVVAETDYQSFAVLYLERA----GQLSVKLYARSLPVSDSVLSGFEQRVQEAHLTEDQIFYFPKYGFCEAAdqfhvldev

d1ew3a_ ---------vaIRNFDISKISGEWYSIFLASdVKEKieengSMRV-FVDVIRALDNSSLYAEYQTK-----vNGECTEFPMVFDKTEEDGVYSLNY--------DGYNVFRISEFENDEHIILYLVNFDKD---rPFQLFEFYAREP-DVSPEIKEEFVKIVQKRGIVKeNIIDLTkidRCFQLrg

d1jyda_ erdcrvssfrvKENFDKARFSGTWYAMAKKD-PEGL-----FLQDnIVAEFSVDETGQMSATAKGRvrllnnWDVCADMVGTFTDTEDPAKFKMKYwgvasflqKGNDDHWIVDTDYDTYAVQYSCRLLNLdgtcADSYSFVFSRDPnGLPPEAQKIVRQRQEELCLAR-QYRLIV---HNGYC--

d1ew3a_ vairnfdiSKISGEWYSIFLAS--DVKE------kiEENGSmRVFVDVIRALDnSSLYAEYQTKvngeCTEFPMVF--------------------DKTEED--GVYS-lnydGYNVFRISEFenDEHIILYLVNFdkdrPFQLFEFYAREpdvspeikeefvkivqkrgivkeniidltkidrcfqlrg

d1kqxa_ -------pADFNGTWEMLSNDNfeDVMKaldidfatRKIAVhLKQTKVIVQNG-DKFETKTLST----FRNYEVNFvigeefdeqtkgldnrtvktLVKWDGdkLVCVqkgekENRGWKQWIE--GDLLHLEIHCQ----DKVCHQVFKKK--------------------------------------n

d1ew3a_ vairnfdISKISGEWYSIFLAS--DVKE------kiEENGSmRVFVDVIRALDNssLYAEYQTKvngeCTEFPMV------------------fDKTEED--GVYSLN-ydGYNVFRISEFEndEHIILYLVNFdkdrPFQLFEFYAREpdvspeikeefvkivqkrgivkeniidltkidrcfqlrg

d1liba_ -------CDAFVGTWKLVSSENfdDYMKevgvgfatRKVAGmAKPNMIISVNGD-lVTIRSEST----FKNTEISfklgvefdeitaddrkvksIITLDGgaLVQVQKwdgKSTTIKRKRDG--DKLVVECVMK----GVTSTRVYERA---------------------------------------

d1ew3a_ vairnfdISKISGEWYSIFLAS-DVKEK-------ieengsmrVFVDVIRALDnSSLYAEYQTKvngeCTEFPMV--------------------fDKTEED--GVYSLN-yDGYNVFRISEFenDEHIILYLVNFdkdrPFQLFEFYAREpdvspeikeefvkivqkrgivkeniidltkidrcfqlrg

d1lpja_ -------PADLSGTWTLLSSDNfEGYMLalgidfatrkiakllKPQKVIEQNG-DSFTIHTNSS----LRNYFVKfkvgeefdednrgldnrkcksLVIWDNdrLTCIQKgeKKNRGWTHWIE--GDKLHLEMFCE----GQVCKQTFQRA---------------------------------------

d1ew3a_ --------------------vairnfDISKISGEWYSIFLASdvkekieenGSMRV---FVDVIRALDnSSLYAEYQTKV-NGECTEFPMVFDKT--EEDGvYSLNY--DGYNVFRISEFENDEHIILYLVnFDKD--RPFQLFEFYAREPDVSPEIKEEFVKIVQKRGIVKENIIDLTKI-DRCF-QLRG-

d1obqa_ dkipdfvvpgkcasvdrnklwaeqtpNRNSYAGVWYQFALTN--------nPYQLIekcVRNEYSFDG-KQFVIESTGIAyDGNLLKRNGKLYPNpfGEPH-LSIDYenSFAAPLVILETDYSNYACLYSC-IDYNfgYHSDFSFIFSRSANLADQYVKKCEAAFKNINVDTTRFVKTVQGsSCPYdTQKTl

d1ew3a_ ------------------vairnfDISKISGEWYSIF----------------------lasdvkekieengsMRVFVDVIRAldnssLYAEYQTKvngeCTEFPMVFDKTEED------------GVYSLNYD---gyNVFRISEF----enDEHIILYLVNF---dkdrpfqLFEFYAREPD----VSPEIKEEfvkivqkrgivkeniidltkidrcfqlrg

d1oeja_ gkplteveqkaangvfddanvqnrTLSDWDGVWQSVYpllqsgkldpvfqkkadadktktfaeikdyyhkgyaTDIEMIGIED-----GIVEFHRN----NETTSCKYDYDGYKiltyksgkkgvrYLFECKDPeskapKYIQFSDHiiaprkSSHFHIFMGNDsqqsllnemeNWPTYYPYQLsseeVVEEMMSH-----------------------------

d1ew3a_ vairnfdISKISGEWYSIFLAS--DVKE------kiEENGSMrvFVDVIRALDnSSLYAEYQTKvngeCTEFPMVFDK-------------------TEED---GVYS-lnydGYNVFRISEFenDEHIILYLVNFdkdrPFQLFEFYAREpdvspeikeefvkivqkrgivkeniidltkidrcfqlrg

d1opaa_ -------TKDQNGTWEMESNENfeGYMKaldidfatRKIAVRltQTKIIVQDG-DNFKTKTNST----FRNYDLDFTVgvefdehtkgldgrnvktlVTWEgntLVCVqkgekENRGWKQWVE--GDKLYLELTCG----DQVCRQVFKKK---------------------------------------

d1ew3a_ vairnfdisKISGEWYSIFLAS--DVKE------kiEENGsmRVFVDVIRALDnSSLYAEYQTKvngeCTEFPMVFDK----------------TEED----GVYSlnydgYNVFRISEfeNDEHIILYLVNFdkdrPFQLFEFYAREpdvspeikeefvkivqkrgivkeniidltkidrcfqlrg

d1p6pa_ ---------AFNGTWNVYAQENyeNFLRtvglpediIKVAkdVNPVIEIEQNG-NEFVVTSKTP----KQTHSNSFTVgkeseitsmdgkkikvTVQLeggkLICK---sdKFSHIQEV--NGDEMVEKITIG----SSTLTRKSKRV---------------------------------------

d1ew3a_ VAIR-------------------NFDISKISGEWYSIFLASdvkekieenGSMR---vFVDVIRALDNSSLYAEYQTKV--NGECTEFPMVFDKTEE--DGVYSLNYD--GYNVFRI-SEFENDEHIILYLVnfdkdrpFQLFEFYAREPDVSPEIKEEFVKIVQKRGIVKENIIDLTKIdrcfqlrg

d1qwda_ HLEStslykkssstpprgvtvvnNFDAKRYLGTWYEIARFD--------hRFERglekVTATYSLRDDGGLNVINKGYNpdRGMWQQSEGKAYFTGAptRAALKVSFFgpFYGGYNViALDREYRHALVCGP------dRDYLWILSRTPTISDEVKQEMLAVATREGFDVSKFIWVQQP-------g

d1ew3a_ vairnfdiskISGEWYSiFLASDV------KEKIeengsmRVFVDVIRALDnSSLYAEYQTKVNGECTEFPMVFDKTeedgvySLNY--------DGYN------------------------VFRISEfendEHIILYLVNFDKD-RPFQLFEFYAREPdvspeikeefvkivqkrgivkeniidltkidrcfqlrg

d1r0ua_ --gfqsnamkQETPITL-HVKSVIeddgnqEVIE------FRTTGFYYVKQ-NKVYLSYYEEHDLGKVKTIVKVSEG------EVLVmrsgavkmNQRFvtgastiakykmsfgelelktstkSIQSDL--deEKGRISIAYDMHVgHLHNMTITYEGGT--------------------------------------

d1ew3a_ vairnfdisKISGEWYSIFLAS--DVKE------kiEENGSmRVFVDVIRALDnSSLYAEYQTKvngeCTEFPMV------------------fdKTEED--GVYSlnydgYNVFRISEFenDEHIILYLVNFdkdrPFQLFEFYAREpdvspeikeefvkivqkrgivkeniidltkidrcfqlrg

d1tvqa_ ---------AFSGTWQVYAQENyeEFLKalalpedlIKMARdIKPIVEIQQKG-DDFVVTSKTP----RQTVTNSftlgkeadittmdgkklkctVHLANgkLVTK---seKFSHEQEVK--GNEMVETITFG----GVTLIRRSKRV---------------------------------------

d1ew3a_ ----------------------------------------------------------------------------------------------------------------------------------------------------vairnfdiSKISGEWYSIFL---------------asdvkekieeNGSM---rVFVDVIRALDnSSLYAEYQTKvngecTEFPMV------------------fDKTEED---GVYSLNY---dGYNVFRISEFEN--DEHIILYLVNFdkdrPFQLFEFYARE--------pdvspeikeefvkivqkrgivkeniidltkidrcfqlrg

d1vpra1 ekgfeagdnklggalnakhvekygdnfkngxhkpefhedglhkpxevggkkfesgfhyllechelggknasggyggplcedpygsevqaxtekllkeadsdrtlcfnnfqdpcpqltkeqvaxckgfdygdktlklpcgplpwpaglpepgyvpktNPLHGRWITVSGgqaafikeaiksgmlgaaeankivaDTDHhqtgGXYLRINQFG-DVCTVDASVA---kfARAKRTwksghyfyeplvsggnllgVWVLPEeyrKIGFFWExesgRCFRIERRAFPVgpYTFXRQATEVG----GKISFVFYVKVsndpesdpiplqsrdytalagrdnaptnlgkpyptlakdldypkkrd

d1ew3a_ vairnfdiSKISGEWYSIFLAS--DVKE-------kiEENG-smRVFVDVIRALDnSSLYAEYQTKvngeCTEFPMVFDK-----------------TEED----GVYS----lnydGYNVFRISEFENdEHIILYLVNFdkdrPFQLFEFYAREpdvspeikeefvkivqkrgivkeniidltkidrcfqlrg

d1xcaa_ --------PNFSGNWKIIRSENfeELLKvlgvnvmlrKIAVaaaSKPAVEIKQEG-DTFYIKTSTT----VRTTEINFKVgeefeeqtvdgrpckslVKWEsenkMVCEqkllkgegPKTSWTMELTND-GELILTMTAD----DVVCTRVYVRE---------------------------------------

d1ew3a_ vairnfdisKISGEWYSIFLASdvkekieenGSMRVFVDVIRALDNSSLYAEYQTkvNGECTEFPMVFDKTEEDGVYSLNYdgYNVFRISEFENDEHIILYLVNFdkDRPFQLFEFYAREP-DVSPeIKEEFVKIVQKRGIVKENIIDlTKIDrcfqlrg

d1xkia_ ---------DVSGTWYLKAMTV---------NLESVTPMTLTTLEGGNLEAKVTM--SGRCQEVKAVLEKTDEPGKYTADG-gKHVAYIIRSHVKDHYIFYSEGE--GKPVRGVKLVGRDPkNNLE-ALEDFEKAAGARGLSTESILI-PRQS-------

d1ew3a_ ----vaIRNFDISKISGEWYSIFLASDVKEKIE-ENGSMRVFVDVIRALDNSSLYAEYQTKVNGECTEFPMVFDKTEEDGVYSLNYDGYNVFRISEFENDEHIILYLVNFDKdRPFQLFEFYAREPDVSPEIKEEFVKIVQKRGIVkeNIIDLTK---iDRCFqlrg

d1yupa1 iivtqtMKDLDVQKVAGTWYSLAMAASDISLLDaQSAPLRVYVEELKPTPGGDLEILLQKWENGKCAQKKIIAEKTEIPAVFKIDALNENKVLVLDTDYKKYLLFCMENSAEpEQSLACQCLVRTPEVDDEAMEKFDKALKALPMH--IRLSFNPtqleEQCR---v

d1ew3a_ --vairnfDISKISGEWYSiFLASDV---KEKIeengsmRVFVDVIRALDNSSLYAEYQTK--vNGEC-TEFPMV--------------------fdkTEED------GVYSL------nyDGYNVFRISEFEndEHIILYLVNFDKDR--pFQLFEFYAREpdvspeikeefvkivqkrgivkeniidltkidrcfqlrg

d2a13a1 ppvhpfvaPLSYLLGTWRG-QGEGEYptiPSFR------YGEEIRFSHSGKPVIAYTQKTWkleSGAPxHAESGYfrprpdgsievviaqstglvevqKGTYnvdeqsIKLKSdlvgnaskVKEISREFELVD--GKLSYVVRXSTTTNplqPHLKAILDKL---------------------------------------

d1ew3a_ vairnfdiSKISGEWYSIFLA--------sdvkekieengsmrvFVDVIRALDnSSLYAEYQTKvngeCTEFPMVFDK-----------------TEED----GVYSlnydGYNVFRISEFenDEHIILYLVNFdkdrPFQLFEFYAREpdvspeikeefvkivqkrgivkeniidltkidrcfqlrg

d2f73a1 --------MSFSGKYQLQSQEnfeafmkaiglpeeliqkgkdikGVSEIVQNG-KHFKFTITAG----SKVIQNEFTVgeeceletmtgekvktvVQLEgdnkLVTT---fKNIKSVTELN--GDIITNTMTLG----DIVFKRISKRI---------------------------------------

d1ew3a_ -vairnfDISKISGEWYSiFLASDV---KEKIeengsmRVFVDVIRALDNSSLYAEYQTK--vNGEC-tEFPMVFD--------------------kTEED--------GVYS------------lnydGYNVFRISEFenDEHIILYLVNFDKDR--PFQLFEFYAREpdvspeikeefvkivqkrgivkeniidltkidrcfqlrg

d2fr2a1 dlapalqALSPLLGSWAG-RGAGKYptiRPFE------YLEEVVFAHVGKPFLTYTQQTRavaDGKPlhSETGYLRvcrpgcvelvlahpsgiteieVGTYsvtgdvieLELStradgsiglaptakevTALDRSYRID--GDELSYSLQMRAVGQplQDHLAAVLHRQ--------------------------------------r

d1ew3a_ --vairnfdiSKISGEWYSIFLasdvkekieengsmrVFVDVIRALDnSSLYAEYQTkvngectEFPMV-FDKT-------eedGVYSLNydGYNVFRISEFENDEHIILYLVNFD----------------------------KDRPFQLFEFYAREpdvspeikeefvkivqkrgivkeniidltkidrCFQLRG-----------------

d2gc9a1 xtktfktlddFLGTHFIYTYDN--------------gWEYEWYAKND-HTVDYRIHG---gxvaGRWVTdQKADivxltegiykISWTEP--TGTDVALDFXPNEKKLHGTIFFPKwveehpeitvtyqnehidlxeqsrekyaTYPKLVVPEFANIT----------------yxgdagqnnedviseapYKEXPNdirngkyfdqnyhrlnk

d1ew3a_ VAIRNfDISKISGEWYSiFLASDV----KEKIeengsmRVFVDVIRALDNSSLYAEYQTkvngeCTEFPMVF---------------dkTEED-----GVYS----lnydGYNVFRISEFeNDEHIILYLVNFD--KDRPFQLFEFYAREpdvspeikeefvkivqkrgivkeniidltkidrcfqlrg

d2o62a1 ERPLL-QINDLLGEWRG-QAVTIYrdlrPPDI------YSTTLKIQLDDAGRLXQSTSF----gERTITSTAtikgsivlfdqdpekqvQVLLlpdgaSATSplkvqlrqPLFLEAGWLI-QSDLRQRXIRSYNdkGEWVSLTLVTEERV---------------------------------------

d1ew3a_ ------vaIRNFDISKI--SGEWYSIFLASDVKEKIEENgsmRVFVDVIRALDNSSLYAEYQTKVNGECTEFPMV-FDKTeEDGVYSLNY---------------dGYNVFRISEFeNDEHIILYLVNFDKDRP-FQLFEFYAREP-DVSPEIKEEFVKIVQKrgiVKENIIDLTKIDRcFQLRG--------

d2ofmx1 actknaiaQTGFNKDKYfnGDVWYVTDYLDLEPDDVPKR---YCAALAAGTASGKLKEALYHYDPKTQDTFYDVSeLQVE-SLGKYTANFkkvdkngnvkvavtagNYYTFTVMYA-DDSSALIHTCLHKGNKDlGDLYAVLNRNKdAAAGDKVKSAVSAATL---EFSKFISTKENNC-AYDNDslkslltk

d1ew3a_ vAIRNF-----DISKISGEWYSIFLASDVKEKIEENGSMRVFVDVIRALdNSSLYAEYQTKVNGECTEFPMVFDKTEEDGVYSLNYDGYNVFRISEFENDEHIILYLVNFDKDRPFQLFEFYAREPDVSPEIKEEFVKIVQKRGIVKENIIDLTKiDRCFqlrg

d2ozqa1 -EEASStgrnfNVEKINGEWHTIILASDKREKIEDNGNFRLFLEQIHVL-EKSLVLKFHTVRDEECSELSMVADKTEKAGEYSVTYDGFNTFTIPKTDYDNFLMAHLINEKDGETFQLMGLYGREPDLSSDIKERFAQLCEEHGILRENIIDLSN-ANRC----

d1ew3a_ ------vaIRNFDISKI--SGEWYSIFLASDVKEkiEENGsmrVFVDVIRALDNSSLYAEYQTKVNGECTEFPMV-FDKTeEDGVYSLNY---------------dGYNVFRISEFeNDEHIILYLVNFD-KDRPFQLFEFYAREPDVS-PEIKEEFVKIVQKrgiVKENIIDLTKIDRCfQLRG--------

d3np1a_ kctknalaQTGFNKDKYfnGDVWYVTDYLDLEPDdvPKRY---CAALAAGTASGKLKEALYHYDPKTQDTFYDVSeLQEE-SPGKYTANFkkvekngnvkvdvtsgNYYTFTVMYA-DDSSALIHTCLHKgNKDLGDLYAVLNRNKDTNaGDKVKGAVTAASL---KFSDFISTKDNKCE-YDNVslkslltk

d1exsa_ ------vevtpimTELDTQKVAGtwhtvamavsdvslldakssplkayveglkpTPEGdleILLQKrendkcaqevLLAKKTDIP----AVFKINA--ldeNQLFLLDTDY----dSHLLLCMENSASpehSLVCQSLArtlevddqirekfedalktlsvpmrilpaqleEQCRV------

d1avgi_ aegddcsiekamgDFKPEEFFNG---------twylahgpgvtspavcqkfttsGSKGftqIVEIG-ynkfesnvkFQCNQVDNKngeqYSFKCKSsdnteFEADFTFISVsydnfALVCRSITFTSQ---PKEDRYLV---------------------------fertkSDTDPdakeic

d1exsa_ vevtpimteldtQKVAGTWHTVAMAV---SDVSL-----lDAKSS-pLKAYVEGLKPTPeGDLEILLQkrendKCAQEVLLAKKTDI---------------------pAVFK----inaldeNQLFLLDTdyDSHLLLCMEnsasPEHSLVCQSLARTlevddqirekfedalktlsvpmrilpaqleeqcrv

d1cbia_ ------------PNFAGTWKMRSSENfdeLLKALgvnamlRKVAVaaASKPHVEIRQDG-DQFYIKTS----tTVRTTEINFKVGEGfeeetvdgrkcrslptwenenkIHCTqtllegdgpkTYWTRELA--NDELILTFG----ADDVVCTRIYVRE-----------------------------------

d1exsa_ vevtpimtelDTQKVAGTWHTVAMAV--sdvSLLDAK----sspLKAYVEGLKPTP--EGDLEILLQkrendkCAQEVLLAK----------------kTDIP------AVFKinaldeNQLFLLDTdyDSHLLLCMENSAspehSLVCQSLARTlevddqirekfedalktlsvpmrilpaqleeqcrv

d1ftpa_ ----------VKEFAGIKYKLDSQTNfeeymKAIGVGaierkagLALSPVIELEILdgDKFKLTSKT------AIKNTEFTFklgeefdeetldgrkvkSTITqdgpnkLVHEqkgdhpTIIIREFS--KEQCVITIKLGD----LVATRIYKAQ-----------------------------------

d1exsa_ vevtpimteLDTQkvAGTWHTVAMAVsdvSLLD--------AKSSplKAYVEGLKPTPEGdLEILLQKREndkcAQEVLL--------------------aKKTDIP--AVFKI-naldeNQLFLLDTdyDSHLLLCMENSAspehSLVCQSLARTlevddqirekfedalktlsvpmrilpaqleeqcrv

d1ggla_ ---------PPNL--TGYYRFVSQKN-meDYLQalnislavRKIAllLKPDKEIEHQGNH-MTVRTLSTF----RNYTVQfdvgvefeedlrsvdgrkcqtIVTWEEehLVCVQkgevpnRGWRHWLE--GEMLYLELTARD----AVCEQVFRKV----------------------------------h

d1exsa_ vevtPIMTeLDTQKVAGTWHTVAMAVSdVSLL-daKSSPLkAYVEGLKPTPEGDLEILLQKRENDKCAQEVLLAKKTdIPAVFKINALDENQLFLLDTdyDSHLLLCMENS--ASPEhsLVCQSLARTLEVDDQIREKFEDALKtlSVPM---RILPAQLEEQCR---v

d1gm6a_ ---vVTSN-FDASKIAGEWYSILLASD-AKENieeNGSMR-VFVEHIRVLDNSSLAFKFQRKVNGECTDFYAVCDKV-GDGVYTVAYYGENKFRLLEVnySDYVILHLVDVngDKTF--QLMEFYGRKPDVEPKLKDKFVEICQ--QYGIikeNIIDLTKIDRCFqlrg

d1exsa_ -----vevTPIMTELDTQKVAGTWHTVAMAVsdvsLLDAksSPLKAYVEGLKPTPEGDLEILLQKREN-----DKCAQEVLLAKKTDIPAVFKINA--------ldeNQLFLLDTDyDSHLLLCMENSA----SPEHsLVCQSLARTLEV-DDQIREKFEDALKTLSVPMRILPAQLEEQCrv

d1hbqa_ erdcrvssFRVKENFDKARFAGTWYAMAKKD----PEGL--FLQDNIVAEFSVDENGQMSATAKGRVRllnnwDVCADMVGTFTDTEDPAKFKMKYwgvasflqkgnDDHWIIDTDyETFAVQYSCRLLnldgTCAD-SYSFVFARDPSGfSPEVQKIVRQRQEELCLARQYRLIPHNGYCng

d1exsa_ --vevTPIM-TELDTQKVAGTWHTVAMAVSDvslldaksspLKAYVEGLKPTpEGDLEILLQKRENDKCAQEVLLAKKTDIPAVFKINA---lDENQLFLLDTdyDSHLLLCMENSAspehSLVCQSLARTLEVDDQIREKFEDALKTLSVPM-RILPAQLEEQC----------rv

d1iw2a_ aspisTIQPkANFDAQQFAGTWLLVAVGSAG---------rAEATTLHVAPQ-GTAMAVSTFRKLDGICWQVRQLYGDTGVLGRFLLQArgarGAVHVVVAETdyQSFAVLYLERAG----QLSVKLYARSLPVSDSVLSGFEQRVQEAHLTEdQIFYFPKYGFCeaadqfhvldev

d1exsa_ ----vevTPIM-TELDTQKVAGTWHTVAMAVsdvSLLDaksSPLKaYVEGLKPTPEGDLEILLQKRE-----NDKCAQEVLLAKKTDIPAVFKINA--------LDENQLFLLDTDyDSHLLLCMENSA----SPEHsLVCQSLARTLEV-DDQIREKFEDALKTLSVPMRILPaQLEEQCrv

d1jyda_ erdcrvsSFRVkENFDKARFSGTWYAMAKKD---PEGL---FLQDnIVAEFSVDETGQMSATAKGRVrllnnWDVCADMVGTFTDTEDPAKFKMKYwgvasflqKGNDDHWIVDTDyDTYAVQYSCRLLnldgTCAD-SYSFVFSRDPNGlPPEAQKIVRQRQEELCLARQYRLiVHNGYC--

d1exsa_ vevtpimteldtqKVAGTWHTVAMAV--sdvsLLDAK-----ssplkaYVEGLKPTPeGDLEILLQKRendkCAQEVLLAKK-------------------TDIP---AVFKI-naldenQLFLLDTdyDSHLLLCMENsaspEHSLVCQSLARTlevddqirekfedalktlsvpmrilpaqleeqcrv

d1kqxa_ -----------paDFNGTWEMLSNDNfedvmkALDIDfatrkiavhlkQTKVIVQNG-DKFETKTLST----FRNYEVNFVIgeefdeqtkgldnrtvktlVKWDgdkLVCVQkgekenrGWKQWIE--GDLLHLEIHC----QDKVCHQVFKKK----------------------------------n

d1exsa_ vevtpimteldTQKVAGTWHTVAMA-------vsdvslldakssplkAYVEGLKPTPeGDLEILLQKRendkCAQEVLLAK---------------------ktdipavfkinaldenqlFLLDTdyDSHLLLCMEnsasPEHSLVCQSLARTlevddqirekfedalktlsvpmrilpaqleeqcrv

d1liba_ -----------CDAFVGTWKLVSSEnfddymkevgvgfatrkvagmaKPNMIISVNG-DLVTIRSEST----FKNTEISFKlgvefdeitaddrkvksiitldggalvqvqkwdgksttiKRKRD--GDKLVVECV----MKGVTSTRVYERA-----------------------------------

d1exsa_ vevtpimteldtqKVAGTWHTVAMAVsdvSLLD--------AKSSplKAYVEGLKPTPeGDLEILLQKREndkcAQEVLL--------------------akKTDIP--AVFK-inaldeNQLFLLDTdyDSHLLLCMENSAspehSLVCQSLARTlevddqirekfedalktlsvpmrilpaqleeqcrv

d1lpja_ -----------paDLSGTWTLLSSDN-feGYMLalgidfatRKIAklLKPQKVIEQNG-DSFTIHTNSSL----RNYFVKfkvgeefdednrgldnrkckslVIWDNdrLTCIqkgekknRGWTHWIE--GDKLHLEMFCEG----QVCKQTFQRA-----------------------------------

d1exsa_ --------------vevtPIMT--ELDTQKVAGTWHTVAMAVsdvslldakSSPLK--aYVEGLKPTPeGDLEILLQKRE-NDKCAQEVLLAKKT--DIPAvFKINA--ldeNQLFLLDTDyDSHLLLCMENSA-speHSLVCQSLARTLEVDDQIREKFEDALKTLSVPM-RILP--aqLEEQC----rv

d1obqa_ dkipdfvvpgkcasvdrnKLWAeqTPNRNSYAGVWYQFALTN--------nPYQLIekcVRNEYSFDG-KQFVIESTGIAyDGNLLKRNGKLYPNpfGEPH-LSIDYensfaAPLVILETDySNYACLYSCIDYnfgyHSDFSFIFSRSANLADQYVKKCEAAFKNINVDTtRFVKtvqgSSCPYdtqktl

d1exsa_ -----VEVT---------piMTELDTQKVAGTWHTVA---------------------mavsdvslldaksspLKAYVEGLKPtpegdLEILLQKRendkCAQEVLLAKKTDIP------------AVFKIN---aldENQLFLLDT----dyDSHLLLCMENSA---spehsLVCQSLARTLE----VDDQIREKfedalktlsvpmrilpaqleeqcrv

d1oeja_ gkpltEVEQkaangvfddanVQNRTLSDWDGVWQSVYpllqsgkldpvfqkkadadktktfaeikdyyhkgyaTDIEMIGIED-----GIVEFHRN----NETTSCKYDYDGYKiltyksgkkgvrYLFECKdpeskaPKYIQFSDHiiaprkSSHFHIFMGNDSqqsllnemENWPTYYPYQLsseeVVEEMMSH-------------------------

d1exsa_ vevtpimteldtqKVAGTWHTVAMAV-sdvsLLDAK------ssplkaYVEGLKPTPeGDLEILLQKRendkCAQEVLLAKK------------------TDIP----AVFK-inaldeNQLFLLDTdyDSHLLLCMENsaspEHSLVCQSLARTlevddqirekfedalktlsvpmrilpaqleeqcrv

d1opaa_ -----------tkDQNGTWEMESNENfegymKALDIdfatrkiavrltQTKIIVQDG-DNFKTKTNST----FRNYDLDFTVgvefdehtkgldgrnvktLVTWegntLVCVqkgekenRGWKQWVE--GDKLYLELTC----GDQVCRQVFKKK-----------------------------------

d1exsa_ vevtpimteldtqKVAGTWHTVAMA-------vsdvslldakssplkaYVEGLKPTPeGDLEILLQKRendkCAQEVLL----------------akkTDIP----AVFKinaldeNQLFLLDTdyDSHLLLCMENSaspeHSLVCQSLARTlevddqirekfedalktlsvpmrilpaqleeqcrv

d1p6pa_ -------------AFNGTWNVYAQEnyenflrtvglpediikvakdvnPVIEIEQNG-NEFVVTSKTP----KQTHSNSftvgkeseitsmdgkkikvTVQLeggkLICK---sdkFSHIQEVN--GDEMVEKITIG----SSTLTRKSKRV-----------------------------------

d1exsa_ vevTPIM---------------TELDTQKVAGTWHTVAMAVsdvslldaksSPLK---aYVEGLKPTPEGDLEILLQKRE--NDKCAQEVLLAKKTDIP--AVFKINAL--DENQLFLLDTDY-DSHLLLCMEnsaspehsLVCQSLARTLEVDDQIREKFEDALKTLSVPM-RILPAQLEeqcrv

d1qwda_ hleSTSLykkssstpprgvtvvNNFDAKRYLGTWYEIARFD---------hRFERglekVTATYSLRDDGGLNVINKGYNpdRGMWQQSEGKAYFTGAPtrAALKVSFFgpFYGGYNVIALDReYRHALVCGP------drDYLWILSRTPTISDEVKQEMLAVATREGFDVsKFIWVQQP----g

d1exsa_ vevtpimteldtqkVAGTWHTvAMAVSDVslldaksspLKAYVEGLKPTPeGDLEILLQKRENDKCAQEVLLAKktdipAVFKI-------------------------------naldenqLFLLDtdydshLLLCMENSA-spEHSLVCQSLARTLevddqirekfedalktlsvpmrilpaqleeqcrv

d1r0ua_ ------gfqsnamkQETPITL-HVKSVIE-ddgnqeviEFRTTGFYYVKQ-NKVYLSYYEEHDLGKVKTIVKVS-----EGEVLvmrsgavkmnqrfvtgastiakykmsfgelelktstksIQSDL--deekGRISIAYDMhvgHLHNMTITYEGGT----------------------------------

d1exsa_ vevtpimteldtqKVAGTWHTVAMAVsdvSLLD--------AKSSpLKAYVEGLKPTPeGDLEILLQKRendkCAQEVLLAKK---------------TDIP-----AVFKinaldeNQLFLLDTdyDSHLLLCMENsaspEHSLVCQSLARTlevddqirekfedalktlsvpmrilpaqleeqcrv

d1tvqa_ -------------AFSGTWQVYAQEN-yeEFLKalalpedlIKMArDIKPIVEIQQKG-DDFVVTSKTP----RQTVTNSFTLgkeadittmdgkklkCTVHlangkLVTK---sekFSHEQEVK--GNEMVETITF----GGVTLIRRSKRV-----------------------------------

d1exsa_ -------------------------------------------------------------------------------------------------------------------------------------------------vevtpimtELDTqKVAGTWHTVAM--------------avsdvsllDAKSS---PLKAYVEGLKPTPeGDLEILLQKREndkCAQEVLLAKKT---------------------dipAVFKIN---aldeNQLFLLDTDY--dSHLLLCMENsaspEHSLVCQSLART------------levddqirekfedalktlsvpmrilpaqleeqcrv

d1vpra1 ekgfeagdnklggalnakhvekygdnfkngxhkpefhedglhkpxevggkkfesgfhyllechelggknasggyggplcedpygsevqaxtekllkeadsdrtlcfnnfqdpcpqltkeqvaxckgfdygdktlklpcgplpwpaglpepgyvPKTN-PLHGRWITVSGgqaafikeaiksgmlgaaeankIVADTdhhQTGGXYLRINQFG-DVCTVDASVAK---FARAKRTWKSGhyfyeplvsggnllgvwvlpeeyrKIGFFWexesgrcFRIERRAFPVgpyTFXRQATEV----GGKISFVFYVKVsndpesdpiplqsrdytalagrdnaptnlgkpyptlakdldypkkrd

d1exsa_ vevtpimteldtQKVAGTWHTVAMAV---SDVSL-----lDAKSS-pLKAYVEGLKPTPeGDLEILLQKRendkCAQEVLLAKK----------------TDIP-----AVFK----inaldeNQLFLLDTdYDSHLLLCMENSAspehSLVCQSLARTlevddqirekfedalktlsvpmrilpaqleeqcrv

d1xcaa_ ------------PNFSGNWKIIRSENfeeLLKVLgvnvmlRKIAVaaASKPAVEIKQEG-DTFYIKTSTT----VRTTEINFKVgeefeeqtvdgrpcksLVKWesenkMVCEqkllkgegpkTSWTMELT-NDGELILTMTADD----VVCTRVYVRE-----------------------------------

d1exsa_ vevtpimteldtqKVAGTWHTVAMAVsdvslldakSSPLkAYVEGLKPTPEGDLEILLQKreNDKCAQEVLLAKKTDIPAVFKINaldeNQLFLLDTDYDSHLLLCMENSASPehSLVCQSLARTLEVDDQIREKFEDALKTLSV-PMRILpAQLEEqcrv

d1xkia_ -------------DVSGTWYLKAMTV---------NLES-VTPMTLTTLEGGNLEAKVTM--SGRCQEVKAVLEKTDEPGKYTAD-ggkHVAYIIRSHVKDHYIFYSEGEGKP--VRGVKLVGRDPKNNLEALEDFEKAAGARGLsTESIL-IPRQS----

d1exsa_ vevtPIMTELDTQKVAGTWHTVAMAVSdVSLL--daKSSPLkAYVEGLKPTPEGDLEILLQKRENDKCAQEVLLAKKTDIPAVFKINALDENQLFLLDTDyDSHLLLCMENSASPEHSLVCQSLARTLEVDDQIREKFEDALKTLsVPMRILPAQLE--EQCR-v

d1yupa1 iivtQTMKDLDVQKVAGTWYSLAMAAS-DISLldaqSAPLR-VYVEELKPTPGGDLEILLQKWENGKCAQKKIIAEKTEIPAVFKIDALNENKVLVLDTDyKKYLLFCMENSAEPEQSLACQCLVRTPEVDDEAMEKFDKALKAL-PMHIRLSFNPTqlEEQCrv

d1exsa_ vevtpimtelDTQKVAGTWHTvAMAVSDVSLldaksspLKAYVEGLKptpegdlEILLQKR--eNDKC-AQEVLL---------------------aKKTD-----ipAVFKI------naldeNQLFLLDTDydSHLLLCMENS----ASPEhsLVCQSLARTlevddqirekfedalktlsvpmrilpaqleeqcrv

d2a13a1 --ppvhpfvaPLSYLLGTWRG-QGEGEYPTI----psfRYGEEIRFShsgkpviAYTQKTWkleSGAPxHAESGYfrprpdgsievviaqstglvevQKGTynvdeqsIKLKSdlvgnaskvkeISREFELVD--GKLSYVVRXStttnPLQP--HLKAILDKL-----------------------------------

d1exsa_ vevtpimteldtQKVAGTWHTVAMAVSDV--sLLDAK-----ssplkaYVEGLKPTPeGDLEILLQKRendkCAQEVLLAKK---------------TDIP------AVFKinaldeNQLFLLDTdyDSHLLLCMENsaspEHSLVCQSLARTlevddqirekfedalktlsvpmrilpaqleeqcrv

d2f73a1 ------------MSFSGKYQLQSQENFEAfmkAIGLPeeliqkgkdikGVSEIVQNG-KHFKFTITAG----SKVIQNEFTVgeeceletmtgekvkTVVQlegdnkLVTT---fknIKSVTELN--GDIITNTMTL----GDIVFKRISKRI-----------------------------------

d1exsa_ vevtpimteLDTQKVAGTWHTvAMAVSdvSLLDAksspLKAYvEGLKPT--PEGDlEILLQKRENDKCA-QEVLLAK--------------------kTDIP---------AVFK------------inaldeNQLFLLDTdyDSHLLLCMENS---ASPEhSLVCQSLARTlevddqirekfedalktlsvpmrilpaqleeqcrv

d2fr2a1 ---dlapalQALSPLLGSWAG-RGAGK--YPTIR--pfEYLE-EVVFAHvgKPFLtYTQQTRAVADGKPlHSETGYLrvcrpgcvelvlahpsgiteiEVGTysvtgdvieLELStradgsiglaptakevtaLDRSYRID--GDELSYSLQMRavgQPLQ-DHLAAVLHRQ----------------------------------r

d1exsa_ -vevtpimtELDTQkvAGTWHTVAmavsdvslldakssplkaYVEGLKPTPEGDLEILLQKrendkcAQEVLL-AKKT-------dipaVFKINAldENQLFLLDTDYDSHLLLCMENSAS----------------------------pEHSLVCQSLART-----levddqirekfedalktlsvpmrilpaqleeqcrv

d2gc9a1 xtktfktldDFLGT--HFIYTYDN-----------------gWEYEWYAKNDHTVDYRIHG---gxvAGRWVTdQKADivxltegiykiSWTEPT--GTDVALDFXPNEKKLHGTIFFPKWveehpeitvtyqnehidlxeqsrekyatyPKLVVPEFANITyxgdagqnnedviseapykexpndirngkyfdqnyhrlnk

d1exsa_ vevtpIMTELDTQKVAGTWHTvAMAVSDVslldaksspLKAYVeGLKPTP--egdlEILLQKrendkcAQEVLL-----------------aKKTD----ipAVFK----inaldeNQLFLLDTDyDSHLLLCMENSA--spEHSLVCQSLARTlevddqirekfedalktlsvpmrilpaqleeqcrv

d2o62a1 -----ERPLLQINDLLGEWRG-QAVTIYR---dlrppdIYSTT-LKIQLDdagrlxQSTSFG------ERTITStatikgsivlfdqdpekqVQVLllpdgaSATSplkvqlrqplFLEAGWLIQ-SDLRQRXIRSYNdkgeWVSLTLVTEERV-----------------------------------

d1exsa_ ---vevtpiMTELdTQKVA--GTWHTVAMAVSdVSLLDAkssplKAYVeGLKPTPEgDLEILLQKRE--NDKCAQEVLLAKKTdIPAVFKINA---------------ldeNQLFLLDTDyDSHLLLCMENSA-spehSLVCQSLARTLEVDdqIREKFEDALKTLSVPM-RILP-AQLEEQCRV---------

d2ofmx1 actknaiaqTGFN-KDKYFngDVWYVTDYLDL-EPDDVP--kryCAAL-AAGTASG-KLKEALYHYDpkTQDTFYDVSELQVE-SLGKYTANFkkvdkngnvkvavtagnyYTFTVMYAD-DSSALIHTCLHKgnkdlGDLYAVLNRNKDAA--AGDKVKSAVSAATLEFsKFIStKENNCAYDNdslkslltk

d1exsa_ vevtpiMTELDTQKVAGTWHTVAMAVSdVSLL-daKSSPLkAYVEGLKPTpEGDLEILLQKRENDKCAQEVLLAKKTDIPAVFKINALDENQLFLLDTDyDSHLLLCMENS--ASPEhsLVCQSLARTLEVDDQIREKFEDALKTLSVPM-RILPaQLEEQCrv

d2ozqa1 eeasstGRNFNVEKINGEWHTIILASD-KREKiedNGNFR-LFLEQIHVL-EKSLVLKFHTVRDEECSELSMVADKTEKAGEYSVTYDGFNTFTIPKTDyDNFLMAHLINEkdGETF--QLMGLYGREPDLSSDIKERFAQLCEEHGILReNIIDlSNANRC--

d1exsa_ ---vevtpiMTELdTQKVA--GTWHTVAMAVsdvSLLDAkssplKAYVEGLKPTPEgDLEILLQKRE--NDKCAQEVLLAKKTdIPAVFKINA---------------ldENQLFLLDTDyDSHLLLCMENS---ASPEhsLVCQSLARTLEVDdqIREKFEDALKTLSVPM-RILP-AQLEEQCRV---------

d3np1a_ kctknalaqTGFN-KDKYFngDVWYVTDYLD---LEPDD-vpkrYCAALAAGTASG-KLKEALYHYDpkTQDTFYDVSELQEE-SPGKYTANFkkvekngnvkvdvtsgnYYTFTVMYAD-DSSALIHTCLHkgnKDLG--DLYAVLNRNKDTN--AGDKVKGAVTAASLKFsDFIStKDNKCEYDNvslkslltk

d1ftpa_ -----------vKEFAgikykldsqtnfeeymkaigvgaierkaglalspvieleildgdkfkltsktaiknteftfklgeefdeeTLDGRKVKSTIT-qDGPNKLVHEQKG--DHPTIIIREFSK------EQCVITIKLG-DLVATRIYKAQ--------------

d1avgi_ aegddcsiekamGDFK--------------------------peeffngtwylahgpgvtspavcqkfttsgskgftqiveigynkFESNVKFQCNQVdnKNGEQYSFKCKSsdNTEFEADFTFISvsydnfALVCRSITFTsQPKEDRYLVFErtksdtdpdakeic

d1ftpa_ VKEFAgIKYKLDSQTNFEEYMKAIGVGAIERKAGLALSPVIELEILdGDKFKLTSKTAIKNTEFTFKLGEEFDEET--LDGRKVKSTITQDGPnKLVHEQKGDH-PTIIIREFSKEQCVITIKLGDLVATRIYKAQ-

d1ggla_ PPNLT-GYYRFVSQKNMEDYLQALNISLAVRKIALLLKPDKEIEHQ-GNHMTVRTLSTFRNYTVQFDVGVEFEEDLrsVDGRKCQTIVTWEEE-HLVCVQKGEVpNRGWRHWLEGEMLYLELTARDAVCEQVFRKVh

d1ftpa_ VKEFAgIKYKLDSQTNFEEYMKAIGVGAIERKAGLALSPVIELEILdGDKFKLTSKTAIKNTEFTFKLGEEFDEET--LDGRKVKSTITQDGPnKLVHEQKGDH-PTIIIREFSKEQCVITIKLGDLVATRIYKAQ-

d1kqxa_ PADFN-GTWEMLSNDNFEDVMKALDIDFATRKIAVHLKQTKVIVQN-GDKFETKTLSTFRNYEVNFVIGEEFDEQTkgLDNRTVKTLVKWDGD-KLVCVQKGEKeNRGWKQWIEGDLLHLEIHCQDKVCHQVFKKKn

d1ftpa_ VKEFAgIKYKLDSQTNFEEYMKAIGVGAIERKAGLALSPVIELEILDgDKFKLTSKTAIKNTEFTFKLGEEFDEETLDGRKVKSTITQDGPnKLVHEQKGD-HPTIIIREFSKEQCVITIKLGDLVATRIYKAQ

d1liba_ CDAFV-GTWKLVSSENFDDYMKEVGVGFATRKVAGMAKPNMIISVNG-DLVTIRSESTFKNTEISFKLGVEFDEITADDRKVKSIITLDGG-ALVQVQKWDgKSTTIKRKRDGDKLVVECVMKGVTSTRVYERA

d1ftpa_ VKEFAgIKYKLDSQTNFEEYMKAIGVGAIERKAGLALSPVIELEILdGDKFKLTSKTAIKNTEFTFKLGEEFDEET--LDGRKVKSTITQDGPnKLVHEQKGDH-PTIIIREFSKEQCVITIKLGDLVATRIYKAQ

d1lpja_ PADLS-GTWTLLSSDNFEGYMLALGIDFATRKIAKLLKPQKVIEQN-GDSFTIHTNSSLRNYFVKFKVGEEFDEDNrgLDNRKCKSLVIWDND-RLTCIQKGEKkNRGWTHWIEGDKLHLEMFCEGQVCKQTFQRA

d1ftpa_ -------------------------vKEFAgIKYKLDS------------------------------QTNFeeymkaigvgaierkaglaLSPVIELEIldgdkFKLTSKTAIKNTEFTfklgeefdeetldgrkvkSTITQDG--------PNKLVHEQKGD---hpTIIIREF------sKEQCVITIKLG-------dlvATRIYKAQ--------------

d1oeja_ gkplteveqkaangvfddanvqnrtlSDWD-GVWQSVYpllqsgkldpvfqkkadadktktfaeikdyYHKG----------------yatDIEMIGIED-----GIVEFHRNNETTSCK-----------------yDYDGYKIltyksgkkGVRYLFECKDPeskapKYIQFSDhiiaprkSSHFHIFMGNDsqqsllnemeNWPTYYPYqlsseevveemmsh

d1ftpa_ VKEFAgIKYKLDSQTNFEEYMKAIGVGAIERKAGLALSPVIELEILdGDKFKLTSKTAIKNTEFTFKLGEEFDEET--LDGRKVKSTITQDGPnKLVHEQKGDH-PTIIIREFSKEQCVITIKLGDLVATRIYKAQ

d1opaa_ TKDQN-GTWEMESNENFEGYMKALDIDFATRKIAVRLTQTKIIVQD-GDNFKTKTNSTFRNYDLDFTVGVEFDEHTkgLDGRNVKTLVTWEGN-TLVCVQKGEKeNRGWKQWVEGDKLYLELTCGDQVCRQVFKKK

d1ftpa_ vkEFAGiKYKLDSQTNFEEYMKAIGVGAIERKAGLALSPVIELEILdGDKFKLTSKTAIKNTEFTFKLGEEFDEETLDGRKVKSTITQDgPNKLVHEQKgdhPTIIIREFSKEQCVITIKLGDLVATRIYKAQ

d1p6pa_ --AFNG-TWNVYAQENYENFLRTVGLPEDIIKVAKDVNPVIEIEQN-GNEFVVTSKTPKQTHSNSFTVGKESEITSMDGKKIKVTVQLE-GGKLICKSD---KFSHIQEVNGDEMVEKITIGSSTLTRKSKRV

d1ftpa_ vkefagikykldsqtnfeeymkaigvgaierkaglalspvieleildgdkfkltsktaiknteftfklgeefdeetldgrkvksTITQDGP---NKLVHEQKGD-HPTIIIREFSKEQCVITIKLGdLVATRIYKAQ--------------------------------------------------------

d1r0ua_ --------------------------------------------------gfqsnamkqetpitlhvksvieddgnqeviefrtTGFYYVKqnkVYLSYYEEHDlGKVKTIVKVSEGEVLVMRSGA-VKMNQRFVTGastiakykmsfgelelktstksiqsdldeekgrisiaydmhvghlhnmtityeggt

d1ftpa_ vkEFAGiKYKLDSQTNFEEYMKAIGVGAIERKAGLALSPVIELEILdGDKFKLTSKTAIKNTEFTFKLGEEFDEET-LDGRkVKSTITQDGPnKLVHEQKgdhPTIIIREFSKEQCVITIKLGDLVATRIYKAQ

d1tvqa_ --AFSG-TWQVYAQENYEEFLKALALPEDLIKMARDIKPIVEIQQK-GDDFVVTSKTPRQTVTNSFTLGKEADITTmDGKK-LKCTVHLANG-KLVTKSE---KFSHEQEVKGNEMVETITFGGVTLIRRSKRV

d1ftpa_ -----------------------------------------------------------------------------------------------------------------------------------------------------------vKEFAGiKYKLDSQ-------tnfeeymkaigvGAIER---kaglalSPVIELEILdgDKFKLTSKTA-iknTEFTFKLGEEFDEETLDGRKVKSTITQDGPNKLVHEQKG---DHPTIIIREFSK----EQCVITIKLGDLVATRIYKAQ-----------------------------------------------

d1vpra1 ekgfeagdnklggalnakhvekygdnfkngxhkpefhedglhkpxevggkkfesgfhyllechelggknasggyggplcedpygsevqaxtekllkeadsdrtlcfnnfqdpcpqltkeqvaxckgfdygdktlklpcgplpwpaglpepgyvpktNPLHG-RWITVSGgqaafikeaiksgmlgaaeANKIVadtdhhqtgGXYLRINQF-gDVCTVDASVAkfarAKRTWKSGHYFYEPLVSGGNLLGVWVLPEEYRKIGFFWExesGRCFRIERRAFPvgpyTFXRQATEVGGKISFVFYVKVsndpesdpiplqsrdytalagrdnaptnlgkpyptlakdldypkkrd

d1ftpa_ vKEFAgIKYKLDSQTNFEEYMKAIGVGAIERKAGLAL--SPVIELEILdGDKFKLTSKTAIKNTEFTFKLGEEFDEETLDGRKVKSTITQDGPNKLVHEQKG----DHPTIIIREFS-KEQCVITIKLGDLVATRIYKAQ

d1xcaa_ -PNFS-GNWKIIRSENFEELLKVLGVNVMLRKIAVAAasKPAVEIKQE-GDTFYIKTSTTVRTTEINFKVGEEFEEQTVDGRPCKSLVKWESENKMVCEQKLlkgeGPKTSWTMELTnDGELILTMTADDVVCTRVYVRE

d1ftpa_ ---------VKEFAGiKYKLdSQTNfeEYMKaigvgaierkagLALSPVIELEILDGDKFKLTSKT-------AIKNTEFTFKL----GEEFDEETLDGRKVKSTITQDG-PNKLVHEQKG------DHPTIIIREFSKEQCVITIKL------GDLVATRIYKAQ

d2a13a1 ppvhpfvapLSYLLG-TWRG-QGEG--EYPT---------ipsFRYGEEIRFSHSGKPVIAYTQKTwklesgaPXHAESGYFRPrpdgSIEVVIAQSTGLVEVQKGTYNVdEQSIKLKSDLvgnaskVKEISREFELVDGKLSYVVRXstttnpLQPHLKAILDKL

d1ftpa_ vKEFAgIKYKLDSQTNFEEYMKAIGVGAIERKAGLALSPVIELEILdGDKFKLTSKtaIKNTEFTFKLGEEFDEETLDGRKVKSTITQDGPNKLVHEQKgdhPTIIIREFSKEQCVITIKLGDLVATRIYKAQ

d2f73a1 -MSFS-GKYQLQSQENFEAFMKAIGLPEELIQKGKDIKGVSEIVQN-GKHFKFTITagSKVIQNEFTVGEECELETMTGEKVKTVVQLEGDNKLVTTFK---NIKSVTELNGDIITNTMTLGDIVFKRISKRI

d1ftpa_ --------VKEFAGiKYKLdSQTNfeeYMKAigvgaierkagLALSPVIELEILDGDKFKLTSKT-------AIKNTEFTFKL----GEEFDEETLDGRKVKSTITQDG---PNKLVHEQK------------gDHPTIIIREFSKEQCVITIKL------GDLVATRIYKAQ-

d2fr2a1 dlapalqaLSPLLG-SWAG-RGAG---KYPT--------irpFEYLEEVVFAHVGKPFLTYTQQTravadgkPLHSETGYLRVcrpgCVELVLAHPSGITEIEVGTYSVtgdVIELELSTRadgsiglaptakeVTALDRSYRIDGDELSYSLQMravgqpLQDHLAAVLHRQr

d1ftpa_ ----------VKEFaGIKYKLDSqtnfeeymkaigvgaierkaglalSPVIELEILdgdKFKLTSKT-aiKNTEFTFklgeefdeetldgrkvksTITQDGP----NKLVHEQKgdHPTIIIREFSK--EQCVITIKLG--------------------------------DLVATRIYKAQ----------------------------------------

d2gc9a1 xtktfktlddFLGT-HFIYTYDN------------------------GWEYEWYAKndhTVDYRIHGgxvAGRWVTD-----------------qKADIVXLtegiYKISWTEP--TGTDVALDFXPneKKLHGTIFFPkwveehpeitvtyqnehidlxeqsrekyatypKLVVPEFANITyxgdagqnnedviseapykexpndirngkyfdqnyhrlnk

d1ftpa_ ------VKEFAGiKYKLdSQTNFEEymkaigvgaierkagLALSPVIELEILDGDKFKLTSKTAIKNTEFTFK----LGEEFDEETldgrkvKSTITQDGPNKLVHEQKG----DHPTIIIREFS-KEQCVITIKLG------DLVATRIYKAQ

d2o62a1 erpllqINDLLG-EWRG-QAVTIYR----------dlrppDIYSTTLKIQLDDAGRLXQSTSFGERTITSTATikgsIVLFDQDPE-----kQVQVLLLPDGASATSPLKvqlrQPLFLEAGWLIqSDLRQRXIRSYndkgewVSLTLVTEERV

d1ggla_ -----------PPNLTgyyrfvsqknmedylqalnislavrkialllkpdkeieHQGNHMTVRTL-STFRN---ytvqfDVGV-----efeedlrSVDG--RKCQTIVTW--EEEHLVCVQKG-EVPNRGWRHWLEGE------MLYLELTARD-AVCEQVFR-------------kvh

d1avgi_ aegddcsiekaMGDFK-------------------------------------pEEFFNGTWYLAhGPGVTspavcqkfTTSGskgftqiveigyNKFEsnVKFQCNQVDnkNGEQYSFKCKSsDNTEFEADFTFISVsydnfaLVCRSITFTSqPKEDRYLVfertksdtdpdakeic

d1ggla_ pPNLTGYYRFVSQKNMEDYLQALNISLAVRKIALLLKPDKEIEHQGNHMTVRTLSTFRNYTVQFDVGVEFEEDLRSVDGRKCQTIVTWEEEHLVCVQKGEVPNRGWRHWLEGEMLYLELTARDAVCEQVFRKVH

d1kqxa_ pADFNGTWEMLSNDNFEDVMKALDIDFATRKIAVHLKQTKVIVQNGDKFETKTLSTFRNYEVNFVIGEEFDEQTKGLDNRTVKTLVKWDGDKLVCVQKGEKENRGWKQWIEGDLLHLEIHCQDKVCHQVFKKKN

d1ggla_ pPNLTGYYRFVSQKNMEDYLQALNISLAVRKIALLLKPDKEIEHQGNHMTVRTLSTFRNYTVQFDVGVEFEEDLrsVDGRKCQTIVTWEEEHLVCVQKGEVPNRGWRHWLEGEMLYLELTARDAVCEQVFRKVh

d1liba_ cDAFVGTWKLVSSENFDDYMKEVGVGFATRKVAGMAKPNMIISVNGDLVTIRSESTFKNTEISFKLGVEFDEIT--ADDRKVKSIITLDGGALVQVQKWDGKSTTIKRKRDGDKLVVECVMKGVTSTRVYERA-

d1ggla_ pPNLTGYYRFVSQKNMEDYLQALNISLAVRKIALLLKPDKEIEHQGNHMTVRTLSTFRNYTVQFDVGVEFEEDLRSVDGRKCQTIVTWEEEHLVCVQKGEVPNRGWRHWLEGEMLYLELTARDAVCEQVFRKVh

d1lpja_ pADLSGTWTLLSSDNFEGYMLALGIDFATRKIAKLLKPQKVIEQNGDSFTIHTNSSLRNYFVKFKVGEEFDEDNRGLDNRKCKSLVIWDNDRLTCIQKGEKKNRGWTHWIEGDKLHLEMFCEGQVCKQTFQRA-

d1ggla_ -------------------------ppNLTGYYRFVS----------------------------------QKnmedylqalnislavrkiallLK-PDKEIEHqgnhMTVRTLSTFRNYTVQfdvgvefeedlrsvdgrkcqtIVTWEE----------EHLVCVQK--gevpnRGWRHWLE------GEMLYLELTAR-------davCEQVFRKVH-------------

d1oeja_ gkplteveqkaangvfddanvqnrtlsDWDGVWQSVYpllqsgkldpvfqkkadadktktfaeikdyyhkgYA---------------------TDiEMIGIED----GIVEFHRNNETTSCK--------------------yDYDGYKiltyksgkkgVRYLFECKdpeskapKYIQFSDHiiaprkSSHFHIFMGNDsqqsllnemeNWPTYYPYQlsseevveemmsh

d1ggla_ pPNLTGYYRFVSQKNMEDYLQALNISLAVRKIALLLKPDKEIEHQGNHMTVRTLSTFRNYTVQFDVGVEFEEDLRSVDGRKCQTIVTWEEEHLVCVQKGEVPNRGWRHWLEGEMLYLELTARDAVCEQVFRKVh

d1opaa_ tKDQNGTWEMESNENFEGYMKALDIDFATRKIAVRLTQTKIIVQDGDNFKTKTNSTFRNYDLDFTVGVEFDEHTKGLDGRNVKTLVTWEGNTLVCVQKGEKENRGWKQWVEGDKLYLELTCGDQVCRQVFKKK-

d1ggla_ ppNLTGYYRFVSQKNMEDYLQALNISLAVRKIALLLKPDKEIEHQGNHMTVRTLSTFRNYTVQFDVGVEFEEDLrsVDGRKCQTIVTWEEEHLVCVQKgevpNRGWRHWLEGEMLYLELTARDAVCEQVFRKVh

d1p6pa_ --AFNGTWNVYAQENYENFLRTVGLPEDIIKVAKDVNPVIEIEQNGNEFVVTSKTPKQTHSNSFTVGKESEITS--MDGKKIKVTVQLEGGKLICKSD----KFSHIQEVNGDEMVEKITIGSSTLTRKSKRV-

d1ggla_ -----------------------------------------pPNLTGYYRFvsqknmedylqalnislavrkiALLLKPDKEIEHQGNHMTVRTLStfrNYTVQFDVGVEFEEDLRS-vDGRKCQTIVTW---------eeeHLVCVQKGEvpnRGWRHWLEGEmlyleltardavceqvfrkvh

d1r0ua_ gfqsnamkqetpitlhvksvieddgnqeviefrttgfyyvkqNKVYLSYYE---------------------eHDLGKVKTIVKVSEGEVLVMRSG-avKMNQRFVTGASTIAKYKMsfGELELKTSTKSiqsdldeekgriSIAYDMHVG---HLHNMTITYE------------------ggt

d1ggla_ ppNLTGYYRFVSQKNMEDYLQALNISLAVRKIALLLKPDKEIEHQGNHMTVRTLSTFRNYTVQFDVGVEFEEDLrSVDGrKCQTIVTWEEEHLVCVQKgevpNRGWRHWLEGEMLYLELTARDAVCEQVFRKVh

d1tvqa_ --AFSGTWQVYAQENYEEFLKALALPEDLIKMARDIKPIVEIQQKGDDFVVTSKTPRQTVTNSFTLGKEADITT-MDGK-KLKCTVHLANGKLVTKSE----KFSHEQEVKGNEMVETITFGGVTLIRRSKRV-

d1ggla_ -----------------------------------------------------------------------------------------------------------------------------------------------------------pPNLTGYYRFVSQ------knmedylqalniSLAVRK----ialllKPDKEIEHQGNHMTVRTLST-frnYTVQFDVGVEFEEDLrsVDGRKCQTIVTWEEE-HLVCVQKGE--VPNRGWRHWLEG----EMLYLELTARDAVCEQVFRKVH----------------------------------------------

d1vpra1 ekgfeagdnklggalnakhvekygdnfkngxhkpefhedglhkpxevggkkfesgfhyllechelggknasggyggplcedpygsevqaxtekllkeadsdrtlcfnnfqdpcpqltkeqvaxckgfdygdktlklpcgplpwpaglpepgyvpktNPLHGRWITVSGgqaafikeaiksgmlgaaEANKIVadtdhhqtgGXYLRINQFGDVCTVDASVAkfarAKRTWKSGHYFYEPL--VSGGNLLGVWVLPEEyRKIGFFWEXesGRCFRIERRAFPvgpyTFXRQATEVGGKISFVFYVKVSndpesdpiplqsrdytalagrdnaptnlgkpyptlakdldypkkrd

d1ggla_ pPNLTGYYRFVSQKNMEDYLQALNISLAVRKIALL--LKPDKEIEHQGNHMTVRTLSTFRNYTVQFDVGVEFEEDLrsVDGRKCQTIVTWEEE-HLVCVQKG---EVPNRGWRHWLE-GEMLYLELTARDAVCEQVFRKVh

d1xcaa_ -PNFSGNWKIIRSENFEELLKVLGVNVMLRKIAVAaaSKPAVEIKQEGDTFYIKTSTTVRTTEINFKVGEEFEEQT--VDGRPCKSLVKWESEnKMVCEQKLlkgEGPKTSWTMELTnDGELILTMTADDVVCTRVYVRE-

d1ggla_ ---------pPNLTGYYRfVSQKnmedylqalnislavrkiallLKPDKEIEHQ-GNHMTVRTLS-------TFRNYTVQFDV----GVEFEEDLrsVDGRKCQTIVTWEE--EHLVCVQKGE-----VPNRGWRHWLEGEMLYLELTAR-----DAVC-EQVFRKVh

d2a13a1 ppvhpfvaplSYLLGTWR-GQGE-----------geyptipsfrYGEEIRFSHSgKPVIAYTQKTwklesgaPXHAESGYFRPrpdgSIEVVIAQ--STGLVEVQKGTYNVdeQSIKLKSDLVgnaskVKEISREFELVDGKLSYVVRXStttnpLQPHlKAILDKL-

d1ggla_ pPNLTGYYRFVSQKNMEDYLQALNISLAVRKIALLLKPDKEIEHQGNHMTVRTLSTFRNYTVQFDVGVEFEEDLrsVDGRKCQTIVTWEEE-HLVCVQKgevpNRGWRHWLEGEMLYLELTARDAVCEQVFRKVh

d2f73a1 -MSFSGKYQLQSQENFEAFMKAIGLPEELIQKGKDIKGVSEIVQNGKHFKFTITAGSKVIQNEFTVGEECELET--MTGEKVKTVVQLEGDnKLVTTFK----NIKSVTELNGDIITNTMTLGDIVFKRISKRI-

d1ggla_ --------pPNLTGYYRFVSQKnmedylqalnislavrkiallLKPDKEIEHQ-GNHMTVRTLS-------TFRNYTVQFDV----GVEFEEDLrsVDGRKCQTIVTWEE----EHLVCV----QKGE-------VPNRGWRHWLEGEMLYLELTAR-----DAVC-EQVFRKVH

d2fr2a1 dlapalqalSPLLGSWAGRGAG------------kyptirpfeYLEEVVFAHVgKPFLTYTQQTravadgkPLHSETGYLRVcrpgCVELVLAH--PSGITEIEVGTYSVtgdvIELELStradGSIGlaptakeVTALDRSYRIDGDELSYSLQMRavgqpLQDHlAAVLHRQR

d1ggla_ ----------PPNLTGYYRFVSqknmedylqalnislavrkialllKPDKEIEHQGN-HMTVRTLS-tfrNYTVQFDVgvefeedlrsvdgrkcqtiVTWEEE-----HLVCVQKgevpNRGWRHWLE--GEMLYLELTAR--------------------------------DAVCEQVFRKVH---------------------------------------

d2gc9a1 xtktfktlddFLGTHFIYTYDN------------------------GWEYEWYAKNDhTVDYRIHGgxvaGRWVTDQK-------------------ADIVXLtegiyKISWTEP---tGTDVALDFXpnEKKLHGTIFFPkwveehpeitvtyqnehidlxeqsrekyatypKLVVPEFANITYxgdagqnnedviseapykexpndirngkyfdqnyhrlnk

d1ggla_ ------pPNLTGYYRFvSQKNMEdylqalnislavrkiaLLLKPDKEIEHQG-NHMTVRTLSTFRNYTVQFDV---GVEFEEDLRsvdgrkcQTIVTWEEE-HLVCVQKGE---VPNRGWRHWL-EGEMLYLELTAR------DAVCEQVFRKVh

d2o62a1 erpllqiNDLLGEWRG-QAVTIY----------rdlrppDIYSTTLKIQLDDaGRLXQSTSFGERTITSTATIkgsIVLFDQDPE------kQVQVLLLPDgASATSPLKVqlrQPLFLEAGWLiQSDLRQRXIRSYndkgewVSLTLVTEERV-

d1gm6a_ ---------vvTSNFDASKIA-GEWYsILLAsdakenIEENgsmrvfvehirVLDNsslAFKF----qrkvngeCTDFYAVCDK-VGDGVYTVAY------YGENKFRLLEVNYSDYVILHLVDVN-GDKTFQLMEFYGRKPDvepklkdkfveicqqygiikeniidlTKIDRCFQLRg

d1avgi_ aegddcsiekaMGDFKPEEFFnGTWY-LAHG----pgVTSP----avcqkftTSGS--kGFTQiveigynkfesNVKFQCNQVDnKNGEQYSFKCkssdntEFEADFTFISVSYDNFALVCRSITFtSQPKEDRYLVFERTKS--------------------------DTDPDAKEIC-

d1gm6a_ vvtsnfdaSKIAGEWYSILLA-SDAK-------eniEENG-SMRV-FVEHIRVLDnSSLAFKFQrkvngeCTDFYAVCD-----------------KVGD-----GVYT----vayyGENKFRLlEVNYsDYVILHLVDVngdkTFQLMEFYGRKpdvepklkdkfveicqqygiikeniidltkidrcfqlrg

d1cbia_ --------PNFAGTWKMRSSEnFDELlkalgvnamlRKVAvAAASkPHVEIRQDG-DQFYIKTS----ttVRTTEINFKvgegfeeetvdgrkcrsLPTWenenkIHCTqtllegdgPKTYWTR-ELAN-DELILTFGAD----DVVCTRIYVRE---------------------------------------

d1gm6a_ vvtsnfdASKIAG-EWYSILLAS--DAKE------niEENGsmRVFVEHIRVLDNSSLAFKFQRKvngeCTDFYAV------------------cDKVGD----GVYTVayyGENKFRLlEVNYsDYVILHLVDVngdkTFQLMEFYGRKpdvepklkdkfveicqqygiikeniidltkidrcfqlrg

d1ftpa_ -------VKEFAGiKYKLDSQTNfeEYMKaigvgaieRKAGlaLSPVIELEILDGDKFKLTSKTA----IKNTEFTfklgeefdeetldgrkvksTITQDgpnkLVHEQkgdHPTIIIR-EFSK-EQCVITIKLG----DLVATRIYKAQ---------------------------------------

d1gm6a_ vvtsnfdASKIAGEWYSILLAS--DAKE------niEENGSmRVFVEHIRVLDNSsLAFKFQRKvngeCTDFYAV--------------------cDKVGD---GVYT-vayyGENKFRLlEVNYsDYVILHLVDVngdkTFQLMEFYGRKpdvepklkdkfveicqqygiikeniidltkidrcfqlrg

d1ggla_ -------PPNLTGYYRFVSQKNmeDYLQalnislavRKIALlLKPDKEIEHQGNH-MTVRTLST----FRNYTVQfdvgvefeedlrsvdgrkcqtIVTWEeehLVCVqkgevPNRGWRH-WLEG-EMLYLELTAR----DAVCEQVFRKV--------------------------------------h

d1gm6a_ ---------vvTSNFDASKIAGEWYSILLASdAKENieengSMRV-FVEHIRVLDNSSLAFKFQRKVN-----GECTDFYAVCDKV-GDGVYTVAY--------YGENKFRLLEVNYSDYVILHLVDVNGD---kTFQLMEFYGRKP-DVEPKLKDKFVEICQQYGIIKeNIIDLTkIDRCFQLrg

d1hbqa_ erdcrvssfrvKENFDKARFAGTWYAMAKKD-PEGL-----FLQDnIVAEFSVDENGQMSATAKGRVRllnnwDVCADMVGTFTDTeDPAKFKMKYwgvasflqKGNDDHWIIDTDYETFAVQYSCRLLNLdgtcADSYSFVFARDPsGFSPEVQKIVRQRQEELCLAR-QYRLIP-HNGYCNG--

d1gm6a_ -------vvTSNFDASKIAGEWYSILLASDAkenieengsmrvFVEHIRVLdNSSLAFKFQRKVNGECTDFYAVCDKV-GDGVYTVAY---YGENKFRLLEVNYSDYVILHLVDVngdkTFQLMEFYGRKPDVEPKLKDKFVEICQQYGIIKENIIDLTKIDRCFQL-------rg

d1iw2a_ aspistiqpKANFDAQQFAGTWLLVAVGSAG--------raeaTTLHVAPQ-GTAMAVSTFRKLDGICWQVRQLYGDTgVLGRFLLQArgaRGAVHVVVAETDYQSFAVLYLERA----GQLSVKLYARSLPVSDSVLSGFEQRVQEAHLTEDQIFYFPKYGFCEAAdqfhvldev

d1gm6a_ ---------vvTSNFDASKIAGEWYSILLASdAKENieengSMRV-FVEHIRVLDNSSLAFKFQRKV-----NGECTDFYAVCDKV-GDGVYTVAY--------YGENKFRLLEVNYSDYVILHLVDVNGD---kTFQLMEFYGRKP-DVEPKLKDKFVEICQQYGIIKeNIIDLTkidRCFQLrg

d1jyda_ erdcrvssfrvKENFDKARFSGTWYAMAKKD-PEGL-----FLQDnIVAEFSVDETGQMSATAKGRVrllnnWDVCADMVGTFTDTeDPAKFKMKYwgvasflqKGNDDHWIVDTDYDTYAVQYSCRLLNLdgtcADSYSFVFSRDPnGLPPEAQKIVRQRQEELCLAR-QYRLIV---HNGYC--

d1gm6a_ vvtsnfdaSKIAGEWYSILLAS--DAKE------niEENGSmRVFVEHIRVLDnSSLAFKFQRKvngeCTDFYAV--------------------cdKVGD---GVYT-vayygeNKFRLlEVNYsDYVILHLVDVngdkTFQLMEFYGRKpdvepklkdkfveicqqygiikeniidltkidrcfqlrg

d1kqxa_ -------pADFNGTWEMLSNDNfeDVMKaldidfatRKIAVhLKQTKVIVQNG-DKFETKTLST----FRNYEVNfvigeefdeqtkgldnrtvktlVKWDgdkLVCVqkgekenRGWKQ-WIEG-DLLHLEIHCQ----DKVCHQVFKKK--------------------------------------n

d1gm6a_ vvtsnfdASKIAGEWYSILLAS--DAKE------niEENGSmrVFVEHIRVLDNSsLAFKFQRKvngeCTDFYAV------------------cDKVGD---GVYTVA-yyGENKFRLLEVNysDYVILHLVDVngdkTFQLMEFYGRKpdvepklkdkfveicqqygiikeniidltkidrcfqlrg

d1liba_ -------CDAFVGTWKLVSSENfdDYMKevgvgfatRKVAGmaKPNMIISVNGDL-VTIRSEST----FKNTEISfklgvefdeitaddrkvksIITLDggaLVQVQKwdgKSTTIKRKRDG--DKLVVECVMK----GVTSTRVYERA---------------------------------------

d1gm6a_ vvtsnfdaSKIAGEWYSILLAS--DAKE------niEENGSmRVFVEHIRVLDnSSLAFKFQRKvngeCTDFYAVCD--------------------KVGD---GVYT-vayygeNKFRLlEVNYsDYVILHLVDVngdkTFQLMEFYGRKpdvepklkdkfveicqqygiikeniidltkidrcfqlrg

d1lpja_ -------pADLSGTWTLLSSDNfeGYMLalgidfatRKIAKlLKPQKVIEQNG-DSFTIHTNSS----LRNYFVKFKvgeefdednrgldnrkckslVIWDndrLTCIqkgekknRGWTH-WIEG-DKLHLEMFCE----GQVCKQTFQRA---------------------------------------

d1gm6a_ ------------------vVTSNF--DASKIAGEWYSILLASdakenieenGSMRV---FVEHIRVLDnSSLAFKFQRKV-NGECTDFYAVCDKVG--dgVYTVAY--YGENKFRLLEVNYSDYVILHLVDVNG-DKTFQLMEFYGRKPDVEPKLKDKFVEICQQYGIIKENIIDLTKI-DRCF-QLRG-

d1obqa_ dkipdfvvpgkcasvdrnkLWAEQtpNRNSYAGVWYQFALTN--------nPYQLIekcVRNEYSFDG-KQFVIESTGIAyDGNLLKRNGKLYPNPfgepHLSIDYenSFAAPLVILETDYSNYACLYSCIDYNfGYHSDFSFIFSRSANLADQYVKKCEAAFKNINVDTTRFVKTVQGsSCPYdTQKTl

d1gm6a_ ------------------vvtSNFDASKIAGEWYSIL----------------------lasdakenieengsMRVFVEHIRVldnssLAFKFQRKvngeCTDFYAVCDKVGD-------------GVYTVAYY---geNKFRLLE----vnYSDYVILHLVDV---ngdktfqLMEFYGRKPD----VEPKLKDKfveicqqygiikeniidltkidrcfqlrg

d1oeja_ gkplteveqkaangvfddanvQNRTLSDWDGVWQSVYpllqsgkldpvfqkkadadktktfaeikdyyhkgyaTDIEMIGIED-----GIVEFHRN----NETTSCKYDYDGYkiltyksgkkgvrYLFECKDPeskapKYIQFSDhiiaprKSSHFHIFMGNDsqqsllnemeNWPTYYPYQLsseeVVEEMMSH-----------------------------

d1gm6a_ vvtsnfdaSKIAGEWYSILLAS--DAKE------niEENGSmrvFVEHIRVLDNSsLAFKFQRKvngeCTDFYAV--------------------cDKVGD---GVYTV-ayyGENKFRLlEVNYsDYVILHLVDVngdkTFQLMEFYGRKpdvepklkdkfveicqqygiikeniidltkidrcfqlrg

d1opaa_ -------tKDQNGTWEMESNENfeGYMKaldidfatRKIAVrltQTKIIVQDGDN-FKTKTNST----FRNYDLDftvgvefdehtkgldgrnvktLVTWEgntLVCVQkgekENRGWKQ-WVEG-DKLYLELTCG----DQVCRQVFKKK---------------------------------------

d1gm6a_ vvtsnfdasKIAGEWYSILLAS--DAKE------niEENGSmRVFVEHIRVLDnSSLAFKFQRKvngeCTDFYAVCD-----------------KVGD----GVYTvayygENKFRLlEVNYsDYVILHLVDVngdkTFQLMEFYGRKpdvepklkdkfveicqqygiikeniidltkidrcfqlrg

d1p6pa_ ---------AFNGTWNVYAQENyeNFLRtvglpediIKVAKdVNPVIEIEQNG-NEFVVTSKTP----KQTHSNSFTvgkeseitsmdgkkikvTVQLeggkLICK---sdKFSHIQ-EVNG-DEMVEKITIG----SSTLTRKSKRV---------------------------------------

d1gm6a_ -VVTS------------------NFDASKIAGEWYSILLASdakenieenGSMR---vFVEHIRVLDNSSLAFKFQRKV--NGECTDFYAVCDKVG---DGVYTVAYY--GENKFRLLEVN-YSDYVILHLVdvngdktFQLMEFYGRKPDVEPKLKDKFVEICQQYGIIKENIIDLTKIdrcfqlrg

d1qwda_ hLESTslykkssstpprgvtvvnNFDAKRYLGTWYEIARFD--------hRFERglekVTATYSLRDDGGLNVINKGYNpdRGMWQQSEGKAYFTGaptRAALKVSFFgpFYGGYNVIALDrEYRHALVCGP------dRDYLWILSRTPTISDEVKQEMLAVATREGFDVSKFIWVQQP-------g

d1gm6a_ vvtsnfdaSKIAGEWYSiLLASDA------KENIeengsmRVFVEHIRVLDnSSLAFKFQRKVNGECTDFYAVCDKVgdgvyTVAY--------YGEN------------------------KFRLLevnYSDYVILHLVDVNGD-KTFQLMEFYGRKPdvepklkdkfveicqqygiikeniidltkidrcfqlrg

d1r0ua_ --gfqsnaMKQETPITL-HVKSVIeddgnqEVIE------FRTTGFYYVKQ-NKVYLSYYEEHDLGKVKTIVKVSEG-----EVLVmrsgavkmNQRFvtgastiakykmsfgelelktstkSIQSD--lDEEKGRISIAYDMHVgHLHNMTITYEGGT--------------------------------------

d1gm6a_ vvtsnfdasKIAGEWYSILLA--------sdakenieengsmrVFVEHIRVLDnSSLAFKFQRKvngeCTDFYAVC-----------------dkvgdgvytvayygenkfrlLEVNySDYVILHLVDVngdkTFQLMEFYGRKpdvepklkdkfveicqqygiikeniidltkidrcfqlrg

d1tvqa_ ---------AFSGTWQVYAQEnyeeflkalalpedlikmardiKPIVEIQQKG-DDFVVTSKTP----RQTVTNSFtlgkeadittmdgkklkctvhlangklvtksekfsheQEVK-GNEMVETITFG----GVTLIRRSKRV---------------------------------------

d1gm6a_ -----------------------------------------------------------------------------------------------------------------------------------------------------vvtsNFDAskIAGEWYSiLLASdakenIEEN-------------------------gSMRVFVeHIRVLdnSSLAFKFQRKVngecTDFYAV--------------------CDKVGDGVYTVAY-------yGENKFRLLEvnysdYVILHLVDVNGDKTFQLMEFYGR------kpdvepklkdkfveicqqygiikeniidltkidrcfqlrg

d1vpra1 ekgfeagdnklggalnakhvekygdnfkngxhkpefhedglhkpxevggkkfesgfhyllechelggknasggyggplcedpygsevqaxtekllkeadsdrtlcfnnfqdpcpqltkeqvaxckgfdygdktlklpcgplpwpaglpepgyvPKTN--PLHGRWI-TVSG----gQAAFikeaiksgmlgaaeankivadtdhhqTGGXYL-RINQF-gDVCTVDASVAK---fARAKRTwksghyfyeplvsggnllgvWVLPEEYRKIGFFwexesgrcFRIERRAFP--vgpYTFXRQATEVGGKISFVFYVKVSndpesdpiplqsrdytalagrdnaptnlgkpyptlakdldypkkrd

d1gm6a_ vvtsnfdaSKIAGEWYSILLAS--DAKE-------nieENGS-mRVFVEHIRVLDnSSLAFKFQRKvngeCTDFYAV-----------------cdKVGD-----GVYT----vayygeNKFRLlEVNYSDYVILHLVDVngdkTFQLMEFYGRKpdvepklkdkfveicqqygiikeniidltkidrcfqlrg

d1xcaa_ --------PNFSGNWKIIRSENfeELLKvlgvnvmlrkIAVAaaSKPAVEIKQEG-DTFYIKTSTT----VRTTEINfkvgeefeeqtvdgrpcksLVKWesenkMVCEqkllkgegpkTSWTM-ELTNDGELILTMTAD----DVVCTRVYVRE---------------------------------------

d1gm6a_ vvtsnfdasKIAGEWYSILLASdakenieenGSMRVFVEHIRVLDNSSLAFKFQRkvNGECTDFYAVCDKV-GDGVYTVAyygENKFRLLEVNYSDYVILHLVDVngDKTFQLMEFYGRKP-DVEPkLKDKFVEICQQYGIIKENIIDlTKIDrcfqlrg

d1xkia_ ---------DVSGTWYLKAMTV---------NLESVTPMTLTTLEGGNLEAKVTM--SGRCQEVKAVLEKTdEPGKYTAD-ggKHVAYIIRSHVKDHYIFYSEGE--GKPVRGVKLVGRDPkNNLE-ALEDFEKAAGARGLSTESILI-PRQS-------

d1gm6a_ ----vvTSNFDASKIAGEWYSILLASDAKENIE-ENGSMRVFVEHIRVLDNSSLAFKFQRKVNGECTDFYAVCDKV-GDGVYTVAYYGENKFRLLEVNYSDYVILHLVDVNgdkTFQLMEFYGRKPDVEPKLKDKFVEICQQYGIIkeNIIDLTK---iDRCFqlrg

d1yupa1 iivtqtMKDLDVQKVAGTWYSLAMAASDISLLDaQSAPLRVYVEELKPTPGGDLEILLQKWENGKCAQKKIIAEKTeIPAVFKIDALNENKVLVLDTDYKKYLLFCMENSAepeQSLACQCLVRTPEVDDEAMEKFDKALKALPMH--IRLSFNPtqleEQCR---v

d1gm6a_ --vvtsnfDASKIAGEWYSiLLASDA---KENIeengsmRVFVEHIRVLDNSSLAFKFQRK--vNGEC-TDFYAVCD---------------------KVGD------GVYTVA------yyGENKFRLLEVNysDYVILHLVDVNG-DKTF-QLMEFYGRKpdvepklkdkfveicqqygiikeniidltkidrcfqlrg

d2a13a1 ppvhpfvaPLSYLLGTWRG-QGEGEYptiPSFR------YGEEIRFSHSGKPVIAYTQKTWkleSGAPxHAESGYFRprpdgsievviaqstglvevqKGTYnvdeqsIKLKSDlvgnaskvKEISREFELVD--GKLSYVVRXSTTtNPLQpHLKAILDKL---------------------------------------

d1gm6a_ vvtsnfdaSKIAGEWYSILLAS--DAKE------niEENGsmrvFVEHIRVLDnSSLAFKFQRKvngeCTDFYAVCD------------------KVGD----GVYTvayyGENKFRLlEVNYsDYVILHLVDVngdkTFQLMEFYGRKpdvepklkdkfveicqqygiikeniidltkidrcfqlrg

d2f73a1 --------MSFSGKYQLQSQENfeAFMKaiglpeelIQKGkdikGVSEIVQNG-KHFKFTITAG----SKVIQNEFTvgeeceletmtgekvktvVQLEgdnkLVTT---fKNIKSVT-ELNG-DIITNTMTLG----DIVFKRISKRI---------------------------------------

d1gm6a_ -vvtsnFDASKIAGEWysillasdakenieengsmrvfvEHIRVLDNSSLAFKFQRKV---NGECTDFYAVCDKVGDGVYTVAY------YGENKFRLlEVNYsDYVILHLVD----VNGD-------ktFQLMEFYGRKPdvepklkdkfveicqqYGIIkeNIIDLtkidrcfqlrg

d2fr2a1 dlapalQALSPLLGSW----agrgagkyptirpfeyleeVVFAHVGKPFLTYTQQTRAvadGKPLHSETGYLRVCRPGCVELVLahpsgiTEIEVGTY-SVTG-DVIELELSTradgSIGLaptakevtaLDRSYRIDGDE----lsyslqmravgqPLQD--HLAAV------lhrqr

d1gm6a_ ----vvtsnfDASKiAGEWYSILlasdakenieengsmrvFVEHIRVLDNSSLAFKFQRkvngectDFYAV-CDKVGD--------GVYTVAyyGENKFRLlEVNY-SDYVILHLVDVN----------------------------GDKTFQLMEFYGRKpdvepklkdkfveicqqygiikeniidltkidrCFQLRG-----------------

d2gc9a1 xtktfktlddFLGT-HFIYTYDN----------------gWEYEWYAKNDHTVDYRIHG---gxvaGRWVTdQKADIVxltegiykISWTEP--TGTDVAL-DFXPnEKKLHGTIFFPKwveehpeitvtyqnehidlxeqsrekyaTYPKLVVPEFANIT----------------yxgdagqnnedviseapYKEXPNdirngkyfdqnyhrlnk

d1gm6a_ vvTSNFDASKIAGEWYSiLLASDA----KENIeengsmRVFVEHIRVLDnssLAFKFQRkvngeCTDFYAV----------------cDKVGD-----GVYT----vayyGENKFRLlEVNYSDYVILHLVDVN--GDKTFQLMEFYGRKpdvepklkdkfveicqqygiikeniidltkidrcfqlrg

d2o62a1 -eRPLLQINDLLGEWRG-QAVTIYrdlrPPDI------YSTTLKIQLDDagrLXQSTSF----gERTITSTatikgsivlfdqdpekqVQVLLlpdgaSATSplkvqlrqPLFLEAG-WLIQSDLRQRXIRSYNdkGEWVSLTLVTEERV---------------------------------------

d1gm6a_ ------vvTSNFDASKIA--GEWYSILLASDAKENIEENgsmRVFVEHIRVLDNSSLAFKFQRKVNGECTDFYAV-CDKVGDGVYTVAY---------------yGENKFRLLEVNySDYVILHLVDVNGDKT-FQLMEFYGRKP-DVEPK-LKDKFVEICQqygiIKENIIDLTKIDRcFQLRG--------

d2ofmx1 actknaiaQTGFNKDKYFngDVWYVTDYLDLEPDDVPKR---YCAALAAGTASGKLKEALYHYDPKTQDTFYDVSeLQVESLGKYTANFkkvdkngnvkvavtagNYYTFTVMYAD-DSSALIHTCLHKGNKDlGDLYAVLNRNKdAAAGDkVKSAVSAATL----EFSKFISTKENNC-AYDNDslkslltk

d1gm6a_ -VVTSN---FDASKIAGEWYSILLASDAKENIEENGSMRVFVEHIRVLdNSSLAFKFQRKVNGECTDFYAVCDKV-GDGVYTVAYYGENKFRLLEVNYSDYVILHLVDVNGDKTFQLMEFYGRKPDVEPKLKDKFVEICQQYGIIKENIIDLTKiDRCFqlrg

d2ozqa1 eEASSTgrnFNVEKINGEWHTIILASDKREKIEDNGNFRLFLEQIHVL-EKSLVLKFHTVRDEECSELSMVADKTeKAGEYSVTYDGFNTFTIPKTDYDNFLMAHLINEKDGETFQLMGLYGREPDLSSDIKERFAQLCEEHGILRENIIDLSN-ANRC----

d1gm6a_ ------vvTSNFDASKIA--GEWYSILLASDAKEniEENGsmrVFVEHIRVLDNsSLAFKFQRKV--NGECTDFYAVCDKVGDGVYTVAY---------------yGENKFRLLEVNySDYVILHLVDVNGDKT-FQLMEFYGRKPDVE-PKLKDKFVEICQQygiIKENIIDLTKIDRCfQLRG--------

d3np1a_ kctknalaQTGFNKDKYFngDVWYVTDYLDLEPDdvPKRY---CAALAAGTASG-KLKEALYHYDpkTQDTFYDVSELQEESPGKYTANFkkvekngnvkvdvtsgNYYTFTVMYAD-DSSALIHTCLHKGNKDlGDLYAVLNRNKDTNaGDKVKGAVTAASL---KFSDFISTKDNKCE-YDNVslkslltk

d1hbqa_ erdCRVS--SFRVKENFDKARFA-GTWYAMAkkdpeglflqdnivaeFSVDeNGQMsatakgrvrllnnwdVCADMVGTFTD-TEDPaKFKMKYWGVAsfLQKGNDDHWIIDTDYETFAVQYSCRllnldgtcADSYSFVFARDPSgfspevqkivrqrqeelclarqyrliPHNG-YCNG

d1avgi_ --aEGDDcsIEKAMGDFKPEEFFnGTWYLAH----gpgvtspavcqkFTTS-GSKG--ftqiveigynkfeSNVKFQCNQVDnKNGE-QYSFKCKSSD--NTEFEADFTFISVSYDNFALVCRSI--tftsqpKEDRYLVFERTKS-------------------------dTDPDaKEIC

d1hbqa_ erdcrvssfrvkenfdkaRFAGTWYAMAKK---------------dpeglflqDNIVAEFSVDeNGQMSATAKGRvrllnnwdvCADMVG------------------tfTDTEDP---AKFKmkywgvasflqkgnDDHWIIDTDYetFAVQYSCRLlnldgtcADSYSFVFARDpsgfspevqkivrqrqeelclarqyrliphngycng

d1cbia_ -----------------pNFAGTWKMRSSEnfdellkalgvnamlrkvavaaaSKPHVEIRQD-GDQFYIKTSTT---------VRTTEInfkvgegfeeetvdgrkcrsLPTWENenkIHCT----qtllegdgpkTYWTRELAND--ELILTFGAD-------DVVCTRIYVRE------------------------------------

d1hbqa_ erdcrvssfrvkenfdKARFAG-TWYAMAKKD---PEGL----------FLQDNIVAEFSVDENGQMSATAKGRvrllnnwdvCADMVGTF------------------TDTEDP---AKFKmkywgvasflqkGNDDHWIiDTDYeTFAVQYSCRLlnldgtcADSYSFVFARDpsgfspevqkivrqrqeelclarqyrliphngycng

d1ftpa_ ----------------VKEFAGiKYKLDSQTNfeeYMKAigvgaierkaGLALSPVIELEILDGDKFKLTSKTA---------IKNTEFTFklgeefdeetldgrkvksTITQDGpnkLVHE--------qkgdHPTIIIR-EFSK-EQCVITIKLG-------DLVATRIYKAQ------------------------------------

d1hbqa_ erdcrvssfrvkenfdkARFAGTWYAMAKK-DPEGL------------FLQDNIVAEFSVDeNGQMSATAKGRvrllnnwdvCADMVGTF--------------------TDTED--pAKFKmkywgvasflqkgnDDHWIiDTDYeTFAVQYSCRLlnldgtcADSYSFVFARDpsgfspevqkivrqrqeelclarqyrliphngycng

d1ggla_ ----------------pPNLTGYYRFVSQKnMEDYLqalnislavrkiALLLKPDKEIEHQ-GNHMTVRTLST---------FRNYTVQFdvgvefeedlrsvdgrkcqtIVTWEeehLVCV-------qkgevpnRGWRH-WLEG-EMLYLELTAR-------DAVCEQVFRKV-----------------------------------h

d1hbqa_ erdcRVSSFRVKENFDKARFAGTWYAMAKKDPEglflQDNIVAEFSVDeNGQMSATAKGRVRllnnwDVCADMVGTFTDTEDPAKFKMKywgvasflqKGNDDHWIIDTDYETFAVQYSCRLLnldgtcaDSYSFVFARDPSgFSPEVQKIVRQRQEELCLAR-QYRLIPHNGYC----------ng

d1iw2a_ --asPISTIQPKANFDAQQFAGTWLLVAVGSAG---rAEATTLHVAPQ-GTAMAVSTFRKLD-----GICWQVRQLYGDTGVLGRFLLQ-----argaRGAVHVVVAETDYQSFAVLYLERAG-------QLSVKLYARSLP-VSDSVLSGFEQRVQEAHLTEdQIFYFPKYGFCeaadqfhvldev

d1hbqa_ ERDCRVSSFRVKENFDKARFAGTWYAMAKKDPEGLFLQDNIVAEFSVDENGQMSATAKGRVRLLNNWDVCADMVGTFTDTEDPAKFKMKYWGVASFLQKGNDDHWIIDTDYETFAVQYSCRLLNLDGTCADSYSFVFARDPSGFSPEVQKIVRQRQEELCLARQYRLIPHNGYCng

d1jyda_ ERDCRVSSFRVKENFDKARFSGTWYAMAKKDPEGLFLQDNIVAEFSVDETGQMSATAKGRVRLLNNWDVCADMVGTFTDTEDPAKFKMKYWGVASFLQKGNDDHWIVDTDYDTYAVQYSCRLLNLDGTCADSYSFVFSRDPNGLPPEAQKIVRQRQEELCLARQYRLIVHNGYC--

d1hbqa_ erdcrvssfrvkenfdkaRFAGTWYAMAKK-------------dpeglflqdNIVAEFSVDeNGQMSATAKGRvrllnnwdvCADMVGTF---------------------TDTEDPAKFKmkywgvasflqkgnddHWIIDTDyeTFAVQYSCRLlnldgtcADSYSFVFARDPsgfspevqkivrqrqeelclarqyrliphngycng

d1kqxa_ ----------------paDFNGTWEMLSNDnfedvmkaldidfatrkiavhlKQTKVIVQN-GDKFETKTLST---------FRNYEVNFvigeefdeqtkgldnrtvktlVKWDGDKLVC------vqkgekenrgWKQWIEG--DLLHLEIHCQ-------DKVCHQVFKKKN-----------------------------------

d1hbqa_ erdcrvssfrvkenfdKARFAGTWYAMAKK-DPEGL------------FLQDNIVAEFSVDENgQMSATAKGRvrllnnwdvCADMVGT------------------fTDTEDP--AKFKMkywgvasflqkgNDDHWIIDTDyeTFAVQYSCRLlnldgtcADSYSFVFARDpsgfspevqkivrqrqeelclarqyrliphngycng

d1liba_ ----------------CDAFVGTWKLVSSEnFDDYMkevgvgfatrkvAGMAKPNMIISVNGD-LVTIRSEST---------FKNTEISfklgvefdeitaddrkvksIITLDGgaLVQVQ-------kwdgkSTTIKRKRDG--DKLVVECVMK-------GVTSTRVYERA------------------------------------

d1hbqa_ erdcrvssfrvkenfdkaRFAGTWYAMAKK-DPEGL------------FLQDNIVAEFSVDeNGQMSATAKGRvrllnnwdvCADMVGTF--------------------TDTEDP--AKFKmkywgvasflqkgnDDHWIiDTDYeTFAVQYSCRLlnldgtcADSYSFVFARDpsgfspevqkivrqrqeelclarqyrliphngycng

d1lpja_ ----------------paDLSGTWTLLSSDnFEGYMlalgidfatrkiAKLLKPQKVIEQN-GDSFTIHTNSS---------LRNYFVKFkvgeefdednrgldnrkcksLVIWDNdrLTCI-------qkgekknRGWTH-WIEG-DKLHLEMFCE-------GQVCKQTFQRA------------------------------------

d1hbqa_ ------erdCRVSSFR---VKENF--DKARFAGTWYAMAKKDPEGLFLQDNIVAEFSVDeNGQMSATAKGRVRLlnnwDVCADMVGTFTDT--EDPAkFKMKYWgvasflQKGNDDHWIIDTDYETFAVQYSCrLLNLDGTCaDSYSFVFARDPsGFSPEVQKIVRQRQEELCLA-RQYRLIPHN-GYCNG-----

d1obqa_ dkipdfvvpGKCASVDrnkLWAEQtpNRNSYAGVWYQFALTNNPYQLIEKCVRNEYSFD-GKQFVIESTGIAYD----GNLLKRNGKLYPNpfGEPH-LSIDYE------NSFAAPLVILETDYSNYACLYSC-IDYNFGYH-SDFSFIFSRSA-NLADQYVKKCEAAFKNINVDtTRFVKTVQGsSCPYDtqktl

d1hbqa_ ---------erdcrvssfrvkENFDKARFAGTWYAMA---------------------------kkdpeglflqdnIVAEFSVdengqMSATAKGRvrllnnwdvCADMVGTFTDTED------------pAKFKMKywgvasflQKGN--DDHWIID----tdYETFAVQYSCRLlnldgtcadsYSFVFAR-DPSG--FSPEVQKIvrqrqeelclarqyrliphngycng

d1oeja_ gkplteveqkaangvfddanvQNRTLSDWDGVWQSVYpllqsgkldpvfqkkadadktktfaeikdyyhkgyatdiEMIGIED-----GIVEFHRN---------NETTSCKYDYDGYkiltyksgkkgvrYLFECK-------dPESKapKYIQFSDhiiaprKSSHFHIFMGNDsqqsllnemeNWPTYYPyQLSSeeVVEEMMSH-------------------------

d1hbqa_ erdcrvssfrvkenfdkARFAGTWYAMAKK-DPEGL------------FLQDNIVAEFSVDeNGQMSATAKGRvrllnnwdvCADMVGTF--------------------TDTEDP--AKFKmkywgvasflqkgnDDHWIiDTDYeTFAVQYSCRLlnldgtcADSYSFVFARDpsgfspevqkivrqrqeelclarqyrliphngycng

d1opaa_ ----------------tKDQNGTWEMESNEnFEGYMkaldidfatrkiAVRLTQTKIIVQD-GDNFKTKTNST---------FRNYDLDFtvgvefdehtkgldgrnvktLVTWEGntLVCV-------qkgekenRGWKQ-WVEG-DKLYLELTCG-------DQVCRQVFKKK------------------------------------

d1hbqa_ erdcrvssfrvkenfdkaRFAGTWYAMAKKD---PEGL----------FLQDNIVAEFSVDenGQMSATAKGRvrllnnwdvCADMVGTFTD----------------TEDP----AKFKmkywgvasflqkgnDDHWIiDTDYeTFAVQYSCRLlnldgtcADSYSFVFARDpsgfspevqkivrqrqeelclarqyrliphngycng

d1p6pa_ ------------------AFNGTWNVYAQENyenFLRTvglpediikvAKDVNPVIEIEQN-gNEFVVTSKTP---------KQTHSNSFTVgkeseitsmdgkkikvTVQLeggkLICK-----------sdkFSHIQ-EVNG-DEMVEKITIG-------SSTLTRKSKRV------------------------------------

d1hbqa_ ----------erdcrVSSFRVKENFDKARFAGTWYAMAKKDPegLFLQ--DNIVAEFSVDENGQMSATAKGRVRLlnnWDVCADMVGTFTDTED--PAKFKMKYWgvasflQKGNDDHWIIDTDY-ETFAVQYSCRllnldgtcadSYSFVFARDPsGFSPEVQKIVRQRQEELCLA-RQYRLIPHNGycng

d1qwda_ hlestslykkssstpPRGVTVVNNFDAKRYLGTWYEIARFDH--RFERglEKVTATYSLRDDGGLNVINKGYNPD---RGMWQQSEGKAYFTGAptRAALKVSFF------GPFYGGYNVIALDReYRHALVCGPD---------rDYLWILSRTP-TISDEVKQEMLAVATREGFDvSKFIWVQQPG----

d1hbqa_ erdcrvssfrvkenfDKARfAGTWYAmAKKDPE-----glflqDNIVAEFSVDeNGQMSATAKGRVRllnnwDVCADMVGTFTDtedpakFKMKYWGVASFLQKGNDD------------------------HWIIdtdYETFAVQYSCRLLNLDgtCADSYSFVFARDPsgfspevqkivrqrqeelclarqyrliphngycng

d1r0ua_ ----------gfqsnAMKQ-ETPITL-HVKSVIeddgnqevieFRTTGFYYVK-QNKVYLSYYEEHD-----LGKVKTIVKVSE------GEVLVMRSGAVKMNQRFVtgastiakykmsfgelelktstksIQSD--lDEEKGRISIAYDMHVG--HLHNMTITYEGGT-----------------------------------

d1hbqa_ erdcrvssfrvkenfdkaRFAGTWYAMAKKD---PEGL----------FLQDNIVAEFSVDEnGQMSATAKGRvrllnnwdvCADMVGTF------------------TDTEDP--AKFKmkywgvasflqkgnDDHWIiDTDYeTFAVQYSCRLlnldgtcADSYSFVFARDpsgfspevqkivrqrqeelclarqyrliphngycng

d1tvqa_ ------------------AFSGTWQVYAQENyeeFLKAlalpedlikmARDIKPIVEIQQKG-DDFVVTSKTP---------RQTVTNSFtlgkeadittmdgkklkcTVHLANgkLVTK-----------sekFSHEQ-EVKG-NEMVETITFG-------GVTLIRRSKRV------------------------------------

d1hbqa_ ----------------------------------------------------------------------------------------------erdcrvSSFRV---------------------------------------------kenfdkARFAGTWYAMAK-----------------------kdpeglflqdNIVAEFSVDeNGQMSATAKGRVrllnnwdvCADMVGTF-------------------tDTED--pAKFKmkywgvasflqkgnDDHWIiDTDY---ETFAVQYSCRLlnldgtcADSYSFVFARD-----------psgfspevqkivrqrqeelclarqyrliphngycng

d1vpra1 ekgfeagdnklggalnakhvekygdnfkngxhkpefhedglhkpxevggkkfesgfhyllechelggknasggyggplcedpygsevqaxtekllkeadsDRTLCfnnfqdpcpqltkeqvaxckgfdygdktlklpcgplpwpaglpepgyvpktNPLHGRWITVSGgqaafikeaiksgmlgaaeankivadtdhhqtgGXYLRINQF-GDVCTVDASVAK--------FARAKRTWksghyfyeplvsggnllgvwVLPEeyrKIGF-----fwexesgrcFRIER-RAFPvgpYTFXRQATEVG-------GKISFVFYVKVsndpesdpiplqsrdytalagrdnaptnlgkpyptlakdldypkkrd

d1hbqa_ erdcrvssfrvkenfdkARFAGTWYAMAKKD---PEGL------------fLQDNIVAEFSVDeNGQMSATAKGRvrllnnwdvCADMVGTF------------------TDTEDP---AKFKmkywgvasflqkgnDDHWIiDTDYETFAVQYSCRLlnldgtcADSYSFVFARDpsgfspevqkivrqrqeelclarqyrliphngycng

d1xcaa_ -----------------PNFSGNWKIIRSENfeeLLKVlgvnvmlrkiavaAASKPAVEIKQE-GDTFYIKTSTT---------VRTTEINFkvgeefeeqtvdgrpcksLVKWESenkMVCE----qkllkgegpkTSWTM-ELTNDGELILTMTAD-------DVVCTRVYVRE------------------------------------

d1hbqa_ erdcrvssfrvkenfdkaRFAGTWYAMAKKDpegLFLQDnIVAEFSVDENGQMSATAKGrvrllnnWDVCADMVGTFTDTEDPAKFKMKywgvasflqkgNDDHWIIDTDYETFAVQYSCRLLNldgtcADSYSFVFARDPSGFSPeVQKIVRQRQEELCLARQ-YRLIPHngycng

d1xkia_ ------------------DVSGTWYLKAMTV---NLESV-TPMTLTTLEGGNLEAKVTM-------SGRCQEVKAVLEKTDEPGKYTAD---------ggKHVAYIIRSHVKDHYIFYSEGEGK-----PVRGVKLVGRDPKNNLE-ALEDFEKAAGARGLSTEsILIPRQ-----s

d1hbqa_ erdcrvSSFRVKENFDKARFAGTWYAMAKKD-PEGL------FLQDnIVAEFSVDENGQMSATAKGRVrllnnWDVCADMVGTFTDTEDPAKFKMKYwgvasflqKGNDDHWIIDTDYETFAVQYSCRLLnldgtCADSYSFVFARDPSGFSpEVQKIVRQRQEELCLarQYRLI---PHNGYCNg

d1yupa1 -----iIVTQTMKDLDVQKVAGTWYSLAMAAsDISLldaqsaPLRV-YVEELKPTPGGDLEILLQKWE-----NGKCAQKKIIAEKTEIPAVFKIDA--------LNENKVLVLDTDYKKYLLFCMENSA---epEQSLACQCLVRTPEVDD-EAMEKFDKALKALPM-hIRLSFnptQLEEQCRv

d1hbqa_ erdcrvssfrvkenfDKARFAGTWYAmAKKDPEG--lflQDNIVAEFSVDENGQMSATAKGRVrllnnwDVCADMVGTFTD-TEDPakfKMKYWGVASflQKGND---------------------------DHWIIDTDyeTFAVQYSCRlLNLDGTCADSYSFVFARDpsgfspevqkivrqrqeelclarqyrliphngycng

d2a13a1 -------ppvhpfvaPLSYLLGTWRG-QGEGEYPtipsfRYGEEIRFSHSGKPVIAYTQKTWK--lesgAPXHAESGYFRPrPDGS--iEVVIAQSTG--LVEVQkgtynvdeqsiklksdlvgnaskvkeiSREFELVD--GKLSYVVRX-STTTNPLQPHLKAILDKL------------------------------------

d1hbqa_ erdcrvssfrvkenfdkARFAGTWYAMAKKD---PEGL----------fLQDNIVAEFSVDEnGQMSATAKGRvrllnnwdvCADMVGTF------------------TDTED---pAKFKmkywgvasflqkgNDDHWIiDTDYeTFAVQYSCRLlnldgtcADSYSFVFARDpsgfspevqkivrqrqeelclarqyrliphngycng

d2f73a1 -----------------MSFSGKYQLQSQENfeaFMKAiglpeeliqkgKDIKGVSEIVQNG-KHFKFTITAG---------SKVIQNEFtvgeeceletmtgekvktVVQLEgdnkLVTT-----------fkNIKSVT-ELNG-DIITNTMTLG-------DIVFKRISKRI------------------------------------

d1hbqa_ erdcrvssfrvkenFDKARFAGTWYAmAKKDPE--glflQDNIVAEFSVDENGQMSATAKGRVrllnnwDVCADMVGTFTDTEDPaKFKMKYWGVASflQKGNDD----------------------------------hwiIDTDyETFAVQYSCRLlnlDGTCADSYSFVFARDPsgfspevqkivrqrqeelclarqyrliphngycng

d2fr2a1 --------dlapalQALSPLLGSWAG-RGAGKYptirpfEYLEEVVFAHVGKPFLTYTQQTRA--vadgKPLHSETGYLRVCRPG-CVELVLAHPSG--ITEIEVgtysvtgdvielelstradgsiglaptakevtaldrsYRID-GDELSYSLQMR-avGQPLQDHLAAVLHRQR-----------------------------------

d1hbqa_ erdcrvssfrvkENFD--KARFA--------------------GTWYAmaKKDPEGL--flqdnIVAEFSVDENGQMSATAKGRvrllnnwdVCADMVGTFTDTedpaKFKMKYWGVA---------------------------------SFLQKGNDDH-wIIDTDYETfavqyscrllnldgtcadsysfvfardpsgfspevqkivrQRQEELCLArqyrliphngycng

d2gc9a1 -----------xTKTFktLDDFLgthfiytydngweyewyaknDHTVD-yRIHGGXVagrwvtdQKADIVXLTEGIYKISWTEP--------TGTDVALDFXPN----EKKLHGTIFFpkwveehpeitvtyqnehidlxeqsrekyatypKLVVPEFANItyXGDAGQNN-----------------------------edviseapykeXPNDIRNGK---yfdqnyhrlnk

d1hbqa_ erdcrvssfrvkENFDKARFAGTWYAmAKKDPE---glflqDNIVAEFSVDENGQMSATAKGRVrllnnwdvcADMVGTF----------------TDTEDP----AKFKmkywgvasflqkgnDDHWIIDTDyETFAVQYSCRlLNLDGTCADSYSFVFARDpsgfspevqkivrqrqeelclarqyrliphngycng

d2o62a1 ----------erPLLQINDLLGEWRG-QAVTIYrdlrppdiYSTTLKIQLDDAGRLXQSTSFGE---------RTITSTAtikgsivlfdqdpekqVQVLLLpdgaSATS----plkvqlrqplFLEAGWLIQ-SDLRQRXIRS-YNDKGEWVSLTLVTEERV------------------------------------

d1hbqa_ erdcrVSSFRVKENFDKARFA--GTWYAMAKKDP--EGLFlQDNIVAEFSVDENgQMSATAKGRVRllnnWDVCADMVGTFTDTeDPAKFKMKYWGVA----SFLQK---GNDDHWIIDTDyETFAVQYSCRllnldgtcaDSYSFVFARDPSGFspeVQKIVRQRQEELCLA-RQYRLIP-HNGYCN---------g

d2ofmx1 ---acTKNAIAQTGFNKDKYFngDVWYVTDYLDLepDDVPkRYCAALAAGTASG-KLKEALYHYDP---kTQDTFYDVSELQVE-SLGKYTANFKKVDkngnVKVAVtagNYYTFTVMYAD-DSSALIHTCL--hkgnkdlGDLYAVLNRNKDAA---AGDKVKSAVSAATLEfSKFISTKeNNCAYDndslkslltk

d1hbqa_ erdcrvssfrVKEN--FDKARFAGTWYAMAKKD-PEGL-----FLQDnIVAEFSVDeNGQMSATAKGRVrllnnWDVCADMVGTFTDTEDPAKFKMKYwgvasflqKGNDDHWIIDTDYETFAVQYSCRLlnldgtcADSYSFVFARDPsGFSPEVQKIVRQRQEELCLAR-QYRLI--PHNGycng

d2ozqa1 -------eeaSSTGrnFNVEKINGEWHTIILASdKREKiedngNFRL-FLEQIHVL-EKSLVLKFHTVR-----DEECSELSMVADKTEKAGEYSVTY--------DGFNTFTIPKTDYDNFLMAHLINE---kdgeTFQLMGLYGREP-DLSSDIKERFAQLCEEHGILReNIIDLsnANRC----

d1hbqa_ erdcrVSSFRVKENFDKARFA--GTWYAMAKKDPEG--lflQDNIVAEFSVDENgQMSATAKGRVRllnnWDVCADMVGTFTDTeDPAKFKMKYWGVA----SFLQK---GNDDHWIIDTDyETFAVQYSCRLlnldgtcADSYSFVFARDPSGFspeVQKIVRQRQEELCLA-RQYRLIP-HNGYCN---------g

d3np1a_ ---kcTKNALAQTGFNKDKYFngDVWYVTDYLDLEPddvpkRYCAALAAGTASG-KLKEALYHYDP---kTQDTFYDVSELQEE-SPGKYTANFKKVEkngnVKVDVtsgNYYTFTVMYAD-DSSALIHTCLH--kgnkdLGDLYAVLNRNKDTN---AGDKVKGAVTAASLKfSDFISTKdNKCEYDnvslkslltk

d1iw2a_ --ASPISTIQPKANFDAQQFA-GTWLLVAvgsagRAEAttlhvAPQG---tamavstfrkldgiCWQVRQLYGD-TGVLgRFLLQ---argARGAVHVVVAETDYQSFAVLYLER-----AGQLSVKLYARSLPvsdsvlsgfeqrvqeahltedqifyfPKYG-FCEAadqfhvldev

d1avgi_ aeGDDCSIEKAMGDFKPEEFFnGTWYLAH-gpgvTSPAvcqkfTTSGskgftqiveigynkfesNVKFQCNQVDnKNGE-QYSFKckssdnTEFEADFTFISVSYDNFALVCRSItftsqPKEDRYLVFERTKS-------------------------dTDPDaKEIC----------

d1iw2a_ aspistiqpkanfdaQQFAGTWLLVAVG------------------sagraEATTLHVAPQGTAMAVSTFRKldgiCWQVRQLYGD-----------------TGVL----GRFLlqARGA--RGAVHVVVAETDYqsFAVLYLERAGQLSVKLYARSlpvsdsvlsgfeqrvqeahltedqifyfpkygfceaadqfhvldev

d1cbia_ ---------------PNFAGTWKMRSSEnfdellkalgvnamlrkvavaaaSKPHVEIRQDGDQFYIKTSTT----VRTTEINFKVgegfeeetvdgrkcrslPTWEnenkIHCT-qTLLEgdGPKTYWTRELAND--ELILTFGADDVVCTRIYVRE----------------------------------------------

d1iw2a_ aspistiqpkanfdAQQFAG-TWLLVAVGSAG----------------RAEATTLHVAPQG-taMAVSTFRKldgiCWQVRQL------------------yGDTGVL---GRFLlQARGarGAVHVVVAetdYQSFAVLYLERAGQLSVKLYARSlpvsdsvlsgfeqrvqeahltedqifyfpkygfceaadqfhvldev

d1ftpa_ --------------VKEFAGiKYKLDSQTNFEeymkaigvgaierkagLALSPVIELEILDgdkFKLTSKTA----IKNTEFTfklgeefdeetldgrkvksTITQDGpnkLVHE-QKGD--HPTIIIRE--fSKEQCVITIKLGDLVATRIYKAQ----------------------------------------------

d1iw2a_ aspistiqpkanfdAQQFAGTWLLVAVGSAG----------------RAEATTLHVAPQGTAMAVSTFRKldgiCWQVRQL--------------------ygDTGV-LGRFLLQArGARGAVHVVVaETDYqSFAVLYLERAGQLSVKLYARSlpvsdsvlsgfeqrvqeahltedqifyfpkygfceaadqfhvldev

d1ggla_ --------------PPNLTGYYRFVSQKNMEdylqalnislavrkiaLLLKPDKEIEHQGNHMTVRTLST----FRNYTVQfdvgvefeedlrsvdgrkcqtiVTWEeEHLVCVQK-GEVPNRGWRH-WLEG-EMLYLELTARDAVCEQVFRKV---------------------------------------------h

d1iw2a_ --asPISTIQPKANFDAQQFAGTWLLVAVGSAG---rAEATTLHVAPQ-GTAMAVSTFRKLD-----GICWQVRQLYGDTGVLGRFLLQ-----argaRGAVHVVVAETDYQSFAVLYLERAG-------QLSVKLYARS-LPVSDSVLSGFEQRVQEAHLTEdQIFYFPKYGFCeaadqfhvldev

d1jyda_ erdcRVSSFRVKENFDKARFSGTWYAMAKKDPEglflQDNIVAEFSVDeTGQMSATAKGRVRllnnwDVCADMVGTFTDTEDPAKFKMKywgvasflqKGNDDHWIVDTDYDTYAVQYSCRLLnldgtcaDSYSFVFSRDpNGLPPEAQKIVRQRQEELCLAR-QYRLIVHNGYC------------

d1iw2a_ aspistiqpkanfdaQQFAGTWLLVAVGSAG----------------RAEATTLHVAPQGTAMAVSTFRKldgiCWQVRQLYGD-------------------TGVL---GRFLlqARGARGAVHVVVaETDYqSFAVLYLERAGQLSVKLYARSLpvsdsvlsgfeqrvqeahltedqifyfpkygfceaadqfhvldev

d1kqxa_ --------------pADFNGTWEMLSNDNFEdvmkaldidfatrkiaVHLKQTKVIVQNGDKFETKTLST----FRNYEVNFVIgeefdeqtkgldnrtvktlVKWDgdkLVCV--QKGEKENRGWKQ-WIEG-DLLHLEIHCQDKVCHQVFKKKN---------------------------------------------

d1iw2a_ aspistiqpkanfdAQQFAGTWLLVAVGSAG----------------RAEATTLHVAPQGTaMAVSTFRKldgiCWQVRQL------------------yGDTGVL--GRFLlQARGARgAVHVVVAETDyqSFAVLYLERAGQLSVKLYARSlpvsdsvlsgfeqrvqeahltedqifyfpkygfceaadqfhvldev

d1liba_ --------------CDAFVGTWKLVSSENFDdymkevgvgfatrkvaGMAKPNMIISVNGDlVTIRSEST----FKNTEISfklgvefdeitaddrkvksIITLDGgaLVQV-QKWDGK-STTIKRKRDG--DKLVVECVMKGVTSTRVYERA----------------------------------------------

d1iw2a_ aspistiqpkanfdaqQFAGTWLLVAVGSAG----------------RAEATTLHVAPQGTAMAVSTFRKldgiCWQVRQL--------------------yGDTGVL--GRFLlQARGaRGAVHVVVaETDYqSFAVLYLERAGQLSVKLYARSlpvsdsvlsgfeqrvqeahltedqifyfpkygfceaadqfhvldev

d1lpja_ --------------paDLSGTWTLLSSDNFEgymlalgidfatrkiaKLLKPQKVIEQNGDSFTIHTNSS----LRNYFVKfkvgeefdednrgldnrkcksLVIWDNdrLTCI-QKGE-KKNRGWTH-WIEG-DKLHLEMFCEGQVCKQTFQRA----------------------------------------------

d1iw2a_ ---------ASPISTIQ--PKANF--DAQQFAGTWLLVAVGSAG---rAEATTLHVAPQGTAMAVSTFRKL-DGICWQVRQLYGDT--GVLGrFLLQargARGAVHVVVAETDYQSFAVLYLERA-----GQLSVKLYARSLPVSDSVLSGFEQRVQEAHLTEDQIFYFPK--YGFC--EAADqfhvldev

d1obqa_ dkipdfvvpGKCASVDRnkLWAEQtpNRNSYAGVWYQFALTNNPyqliEKCVRNEYSFDGKQFVIESTGIAyDGNLLKRNGKLYPNpfGEPH-LSID-yeNSFAAPLVILETDYSNYACLYSCIDynfgyHSDFSFIFSRSANLADQYVKKCEAAFKNINVDTTRFVKTVQgsSCPYdtQKTL--------

d1iw2a_ -----------aspistiqpkANFDAQQFAGTWLLVA------------------------------vgsagraeaTTLHVAPqgtaMAVSTFRKldgiCWQVRQLYGDTGV------------lGRFLLQARGargaVHVVVAE----tdYQSFAVLYLERAG-------qlSVKLYAR-SLPV--SDSVLSGfeqrvqeahltedqifyfpkygfceaadqfhvldev

d1oeja_ gkplteveqkaangvfddanvQNRTLSDWDGVWQSVYpllqsgkldpvfqkkadadktktfaeikdyyhkgyatdiEMIGIED----GIVEFHRN----NETTSCKYDYDGYkiltyksgkkgvrYLFECKDPEskapKYIQFSDhiiaprKSSHFHIFMGNDSqqsllnemeNWPTYYPyQLSSeeVVEEMMS-----------------------------------h

d1iw2a_ aspistiqpkanfdaQQFAGTWLLVAVGSAG----------------rAEATTLHVAPQGTAMAVSTFRKldgiCWQVRQLYGD-------------------TGVL---GRFLlqarGARGAVHVVVaETDYqSFAVLYLERAGQLSVKLYARSlpvsdsvlsgfeqrvqeahltedqifyfpkygfceaadqfhvldev

d1opaa_ --------------tKDQNGTWEMESNENFEgymkaldidfatrkiavRLTQTKIIVQDGDNFKTKTNST----FRNYDLDFTVgvefdehtkgldgrnvktlVTWEgntLVCV--qkGEKENRGWKQ-WVEG-DKLYLELTCGDQVCRQVFKKK----------------------------------------------

d1iw2a_ aspistiqpkanfdaqQFAGTWLLVAVGSAG----------------RAEATTLHVAPQGTAMAVSTFRKldgiCWQVRQLYGD----------------TGVL----GRFLlqargarGAVHVVVaETDYqSFAVLYLERAGQLSVKLYARSlpvsdsvlsgfeqrvqeahltedqifyfpkygfceaadqfhvldev

d1p6pa_ ----------------AFNGTWNVYAQENYEnflrtvglpediikvaKDVNPVIEIEQNGNEFVVTSKTP----KQTHSNSFTVgkeseitsmdgkkikvTVQLeggkLICK------sDKFSHIQ-EVNG-DEMVEKITIGSSTLTRKSKRV----------------------------------------------

d1iw2a_ ----------ASPIS--TIQPKANFDAQQFAGTWLLVAVGSAG---RAEATTLHVAPQ-GTAMAVSTFRKL--DGICWQVRQLYGDTGV--LGRFLLQargaRGAVHVVVAETD-YQSFAVLYLEragQLSVKLYARSLPVSDSVLSGFEQRVQEAHLTEDQIFYFPKYgfceaadqfhvldev

d1qwda_ hlestslykkSSSTPprGVTVVNNFDAKRYLGTWYEIARFDHRferGLEKVTATYSLRdDGGLNVINKGYNpdRGMWQQSEGKAYFTGAptRAALKVS-ffgPFYGGYNVIALDrEYRHALVCGP--dRDYLWILSRTPTISDEVKQEMLAVATREGFDVSKFIWVQQP--------------g

d1iw2a_ aspistiqpkanfdaqqfagtwllvavgsagraeattlhvapqgTAMAVSTFRKLDGICWQVRQLyGDTGVL-------GRFLLQArgARGAVHVVVAETDyqSFAVLYLERAgQLSVKLYARSLpvsdsvlsgfeqrvqeahltedQIFY----------------------FPKYGFCEAadqfhvldev

d1r0ua_ --------------------------------gfqsnamkqetpITLHVKSVIEDDGNQEVIEFR-TTGFYYvkqnkvyLSYYEEH--DLGKVKTIVKVSE--GEVLVMRSGA-VKMNQRFVTGA----------stiakykmsfgeLELKtstksiqsdldeekgrisiaydMHVGHLHNM--tityeggt

d1iw2a_ aspistiqpkanfdaqQFAGTWLLVAVGSAG----------------RAEATTLHVAPQGTAMAVSTFRKldgiCWQVRQL---------------ygdTGVL-----GRFLlqargarGAVHVVVaETDYqSFAVLYLERAGQLSVKLYARSlpvsdsvlsgfeqrvqeahltedqifyfpkygfceaadqfhvldev

d1tvqa_ ----------------AFSGTWQVYAQENYEeflkalalpedlikmaRDIKPIVEIQQKGDDFVVTSKTP----RQTVTNSftlgkeadittmdgkklkCTVHlangkLVTK------sEKFSHEQ-EVKG-NEMVETITFGGVTLIRRSKRV----------------------------------------------

d1iw2a_ -----------------------------------------------------------------------------------------------------aspISTI----------------------------------------qpkanfdaQQFAGTWLLVAV--------------------------gsagraeaTTLHVAPQGTAMAVSTFRKldgicWQVRQL------------------yGDTGVL---GRFLLqARGA-RGAVHVVVaETDY---QSFAVLYLERAGQLSVKLYARS-lpvsdsvlsgfeqrvqeahltedqifyfpkygfceaadqfhvldev

d1vpra1 ekgfeagdnklggalnakhvekygdnfkngxhkpefhedglhkpxevggkkfesgfhyllechelggknasggyggplcedpygsevqaxtekllkeadsdrtlCFNNfqdpcpqltkeqvaxckgfdygdktlklpcgplpwpaglpepgyvpktNPLHGRWITVSGgqaafikeaiksgmlgaaeankivadtdhhqtggXYLRINQFGDVCTVDASVA---kfARAKRTwksghyfyeplvsggnllgVWVLPEeyrKIGFF-WEXEsGRCFRIER-RAFPvgpYTFXRQATEVGGKISFVFYVKVsndpesdpiplqsrdytalagrdnaptnlgkpyptlakdldypkkrd

d1iw2a_ aspistiqpkanfdaQQFAGTWLLVAVGSAG----------------RAEA--TTLHVAPQGTAMAVSTFRKldgiCWQVRQL-----------------ygDTGVL----GRFLlQARG--ARGAVHVVVaETDYQSFAVLYLERAGQLSVKLYARSlpvsdsvlsgfeqrvqeahltedqifyfpkygfceaadqfhvldev

d1xcaa_ ---------------PNFSGNWKIIRSENFEellkvlgvnvmlrkiaVAAAskPAVEIKQEGDTFYIKTSTT----VRTTEINfkvgeefeeqtvdgrpcksLVKWEsenkMVCE-QKLLkgEGPKTSWTM-ELTNDGELILTMTADDVVCTRVYVRE----------------------------------------------

d1iw2a_ aspistiqpkanfdaqQFAGTWLLVAVGSagraeATTLHVAPQ-GTAMAVSTFRklDGICWQVRQLYGDTGVLGRFLLQArgarGAVHVVVAETDYQSFAVLYLERA--GQLSVKLYARS-LPVSDsVLSGFEQRVQEAHLTEDQIFYFPKYgfceaadqfhvldev

d1xkia_ ----------------DVSGTWYLKAMTV-nlesVTPMTLTTLeGGNLEAKVTM--SGRCQEVKAVLEKTDEPGKYTADG----GKHVAYIIRSHVKDHYIFYSEGEgkPVRGVKLVGRDpKNNLE-ALEDFEKAAGARGLSTESILIPRQS---------------

d1iw2a_ aspisTIQPKANFDAQQFAGTWLLVAVGSAG---------raeaTTLHVAPQ-GTAMAVSTFRKLDGICWQVRQLYGDTGVLGRFLLQargarGAVHVVVAETDYQSFAVLYLERAG----QLSVKLYARSLPVSDSVLSGFEQRVQEAHLTedQIFYF---PKYGFCeaadqfhvldev

d1yupa1 ---iiVTQTMKDLDVQKVAGTWYSLAMAASDislldaqsaplrvYVEELKPTpGGDLEILLQKWENGKCAQKKIIAEKTEIPAVFKID---alNENKVLVLDTDYKKYLLFCMENSAepeqSLACQCLVRTPEVDDEAMEKFDKALKALPMH--IRLSFnptQLEEQC----------rv

d1iw2a_ aspistiqpkanfDAQQFAGTWLLvAVGSAGR-----AEATTLHVAP-qGTAMAVSTFRKL--DGIC-WQVRQLYG--------------------dTGVL------GRFL-lqARGA--RGAVHVVVAETDyqSFAVLYLER------AGQLSVKLYARSlpvsdsvlsgfeqrvqeahltedqifyfpkygfceaadqfhvldev

d2a13a1 -----ppvhpfvaPLSYLLGTWRG-QGEGEYPtipsfRYGEEIRFSHsgKPVIAYTQKTWKleSGAPxHAESGYFRprpdgsievviaqstglvevqKGTYnvdeqsIKLKsdlVGNAskVKEISREFELVD--GKLSYVVRXstttnpLQPHLKAILDKL----------------------------------------------

d1iw2a_ aspistiqpkanfdaQQFAGTWLLVAVGSAG----------------RAEATTLHVAPQGTAMAVSTFRKldgiCWQVRQLYGD----------------TGVL-----GRFLlqargarGAVHVVVaETDYqSFAVLYLERAGQLSVKLYARSlpvsdsvlsgfeqrvqeahltedqifyfpkygfceaadqfhvldev

d2f73a1 ---------------MSFSGKYQLQSQENFEafmkaiglpeeliqkgKDIKGVSEIVQNGKHFKFTITAG----SKVIQNEFTVgeeceletmtgekvktVVQLegdnkLVTT------fKNIKSVT-ELNG-DIITNTMTLGDIVFKRISKRI----------------------------------------------

d1iw2a_ aspistiqpkanFDAQQFAGTWL----lvavgsagRAEATTLHVAPQ-GTAMAVSTFRKL---DGICWQVRQLYGDTgVLGRFLLQ---argARGAVHVVvAETDyQSFAVLYLERAG---------------QLSVKLYARSLpvsdsvlsgfeqrvqeahlteDQIFYFpkygfceaadqfhvldev

d2fr2a1 ------dlapalQALSPLLGSWAgrgagkyptirpFEYLEEVVFAHVgKPFLTYTQQTRAvadGKPLHSETGYLRVC-RPGCVELVlahpsgITEIEVGT-YSVT-GDVIELELSTRAdgsiglaptakevtaLDRSYRIDGDE-------lsyslqmravgqplQDHLAA------------vlhrqr

d1iw2a_ aspistiqpkanfDAQQfAGTWLLVAVgsagraeaTTLHVAPQGTAmAVSTFRkldgicWQVRQLYGDT-------gvlGRFLLQArgargAVHVVVaETDY-QSFAVLYLERA--------------------------------GQLSVKLYARSlpvsdsvlsgfeqrvqeahltedqifyfpkYGFC------eaadqfhvldev

d2gc9a1 ----xtktfktldDFLG-THFIYTYDN------gwEYEWYAKNDHT-VDYRIH-ggxvaGRWVTDQKADivxltegiykISWTEPT-----GTDVAL-DFXPnEKKLHGTIFFPkwveehpeitvtyqnehidlxeqsrekyatypKLVVPEFANIT------------yxgdagqnnedviseapyKEXPndirngkyfdqnyhrlnk

d1iw2a_ aspistiqpkANFDAQQFAGTWLLvAVGSAG------rAEATTLHVAPQ-gtaMAVSTFRKldgiCWQVRQL----------------yGDTGVL----GRFL-lqarGARGAVHVVVaETDYQSFAVLYLERA------GQLSVKLYARSlpvsdsvlsgfeqrvqeahltedqifyfpkygfceaadqfhvldev

d2o62a1 --------erPLLQINDLLGEWRG-QAVTIYrdlrppdIYSTTLKIQLDdagrLXQSTSFG----ERTITSTatikgsivlfdqdpekqVQVLLLpdgaSATSplkvqLRQPLFLEAG-WLIQSDLRQRXIRSYndkgewVSLTLVTEERV----------------------------------------------

d1iw2a_ aSPIStIQPKANFDAQQFA--GTWLLVAVGSAG-----RAEATTLHVAPQGTAMAVSTFRKL--DGICWQVRQLYGDTgVLGRFLLQ------------aRGARGAVHVVVAETDyQSFAVLYLER-----AGQLSVKLYARS--LPVSDSVLSGFEQRVQeahlTEDQIFYFP--kYGFCEAadqfhvldev

d2ofmx1 aCTKN-AIAQTGFNKDKYFngDVWYVTDYLDLEpddvpKRYCAALAAGTASGKLKEALYHYDpkTQDTFYDVSELQVE-SLGKYTANfkkvdkngnvkvaVTAGNYYTFTVMYAD-DSSALIHTCLhkgnkDLGDLYAVLNRNkdAAAGDKVKSAVSAATL----EFSKFISTKennCAYDND--slkslltk

d1iw2a_ aspistiQPKA-NFDAQQFAGTWLLVAVGSAG--------raeaTTLHVAPQGTAMAVSTFRKLDGICWQVRQLYGDTGVLGRFLLQArgaRGAVHVVVAETDYQSFAVLYLERA----GQLSVKLYARSLPVSDSVLSGFEQRVQEAHLTEDQIFYFPKYGFCeaadqfhvldev

d2ozqa1 ----eeaSSTGrNFNVEKINGEWHTIILASDKrekiedngnfrlFLEQIHVLEKSLVLKFHTVRDEECSELSMVADKTEKAGEYSVTY---DGFNTFTIPKTDYDNFLMAHLINEkdgeTFQLMGLYGREPDLSSDIKERFAQLCEEHGILRENIIDLSNANRC------------

d1iw2a_ aSPISTIQPKANFDAQQFA--GTWLLVAVGSAG-----RAEATTLHVAPQGTAMAVSTFRKL--DGICWQVRQLYGDTgVLGRFLLQ------------aRGARGAVHVVVAETDyQSFAVLYLER-----AGQLSVKLYARS--LPVSDSVLSGFEQRVQeahlTEDQIFYFPK--YGFCEAAdqfhvldev

d3np1a_ -KCTKNALAQTGFNKDKYFngDVWYVTDYLDLEpddvpKRYCAALAAGTASGKLKEALYHYDpkTQDTFYDVSELQEE-SPGKYTANfkkvekngnvkvdVTSGNYYTFTVMYAD-DSSALIHTCLhkgnkDLGDLYAVLNRNkdTNAGDKVKGAVTAASL----KFSDFISTKDnkCEYDNVS--lkslltk

d1jyda_ erDCRV-SSFRVKENFDKARFS-GTWYAMAkkdpeglflqdnivaefsVDETGqmsatakgrvrllnnwDVCADMVGTFT--DTEDpaKFKMKYWGVAsfLQKGNDDHWIVDTDYDTYAVQYSCRllnldgtcADSYSFVFSRDPNglppeaqkivrqrqeelclarqyrliVHNG---yc

d1avgi_ -aEGDDcSIEKAMGDFKPEEFFnGTWYLAH----gpgvtspavcqkftTSGSK---gftqiveigynkfESNVKFQCNQVdnKNGE--QYSFKCKSSD--NTEFEADFTFISVSYDNFALVCRSI--tftsqpKEDRYLVFERTKS-------------------------dTDPDakeic

d1jyda_ erdcrvssfrvkenfdkaRFSGTWYAMAKKDPE---------------GLFLQDNIVAEFSVDEtGQMSATAKGrvrllnnwdvCADMVG------------------tfTDTED---pAKFKmkywgvasflqkgnDDHWIvDTDYdTYAVQYSCRLLnldgtcaDSYSFVFSRDpnglppeaqkivrqrqeelclarqyrlivhngyc

d1cbia_ -----------------pNFAGTWKMRSSENFDellkalgvnamlrkvAVAAASKPHVEIRQDG-DQFYIKTST---------tVRTTEInfkvgegfeeetvdgrkcrsLPTWEnenkIHCT----qtllegdgpkTYWTR-ELAN-DELILTFGADD-------VVCTRIYVRE----------------------------------

d1jyda_ erdcrvssfrvkenfdKARFSG-TWYAMAKK-DPEGL------------FLQDNIVAEFSVDETGQMSATAKGRvrllnnwdvCADMVGT------------------fTDTEDP---AKFKmkywgvasflqkgNDDHWIvDTDYdTYAVQYSCRLlnldgtcADSYSFVFSRDpnglppeaqkivrqrqeelclarqyrlivhngyc

d1ftpa_ ----------------VKEFAGiKYKLDSQTnFEEYMkaigvgaierkaGLALSPVIELEILDGDKFKLTSKTA---------IKNTEFTfklgeefdeetldgrkvksTITQDGpnkLVHE--------qkgdhPTIIIR-EFSK-EQCVITIKLG-------DLVATRIYKAQ----------------------------------

d1jyda_ erdcrvssfrvkenfdkARFSGTWYAMAKK-------------dpeglflqDNIVAEFSVDeTGQMSATAKGRvrllnnwdvCADMVGTFTDT--------------edpakfkmkywgvasflqkgnddhwiVDTDyDTYAVQYSCRLlnldgtcADSYSFVFSRDpnglppeaqkivrqrqeelclarqyrlivhngyc

d1ggla_ ----------------pPNLTGYYRFVSQKnmedylqalnislavrkiallLKPDKEIEHQ-GNHMTVRTLST---------FRNYTVQFDVGvefeedlrsvdgrkcqtivtweeehlvcvqkgevpnrgwrHWLE-GEMLYLELTAR-------DAVCEQVFRKV---------------------------------h

d1jyda_ erdcrvssfrvkenfdkARFSGTWYAMAKK-DPEGL------------FLQDNIVAEFSVDeTGQMSATAKGRVrllnnwdvcADMVGTF--------------------TDTED--PAKFKmkywgvasflqkgnDDHWIvDTDYdTYAVQYSCRLlnldgtcADSYSFVFSRDpnglppeaqkivrqrqeelclarqyrlivhngyc

d1kqxa_ ----------------pADFNGTWEMLSNDnFEDVMkaldidfatrkiAVHLKQTKVIVQN-GDKFETKTLSTF---------RNYEVNFvigeefdeqtkgldnrtvktLVKWDgdKLVCV-------qkgekenRGWKQ-WIEG-DLLHLEIHCQ-------DKVCHQVFKKK---------------------------------n

d1jyda_ erdcrvssfrvkenfdKARFSGTWYAMAKK-DPEGL------------FLQDNIVAEFSVDETgQMSATAKGRvrllnnwdvCADMVGT------------------fTDTEDP--AKFKmkywgvasflqkgNDDHWIVDTDydTYAVQYSCRLlnldgtcADSYSFVFSRDpnglppeaqkivrqrqeelclarqyrlivhngyc

d1liba_ ----------------CDAFVGTWKLVSSEnFDDYMkevgvgfatrkvAGMAKPNMIISVNGD-LVTIRSEST---------FKNTEISfklgvefdeitaddrkvksIITLDGgaLVQV-------qkwdgkSTTIKRKRDG--DKLVVECVMK-------GVTSTRVYERA----------------------------------

d1jyda_ erdcrvssfrvkenfdkaRFSGTWYAMAKK-DPEGL------------FLQDNIVAEFSVDEtGQMSATAKGRvrllnnwdvCADMVGTF--------------------TDTEDP--AKFKmkywgvasflqkgnDDHWIvDTDYdTYAVQYSCRLlnldgtcADSYSFVFSRDpnglppeaqkivrqrqeelclarqyrlivhngyc

d1lpja_ ----------------paDLSGTWTLLSSDnFEGYMlalgidfatrkiAKLLKPQKVIEQNG-DSFTIHTNSS---------LRNYFVKFkvgeefdednrgldnrkcksLVIWDNdrLTCI-------qkgekknRGWTH-WIEG-DKLHLEMFCE-------GQVCKQTFQRA----------------------------------

d1jyda_ ------erdcRVSSFR---VKENF--DKARFSGTWYAMAKKDPEGLFLQDNIVAEFSVDeTGQMSATAKGRVRLlnnwDVCADMVGTFTD--TEDPaKFKMKYWgvasflQKGNDDHWIVDTDYDTYAVQYSCrLLNL-DGTCadSYSFVFSRDPnGLPPEAQKIVRQRQEELCL-ARQYRLIVH--NGYC------

d1obqa_ dkipdfvvpgKCASVDrnkLWAEQtpNRNSYAGVWYQFALTNNPYQLIEKCVRNEYSFD-GKQFVIESTGIAYD----GNLLKRNGKLYPnpFGEP-HLSIDYE------NSFAAPLVILETDYSNYACLYSC-IDYNfGYHS--DFSFIFSRSA-NLADQYVKKCEAAFKNINVdTTRFVKTVQgsSCPYdtqktl

d1jyda_ ---------erdcrvssfrvkENFDKARFSGTWYAMA---------------------------kkdpeglflqdnIVAEFSVdetgqMSATAKGRvrllnnwdvCADMVGTFTDTED------------pAKFKMKywgvasflQKGN-DDHWIVD----tdYDTYAVQYSCRLlnldgtcadsYSFVFSR-DPNG--LPPEAQKIvrqrqeelclarqyrlivhngyc

d1oeja_ gkplteveqkaangvfddanvQNRTLSDWDGVWQSVYpllqsgkldpvfqkkadadktktfaeikdyyhkgyatdiEMIGIED-----GIVEFHRN---------NETTSCKYDYDGYkiltyksgkkgvrYLFECK------dpESKApKYIQFSDhiiaprKSSHFHIFMGNDsqqsllnemeNWPTYYPyQLSSeeVVEEMMSH-----------------------

d1jyda_ erdcrvssfrvkenfdkARFSGTWYAMAKK-DPEGL------------FLQDNIVAEFSVDEtGQMSATAKGRvrllnnwdvCADMVGTF--------------------TDTED--PAKFKmkywgvasflqkgnDDHWIvDTDYdTYAVQYSCRLlnldgtcADSYSFVFSRDpnglppeaqkivrqrqeelclarqyrlivhngyc

d1opaa_ ----------------tKDQNGTWEMESNEnFEGYMkaldidfatrkiAVRLTQTKIIVQDG-DNFKTKTNST---------FRNYDLDFtvgvefdehtkgldgrnvktLVTWEgnTLVCV-------qkgekenRGWKQ-WVEG-DKLYLELTCG-------DQVCRQVFKKK----------------------------------

d1jyda_ erdcrvssfrvkenfdkaRFSGTWYAMAKKD---PEGL----------FLQDNIVAEFSVDeTGQMSATAKGRvrllnnwdvCADMVGTFTD----------------TEDP----AKFKmkywgvasflqkgnDDHWIvDTDYdTYAVQYSCRLlnldgtcADSYSFVFSRDpnglppeaqkivrqrqeelclarqyrlivhngyc

d1p6pa_ ------------------AFNGTWNVYAQENyenFLRTvglpediikvAKDVNPVIEIEQN-GNEFVVTSKTP---------KQTHSNSFTVgkeseitsmdgkkikvTVQLeggkLICK-----------sdkFSHIQ-EVNG-DEMVEKITIG-------SSTLTRKSKRV----------------------------------

d1jyda_ ----------erdcrVSSFRVKENFDKARFSGTWYAMAKKDPegLFLQ--DNIVAEFSVDETGQMSATAKGRVRLlnnWDVCADMVGTFTDTED--PAKFKMKYWgvasflQKGNDDHWIVDTD-yDTYAVQYSCRllnldgtcaDSYSFVFSRDPnGLPPEAQKIVRQRQEELCL-ARQYRLIVHNgyc

d1qwda_ hlestslykkssstpPRGVTVVNNFDAKRYLGTWYEIARFDH--RFERglEKVTATYSLRDDGGLNVINKGYNPD---RGMWQQSEGKAYFTGAptRAALKVSFF------GPFYGGYNVIALDreYRHALVCGPD---------RDYLWILSRTP-TISDEVKQEMLAVATREGFdVSKFIWVQQP--g

d1jyda_ erdcrvssfrvkenfdkARFSGTWYAmAKKDPE-----glflqDNIVAEFSVDeTGQMSATAKGrVRLLnnwdVCADMVGTFTDtedpakFKMKYWGVAsFLQKGND------------------------dHWIVdtdYDTYAVQYSCRLLNLDgtCADSYSFVFSRDPnglppeaqkivrqrqeelclarqyrlivhngyc

d1r0ua_ -----------gfqsnaMKQETPITL-HVKSVIeddgnqevieFRTTGFYYVK-QNKVYLSYYE-EHDL----GKVKTIVKVSE------GEVLVMRSGaVKMNQRFvtgastiakykmsfgelelktstksIQSD--lDEEKGRISIAYDMHVG--HLHNMTITYEGGT---------------------------------

d1jyda_ erdcrvssfrvkenfdkaRFSGTWYAMAKK-------------dpeglflqdNIVAEFSVDeTGQMSATAKGRvrllnnwdvCADMVGTFTDTedpakfkmKYWGvasfLQKGN-----------------dDHWIvDTDYdTYAVQYSCRLlnldgtcADSYSFVFSRDpnglppeaqkivrqrqeelclarqyrlivhngyc

d1tvqa_ ------------------AFSGTWQVYAQEnyeeflkalalpedlikmardiKPIVEIQQK-GDDFVVTSKTP---------RQTVTNSFTLG----keadITTM----DGKKLkctvhlangklvtksekfSHEQ-EVKG-NEMVETITFG-------GVTLIRRSKRV----------------------------------

d1jyda_ -----------------------------------------------------------------------------------------------erdCRVSSFR--------------------------------------------vkenfdkARFSGTWYAMAK---------------------kdpEGLFL--qdNIVAEFSVDeTGQMSATAKGRVrllnnwdvcADMVGTF------------------TDTEDP---AKFKmkywgvasflqkgnDDHWIvDTDY---DTYAVQYSCRLlnldgtcADSYSFVFSRD-------------pnglppeaqkivrqrqeelclarqyrlivhngyc

d1vpra1 ekgfeagdnklggalnakhvekygdnfkngxhkpefhedglhkpxevggkkfesgfhyllechelggknasggyggplcedpygsevqaxtekllkeaDSDRTLCfnnfqdpcpqltkeqvaxckgfdygdktlklpcgplpwpaglpepgyvpktNPLHGRWITVSGgqaafikeaiksgmlgaaeankivADTDHhqtgGXYLRINQF-GDVCTVDASVAK--------fARAKRTWksghyfyeplvsggnllgVWVLPEeyrKIGF-----fwexesgrcFRIER-RAFPvgpYTFXRQATEVG-------GKISFVFYVKVsndpesdpiplqsrdytalagrdnaptnlgkpyptlakdldypkkrd

d1jyda_ erdcrvssfrvkenfdkARFSGTWYAMAKKDPEGL---------------flQDNIVAEFSVDeTGQMSATAKGRvrllnnwdvCADMVGTF------------------TDTEDP---AKFKmkywgvasflqkgndDHWIvDTDYDTYAVQYSCRLlnldgtcADSYSFVFSRDpnglppeaqkivrqrqeelclarqyrlivhngyc

d1xcaa_ -----------------PNFSGNWKIIRSENFEELlkvlgvnvmlrkiavaaASKPAVEIKQE-GDTFYIKTSTT---------VRTTEINFkvgeefeeqtvdgrpcksLVKWESenkMVCE----qkllkgegpktSWTM-ELTNDGELILTMTAD-------DVVCTRVYVRE----------------------------------

d1jyda_ erdcrvssfrvkenfdkaRFSGTWYAMAKKDpegLFLQdNIVAEFSVDETGQMSATAKgrvrllnnWDVCADMVGTFTDTEDPAKFKMKywgvasflqkgNDDHWIVDTDYDTYAVQYSCRLLNldgtcADSYSFVFSRDPNGLPPeAQKIVRQRQEELCLARQ-YRLIVHNgyc

d1xkia_ ------------------DVSGTWYLKAMTV---NLES-VTPMTLTTLEGGNLEAKVT-------mSGRCQEVKAVLEKTDEPGKYTAD---------ggKHVAYIIRSHVKDHYIFYSEGEGK-----PVRGVKLVGRDPKNNLE-ALEDFEKAAGARGLSTEsILIPRQS---

d1jyda_ erdcrvSSFRVKENFDKARFSGTWYAMAKKDPE--------GLFLqdnIVAEFSVDETGQMSATAKgRVRLlnnwDVCADMVGTFTDTEDPAKFKMKYwgvasflqKGNDDHWIVDTDYDTYAVQYSCRLLNldgTCADSYSFVFSRDPNGLPpEAQKIVRQRQEELCLaRQYRLI---VHNG--yc

d1yupa1 -----iIVTQTMKDLDVQKVAGTWYSLAMAASDislldaqsAPLR--vYVEELKPTPGGDLEILLQ-KWEN----GKCAQKKIIAEKTEIPAVFKIDA--------LNENKVLVLDTDYKKYLLFCMENSAE---PEQSLACQCLVRTPEVDD-EAMEKFDKALKALPM-HIRLSFnptQLEEqcrv

d1jyda_ erdcrvssfrvkenfDKARFSGTWYAmAKKDPE----GLFLQdnIVAEFSVDETGQMSATAKGRVRLlnnWDVC-ADMVGTFTD--------------------TEDP------AKFKmkywgvasflqkgnDDHWIvDTDYdTYAVQYSCRlLNLDGTCADSYSFVFSRDpnglppeaqkivrqrqeelclarqyrlivhngyc

d2a13a1 -------ppvhpfvaPLSYLLGTWRG-QGEGEYptipSFRYG--EEIRFSHSGKPVIAYTQKTWKLE---SGAPxHAESGYFRPrpdgsievviaqstglvevqKGTYnvdeqsIKLK--sdlvgnaskvkeISREF-ELVD-GKLSYVVRX-STTTNPLQPHLKAILDKL----------------------------------

d1jyda_ erdcrvssfrvkenfdkARFSGTWYAMAKK-DPEGL------------FLQDNIVAEFSVDeTGQMSATAKGRVrllnnwdvcADMVGTFTD----------------TEDP-----AKFKmkywgvasflqkgnDDHWIvDTDYdTYAVQYSCRLlnldgtcADSYSFVFSRDpnglppeaqkivrqrqeelclarqyrlivhngyc

d2f73a1 -----------------MSFSGKYQLQSQEnFEAFMkaiglpeeliqkGKDIKGVSEIVQN-GKHFKFTITAGS---------KVIQNEFTVgeeceletmtgekvktVVQLegdnkLVTT-----------fknIKSVT-ELNG-DIITNTMTLG-------DIVFKRISKRI----------------------------------

d1jyda_ erdcrvssfrvkenfDKARFSGTWYAmAKKDPE--glflqDNIVAEFSVDETGQMSATAKGRVrllnnwDVCADMVGTFTDTEDPakFKMKYWGVASflQKGNDD----------------------------------hWIVDTDyDTYAVQYSCrLLNLDGTCADSYSFVFSRDpnglppeaqkivrqrqeelclarqyrlivhngyc

d2fr2a1 --------dlapalqALSPLLGSWAG-RGAGKYptirpfeYLEEVVFAHVGKPFLTYTQQTRA--vadgKPLHSETGYLRVCRPG-cVELVLAHPSG--ITEIEVgtysvtgdvielelstradgsiglaptakevtaldRSYRID-GDELSYSLQ-MRAVGQPLQDHLAAVLHRQ---------------------------------r

d1jyda_ erdcrvssfrvkENFD--KARFS--------------------GTWYAmaKKDPEGL--flqdnIVAEFSVDETGQMSATAKGRvrllnnwdVCADMVGTFTDTedpakFKMKYWGVA---------------------------------SFLQKGNDDH-wIVDTDYDTyavqyscrllnldgtcadsysfvfsrdpnglppeaqkivrqrQEELCLArqyrlivhngyc

d2gc9a1 -----------xTKTFktLDDFLgthfiytydngweyewyaknDHTVD-yRIHGGXVagrwvtdQKADIVXLTEGIYKISWTEP--------TGTDVALDFXPN----eKKLHGTIFFpkwveehpeitvtyqnehidlxeqsrekyatypKLVVPEFANItyXGDAGQNN-----------------------------edviseapykexpNDIRNGK-yfdqnyhrlnk

d1jyda_ erdcrvssfrvkENFDKARFSGTWYAmAKKDPE-----GLFLqdNIVAEFSVDETGqMSATAKGRVrllnnwdvcADMVGT----------------fTDTEDP----AKFKmkywgvasflqkgnDDHWIvDTDYDTYAVQYSCRlLNLDGTCADSYSFVFSRDpnglppeaqkivrqrqeelclarqyrlivhngyc

d2o62a1 ----------erPLLQINDLLGEWRG-QAVTIYrdlrpPDIY--STTLKIQLDDAGrLXQSTSFGE---------RTITSTatikgsivlfdqdpekqVQVLLLpdgaSATS----plkvqlrqplFLEAG-WLIQSDLRQRXIRS-YNDKGEWVSLTLVTEERV----------------------------------

d1jyda_ erdcrVSSFRVKENFDKARFS--GTWYAMAKKDP--EGLFlQDNIVAEFSVDETgQMSATAKGRVrllnnWDVCADMVGTFTDTeDPAKFKMKYWGVA----SFLQK---GNDDHWIVDTDyDTYAVQYSCRllnldgtcaDSYSFVFSRDPNGLppeAQKIVRQRQEELCLA-RQYRLIV-HNGYC-----------

d2ofmx1 ---acTKNAIAQTGFNKDKYFngDVWYVTDYLDLepDDVPkRYCAALAAGTASG-KLKEALYHYD---pkTQDTFYDVSELQVE-SLGKYTANFKKVDkngnVKVAVtagNYYTFTVMYAD-DSSALIHTCL--hkgnkdlGDLYAVLNRNKDAA---AGDKVKSAVSAATLEfSKFISTKeNNCAYdndslkslltk

d1jyda_ erdcrvssfrVKEN--FDKARFSGTWYAMAKKD-PEGL-----FLQDnIVAEFSVDeTGQMSATAKGRvrllnnWDVCADMVGTFTDTEDPAKFKMKYwgvasflqKGNDDHWIVDTDYDTYAVQYSCRLLNLdgtcADSYSFVFSRDPnGLPPEAQKIVRQRQEELCLAR-QYRLI--VHNGyc

d2ozqa1 -------eeaSSTGrnFNVEKINGEWHTIILASdKREKiedngNFRL-FLEQIHVL-EKSLVLKFHTV-----rDEECSELSMVADKTEKAGEYSVTY--------DGFNTFTIPKTDYDNFLMAHLINEKDG---eTFQLMGLYGREP-DLSSDIKERFAQLCEEHGILReNIIDLsnANRC--

d1jyda_ erdcrVSSFRVKENFDKARFS--GTWYAMAKKDPEG--lflQDNIVAEFSVDETgQMSATAKGRVrllnnWDVCADMVGTFTDTeDPAKFKMKYWGVA----SFLQK---GNDDHWIVDTDyDTYAVQYSCRLlnldgtcADSYSFVFSRDPNGLppeAQKIVRQRQEELCLA-RQYRLIV-HNGYC-----------

d3np1a_ ---kcTKNALAQTGFNKDKYFngDVWYVTDYLDLEPddvpkRYCAALAAGTASG-KLKEALYHYD---pkTQDTFYDVSELQEE-SPGKYTANFKKVEkngnVKVDVtsgNYYTFTVMYAD-DSSALIHTCLH--kgnkdLGDLYAVLNRNKDTN---AGDKVKGAVTAASLKfSDFISTKdNKCEYdnvslkslltk

d1kqxa_ padfngtwemlsNDNFedvmkaldidfatrkiavhlkqtkvIVQN------------------------GDKFETKTLStfrnyevnfvigeefdeqtkGLDN--RTVKTLVKW--DGDKLVCVQKG-EKENRGWKQWIEGD------LLHLEIHCQ-DKVCHQVFKKKN-------------

d1avgi_ ------------AEGD----------dcsiekamgdfkpeeFFNGtwylahgpgvtspavcqkfttsgsKGFTQIVEIG-------------------yNKFEsnVKFQCNQVDnkNGEQYSFKCKSsDNTEFEADFTFISVsydnfaLVCRSITFTsQPKEDRYLVFERtksdtdpdakeic

d1kqxa_ pADFNGTWEMLSNDNFEDVMKALDIDFATRKIAVHLKQTKVIVQNGDKFETKTLSTFRNYEVNFVIGEEFDEQTkGLDNrTVKTLVKWDGDKLVCVQKGEKENRGWKQWIEGDLLHLEIHCQDKVCHQVFKKKn

d1liba_ cDAFVGTWKLVSSENFDDYMKEVGVGFATRKVAGMAKPNMIISVNGDLVTIRSESTFKNTEISFKLGVEFDEIT-ADDR-KVKSIITLDGGALVQVQKWDGKSTTIKRKRDGDKLVVECVMKGVTSTRVYERA-

d1kqxa_ PADFNGTWEMLSNDNFEDVMKALDIDFATRKIAVHLKQTKVIVQNGDKFETKTLSTFRNYEVNFVIGEEFDEQTKGLDNRTVKTLVKWDGDKLVCVQKGEKENRGWKQWIEGDLLHLEIHCQDKVCHQVFKKKn

d1lpja_ PADLSGTWTLLSSDNFEGYMLALGIDFATRKIAKLLKPQKVIEQNGDSFTIHTNSSLRNYFVKFKVGEEFDEDNRGLDNRKCKSLVIWDNDRLTCIQKGEKKNRGWTHWIEGDKLHLEMFCEGQVCKQTFQRA-

d1kqxa_ -------------------------paDFNGTWEMLS--------------ndnfedvmkaldidfatrkiavhLKQTKVIVQngdkFETKTLSTFRNYEVNFVIgeefdeqtkgldnrtvktlVKWDG---------DKLVCVQK--gekenrGWKQWIE------GDLLHLEIHCQ-------dkvCHQVFKKKN-------------

d1oeja_ gkplteveqkaangvfddanvqnrtlsDWDGVWQSVYpllqsgkldpvfqkkadadktktfaeikdyyhkgyatDIEMIGIED----GIVEFHRNNETTSCKYDY-------------------DGYKIltyksgkkgVRYLFECKdpeskapkYIQFSDHiiaprkSSHFHIFMGNDsqqsllnemeNWPTYYPYQlsseevveemmsh

d1kqxa_ PADFNGTWEMLSNDNFEDVMKALDIDFATRKIAVHLKQTKVIVQNGDKFETKTLSTFRNYEVNFVIGEEFDEQTKGLDNRTVKTLVKWDGDKLVCVQKGEKENRGWKQWIEGDLLHLEIHCQDKVCHQVFKKKn

d1opaa_ TKDQNGTWEMESNENFEGYMKALDIDFATRKIAVRLTQTKIIVQDGDNFKTKTNSTFRNYDLDFTVGVEFDEHTKGLDGRNVKTLVTWEGNTLVCVQKGEKENRGWKQWVEGDKLYLELTCGDQVCRQVFKKK-

d1kqxa_ paDFNGTWEMLSNDNFEDVMKALDIDFATRKIAVHLKQTKVIVQNGDKFETKTLSTFRNYEVNFVIGEEFDEQTkgLDNRTVKTLVKWDGDKLVCVQKgekeNRGWKQWIEGDLLHLEIHCQDKVCHQVFKKKn

d1p6pa_ --AFNGTWNVYAQENYENFLRTVGLPEDIIKVAKDVNPVIEIEQNGNEFVVTSKTPKQTHSNSFTVGKESEITS--MDGKKIKVTVQLEGGKLICKSD----KFSHIQEVNGDEMVEKITIGSSTLTRKSKRV-

d1kqxa_ -----------------------------------------padfNGTWEMlsndnfedvmkaldidfatrkiAVHLKQTKVIVQNGDKFETKTLStFRNYEVNFVIGEEFDEQTKG-lDNRTVKTLVKW---------dgDKLVCVQKGEkenRGWKQWIEGDllhleihcqdkvchqvfkkkn

d1r0ua_ gfqsnamkqetpitlhvksvieddgnqeviefrttgfyyvkqnkvYLSYYE---------------------eHDLGKVKTIVKVSEGEVLVMRSG-AVKMNQRFVTGASTIAKYKMsfGELELKTSTKSiqsdldeekgrISIAYDMHVG---HLHNMTITYE------------------ggt

d1kqxa_ paDFNGTWEMLSNDNFEDVMKALDIDFATRKIAVHLKQTKVIVQNGDKFETKTLSTFRNYEVNFVIGEEFDEQTkGLDNrTVKTLVKWDGDKLVCVQKgekeNRGWKQWIEGDLLHLEIHCQDKVCHQVFKKKn

d1tvqa_ --AFSGTWQVYAQENYEEFLKALALPEDLIKMARDIKPIVEIQQKGDDFVVTSKTPRQTVTNSFTLGKEADITT-MDGK-KLKCTVHLANGKLVTKSE----KFSHEQEVKGNEMVETITFGGVTLIRRSKRV-

d1kqxa_ -----------------------------------------------------------------------------------------------------------------------------------------------------------PADFNGTWEMLSN-------dnfedvmkaldiDFATR---kiavhlKQTKVIVQNGDKFETKTLsTFRN--YEVNFVIGEEFDEQTKglDNRTVKTLVKWDGD-KLVCVQKGE--KENRGWKQWIEG----DLLHLEIHCQDKVCHQVFKKKN----------------------------------------------

d1vpra1 ekgfeagdnklggalnakhvekygdnfkngxhkpefhedglhkpxevggkkfesgfhyllechelggknasggyggplcedpygsevqaxtekllkeadsdrtlcfnnfqdpcpqltkeqvaxckgfdygdktlklpcgplpwpaglpepgyvpkTNPLHGRWITVSGgqaafikeaiksgmlgaaeANKIVadtdhhqtgGXYLRINQFGDVCTVDAS-VAKFarAKRTWKSGHYFYEPLV--SGGNLLGVWVLPEEyRKIGFFWEXesGRCFRIERRAFPvgpyTFXRQATEVGGKISFVFYVKVSndpesdpiplqsrdytalagrdnaptnlgkpyptlakdldypkkrd

d1kqxa_ pADFNGTWEMLSNDNFEDVMKALDIDFATRKIAVH--LKQTKVIVQNGDKFETKTLSTFRNYEVNFVIGEEFDEQTkgLDNRTVKTLVKWDG-DKLVCVQKG---EKENRGWKQWIE-GDLLHLEIHCQDKVCHQVFKKKn

d1xcaa_ -PNFSGNWKIIRSENFEELLKVLGVNVMLRKIAVAaaSKPAVEIKQEGDTFYIKTSTTVRTTEINFKVGEEFEEQT--VDGRPCKSLVKWESeNKMVCEQKLlkgEGPKTSWTMELTnDGELILTMTADDVVCTRVYVRE-

d1kqxa_ ---------PADFNGTWEMlSNDNFEdvmkaldidFATRkiaVHLKQTKVIVQN-GDKFETKTLS-------TFRNYEVNFVI----GEEFDEQTKglDNRTVKTLVKWDG--DKLVCVQKGE-----KENRGWKQWIEGDLLHLEIHCQ-----DKVC-HQVFKKKn

d2a13a1 ppvhpfvapLSYLLGTWRG-QGEGEY--------pTIPS---FRYGEEIRFSHSgKPVIAYTQKTwklesgaPXHAESGYFRPrpdgSIEVVIAQS--TGLVEVQKGTYNVdeQSIKLKSDLVgnaskVKEISREFELVDGKLSYVVRXStttnpLQPHlKAILDKL-

d1kqxa_ pADFNGTWEMLSNDNFEDVMKALDIDFATRKIAVHLKQTKVIVQNGDKFETKTLstFRNYEVNFVIGEEFDEQTkgLDNRTVKTLVKWDG-DKLVCVQKgekeNRGWKQWIEGDLLHLEIHCQDKVCHQVFKKKn

d2f73a1 -MSFSGKYQLQSQENFEAFMKAIGLPEELIQKGKDIKGVSEIVQNGKHFKFTITagSKVIQNEFTVGEECELET--MTGEKVKTVVQLEGdNKLVTTFK----NIKSVTELNGDIITNTMTLGDIVFKRISKRI-

d1kqxa_ --------PADFNGTWEMlSNDNFEdvmkaldidFATRkiaVHLKQTKVIVQN-GDKFETKTLS-------TFRNYEVNFVI----GEEFDEQTKglDNRTVKTLVKWDG----DKLVCV----QKGE-------KENRGWKQWIEGDLLHLEIHCQ-----DKVC-HQVFKKKN

d2fr2a1 dlapalqaLSPLLGSWAG-RGAGKY--------pTIRP---FEYLEEVVFAHVgKPFLTYTQQTravadgkPLHSETGYLRVcrpgCVELVLAHP--SGITEIEVGTYSVtgdvIELELStradGSIGlaptakeVTALDRSYRIDGDELSYSLQMRavgqpLQDHlAAVLHRQR

d1kqxa_ ----------PADFNGTWEMLSndnfedvmkaldidfatrkiavhlKQTKVIVQNG-DKFETKTL-STFRNYEVNFVIgeefdeqtkgldnrtvktlVKWDG-----DKLVCvqkgekenrgWKQWIE--GDLLHLEIHCQ--------------------------------DKVCHQVFKKKN---------------------------------------

d2gc9a1 xtktfktlddFLGTHFIYTYDN------------------------GWEYEWYAKNdHTVDYRIHgGXVAGRWVTDQK-------------------ADIVXltegiYKISW---teptgtdVALDFXpnEKKLHGTIFFPkwveehpeitvtyqnehidlxeqsrekyatypKLVVPEFANITYxgdagqnnedviseapykexpndirngkyfdqnyhrlnk

d1kqxa_ ------pADFNGTWEMlSNDNFEdvmkaldidfatrkiaVHLKQTKVIVQN-gdKFETKTLSTFRNYEVNFV----IGEEFDEQTkgldnrtvKTLVKWDG-DKLVCVQKGE---KENRGWKQWIEG-DLLHLEIHCQ------DKVCHQVFKKKn

d2o62a1 erpllqiNDLLGEWRG-QAVTIY----------rdlrppDIYSTTLKIQLDdagRLXQSTSFGERTITSTATikgsIVLFDQDPE-------kQVQVLLLPdGASATSPLKVqlrQPLFLEAGWLIQsDLRQRXIRSYndkgewVSLTLVTEERV-

d1liba_ -------------cDAFVGtwklvssenfddymkevgvgFATRkvagmakpnmiisvngDLVTIRsestfknteisfklgvefdeitADDRKVKSIITL--DGGALVQVQKW-DGKSTTIKRKRDGD------KLVVECVM-KGVTSTRVYE--------------ra

d1avgi_ aegddcsiekamgdFKPEE-------ffngtwylahgpgVTSP------avcqkfttsgSKGFTQ-------------iveigynkfESNVKFQCNQVDnkNGEQYSFKCKSsDNTEFEADFTFISVsydnfaLVCRSITFtSQPKEDRYLVfertksdtdpdakeic

d1liba_ CDAFVGTWKLVSSENFDDYMKEVGVGFATRKVAGMAKPNMIISVNGDLVTIRSESTFKNTEISFKLGVEFDEIT--ADDRKVKSIITLDGGALVQVQKWDGKSTTIKRKRDGDKLVVECVMKGVTSTRVYERA

d1lpja_ PADLSGTWTLLSSDNFEGYMLALGIDFATRKIAKLLKPQKVIEQNGDSFTIHTNSSLRNYFVKFKVGEEFDEDNrgLDNRKCKSLVIWDNDRLTCIQKGEKKNRGWTHWIEGDKLHLEMFCEGQVCKQTFQRA

d1liba_ -------------------------CDAFVGTWKLVS-----------------------------------SEnfddymkevgvgfatrkvagmaKP-NMIISVngdlVTIRSESTFKNTeisfklgvefdeitaddrkvkSIITLDG---------GALVQVQKWD--gksTTIKRKRD------gDKLVVECVMK-------gvtSTRVYERA--------------

d1oeja_ gkplteveqkaangvfddanvqnrtLSDWDGVWQSVYpllqsgkldpvfqkkadadktktfaeikdyyhkgyAT----------------------DIeMIGIED----GIVEFHRNNETT-----------------sckyDYDGYKIltyksgkkgVRYLFECKDPeskapKYIQFSDHiiaprksSHFHIFMGNDsqqsllnemeNWPTYYPYqlsseevveemmsh

d1liba_ CDAFVGTWKLVSSENFDDYMKEVGVGFATRKVAGMAKPNMIISVNGDLVTIRSESTFKNTEISFKLGVEFDEIT-ADDR-KVKSIITLDGGALVQVQKWDGKSTTIKRKRDGDKLVVECVMKGVTSTRVYERA

d1opaa_ TKDQNGTWEMESNENFEGYMKALDIDFATRKIAVRLTQTKIIVQDGDNFKTKTNSTFRNYDLDFTVGVEFDEHTkGLDGrNVKTLVTWEGNTLVCVQKGEKENRGWKQWVEGDKLYLELTCGDQVCRQVFKKK

d1liba_ cdAFVGTWKLVSSENFDDYMKEVGVGFATRKVAGMAKPNMIISVNGDLVTIRSESTFKNTEISFKLGVEFDEITADDRKVKSIITLDGGALVQVQKwdgkSTTIKRKRDGDKLVVECVMKGVTSTRVYERA

d1p6pa_ --AFNGTWNVYAQENYENFLRTVGLPEDIIKVAKDVNPVIEIEQNGNEFVVTSKTPKQTHSNSFTVGKESEITSMDGKKIKVTVQLEGGKLICKSD----KFSHIQEVNGDEMVEKITIGSSTLTRKSKRV

d1liba_ -----------------------------------------cdafVGTWKLvssenfddymkevgvgfatrkvAGMAKPNMIISVNGDLVTIRSEStFKNTEISFKLGVEFDEITAD---DRKVKSII-TLDG--------gALVQVQKWDgksTTIKRKRDGDklvvecvmkgvtstrvyera

d1r0ua_ gfqsnamkqetpitlhvksvieddgnqeviefrttgfyyvkqnkvYLSYYE---------------------eHDLGKVKTIVKVSEGEVLVMRSG-AVKMNQRFVTGASTIAKYKMsfgELELKTSTkSIQSdldeekgriSIAYDMHVG---HLHNMTITYE-----------------ggt

d1liba_ cdAFVGTWKLVSSENFDDYMKEVGVGFATRKVAGMAKPNMIISVNGDLVTIRSESTFKNTEISFKLGVEFDEITADDRKVKSIITLDGGALVQVQKwdgkSTTIKRKRDGDKLVVECVMKGVTSTRVYERA

d1tvqa_ --AFSGTWQVYAQENYEEFLKALALPEDLIKMARDIKPIVEIQQKGDDFVVTSKTPRQTVTNSFTLGKEADITTMDGKKLKCTVHLANGKLVTKSE----KFSHEQEVKGNEMVETITFGGVTLIRRSKRV

d1liba_ -----------------------------------------------------------------------------------------------------------------------------------------------------------cDAFVGTWKLVSS--------enfddymkevgvGFATR--kvagmaKPNMIISVNGDLVTIRSEsTFKN--TEISFKLGVEFDEITADDRKVKSIITLDGG-ALVQVQKW--DGKSTTIKRKRDG----DKLVVECVMKGVTSTRVYERA-----------------------------------------------

d1vpra1 ekgfeagdnklggalnakhvekygdnfkngxhkpefhedglhkpxevggkkfesgfhyllechelggknasggyggplcedpygsevqaxtekllkeadsdrtlcfnnfqdpcpqltkeqvaxckgfdygdktlklpcgplpwpaglpepgyvpktNPLHGRWITVSGgqaafikeaiksgmlgaaeaNKIVAdtdhhqtgGXYLRINQFGDVCTVDAS-VAKFarAKRTWKSGHYFYEPLVSGGNLLGVWVLPEEyRKIGFFWExeSGRCFRIERRAFPvgpyTFXRQATEVGGKISFVFYVKVsndpesdpiplqsrdytalagrdnaptnlgkpyptlakdldypkkrd

d1liba_ cDAFVGTWKLVSSENFDDYMKEVGVGFATRKVAGMA--KPNMIISVNGDLVTIRSESTFKNTEISFKLGVEFDEITADDRKVKSIITLDG-GALVQVQKW---DGKSTTIKRKRD-GDKLVVECVMKGVTSTRVYERA

d1xcaa_ -PNFSGNWKIIRSENFEELLKVLGVNVMLRKIAVAAasKPAVEIKQEGDTFYIKTSTTVRTTEINFKVGEEFEEQTVDGRPCKSLVKWESeNKMVCEQKLlkgEGPKTSWTMELTnDGELILTMTADDVVCTRVYVRE

d1liba_ ---------CDAFVGTWKLvSSENFDdymkevgvgfatrkvaGMAKPNMIISVN-GDLVTIRSES-------TFKNTEISFKL----GVEFDEITADDRKVKSIITLD--GGALVQVQKW-----dgKSTTIKRKRDGDKLVVECVM------KGVTSTRVYERA

d2a13a1 ppvhpfvapLSYLLGTWRG-QGEGEY-----------ptipsFRYGEEIRFSHSgKPVIAYTQKTwklesgaPXHAESGYFRPrpdgSIEVVIAQSTGLVEVQKGTYNvdEQSIKLKSDLvgnaskvKEISREFELVDGKLSYVVRXstttnpLQPHLKAILDKL

d1liba_ cDAFVGTWKLVSSENFDDYMKEVGVGFATRKVAGMAKPNMIISVNGDLVTIRSEstFKNTEISFKLGVEFDEITADDRKVKSIITLDG-GALVQVQKwdgkSTTIKRKRDGDKLVVECVMKGVTSTRVYERA

d2f73a1 -MSFSGKYQLQSQENFEAFMKAIGLPEELIQKGKDIKGVSEIVQNGKHFKFTITagSKVIQNEFTVGEECELETMTGEKVKTVVQLEGdNKLVTTFK----NIKSVTELNGDIITNTMTLGDIVFKRISKRI

d1liba_ --------CDAFVGTWKLvSSENFDDymkevgvgfatrkvaGMAKPNMIISVN-GDLVTIRSES-------TFKNTEISFKL----GVEFDEITADDRKVKSIITLDG----GALVQVQ----KWDG-------KSTTIKRKRDGDKLVVECVM------KGVTSTRVYERA-

d2fr2a1 dlapalqaLSPLLGSWAG-RGAGKYP-----------tirpFEYLEEVVFAHVgKPFLTYTQQTravadgkPLHSETGYLRVcrpgCVELVLAHPSGITEIEVGTYSVtgdvIELELSTradgSIGLaptakevTALDRSYRIDGDELSYSLQMravgqpLQDHLAAVLHRQr

d1liba_ ----------CDAFVGTWKLVSsenfddymkevgvgfatrkvagmaKPNMIIS-VNGDLVTIRSES-tfKNTEISFklgvefdeitaddrkvksiITLDG-----GALVQVQKwdgKSTTIKRKRD--GDKLVVECVMK--------------------------------GVTSTRVYERA----------------------------------------

d2gc9a1 xtktfktlddFLGTHFIYTYDN------------------------GWEYEWYaKNDHTVDYRIHGgxvAGRWVTD-----------------qkADIVXltegiYKISWTEP---TGTDVALDFXpnEKKLHGTIFFPkwveehpeitvtyqnehidlxeqsrekyatypKLVVPEFANITyxgdagqnnedviseapykexpndirngkyfdqnyhrlnk

d1liba_ ------CDAFVGTWKLvSSENFDdymkevgvgfatrkvaGMAKPNMIISVN-GDLVTIRSESTFKNTEISFK----LGVEFDEITaddrkvKSIITLDG-GALVQVQ-KWDG--KSTTIKRKRDGD-KLVVECVM------KGVTSTRVYERA

d2o62a1 erpllqINDLLGEWRG-QAVTIY----------rdlrppDIYSTTLKIQLDdAGRLXQSTSFGERTITSTATikgsIVLFDQDPE-----kQVQVLLLPdGASATSPlKVQLrqPLFLEAGWLIQSdLRQRXIRSyndkgeWVSLTLVTEERV

d1lpja_ padlsgtwtllsSDNFegymlalgidfatrkiakllkpqkviEQNG---dsftihtNSSLRN--yfvkfKVGE-----efdednrGLDN--RKCKSLVIW--DNDRLTCIQKG-EKKNRGWTHWIEGD------KLHLEMFCE-GQVCKQTFQRA--------------

d1avgi_ ------------AEGD---------------dcsiekamgdfKPEEffngtwylahGPGVTSpavcqkfTTSGskgftqiveigyNKFEsnVKFQCNQVDnkNGEQYSFKCKSsDNTEFEADFTFISVsydnfaLVCRSITFTsQPKEDRYLVFErtksdtdpdakeic

d1lpja_ -------------------------paDLSGTWTLLS--------------sdnfegymlalgidfatrkiaklLKPQKVIEQngdsFTIHTNSSLRNYFVKFKVgeefdednrgldnrkckslVIWDN---------DRLTCI--qkgekknRGWTHWIE------gDKLHLEMFCE-------gqvCKQTFQRA--------------

d1oeja_ gkplteveqkaangvfddanvqnrtlsDWDGVWQSVYpllqsgkldpvfqkkadadktktfaeikdyyhkgyatDIEMIGIED----GIVEFHRNNETTSCKYDY-------------------DGYKIltyksgkkgVRYLFEckdpeskapKYIQFSDHiiaprksSHFHIFMGNDsqqsllnemeNWPTYYPYqlsseevveemmsh

d1lpja_ PADLSGTWTLLSSDNFEGYMLALGIDFATRKIAKLLKPQKVIEQNGDSFTIHTNSSLRNYFVKFKVGEEFDEDNRGLDNRKCKSLVIWDNDRLTCIQKGEKKNRGWTHWIEGDKLHLEMFCEGQVCKQTFQRA

d1opaa_ TKDQNGTWEMESNENFEGYMKALDIDFATRKIAVRLTQTKIIVQDGDNFKTKTNSTFRNYDLDFTVGVEFDEHTKGLDGRNVKTLVTWEGNTLVCVQKGEKENRGWKQWVEGDKLYLELTCGDQVCRQVFKKK

d1lpja_ paDLSGTWTLLSSDNFEGYMLALGIDFATRKIAKLLKPQKVIEQNGDSFTIHTNSSLRNYFVKFKVGEEFDEDnrgLDNRKCKSLVIWDNDRLTCIQKgekkNRGWTHWIEGDKLHLEMFCEGQVCKQTFQRA

d1p6pa_ --AFNGTWNVYAQENYENFLRTVGLPEDIIKVAKDVNPVIEIEQNGNEFVVTSKTPKQTHSNSFTVGKESEIT--sMDGKKIKVTVQLEGGKLICKSD----KFSHIQEVNGDEMVEKITIGSSTLTRKSKRV

d1lpja_ padlsgtwtllssdnfegymlalgidfatrkiakllkpqkvieqngdsftihtnsslrnyfvkfkVGEEFDEDnrgldnrKCKSLVIWDN----DRLTCIQKGEKKNRGWTHWIegdklHLEMFCE-----GQVCKQTFQ-------------------------------------------ra

d1r0ua_ -------------------------------gfqsnamkqetpitlhvksvieddgnqeviefrtTGFYYVKQ-------NKVYLSYYEEhdlgKVKTIVKVSEGEVLVMRSGA-----VKMNQRFvtgasTIAKYKMSFgelelktstksiqsdldeekgrisiaydmhvghlhnmtityeggt

d1lpja_ paDLSGTWTLLSSDNFEGYMLALGIDFATRKIAKLLKPQKVIEQNGDSFTIHTNSSLRNYFVKFKVGEEFDEDNrGLDNrKCKSLVIWDNDRLTCIQKgekkNRGWTHWIEGDKLHLEMFCEGQVCKQTFQRA

d1tvqa_ --AFSGTWQVYAQENYEEFLKALALPEDLIKMARDIKPIVEIQQKGDDFVVTSKTPRQTVTNSFTLGKEADITT-MDGK-KLKCTVHLANGKLVTKSE----KFSHEQEVKGNEMVETITFGGVTLIRRSKRV

d1lpja_ -----------------------------------------------------------------------------------------------------------------------------------------------------------pADLSGTWTLLSS------------------dnfegyMLALgidfatrkiakllKPQKVIEQNGDSFTIHTNSS-lrnYFVKFKVGEEFDEDNrgLDNRKCKSLVIWDN-DRLTCIQKGE--KKNRGWTHWIEG----DKLHLEMFCEGQVCKQTFQRA-----------------------------------------------

d1vpra1 ekgfeagdnklggalnakhvekygdnfkngxhkpefhedglhkpxevggkkfesgfhyllechelggknasggyggplcedpygsevqaxtekllkeadsdrtlcfnnfqdpcpqltkeqvaxckgfdygdktlklpcgplpwpaglpepgyvpktNPLHGRWITVSGgqaafikeaiksgmlgaaeankivADTD--------hhqtgGXYLRINQFGDVCTVDASVAkfarAKRTWKSGHYFYEPL--VSGGNLLGVWVLPEeYRKIGFFWEXesGRCFRIERRAFPvgpyTFXRQATEVGGKISFVFYVKVsndpesdpiplqsrdytalagrdnaptnlgkpyptlakdldypkkrd

d1lpja_ pADLSGTWTLLSSDNFEGYMLALGIDFATRKIAKL--LKPQKVIEQNGDSFTIHTNSSLRNYFVKFKVGEEFDEDNrgLDNRKCKSLVIWDN-DRLTCIQKG---EKKNRGWTHWIE-GDKLHLEMFCEGQVCKQTFQRA

d1xcaa_ -PNFSGNWKIIRSENFEELLKVLGVNVMLRKIAVAaaSKPAVEIKQEGDTFYIKTSTTVRTTEINFKVGEEFEEQT--VDGRPCKSLVKWESeNKMVCEQKLlkgEGPKTSWTMELTnDGELILTMTADDVVCTRVYVRE

d1lpja_ ---------PADLSGTWTLlSSDNFEgymlalgidfatrkiaKLLKPQKVIEQN-GDSFTIHTNS-------SLRNYFVKFKV----GEEFDEDNrGLDNrKCKSLVIWDN--DRLTCIQKG-----EKKNRGWTHWIEGDKLHLEMFCE------GQVCKQTFQRA

d2a13a1 ppvhpfvapLSYLLGTWRG-QGEGEY-----------ptipsFRYGEEIRFSHSgKPVIAYTQKTwklesgaPXHAESGYFRPrpdgSIEVVIAQ-STGL-VEVQKGTYNVdeQSIKLKSDLvgnasKVKEISREFELVDGKLSYVVRXStttnplQPHLKAILDKL

d1lpja_ pADLSGTWTLLSSDNFEGYMLALGIDFATRKIAKLLKPQKVIEQNGDSFTIHTNSsLRNYFVKFKVGEEFDEDNrgLDNRKCKSLVIWDN-DRLTCIQKgekkNRGWTHWIEGDKLHLEMFCEGQVCKQTFQRA

d2f73a1 -MSFSGKYQLQSQENFEAFMKAIGLPEELIQKGKDIKGVSEIVQNGKHFKFTITAgSKVIQNEFTVGEECELET--MTGEKVKTVVQLEGdNKLVTTFK----NIKSVTELNGDIITNTMTLGDIVFKRISKRI

d1lpja_ --------PADLSGTWTLlSSDNFEgymlalgidFATRkiaKLLKPQKVIEQN-GDSFTIHTNS-------SLRNYFVKFKV----GEEFDEDNrgLDNRKCKSLVIWDN----DRLTCIQ----KGEK-------KNRGWTHWIEGDKLHLEMFCE------GQVCKQTFQRA-

d2fr2a1 dlapalqaLSPLLGSWAG-RGAGKY--------pTIRP---FEYLEEVVFAHVgKPFLTYTQQTravadgkPLHSETGYLRVcrpgCVELVLAH--PSGITEIEVGTYSVtgdvIELELSTradgSIGLaptakevTALDRSYRIDGDELSYSLQMRavgqplQDHLAAVLHRQr

d1lpja_ ----------padlSGTWTLLSsdnfegymlalgidfatrkiakllKPQKVIEQNGD-SFTIHTNS-slRNYFVKFKVgeefdednrgldnrkckslVIWDN-----DRLTCIQKgekkNRGWTHWIE--GDKLHLEMFCE--------------------------------GQVCKQTFQRA----------------------------------------

d2gc9a1 xtktfktlddflgtHFIYTYDN------------------------GWEYEWYAKNDhTVDYRIHGgxvAGRWVTDQK-------------------ADIVXltegiYKISWTEP---tGTDVALDFXpnEKKLHGTIFFPkwveehpeitvtyqnehidlxeqsrekyatypKLVVPEFANITyxgdagqnnedviseapykexpndirngkyfdqnyhrlnk

d1lpja_ ------pADLSGTWTLlSSDNFEgymlalgidfatrkiaKLLKPQKVIEQN-gdSFTIHTNSSLRNYFVKFK----VGEEFDEDNrgldnrkcKSLVIWDN-DRLTCIQKGE---KKNRGWTHWIEG-DKLHLEMFCE------GQVCKQTFQRA

d2o62a1 erpllqiNDLLGEWRG-QAVTIY----------rdlrppDIYSTTLKIQLDdagRLXQSTSFGERTITSTATikgsIVLFDQDPE-------kQVQVLLLPdGASATSPLKVqlrQPLFLEAGWLIQsDLRQRXIRSYndkgewVSLTLVTEERV

d1obqa_ dkipdfvvpgkcasvdrnklwAEQTpnRNSY-AGVWYQFAltnnpyqlIEKC-vrneysfDGKQFviestgiaydgnlLKRNGKLYpnPFGEP--hlSIDYE----NSFAAPLVILETDYSNYACLYSCidynfgyHSDFSFIFSRSAnladqyvkkceaafkninvdttrfvktvqGSSCPYdtQKTL-

d1avgi_ ---------aegddcsiekamGDFK--PEEFfNGTWYLAH----gpgvTSPAvcqkfttsGSKGFtqiveigynkfesNVKFQCNQ--VDNKNgeqySFKCKssdnTEFEADFTFISVSYDNFALVCRSitftsqpKEDRYLVFERTK-----------------------------SDTDPD--AKEIc

d1obqa_ dkipdfvvpgkcasvdrnklwaeqtpnrnSYAGVWYQFALTNNP---------------yQLIEKCVRNEYSFDGKQFVIESTGIaydgnLLKRNGKLYPNPfgephlsidyenSFAA-------------------------------PLVIlETDYsNYACLYSCIDynfgyHSDFSFIFSRSanladqyvkkceaafkninvdttrfvktvqgsscpydtqktl

d1cbia_ ----------------------------pNFAGTWKMRSSENFDellkalgvnamlrkvaVAAASKPHVEIRQDGDQFYIKTSTT-----VRTTEINFKVGE---------gfeEETVdgrkcrslptwenenkihctqtllegdgpktYWTR-ELAN-DELILTFGAD-----DVVCTRIYVRE------------------------------------------

d1obqa_ dkipdfvvpgkcasvdrnklwaeqtpnRNSYAG-VWYQFALTNNP-------------yqliEKCVRNEYSFDG-KQFVIESTGIaydgnLLKRNGKLYPNPFgephlsidyENSF-------------------------aAPLVIlETDYsNYACLYSCIDynfgyHSDFSFIFSRSanladqyvkkceaafkninvdttrfvktvqgsscpydtqktl

d1ftpa_ ---------------------------VKEFAGiKYKLDSQTNFEeymkaigvgaierkaglALSPVIELEILDgDKFKLTSKTA-----IKNTEFTFKLGEE-------fdEETLdgrkvkstitqdgpnklvheqkgdhpTIIIR-EFSK-EQCVITIKLG-----DLVATRIYKAQ------------------------------------------

d1obqa_ dkipdfvvpgkcasvdrnklwaeqtpnrNSYAGVWYQFALTNNP-------------yqliEKCVRNEYSFDGKQFVIESTGIaydgnLLKRNGKLYPNP--------------------fgePHLSidyensfaaPLVIlETDYsNYACLYSCIDynfgyHSDFSFIFSRSanladqyvkkceaafkninvdttrfvktvqgsscpydtqktl

d1ggla_ ---------------------------pPNLTGYYRFVSQKNMEdylqalnislavrkialLLKPDKEIEHQGNHMTVRTLST-----FRNYTVQFDVGVefeedlrsvdgrkcqtivtweeeHLVCvqkgevpnrGWRH-WLEG-EMLYLELTAR-----DAVCEQVFRKV-----------------------------------------h

d1obqa_ dkipdfvvpgkcasvdrnklwaeqtpnRNSYAGVWYQFALT-------------nnpyqliekCVRNEYSFDGKQFVIESTGIAydgnlLKRNGKLYPN----------pfgephlsidyensfaaP----------LVIlETDYsNYACLYSCIDynfgyHSDFSFIFSRSanladqyvkkceaafkninvdttrfvktvqgsscpydtqktl

d1kqxa_ ---------------------------PADFNGTWEMLSNDnfedvmkaldidfatrkiavhlKQTKVIVQNGDKFETKTLSTF-----RNYEVNFVIGeefdeqtkgldnrtvktlvkwdgdklvCvqkgekenrgWKQ-WIEG-DLLHLEIHCQ-----DKVCHQVFKKK-----------------------------------------n

d1obqa_ dkipdfvvpgkcasvdrnklwaeqtpnRNSYAGVWYQFALTNNP-------------yqLIEKCVRNEYSFDGKQFVIESTGIaydgnLLKRNGKLYPNPFgephlsidyENSF-------------------------aAPLVILETDysNYACLYSCIDynfgyHSDFSFIFSRSanladqyvkkceaafkninvdttrfvktvqgsscpydtqktl

d1liba_ ---------------------------CDAFVGTWKLVSSENFDdymkevgvgfatrkvAGMAKPNMIISVNGDLVTIRSEST-----FKNTEISFKLGVE-------fdEITAddrkvksiitldggalvqvqkwdgksTTIKRKRDG--DKLVVECVMK-----GVTSTRVYERA------------------------------------------

d1obqa_ dkipdfvvpgkcasvdrnklwaeqtpnRNSYAGVWYQFALTNNP-------------yqLIEKCVRNEYSFDGKQFVIESTGIaydgnLLKRNGKLYPNpfgephlsidyENSF----------------------------aAPLVIlETDYsNYACLYSCIDynfgyHSDFSFIFSRSanladqyvkkceaafkninvdttrfvktvqgsscpydtqktl

d1lpja_ ---------------------------PADLSGTWTLLSSDNFEgymlalgidfatrkiAKLLKPQKVIEQNGDSFTIHTNSS-----LRNYFVKFKVG--------eefDEDNrgldnrkckslviwdndrltciqkgekknRGWTH-WIEG-DKLHLEMFCE-----GQVCKQTFQRA------------------------------------------

d1obqa_ dkipdfvvpgkcasvdrnklwAEQTpnRNSYAGVWYQFA-----------------------------lTNNPyqliekcVRNEYSFdgkqFVIESTGIaydgnLLKRNGKLYPNP----------fgepHLSID---yENSFAAPLVILE--tdysnYACLYS---cidynfgyHSDFSFIFSRSANLADQYVKKCEAAfkninvdttrfvktvqgsscpydtqktl

d1oeja_ gkplteveqkaangvfddanvQNRT--LSDWDGVWQSVYpllqsgkldpvfqkkadadktktfaeikdyYHKG--yatdiEMIGIED----GIVEFHRN-----NETTSCKYDYDGykiltyksgkkgvrYLFECkdpeSKAPKYIQFSDHiiaprksSHFHIFmgndsqqsllnEMENWPTYYPYQLSSEEVVEEMMSH----------------------------

d1obqa_ dkipdfvvpgkcasvdrnklwaeqtpnRNSYAGVWYQFALTNNP-------------yqLIEKCVRNEYSFDGKQFVIESTGIAydgnlLKRNGKLYPNPF-------------GEPH-------lsidyensfaaPLVIlETDYsNYACLYSCIDynfgyHSDFSFIFSRSanladqyvkkceaafkninvdttrfvktvqgsscpydtqktl

d1opaa_ ---------------------------TKDQNGTWEMESNENFEgymkaldidfatrkiAVRLTQTKIIVQDGDNFKTKTNSTF-----RNYDLDFTVGVEfdehtkgldgrnvKTLVtwegntlvcvqkgekenrGWKQ-WVEG-DKLYLELTCG-----DQVCRQVFKKK------------------------------------------

d1obqa_ dkipdfvvpgkcasvdrnklwaeqtpnrnSYAGVWYQFALTNNP-------------yqliEKCVRNEYSFDGKQFVIESTGIaydgnLLKRNGKLYP------------nPFGEPH--lsidyensfaAPLVIlETDYsNYACLYSCIDynfgyHSDFSFIFSRSanladqyvkkceaafkninvdttrfvktvqgsscpydtqktl

d1p6pa_ -----------------------------AFNGTWNVYAQENYEnflrtvglpediikvakDVNPVIEIEQNGNEFVVTSKTP-----KQTHSNSFTVgkeseitsmdgkkIKVTVQleggklicksdkFSHIQ-EVNG-DEMVEKITIG-----SSTLTRKSKRV------------------------------------------

d1obqa_ ----dkipdfvvpgkCASVDRnklWAEQtpNRNSYAGVWYQFALTNNPYQLIEKCVRNEYSFDG-KQFVIESTGIAY-DGNLLKRNGKLYPNPF-gepHLSIDYENSFAAPLVILETD-YSNYACLYSCIdynfgyhSDFSFIFSRSANLADQYVKKCEAAFKNINVDTTRFVKTVQGSscpydtqktl

d1qwda_ hlestslykkssstpPRGVTV---VNNF--DAKRYLGTWYEIARFDHRFERGLEKVTATYSLRDdGGLNVINKGYNPdRGMWQQSEGKAYFTGAptraALKVSFFGPFYGGYNVIALDrEYRHALVCGPD-------RDYLWILSRTPTISDEVKQEMLAVATREGFDVSKFIWVQQPG----------

d1obqa_ dkipdfvvpgkcasvdrnklwaeqtpnrnsyAGVWYQfALTNN-----pyqliekCVRNEYSFDGKQFVIESTGiAYDGNLLKRNGKLYPnpfgephLSIDYE------NSFAA------------------------pLVILetdYSNYACLYSCIDYNFgYHSDFSFIFSRSAnladqyvkkceaafkninvdttrfvktvqgsscpydtqktl

d1r0ua_ ----------------------gfqsnamkqETPITL-HVKSVieddgnqeviefRTTGFYYVKQNKVYLSYYE-EHDLGKVKTIVKVSE-------GEVLVMrsgavkMNQRFvtgastiakykmsfgelelktstksIQSD--lDEEKGRISIAYDMHVgHLHNMTITYEGGT-----------------------------------------

d1obqa_ dkipdfvvpgkcasvdrnklwaeqtpnrnSYAGVWYQFALTNNP-------------yqliEKCVRNEYSFDGKQFVIESTGIaydgnLLKRNGK-------------------lYPNPfgepHLSIdyensfaAPLVIlETDYsNYACLYSCIDynfgyHSDFSFIFSRSanladqyvkkceaafkninvdttrfvktvqgsscpydtqktl

d1tvqa_ -----------------------------AFSGTWQVYAQENYEeflkalalpedlikmarDIKPIVEIQQKGDDFVVTSKTP-----RQTVTNSftlgkeadittmdgkklkctVHLA-ngkLVTK----sekFSHEQ-EVKG-NEMVETITFG-----GVTLIRRSKRV------------------------------------------

d1obqa_ ----------------------------------------------------------------------------------------dkipdfvvpgkcASVDR-----------------------------------------nklwaeqTPNRnSYAGVWYQFAL------------------------tNNPYQliekcVRNEYSFDGKQFVIESTGIAydgnlLKRNGKLYPNPfgephlsidyeNSFA----------------------APLVIlETDY---SNYACLYSCIDynfgyHSDFSFIFSRS-----anladqyvkkceaafkninvdttrfvktvqgsscpydtqktl

d1vpra1 ekgfeagdnklggalnakhvekygdnfkngxhkpefhedglhkpxevggkkfesgfhyllechelggknasggyggplcedpygsevqaxtekllkeadsDRTLCfnnfqdpcpqltkeqvaxckgfdygdktlklpcgplpwpaglpepgyvPKTN-PLHGRWITVSGgqaafikeaiksgmlgaaeankivaDTDHH-qtggXYLRINQFGDVCTVDASVAK----fARAKRTWKSGH-yfyeplvsggNLLGvwvlpeeyrkigffwexesgrcFRIER-RAFPvgpYTFXRQATEVG-----GKISFVFYVKVsndpesdpiplqsrdytalagrdnaptnlgkpyptlakdldypkkrd

d1obqa_ dkipdfvvpgkcasvdrnklwaeqtpnrNSYAGVWYQFALTNNP---------------yqlIEKCVRNEYSFDGKQFVIESTGIaydgnLLKRNGKLYPnpfgephlsidyeNSFA-------------------------------aPLVIlETDYSNYACLYSCIDynfgyHSDFSFIFSRSanladqyvkkceaafkninvdttrfvktvqgsscpydtqktl

d1xcaa_ ----------------------------PNFSGNWKIIRSENFEellkvlgvnvmlrkiavaAASKPAVEIKQEGDTFYIKTSTT-----VRTTEINFKV---------geefEEQTvdgrpckslvkwesenkmvceqkllkgegpktSWTM-ELTNDGELILTMTAD-----DVVCTRVYVRE------------------------------------------

d1obqa_ dkipdfvvpgkcasvdrnklwaeqtpnrnSYAGVWYQFALTNnPYQLIekcVRNEYSFDG-KQFVIESTGiayDGNLLKRNGKLYPNpfGEPH-LSIDyensFAAPLVILETDYSNYACLYSCIDYnfgYHSDFSFIFSRSANLADQYVKKCEAAFKNINVDTTRFVKTVQGsscpydtqktl

d1xkia_ -----------------------------DVSGTWYLKAMTV-NLESV---TPMTLTTLEgGNLEAKVTM---SGRCQEVKAVLEKT--DEPGkYTAD---gGKHVAYIIRSHVKDHYIFYSEGEG---KPVRGVKLVGRDPKNNLEALEDFEKAAGARGLSTESILIPRQS-----------

d1obqa_ dkipdfvvpgkcASVDrnkLWAEQtpNRNSYAGVWYQFALTN---------nPYQLiekcVRNEYSFDGK-QFVIESTGiAYDGNLLKRNGKLYPNpfGEPH-LSIDYenSFAAPLVILETDYSNYACLYSCIDYnfgyHSDFSFIFSRSANLADQYVKKCEAAFKNINVDttRFVKT---VQGSscpydtqktl

d1yupa1 -----------iIVTQ---TMKDL--DVQKVAGTWYSLAMAAsdislldaqsAPLR---vYVEELKPTPGgDLEILLQK-WENGKCAQKKIIAEKT--EIPAvFKIDA--LNENKVLVLDTDYKKYLLFCMENSA-epeQSLACQCLVRTPEVDDEAMEKFDKALKALPMH--IRLSFnptQLEE------qcrv

d1obqa_ dkipdfvvpgkcasvdrnklwaeqTPNRNSYAGVWYQ-faltnnpyqlieKCVRNEYSFDGK-QFVIESTGIA--yDGNLLKRNGKLYPNpfGEPHLSIDYE----NSFAAPLVILEtdysnyaCLYSC----idynfgyhSDFSFI--------------------------FSRSanladqyvkkceaafkninvdttrfvktvqgsscpydtqktl

d2a13a1 ------------------ppvhpfVAPLSYLLGTWRGqgegeyptipsfrYGEEIRFSHSGKpVIAYTQKTWKlesGAPXHAESGYFRPR--PDGSIEVVIAqstgLVEVQKGTYNV----deqSIKLKsdlvgnaskvkeISREFElvdgklsyvvrxstttnplqphlkaiLDKL------------------------------------------

d1obqa_ dkipdfvvpgkcasvdrnklwaeqtpnrNSYAGVWYQFALT-------------nnpyqliekCVRNEYSFDGKQFVIESTGIAydgnlLKRNGKLYPNPfgephlsiDYEN----SFAA-----------------PLVIlETDYsNYACLYSCIDynfgyHSDFSFIFSRSanladqyvkkceaafkninvdttrfvktvqgsscpydtqktl

d2f73a1 ----------------------------MSFSGKYQLQSQEnfeafmkaiglpeeliqkgkdiKGVSEIVQNGKHFKFTITAGS-----KVIQNEFTVGE------ecELETmtgeKVKTvvqlegdnklvttfkniKSVT-ELNG-DIITNTMTLG-----DIVFKRISKRI------------------------------------------

d1obqa_ dkipdfvvpgkcasvdrnklwaeqTPNRNSYAGVWYQfALTN--npyqlieKCVRNEYSFDGK-QFVIESTGIA-YDGNL-LKRNGKLYPNpfGEPHLSIDYE----NSFAAPLV----------------------------------ilETDYsNYACLYsciDYNFGYH-sdfsfIFSRSanladqyvkkceaafkninvdttrfvktvqgsscpydtqktl

d2fr2a1 -------------------dlapaLQALSPLLGSWAG-RGAGkyptirpfeYLEEVVFAHVGKpFLTYTQQTRAvADGKPlHSETGYLRVC--RPGCVELVLAhpsgITEIEVGTysvtgdvielelstradgsiglaptakevtaldrsyRIDG-DELSYSlqmRAVGQPLqdhlaaVLHRQ-----------------------------------------r

d1obqa_ dkipdfvvpgkcasvdrnklwaEQTPNRNSyAGVWYQFAltnnpyqliekcVRNEYSFDGK-QFVIESTGiaydgnllKRNG--KLYP----npfgephLSIDyenSFAAPLVIlETDY-SNYACLYSCIDY---------------------------nfgYHSDFSFIFSRSanladqyvkkceaafkninvdttrfvktvqgsscpYDTQKT----------------l

d2gc9a1 ----------------xtktfkTLDDFLGT-HFIYTYDN-----------gWEYEWYAKNDhTVDYRIHG----gxvaGRWVtdQKADivxltegiykiSWTE---PTGTDVAL-DFXPnEKKLHGTIFFPKwveehpeitvtyqnehidlxeqsrekyatyPKLVVPEFANIT------------------yxgdagqnnedviseapYKEXPNdirngkyfdqnyhrlnk

d1obqa_ dkipdfvvpgkcasvdrnklwAEQTpnRNSYAGVWYQfALTNNP---yqliekCVRNEYSFD-gKQFVIESTGIAydgnlLKRNG----------------------------klypNPFGephlsidyensfaAPLVIlETDYSNYACLYSCIDYNFGYH-SDFSFIFSRSanladqyvkkceaafkninvdttrfvktvqgsscpydtqktl

d2o62a1 -------------------erPLLQ--INDLLGEWRG-QAVTIYrdlrppdiySTTLKIQLDdaGRLXQSTSFGE-----RTITStatikgsivlfdqdpekqvqvlllpdgasatsPLKV-------qlrqplFLEAG-WLIQSDLRQRXIRSYNDKGEWvSLTLVTEERV------------------------------------------

d1obqa_ dkipdfvvpgkCASVDrnkLWAEQtpNRNSYA--GVWYQFALTNNP--yqliEKCVRNEYSFDGKQFVIESTGIAY-DGNLLKRNGKLYPNpfgepHLSIDYEN-------------sFAAPLVILETDySNYACLYSCidynfgyhSDFSFIFSRSANLAdqYVKKCEAAFKNINVDTTRFVKTVqgsSCPYdtQKTL-------

d2ofmx1 ---------acTKNAI---AQTGF--NKDKYFngDVWYVTDYLDLEpddvpkRYCAALAAGTASGKLKEALYHYDPkTQDTFYDVSELQVE--slgKYTANFKKvdkngnvkvavtagNYYTFTVMYAD-DSSALIHTClhkgnkdlGDLYAVLNRNKDAA--AGDKVKSAVSAATLEFSKFISTK-enNCAY--DNDSlkslltk

d1obqa_ dkipdfvvpgkcasvdrnkLWAEQtpNRNSYAGVWYQFALTN--------nPYQLIekcVRNEYSFDGKQFVIESTGIaYDGNLLKRNGKLYPNPfGEPHLSIDYenSFAAPLVILETDYSNYACLYSCIDYNfGYHSDFSFIFSRSANLADQYVKKCEAAFKNINVDTTRFVKT--VQGSscpydtqktl

d2ozqa1 --------------eeassTGRNF--NVEKINGEWHTIILASdkrekiednGNFRL---FLEQIHVLEKSLVLKFHTV-RDEECSELSMVADKTE-KAGEYSVTY--DGFNTFTIPKTDYDNFLMAHLINEKD-GETFQLMGLYGREPDLSSDIKERFAQLCEEHGILRENIIDLsnANRC----------

d1obqa_ dkipdfvvpgKCASVDRnkLWAEQtpNRNSYA--GVWYQFALTNNP--yqliEKCVRNEYSFDGKQFVIESTGIAY-DGNLLKRNGKLYPNpfgepHLSIDYEN-------------sFAAPLVILETDySNYACLYSCIdynfgyHSDFSFIFSRSANLAdqYVKKCEAAFKNINVDTTRFVKTVqgsSCPYdtQKTL-------

d3np1a_ ----------KCTKNAL--AQTGF--NKDKYFngDVWYVTDYLDLEpddvpkRYCAALAAGTASGKLKEALYHYDPkTQDTFYDVSELQEE--spgKYTANFKKvekngnvkvdvtsgNYYTFTVMYAD-DSSALIHTCLhkgnkdLGDLYAVLNRNKDTN--AGDKVKGAVTAASLKFSDFISTK-dnKCEY--DNVSlkslltk

d1oeja_ gkplteveqkaangvfddanvqnrtlsdwdgvwqsvypllqsgkldpvfqkkadadktktfaeikdyyhkgyatdiemigiedgivefhrnnettsckydYDGYKILTyksgkKGVRYLFECKDPESkapKYIQFSDHiiaprkSSHFHIFMGNdsqqsllnemenWPTYYPYQL------------------------------------------sseevveemmsh

d1r0ua_ ------------------------------------------------------------------gfqsnamkqetpitlhvksvieddgnqeviefrtTGFYYVKQ-----NKVYLSYYEEHDLG--kVKTIVKVS------EGEVLVMRSG--------avkmNQRFVTGAStiakykmsfgelelktstksiqsdldeekgrisiaydmhvghlhnmtityeggt

d1oeja_ ----------------------------------------------------------------------------------------------------------------------------------gkplteveqkaangvfddanVQNRtlSDWDGVWQSVypllqsgkldPVFQKKADADKT--kTFAEIKDYYHKGYatdIEMIGIED----GIVEFH-rnnetTSCKYDY------------------DGYKiltyksgkkgvRYLFeckdpeskapkyiQFSDhiiaprksSHFHIFmgndsqqsllnemENWPTYYPYQ---------------------------------lsseevveemmsh

d1vpra1 ekgfeagdnklggalnakhvekygdnfkngxhkpefhedglhkpxevggkkfesgfhyllechelggknasggyggplcedpygsevqaxtekllkeadsdrtlcfnnfqdpcpqltkeqvaxckgfdygdktlklpcgplpwpaglpepGYVPktNPLHGRWITV-----sggqaAFIKEAIKSGMLgaaEANKIVADTDHHQ-tgGXYLRINQfgdvCTVDASvakfarAKRTWKSghyfyeplvsggnllgvwVLPE---------eyRKIGffwexesgrcfriERRA--fpvgpyTFXRQA-------tevggkISFVFYVKVSndpesdpiplqsrdytalagrdnaptnlgkpyptlakdldypkkrd

d1oeja_ gkplteveqkaangvfddanvqnrtlSDWDGVWQSVYpllqsgkldpvfqkkadadktktfaeikdyyhkgyATDIeMIGIED-----GIVEFHR-------NNETTSCKY---------------------DYDGYKILtyksgkkGVRYLFE-cKDPE--skapKYIQFSDHiiaprksSHFHIFMGNDsqqsllnemeNWPTYYPYqlsseevveemmsh

d2a13a1 ----------------ppvhpfvaplSYLLGTWRGQG-------------------------egeyptipsfRYGE-EIRFSHsgkpvIAYTQKTwklesgaPXHAESGYFrprpdgsievviaqstglvevQKGTYNVD-------EQSIKLKsdLVGNaskvkeISREFELV------dGKLSYVVRXS-tttnplqphLKAILDKL--------------

d1oeja_ gkplteveqkaangvfddanvqnrtlSDWDGVWQSVYpllqsgkldpvfqkkadadktktfaeikdyyhkgyATDIeMIGIED-----GIVEFHR-------NNETTSCKY---------------------DYDGYKILTyksgkkGVRYLFE---cKDPE------skapKYIQFSDHiiaprkSSHFHIFMGNDsqqsllnemenWPTYYPYQlsseevveemmsh

d2fr2a1 -----------------dlapalqalSPLLGSWAGRG-------------------------agkyptirpfEYLE-EVVFAHvgkpfLTYTQQTravadgkPLHSETGYLrvcrpgcvelvlahpsgiteiEVGTYSVTG-----dVIELELStradGSIGlaptakevtaLDRSYRID------GDELSYSLQMR-avgqplqdhlAAVLHRQR-------------

d1oeja_ gkplteveqkaangvfddanvQNRT-LSDWdgVWQSVypllqsgkldpvfqkkadadktktfaeikdyyhkgYATDIEMIGIED-GIVEFHR-----NNETTS-CKYDYDGYKIltyksgkkgVRYLFECKDPEskapKYIQFSDHiiaprksshFHIFMGND-----------------------------sqqsllnemeNWPTYYP----------------------yQLSSE--evveemmsh

d2gc9a1 ----------------xtktfKTLDdFLGT--HFIYT----------------------------------yDNGWEYEWYAKNdHTVDYRIhggxvAGRWVTdQKADIVXLTE---------GIYKISWTEPT-gtdVALDFXPN--------eKKLHGTIFfpkwveehpeitvtyqnehidlxeqsrekyatypklvvpEFANITYxgdagqnnedviseapykexpndIRNGKyfdqnyhrlnk

d1oeja_ gkplteveqkaangvfddanvqnrTLSDWDGVWQsvypllqsgkldpvfqkkadadktktfaeikdyyhkgyatdieMIGIED-----GIVEFHRNNETTSCKYDYDGykiltyksgkkgvrYLFECKDPESKapKYIQFSDHIiaprksshfHIFMGND-------------sqqsllnemenwptyypyqlsseevveemmsh

d2o62a1 -------------------erpllQINDLLGEWR-------------------------gqavtiyrdlrppdiystTLKIQLddagrLXQSTSFGERTITSTATIKG--------------SIVLFDQDPEK-qVQVLLLPDG---------ASATSPLkvqlrqplfleagwliqsdlrqrxirsyndkgewvsltlvteerv

d1opaa_ tkdqngtwemesNENFegymkaldidfatrkiavrltqtkiivQDGDNFKTKTN-STFRN---ydlDFTVGV-----efdehtkGLDG--RNVKTLVTW--EGNTLVCVQKG-EKENRGWKQWVEGD------KLYLELTCG-DQVCRQVFK--------------kk

d1avgi_ ------------AEGD--------------dcsiekamgdfkpEEFFNGTWYLAhGPGVTspavcqKFTTSGskgftqiveigyNKFEsnVKFQCNQVDnkNGEQYSFKCKSsDNTEFEADFTFISVsydnfaLVCRSITFTsQPKEDRYLVfertksdtdpdakeic

d1opaa_ -------------------------tkDQNGTWEMES--------------nenfegymkaldidfatrkiavrltQTKIIVQdgdnFKTKTNSTFRNYDLDFTVgvefdehtkgldgrnvktlVTWEG---------NTLVCVQK--gekenrGWKQWVE------GDKLYLELTCG-------dqvCRQVFKKK--------------

d1oeja_ gkplteveqkaangvfddanvqnrtlsDWDGVWQSVYpllqsgkldpvfqkkadadktktfaeikdyyhkgyatdiEMIGIED----GIVEFHRNNETTSCKYDY-------------------DGYKIltyksgkkgVRYLFECKdpeskapkYIQFSDHiiaprkSSHFHIFMGNDsqqsllnemeNWPTYYPYqlsseevveemmsh

d1opaa_ tkDQNGTWEMESNENFEGYMKALDIDFATRKIAVRLTQTKIIVQDGDNFKTKTNSTFRNYDLDFTVGVEFDEHTkgLDGRNVKTLVTWEGNTLVCVQKgekeNRGWKQWVEGDKLYLELTCGDQVCRQVFKKK

d1p6pa_ --AFNGTWNVYAQENYENFLRTVGLPEDIIKVAKDVNPVIEIEQNGNEFVVTSKTPKQTHSNSFTVGKESEITS--MDGKKIKVTVQLEGGKLICKSD----KFSHIQEVNGDEMVEKITIGSSTLTRKSKRV

d1opaa_ -----tkdqNGTWEMeSNENFEgymkalDIDFatrkiavrLTQTKIIVQDGDNFKTKTNST----FRNYDldftvgvefdehtkgldgrnvktlvtwegnTLVC----------------VQKG--------------EKENRGWKQWVEGDKLYLELTCG-----DQVCRQVFKKK-

d1r0ua_ gfqsnamkqETPITL-HVKSVI-----eDDGN---qevieFRTTGFYYVKQNKVYLSYYEEhdlgKVKTI---------------------------vkvSEGEvlvmrsgavkmnqrfvTGAStiakykmsfgelelKTSTKSIQSDLDEEKGRISIAYDmhvghLHNMTITYEGGt

d1opaa_ tkDQNGTWEMESNENFEGYMKALDIDFATRKIAVRLTQTKIIVQDGDNFKTKTNSTFRNYDLDFTVGVEFDEHTkgLDGRnVKTLVTWEGNTLVCVQKgekeNRGWKQWVEGDKLYLELTCGDQVCRQVFKKK

d1tvqa_ --AFSGTWQVYAQENYEEFLKALALPEDLIKMARDIKPIVEIQQKGDDFVVTSKTPRQTVTNSFTLGKEADITT-mDGKK-LKCTVHLANGKLVTKSE----KFSHEQEVKGNEMVETITFGGVTLIRRSKRV

d1opaa_ -----------------------------------------------------------------------------------------------------------------------------------------------------------tKDQNGTWEMESNE-----nfegymkaldiDFATRKI-----avrltQTKIIVQDGDNFKTKTNST-frnYDLDFTVGVEFDEHTKglDGRNVKTLVTWEGN-TLVCVQKG--EKENRGWKQWVEG----DKLYLELTCGDQVCRQVFKKK-----------------------------------------------

d1vpra1 ekgfeagdnklggalnakhvekygdnfkngxhkpefhedglhkpxevggkkfesgfhyllechelggknasggyggplcedpygsevqaxtekllkeadsdrtlcfnnfqdpcpqltkeqvaxckgfdygdktlklpcgplpwpaglpepgyvpktNPLHGRWITVSGGqaafikeaiksgmlgaAEANKIVadtdhhqtggXYLRINQFGDVCTVDASVAkfarAKRTWKSGHYFYEPLV--SGGNLLGVWVLPEEyRKIGFFWExeSGRCFRIERRAFPvgpyTFXRQATEVGGKISFVFYVKVsndpesdpiplqsrdytalagrdnaptnlgkpyptlakdldypkkrd

d1opaa_ tKDQNGTWEMESNENFEGYMKALDIDFATRKIAV--RLTQTKIIVQDGDNFKTKTNSTFRNYDLDFTVGVEFDEHTkgLDGRNVKTLVTWEG-NTLVCVQKG---EKENRGWKQWVE-GDKLYLELTCGDQVCRQVFKKK

d1xcaa_ -PNFSGNWKIIRSENFEELLKVLGVNVMLRKIAVaaASKPAVEIKQEGDTFYIKTSTTVRTTEINFKVGEEFEEQT--VDGRPCKSLVKWESeNKMVCEQKLlkgEGPKTSWTMELTnDGELILTMTADDVVCTRVYVRE

d1opaa_ ---------TKDQNGTWEMeSNENFEgymkalDIDFatrkiaVRLTQTKIIVQD-GDNFKTKTNS-------TFRNYDLDFTV----GVEFDEHTKGldGRNVKTLVTWEG--NTLVCVQKG-----EKENRGWKQWVEGDKLYLELTCG------DQVCRQVFKKK

d2a13a1 ppvhpfvapLSYLLGTWRG-QGEGEY------PTIP-----sFRYGEEIRFSHSgKPVIAYTQKTwklesgaPXHAESGYFRPrpdgSIEVVIAQST--GLVEVQKGTYNVdeQSIKLKSDLvgnasKVKEISREFELVDGKLSYVVRXStttnplQPHLKAILDKL

d1opaa_ tKDQNGTWEMESNENFEGYMKALDIDFATRKIAVRLTQTKIIVQDGDNFKTKTNStFRNYDLDFTVGVEFDEHTkgLDGRNVKTLVTWEG-NTLVCVQKgekeNRGWKQWVEGDKLYLELTCGDQVCRQVFKKK

d2f73a1 -MSFSGKYQLQSQENFEAFMKAIGLPEELIQKGKDIKGVSEIVQNGKHFKFTITAgSKVIQNEFTVGEECELET--MTGEKVKTVVQLEGdNKLVTTFK----NIKSVTELNGDIITNTMTLGDIVFKRISKRI

d1opaa_ --------TKDQNGTWEMeSNENFEgymkalDIDFatrkiaVRLTQTKIIVQD-GDNFKTKTNS-------TFRNYDLDFTV----GVEFdEHTKGldGRNVKTLVTWEG----NTLVCVQK-----------gEKENRGWKQWVEGDKLYLELTCG-----DQVC-RQVFKKK-

d2fr2a1 dlapalqaLSPLLGSWAG-RGAGKY------PTIR-----pFEYLEEVVFAHVgKPFLTYTQQTravadgkPLHSETGYLRVcrpgCVELvLAHPS--GITEIEVGTYSVtgdvIELELSTRadgsiglaptakEVTALDRSYRIDGDELSYSLQMRavgqpLQDHlAAVLHRQr

d1opaa_ ----------tKDQNGTWEMESnenfegymkaldidfatrkiavrlTQTKIIVQDGD-NFKTKTNS-tfrNYDLDFTVgvefdehtkgldgrnvktlVTWEG-----NTLVCVQKgekeNRGWKQWVE--GDKLYLELTCG--------------------------------DQVCRQVFKKK----------------------------------------

d2gc9a1 xtktfktlddfLGTHFIYTYDN------------------------GWEYEWYAKNDhTVDYRIHGgxvaGRWVTDQK-------------------ADIVXltegiYKISWTEP---tGTDVALDFXpnEKKLHGTIFFPkwveehpeitvtyqnehidlxeqsrekyatypKLVVPEFANITyxgdagqnnedviseapykexpndirngkyfdqnyhrlnk

d1opaa_ ------tKDQNGTWEMeSNENFEGymkaldidfatrkiaVRLTQTKIIVQD-gDNFKTKTNSTFRNYDLDFT----VGVEFDEHTkgldgrnvKTLVTWEG-NTLVCVQKGE---KENRGWKQWVEG-DKLYLELTCG------DQVCRQVFKKK

d2o62a1 erpllqiNDLLGEWRG-QAVTIYR----------dlrppDIYSTTLKIQLDdaGRLXQSTSFGERTITSTATikgsIVLFDQDPE-------kQVQVLLLPdGASATSPLKVqlrQPLFLEAGWLIQsDLRQRXIRSYndkgewVSLTLVTEERV

d1p6pa_ --------------AFNG-----twNVYAqenyenflrtvglpediikvakdvnpvieieqngnefvvtsktpkqthsnsftvgkeseitsmdgkkikvTVQL--EGGKLICKSD-----KFSHIQEVNGD------EMVEKITIG-SSTLTRK--------------skrv

d1avgi_ aegddcsiekamgdFKPEeffngtwYLAH------------------------------gpgvtspavcqkfttsgskgftqiveigynkfesnvkfqcNQVDnkNGEQYSFKCKssdntEFEADFTFISVsydnfaLVCRSITFTsQPKEDRYlvfertksdtdpdakeic

d1p6pa_ ---------------------------AFNGTWNVYA--------------qenyenflrtvglpediikvakdvNPVIEIEQngneFVVTSKTPKQTHSNSFTVgkeseitsmdgkkikvtVQLEG---------GKLICK------sdkFSHIQEVN------GDEMVEKITIG-------sstLTRKSKRV--------------

d1oeja_ gkplteveqkaangvfddanvqnrtlsDWDGVWQSVYpllqsgkldpvfqkkadadktktfaeikdyyhkgyatdIEMIGIED----GIVEFHRNNETTSCKYDY-----------------DGYKIltyksgkkgVRYLFEckdpeskapKYIQFSDHiiaprkSSHFHIFMGNDsqqsllnemeNWPTYYPYqlsseevveemmsh

d1p6pa_ afngtwnvyaqenyenflrtvglpediikvakdvnpvieieqngnefvvtsktpkqthsnsftvgkeseitsmdgkkikvTVQLEG----GKLICKSD----KFSHIQEVNGDEMVEKITIGsSTLTRKSKRV--------------------------------------------------------

d1r0ua_ ----------------------------------------------gfqsnamkqetpitlhvksvieddgnqeviefrtTGFYYVkqnkVYLSYYEEhdlgKVKTIVKVSEGEVLVMRSGA-VKMNQRFVTGastiakykmsfgelelktstksiqsdldeekgrisiaydmhvghlhnmtityeggt

d1p6pa_ AFNGTWNVYAQENYENFLRTVGLPEDIIKVAKDVNPVIEIEQNGNEFVVTSKTPKQTHSNSFTVGKESEITS-MDGKkIKVTVQLEGGKLICKSDKFSHIQEVNGDEMVEKITIGSSTLTRKSKRV

d1tvqa_ AFSGTWQVYAQENYEEFLKALALPEDLIKMARDIKPIVEIQQKGDDFVVTSKTPRQTVTNSFTLGKEADITTmDGKK-LKCTVHLANGKLVTKSEKFSHEQEVKGNEMVETITFGGVTLIRRSKRV

d1p6pa_ -------------------------------------------------------------------------------------------------------------------------------------------------------------AFNGTWNVYAQ------enyenflrtvglPEDIIKVAK----dvNPVIEIEQNGNEFVVTSKTP-kqtHSNSFTVGKESEITsmdgKKIKVTVQL-EGGKLICKSD------KFSHIQEVNG----DEMVEKITIGSSTLTRKSKRV-----------------------------------------------

d1vpra1 ekgfeagdnklggalnakhvekygdnfkngxhkpefhedglhkpxevggkkfesgfhyllechelggknasggyggplcedpygsevqaxtekllkeadsdrtlcfnnfqdpcpqltkeqvaxckgfdygdktlklpcgplpwpaglpepgyvpktnPLHGRWITVSGgqaafikeaiksgmlgaaEANKIVADTdhhqtgGXYLRINQFGDVCTVDASVAkfarAKRTWKSGHYFYEPlvsgGNLLGVWVLpEEYRKIGFFWexesgrCFRIERRAFPvgpyTFXRQATEVGGKISFVFYVKVsndpesdpiplqsrdytalagrdnaptnlgkpyptlakdldypkkrd

d1p6pa_ -AFNGTWNVYAQENYENFLRTVGLPEDIIKVAKD--VNPVIEIEQNGNEFVVTSKTPKQTHSNSFTVGKESEITSMDGKKIKVTVQLE-GGKLICKSD-------KFSHIQEVNG-DEMVEKITIGSSTLTRKSKRV

d1xcaa_ pNFSGNWKIIRSENFEELLKVLGVNVMLRKIAVAaaSKPAVEIKQEGDTFYIKTSTTVRTTEINFKVGEEFEEQTVDGRPCKSLVKWEsENKMVCEQKllkgegpKTSWTMELTNdGELILTMTADDVVCTRVYVRE

d1p6pa_ -----------AFNGTWNVyAQENyenFLRTvglpediikvaKDVNPVIEIEQN-gNEFVVTSKT-------PKQTHSNSFTV----GKESEITsmdGKKIKVTVQLEG--GKLICKSD---------KFSHIQEVNGDEMVEKITIG------SSTLTRKSKRV

d2a13a1 ppvhpfvaplsYLLGTWRG-QGEG---EYPT--------ipsFRYGEEIRFSHSgkPVIAYTQKTwklesgaPXHAESGYFRPrpdgSIEVVIAqstGLVEVQKGTYNVdeQSIKLKSDlvgnaskvkEISREFELVDGKLSYVVRXStttnplQPHLKAILDKL

d1p6pa_ -AFNGTWNVYAQENYENFLRTVGLPEDIIKVAKDVNPVIEIEQNGNEFVVTSKTPKQTHSNSFTVGKESEITSMDGKKIKVTVQLE-GGKLICKSDKFSHIQEVNGDEMVEKITIGSSTLTRKSKRV

d2f73a1 mSFSGKYQLQSQENFEAFMKAIGLPEELIQKGKDIKGVSEIVQNGKHFKFTITAGSKVIQNEFTVGEECELETMTGEKVKTVVQLEgDNKLVTTFKNIKSVTELNGDIITNTMTLGDIVFKRISKRI

d1p6pa_ ----------AFNGTWNVyAQENyenFLRTvglpediikvaKDVNPVIEIEQN-gNEFVVTSKT-------PKQTHSNSFTV----GKESEITsmdGKKIKVTVQLEG----GKLICKSD---------------KFSHIQEVNGDEMVEKITI------GSSTLTRKSKRV-

d2fr2a1 dlapalqalsPLLGSWAG-RGAG---KYPT--------irpFEYLEEVVFAHVgkPFLTYTQQTravadgkPLHSETGYLRVcrpgCVELVLAhpsGITEIEVGTYSVtgdvIELELSTRadgsiglaptakevtALDRSYRIDGDELSYSLQMravgqpLQDHLAAVLHRQr

d1p6pa_ ------------afNGTWNVYAqenyenflrtvglpediikvakdvNPVIEIEQNGN-EFVVTSKT-pkQTHSNSFTVgkeseitsmdgkkikvtVQLEG-----GKLICKS-DKFSHIQEVNG--DEMVEKITIG--------------------------------SSTLTRKSKRV----------------------------------------

d2gc9a1 xtktfktlddflgtHFIYTYDN------------------------GWEYEWYAKNDhTVDYRIHGgxvAGRWVTDQK-----------------ADIVXltegiYKISWTEpTGTDVALDFXPneKKLHGTIFFPkwveehpeitvtyqnehidlxeqsrekyatypKLVVPEFANITyxgdagqnnedviseapykexpndirngkyfdqnyhrlnk

d1p6pa_ --------AFNGTWNVyAQENyenFLRTvglpediikvaKDVNPVIEIEQN-gnEFVVTSKTPKQTHSNSFTV---GKESEITsmdgKKIKVTVQLEG-GKLICKS-------dKFSHIQEV-NGDEMVEKITIG------SSTLTRKSKRV

d2o62a1 erpllqinDLLGEWRG-QAVT---IYRD-------lrppDIYSTTLKIQLDdagRLXQSTSFGERTITSTATIkgsIVLFDQD----PEKQVQVLLLPdGASATSPlkvqlrqpLFLEAGWLiQSDLRQRXIRSYndkgewVSLTLVTEERV

d1qwda_ hlestslykkssstpprGVTVVNNFDAKRY-LGTWYEIArfdhrferGLEKvtatYSLRDDG-----glnvinkGYNPdrgmwQQSEGKA--YFTGaptraALKVSFF----GPFYGGYNVIALDreYRHALVCGP-------dRDYLWILSRTPTisdevkqemlavatregfdvskfiwvQQPG-----

d1avgi_ ----------aegddcsIEKAMGDFKPEEFfNGTWYLAH---gpgvtSPAV-cqkFTTSGSKgftqiveigynkFESN-----VKFQCNQvdNKNG----eQYSFKCKssdnTEFEADFTFISVS-yDNFALVCRSitftsqpkEDRYLVFERTKS-------------------------dTDPDakeic

d1qwda_ hlestslykkssstpprgvtvvnnfdaKRYLGTWYEIARFDHR--------------feRGLE-KVTATYSLRdDGGLNVINKGYnpdrgmWQQSEGKAYF----------------TGAPTR---AALK--vsffgpfyGGYNViALDReyRHALVCGPD--RDYLWILSRTptisdevkqemlavatregfdvskfiwvqqpg

d1cbia_ ---------------------------PNFAGTWKMRSSENFDellkalgvnamlrkvaVAAAsKPHVEIRQD-GDQFYIKTSTT------VRTTEINFKVgegfeeetvdgrkcrsLPTWENenkIHCTqtllegdgpkTYWTR-ELAN--DELILTFGAddVVCTRIYVRE--------------------------------

d1qwda_ hlestslykkssstpprgvtvvnnfdAKRYLG-TWYEIARFDHR-------------feRGLEKVTATYSLRDDGGLNVINKGYnpdrgmWQQSEGK------------------ayFTGAPT-rAALKvsffgpfyGGYNVIaldreYRHALVCGPD--RDYLWILSRTptisdevkqemlavatregfdvskfiwvqqpg

d1ftpa_ --------------------------VKEFAGiKYKLDSQTNFEeymkaigvgaierkaGLALSPVIELEILDGDKFKLTSKTA------IKNTEFTfklgeefdeetldgrkvkstITQDGPnkLVHE--qkgdhpTIIIRE---fsKEQCVITIKLgdLVATRIYKAQ--------------------------------

d1qwda_ hlestslykkssstpprgvtvvnnfdAKRYLGTWYEIARFDHR--------------fERGLeKVTATYSLRDdGGLNVINKGYnpdrgmWQQSEGK--------------------ayFTGAPTRAALKvsffgpfyGGYNViALDReyRHALVCGPD--RDYLWILSRTptisdevkqemlavatregfdvskfiwvqqpg

d1ggla_ --------------------------PPNLTGYYRFVSQKNMEdylqalnislavrkiALLL-KPDKEIEHQG-NHMTVRTLST------FRNYTVQfdvgvefeedlrsvdgrkcqtiVTWEEEHLVCV-qkgevpnRGWRH-WLEG--EMLYLELTArdAVCEQVFRKV-------------------------------h

d1qwda_ hlestslykkssstpprgvtvvnnfdaKRYLGTWYEIARFDHR-------------feRGLEKVTATYSLRdDGGLNVINKGYnpdrgmWQQSEGKAY--------------------FTGAPTRAALKvsffgpfyGGYNViALDReyRHALVCGPD--RDYLWILSRTptisdevkqemlavatregfdvskfiwvqqpg

d1kqxa_ --------------------------pADFNGTWEMLSNDNFEdvmkaldidfatrkiAVHLKQTKVIVQN-GDKFETKTLST------FRNYEVNFVigeefdeqtkgldnrtvktlVKWDGDKLVCV-qkgekenRGWKQ-WIEG--DLLHLEIHCqdKVCHQVFKKK-------------------------------n

d1qwda_ hlestslykkssstpprgvtvvnnfdAKRYLGTWYEIARFDHR--------------fERGLeKVTATYSLRDDgGLNVINKGYnpdrgmWQQSEGK------------------aYFTGAPTRAALKvsffgpfyGGYNVIALDreyRHALVCGPD--RDYLWILSRTptisdevkqemlavatregfdvskfiwvqqpg

d1liba_ --------------------------CDAFVGTWKLVSSENFDdymkevgvgfatrkvAGMA-KPNMIISVNGD-LVTIRSEST------FKNTEISfklgvefdeitaddrkvksIITLDGGALVQV-qkwdgksTTIKRKRDG---DKLVVECVMkgVTSTRVYERA--------------------------------

d1qwda_ hlestslykkssstpprgvtvvnnfdAKRYLGTWYEIARFDHR--------------fERGLeKVTATYSLRdDGGLNVINKGYnpdrgmWQQSEGKAYF------------------TGAPTR--AALKvsffgpfyGGYNViaLDREyrHALVCGPD--RDYLWILSRTptisdevkqemlavatregfdvskfiwvqqpg

d1lpja_ --------------------------PADLSGTWTLLSSDNFEgymlalgidfatrkiAKLL-KPQKVIEQN-GDSFTIHTNSS------LRNYFVKFKVgeefdednrgldnrkcksLVIWDNdrLTCI-qkgekknRGWTH-wIEGD--KLHLEMFCegQVCKQTFQRA--------------------------------

d1qwda_ hlestslykkssstpprgvtvvNNFDAKRYLGTWYEIA---------------------------rfdhrferglEKVTATYSLrddggLNVINKGYnpdrgmWQQSEGKAYFTGA----------ptrAALKVsffGPFY-GGYNViALDR----EYRHALVCGP---------DRDYLWILSRTPT----ISDEVKQEmlavatregfdvskfiwvqqpg

d1oeja_ -gkplteveqkaangvfddanvQNRTLSDWDGVWQSVYpllqsgkldpvfqkkadadktktfaeikdyyhkgyatDIEMIGIED-----GIVEFHRN------NETTSCKYDYDGYkiltyksgkkgvrYLFECkdpESKApKYIQF-SDHIiaprKSSHFHIFMGndsqqsllnEMENWPTYYPYQLsseeVVEEMMSH----------------------

d1qwda_ hlestslykkssstpprgvtvvnnfdAKRYLGTWYEIARFDHR-------------feRGLEKVTATYSLRdDGGLNVINKGYnpdrgmWQQSEGKAYF-------------------TGAPT-RAALKvsffgpfyGGYNViALDReyRHALVCGPD--RDYLWILSRTptisdevkqemlavatregfdvskfiwvqqpg

d1opaa_ --------------------------TKDQNGTWEMESNENFEgymkaldidfatrkiAVRLTQTKIIVQD-GDNFKTKTNST------FRNYDLDFTVgvefdehtkgldgrnvktlVTWEGnTLVCV-qkgekenRGWKQ-WVEG--DKLYLELTCgdQVCRQVFKKK--------------------------------

d1qwda_ hlestslykkssstpprgvtvvnnfdakRYLGTWYEIARF-------------dhrfergleKVTATYSLRdDGGLNVINKGYnpdrgmWQQSEGKAYF----------------TGAPT--rAALKvsffgpfyGGYNViALDReyRHALVCGPD--RDYLWILSRTptisdevkqemlavatregfdvskfiwvqqpg

d1p6pa_ ----------------------------AFNGTWNVYAQEnyenflrtvglpediikvakdvNPVIEIEQN-GNEFVVTSKTP------KQTHSNSFTVgkeseitsmdgkkikvTVQLEggkLICK-----sdkFSHIQ-EVNG--DEMVEKITIgsSTLTRKSKRV--------------------------------

d1qwda_ hlestslykkssstpprgvtvvnnfdakrYLGTWYEiARFD-----hrfergleKVTATYSLRDdGGLNVINKGYNpdRGMWQQSEGKAYFtgaptraaLKVSFF------GPFYGG------------------------YNVIAldreYRHALVCGPD-------RDYLWILSRTPtisdevkqemlavatregfdvskfiwvqqpg

d1r0ua_ ---------------------gfqsnamkQETPITL-HVKSvieddgnqeviefRTTGFYYVKQ-NKVYLSYYEEH--DLGKVKTIVKVSE--------GEVLVMrsgavkMNQRFVtgastiakykmsfgelelktstksIQSDL---dEEKGRISIAYdmhvghlHNMTITYEGGT-------------------------------

d1qwda_ hlestslykkssstpprgvtvvnnfdakRYLGTWYEIARFDHR-------------feRGLEKVTATYSLRDdGGLNVINKGYnpdrgmWQQSEGK------------------ayFTGAPTRAALKvsffgpfyGGYNViALDReyRHALVCGPD--RDYLWILSRTptisdevkqemlavatregfdvskfiwvqqpg

d1tvqa_ ----------------------------AFSGTWQVYAQENYEeflkalalpedlikmARDIKPIVEIQQKG-DDFVVTSKTP------RQTVTNSftlgkeadittmdgkklkctVHLANGKLVTK-----sekFSHEQ-EVKG--NEMVETITFggVTLIRRSKRV--------------------------------

d1qwda_ ------------------------------------------------------------------------------------------------hlestSLYKKSsstPPRG-----------------------------------vtvvnnfdaKRYLGTWYEIAR------------------------fDHRFerglekVTATYSLRdDGGLNVINKGYnpdrgmwQQSEGKAY------------------FTGAPT-rAALK-vsffgpfYGGYNViALDRE--yRHALVCGPD--RDYLWILSRT---------------ptisdevkqemlavatregfdvskfiwvqqpg

d1vpra1 ekgfeagdnklggalnakhvekygdnfkngxhkpefhedglhkpxevggkkfesgfhyllechelggknasggyggplcedpygsevqaxtekllkeadsdRTLCFN--nFQDPcpqltkeqvaxckgfdygdktlklpcgplpwpaglpepgyvpktNPLHGRWITVSGgqaafikeaiksgmlgaaeankivaDTDH-hqtggXYLRINQF-GDVCTVDASVA-----kfARAKRTWKsghyfyeplvsggnllgvWVLPEEyrKIGFfwexesgrCFRIER-RAFPVgpyTFXRQATEVggKISFVFYVKVsndpesdpiplqsrdytalagrdnaptnlgkpyptlakdldypkkrd

d1qwda_ hlestslykkssstpprgvtvvnnfdaKRYLGTWYEIARFDHR--------------feRGLE-KVTATYSLRdDGGLNVINKGYnpdrgmWQQSEGKAYF----------------TGAPTR---AALK--vsffgpfyGGYNViALDREyRHALVCGPD--RDYLWILSRTptisdevkqemlavatregfdvskfiwvqqpg

d1xcaa_ ---------------------------PNFSGNWKIIRSENFEellkvlgvnvmlrkiaVAAAsKPAVEIKQE-GDTFYIKTSTT------VRTTEINFKVgeefeeqtvdgrpcksLVKWESenkMVCEqkllkgegpkTSWTM-ELTND-GELILTMTAddVVCTRVYVRE--------------------------------

d1qwda_ hlestslykkssstpprgvtvvnnfdakRYLGTWYEIARFDhRFERglekVTATYSLRDDGGLNVINKGynpdRGMWQQSEGKAYFTGAptRAALKVSffgPFYGGYNVIALDREyRHALVCGP----dRDYLWILSRTP-TISDeVKQEMLAVATREGFDVSKFIWVQQPg

d1xkia_ ----------------------------DVSGTWYLKAMTV-NLES---vTPMTLTTLEGGNLEAKVTM----SGRCQEVKAVLEKTDE--PGKYTAD---GGKHVAYIIRSHVK-DHYIFYSEgegkpVRGVKLVGRDPkNNLE-ALEDFEKAAGARGLSTESILIPRQS-

d1qwda_ hlestslykkssstpPRGVTVVNNFDAKRYLGTWYEIARFD---------hRFERglekVTATYSLRDDGGLNVINKGYNpdRGMWQQSEGKAYFTGAptRAALKVSFFgpFYGGYNVIALDrEYRHALVCGP------drDYLWILSRTPTISDEVKQEMLAVATreGFDVSKFIWV---QQPG----

d1yupa1 ---------------IIVTQTMKDLDVQKVAGTWYSLAMAAsdislldaqsAPLR---vYVEELKPTPGGDLEILLQKWE--NGKCAQKKIIAEKTEI--PAVFKIDAL--NENKVLVLDTD-YKKYLLFCMEnsaepeqsLACQCLVRTPEVDDEAMEKFDKALK--ALPMHIRLSFnptQLEEqcrv

d1qwda_ hlestslykkssstpprgvtvvnnfDAKRYLGTWYEiARFDHR--ferglEKVTATYSLRDDGGLNVINKGYNPDRGMW-QQSEGKAYF--------------------TGAPT----rAALK----vsffgpfyGGYNViALDReyRHALVCGP--------DRDYLWILSRTptisdevkqemlavatregfdvskfiwvqqpg

d2a13a1 -----------------ppvhpfvaPLSYLLGTWRG-QGEGEYptipsfrYGEEIRFSHSGKPVIAYTQKTWKLESGAPxHAESGYFRPrpdgsievviaqstglvevqKGTYNvdeqsIKLKsdlvgnaskvkeISREF-ELVD--GKLSYVVRxstttnplQPHLKAILDKL--------------------------------

d1qwda_ hlestslykkssstpprgvtvvnnfdaKRYLGTWYEIARFDHR-------------feRGLEKVTATYSLRdDGGLNVINKGYnpdrgmWQQSEGKAY-----------------fTGAPT--RAALKvsffgpfyGGYNVIALdreYRHALVCGPD--RDYLWILSRTptisdevkqemlavatregfdvskfiwvqqpg

d2f73a1 ---------------------------MSFSGKYQLQSQENFEafmkaiglpeeliqkGKDIKGVSEIVQN-GKHFKFTITAG------SKVIQNEFTvgeeceletmtgekvktvVQLEGdnKLVTT-----fknIKSVTELN---GDIITNTMTLgdIVFKRISKRI--------------------------------

d1qwda_ hlestslykkssstpprgvtvvnnFDAKRYLGT-wyeiarfdhrferglekVTATYSLRDDGGLNVINKGYNPDRGMW-QQSEGKAYFTGaptRAALKVSFFG----PFYGGYNViALDReyRHALVCGP-----------------drdYLWILSRTPtisdevkqemlavatregfdvSKFIWVQQ--pg

d2fr2a1 ------------------dlapalQALSPLLGSwagrgagkyptirpfeylEEVVFAHVGKPFLTYTQQTRAVADGKPlHSETGYLRVCR---PGCVELVLAHpsgiTEIEVGTY-SVTG--DVIELELStradgsiglaptakevtaldRSYRIDGDE-------lsyslqmravgqplQDHLAAVLhrqr

d1qwda_ hlestslykkssstpprgvtvvnnfDAKRY-LGTWYEiARFDhrferglekVTATYSLrddgGLNVINKGynpdrgmWQQSE-GKAYFtgAPTR-AALKVsFFGP-FYGGYNVIALDreyrhALVCGP--------------------------------------dRDYLWILSRtptisdevkqemlavatregfdvskfiwvQQPG----------------

d2gc9a1 -------------------xtktfkTLDDFlGTHFIY-TYDN------gweYEWYAKN----DHTVDYRI-hggxvaGRWVTdQKADIvxLTEGiYKISW-TEPTgTDVALDFXPNE-----KKLHGTiffpkwveehpeitvtyqnehidlxeqsrekyatypklvVPEFANITY----------xgdagqnnedviseapykeXPNDirngkyfdqnyhrlnk

d1qwda_ HLEStslykkssstpprgvtvvnnfDAKRYLGTWYE--iarfdhrfergleKVTATYSLRDDGGLNVINKGYnpdrgmWQQSEGKAYFTgaptRAALKVSfFGPFYGGYNVIALdreyrhALVCGP---------drDYLWIlsrtptisdevkqemlavatregfdvSKFIWVqqpg

d2o62a1 ERPL--------------------lQINDLLGEWRGqavtiyrdlrppdiySTTLKIQLDDAGRLXQSTSFG------ERTITSTATIK----GSIVLFDqDPEKQVQVLLLPD------GASATSplkvqlrqplfLEAGW-----liqsdlrqrxirsyndkgewvSLTLVTeerv

d1qwda_ hlestslykkssstPPRGVTVVNNFDAKRYL--GTWYEIARFD--HRFERGLEKVTATYSLRDDGgLNVINKGYNPDRGMWQQSEGKAYFTGaptRAALKVSFFG-------------PFYGGYNVIALDreYRHALVCGP-------drDYLWILSRTPTISdeVKQEMLAVATREGFDVSKFIWVQQPG--------------

d2ofmx1 -------------aCTKNAIAQTGFNKDKYFngDVWYVTDYLDlePDDVPKRYCAALAAGTASGK-LKEALYHYDPKTQDTFYDVSELQVES---LGKYTANFKKvdkngnvkvavtaGNYYTFTVMYAD--DSSALIHTClhkgnkdlgDLYAVLNRNKDAA--AGDKVKSAVSAATLEFSKFISTKENNcaydndslkslltk

d1qwda_ HLEStslykkssstpprgvtVVNN-FDAKRYLGTWYEIARFD--------hRFERglekVTATYSLRdDGGLNVINKGYNPdrGMWQQSEGKAYFTGAptRAALKVSFFgpFYGGYNVIALDrEYRHALVCGP------dRDYLWILSRTPTISDEVKQEMLAVATREGFDVSKFIWV--QQPG

d2ozqa1 EEAS----------------STGRnFNVEKINGEWHTIILASdkrekiednGNFR---lFLEQIHVL-EKSLVLKFHTVRD--EECSELSMVADKTEK--AGEYSVTYD--GFNTFTIPKTD-YDNFLMAHLInekdgetFQLMGLYGREPDLSSDIKERFAQLCEEHGILRENIIDLsnANRC

d1qwda_ hlestslykksSSTPprGVTVVNNFDAKRYL--GTWYEIARFDHR--ferGLEKVTATYSLRDDGgLNVINKGYNPDRGMWQQSEGKAYFTGaptRAALKVSFFG-------------PFYGGYNVIALDreYRHALVCGP-------drDYLWILSRTPTISdeVKQEMLAVATREGFDVSKFIWVQQ--------------pg

d3np1a_ -----------KCTK--NALAQTGFNKDKYFngDVWYVTDYLDLEpddvpKRYCAALAAGTASGK-LKEALYHYDPKTQDTFYDVSELQEES---PGKYTANFKKvekngnvkvdvtsGNYYTFTVMYAD--DSSALIHTClhkgnkdlgDLYAVLNRNKDTN--AGDKVKGAVTAASLKFSDFISTKDnkceydnvslkslltk

d1r0ua_ ------------------------------------------------------------------gfQSNA-mkQETP------------------------itlhvksviEDDG-----------------------nqeviefrttgfyyvkqnkvYLSYYE---EHDL----------------------------gkVKTIVKVSEGEVLVMRSGAVK--MNQRFVTGASTIAKYKmsfGELELKTSTkSIQSDldeekgRISIaYDMHVG----HLHNMTITYEGGT---------------------------------------------------------------------

d1vpra1 ekgfeagdnklggalnakhvekygdnfkngxhkpefhedglhkpxevggkkfesgfhyllechelggkNASGgygGPLCedpygsevqaxtekllkeadsdrtlcfnnfqdpCPQLtkeqvaxckgfdygdktlklpcgplpwpaglpepgyvpktnplHGRWITvsgGQAAfikeaiksgmlgaaeankivadtdhhqtggXYLRINQFGDVCTVDASVAKFarAKRTWKSGHYFYEPLV---SGGNLLGVW-VLPEE------YRKI-GFFWEXesgrCFRIERRAFPVGPytfxrqatevggkisfvfyvkvsndpesdpiplqsrdytalagrdnaptnlgkpyptlakdldypkkrd

d1r0ua_ ---gfqsNAMKQ-ETPITLHVKSVIEDdgnQEVIEFRTTGFYYV-kQNKVYLSYYEE---hdlGKVKTIVKVSE-----GEVLVMRSgaVKMNQRFVT-----------gaSTIAKYkmsfgelELKTSTkSIQSDLDEEKGRISIAYdMHVGH--LHNMTITYEGGt

d2a13a1 ppvhpfvAPLSYlLGTWRGQGEGEYPT---IPSFRYGEEIRFSHsgKPVIAYTQKTWklesgaPXHAESGYFRPrpdgsIEVVIAQS--TGLVEVQKGtynvdeqsiklksDLVGNA-------SKVKEI-SREFELVDGKLSYVVRX-STTTNplQPHLKAILDKL-

d1r0ua_ ---gfqsnamkQETPITLHVKSVIEDdgnQEVIEFRTtGFYYVKQ---NKVYLSYYEE-hdlGKVKTIVKVS-------EGEVLVMRsgavKMNQRFV---------------TGAS--TIAKYKmsfgELELKTSTkSIQSDLDEEKGRISIAYDMHV-ghLHNMTITYEGGT

d2fr2a1 dlapalqalspLLGSWAGRGAGKYPT---IRPFEYLE-EVVFAHVgkpFLTYTQQTRAvadgKPLHSETGYLrvcrpgcVELVLAHP----SGITEIEvgtysvtgdvielelSTRAdgSIGLAP----TAKEVTAL-DRSYRIDGDELSYSLQMRAVGqplQDHLAAVLHRQR

d1r0ua_ gfqsnAMKQ---------------------etPITLHVKSVIeddgnqeVIEFR-TTGFYYVKQN-KVYLSYYEEHdlgKVKTIVKVS--EGEVLVMRSGA---------------------------------VKMNQRFVtgastiakykmsfgelelktstkSIQSDldeekgrisiaydmhvGHLH--------nmtityeggt

d2gc9a1 xtktfKTLDdflgthfiytydngweyewyaknDHTVDYRIHG---gxvaGRWVTdQKADIVXLTEgIYKISWTEPT---GTDVALDFXpnEKKLHGTIFFPkwveehpeitvtyqnehidlxeqsrekyatypkLVVPEFAN----------------------iTYXGD--agqnnedviseapyKEXPndirngkyfdqnyhrlnk

d1r0ua_ -gfqsnamkqETPITLHVKSVIEDdgNQEVIEFRTTGFyYVKQ---nkvYLSYYEehdlgkVKTI---------------------------vkvSEGEvlvmrsgavkmnqrfvtGASTIAkykmsfgeleLKTSTkSIQSDL-DEEKGRISIAYDMHV-gHLHNMTITYEGGt

d2o62a1 erpllqindlLGEWRGQAVTIYRD--LRPPDIYSTTLK-IQLDdagrlxQSTSFG------ERTItstatikgsivlfdqdpekqvqvlllpdgaSATS-----------------PLKVQL----------RQPLF-LEAGWLiQSDLRQRXIRSYNDKgeWVSLTLVTEERV-

d1tvqa_ afsgtwqVYAQenyeeflkalalpedlikmardikpiveiQQKGddfvvTSKTPRQTV-TNSFTLG-------keadittMDGKK--LKCTVHLAN------GKLVTKS-EKFSHEQEVKGN----------EMVETITfggVTLIRRSKR----------------v

d1avgi_ -------AEGD--------------dcsiekamgdfkpeeFFNG--twyLAHGPGVTSpAVCQKFTtsgskgftqiveigYNKFEsnVKFQCNQVDnkngeqYSFKCKSsDNTEFEADFTFIsvsydnfalvCRSITFT---SQPKEDRYLvfertksdtdpdakeic

d1tvqa_ ---------------------------AFSGTWQVYA--------------qenyeeflkalalpedlikmardIKPIVEIQQkgddFVVTSKTPRQTVTNSFTLgkeadittmdgkklkctVHLAN---------GKLVTK------sekfSHEQEVK------GNEMVETIT-------fggvTLIRRSKRV--------------

d1oeja_ gkplteveqkaangvfddanvqnrtlsDWDGVWQSVYpllqsgkldpvfqkkadadktktfaeikdyyhkgyatDIEMIGIED----GIVEFHRNNETTSCKYDY-----------------DGYKIltyksgkkgVRYLFEckdpeskapkYIQFSDHiiaprkSSHFHIFMGndsqqsllnemENWPTYYPYqlsseevveemmsh

d1tvqa_ -------------------------------------------afSGTWQVYAQenyeeflkalalpedlikmardiKPIVEIQQKGDDFVVTSKTprqTVTNSFTLGKEADITTMD---GKKLKCTVHLANG---------KLVTKSE-KFSHEQEVKGNemvetitfggvtlirrskrv

d1r0ua_ gfqsnamkqetpitlhvksvieddgnqeviefrttgfyyvkqnkvYLSYYEEHD---------------------lgKVKTIVKVSEGEVLVMRSG-avKMNQRFVTGASTIAKYKMsfgELELKTSTKSIQSdldeekgriSIAYDMHvGHLHNMTITYE-----------------ggt

d1tvqa_ -------------------------------------------------------------------------------------------------------------------------------------------------------------AFSGTWQVYAQ-----enyeeflkalalPEDLIKMAR-----diKPIVEIQQKGDDFVVTSKTP-rqtVTNSFTLGKEADITtmdGKKLKCTVHLANG-KLVTKS------EKFSHEQEVKG----NEMVETITFGGVTLIRRSKRV-----------------------------------------------

d1vpra1 ekgfeagdnklggalnakhvekygdnfkngxhkpefhedglhkpxevggkkfesgfhyllechelggknasggyggplcedpygsevqaxtekllkeadsdrtlcfnnfqdpcpqltkeqvaxckgfdygdktlklpcgplpwpaglpepgyvpktnPLHGRWITVSGgqaafikeaiksgmlgaAEANKIVADtdhhqtgGXYLRINQFGDVCTVDASVAkfarAKRTWKSGHYFYEPlvsGGNLLGVWVLPEEyRKIGFFwexesgRCFRIERRAFPvgpyTFXRQATEVGGKISFVFYVKVsndpesdpiplqsrdytalagrdnaptnlgkpyptlakdldypkkrd

d1tvqa_ -AFSGTWQVYAQENYEEFLKALALPEDLIKMARD--IKPIVEIQQKGDDFVVTSKTPRQTVTNSFTLGKEADITTmDGKKLKCTVHLAN-GKLVTKSE-------KFSHEQEVK-GNEMVETITFGGVTLIRRSKRV

d1xcaa_ pNFSGNWKIIRSENFEELLKVLGVNVMLRKIAVAaaSKPAVEIKQEGDTFYIKTSTTVRTTEINFKVGEEFEEQTvDGRPCKSLVKWESeNKMVCEQKllkgegpKTSWTMELTnDGELILTMTADDVVCTRVYVRE

d1tvqa_ -----------AFSGTWQVyAQENYeeflkALALpedlikmaRDIKPIVEIQQK-GDDFVVTSKT-------PRQTVTNSFTL----GKEADITTmdgKKLKCTVHLAN--GKLVTKS---------EKFSHEQEVKGNEMVETITF------GGVTLIRRSKRV

d2a13a1 ppvhpfvaplsYLLGTWRG-QGEGE-----YPTI------psFRYGEEIRFSHSgKPVIAYTQKTwklesgaPXHAESGYFRPrpdgSIEVVIAQstgLVEVQKGTYNVdeQSIKLKSdlvgnaskvKEISREFELVDGKLSYVVRXstttnpLQPHLKAILDKL

d1tvqa_ -AFSGTWQVYAQENYEEFLKALALPEDLIKMARDIKPIVEIQQKGDDFVVTSKtpRQTVTNSFTLGKEADITTMDGKKLKCTVHLAN-GKLVTKSEKFSHEQEVKGNEMVETITFGGVTLIRRSKRV

d2f73a1 mSFSGKYQLQSQENFEAFMKAIGLPEELIQKGKDIKGVSEIVQNGKHFKFTITagSKVIQNEFTVGEECELETMTGEKVKTVVQLEGdNKLVTTFKNIKSVTELNGDIITNTMTLGDIVFKRISKRI

d1tvqa_ ----------AFSGTWQVyAQENYeeflkalALPEdlikmaRDIKPIVEIQQK-GDDFVVTSKT-------PRQTVTNSFTL----GKEADITTMDGKKLKCTVHLAN----GKLVTKSE---------------KFSHEQEVKGNEMVETITF------GGVTLIRRSKRV-

d2fr2a1 dlapalqalsPLLGSWAG-RGAGK------yPTIR-----pFEYLEEVVFAHVgKPFLTYTQQTravadgkPLHSETGYLRVcrpgCVELVLAHPSGITEIEVGTYSVtgdvIELELSTRadgsiglaptakevtALDRSYRIDGDELSYSLQMravgqpLQDHLAAVLHRQr

d1tvqa_ ------------afSGTWQVYAqenyeeflkalalpedlikmardiKPIVEIQQKGD-DFVVTSKT-prQTVTNSFtlgkeadittmdgkklkcTVHLAN-----GKLVTKSE-KFSHEQEVKG--NEMVETITFG--------------------------------GVTLIRRSKRV----------------------------------------

d2gc9a1 xtktfktlddflgtHFIYTYDN------------------------GWEYEWYAKNDhTVDYRIHGgxvAGRWVTD-----------------qKADIVXltegiYKISWTEPtGTDVALDFXPneKKLHGTIFFPkwveehpeitvtyqnehidlxeqsrekyatypKLVVPEFANITyxgdagqnnedviseapykexpndirngkyfdqnyhrlnk

d1tvqa_ --------AFSGTWQVyAQENYEEflkalalpedlikmaRDIKPIVEIQQK-GDDFVVTSKTPRQTVTNSFTL---GKEADITtmdgKKLKCTVHLAN-GKLVTKS-------eKFSHEQEV-KGNEMVETITFG------GVTLIRRSKRV

d2o62a1 erpllqinDLLGEWRG-QAVTIYR----------dlrppDIYSTTLKIQLDdAGRLXQSTSFGERTITSTATIkgsIVLFDQD----PEKQVQVLLLPdGASATSPlkvqlrqpLFLEAGWLiQSDLRQRXIRSYndkgewVSLTLVTEERV

d1vpra1 ekgfeagdnklggalnakhvekygdnfkngxhkpefhedglhkpxevggkkfesgfhyllechelggknasggyggplcedpygsevqaxtekllkeadsdrtlcfnnfqdpcpqltkeqvaxckgfdygdktlklpcgplpwpaglpepgyVPKT-NPLHGRWITvSGGQaafikeaiksgmlgaaeankivadtdhhqtggXYLRIN-qfgdVCTVDAS---------VAKFaraKRTWKS----GHYFYEPLVSGGNLLGVWVLPE-EYRKIGFF-WEXES--GRCFRIERRAFPvgpyTFXRQATEV-----GGKI-SFVFYVKVsndpesdpiplqsrdytalagrdnaptnlgkpyptlakdldypkkrd

d2a13a1 ---------------------------------------------------------------------------------------------------------------------------------------------------ppvhpFVAPlSYLLGTWRG-QGEG---------------------eyptipsfrygEEIRFShsgkpVIAYTQKtwklesgapXHAE---SGYFRPrpdgSIEVVIAQSTGLVEVQKGTYNVdEQSIKLKSdLVGNAskVKEISREFELVD----GKLSYVVRXstttnPLQPhLKAILDKL-----------------------------------------------

d1vpra1 ekgfeagdnklggalnakhvekygdnfkngxhkpefhedglhkpxevggkkfesgfhyllechelggknasggyggplcedpygsevqaxtekllkeadsdrtlcfnnfqdpcpqltkeqvaxckgfdygdktlklpcgplpwpaglpepgyVPKT-NPLHGRWITvSGGQaafikeaiksgmlgaaeaNKIVadtdhhqtgGXYLRINQ-fgDVCTVDAS--------VAKFaraKRTWKS----GHYFYEPLVSGGNLLGVWVLPE---eYRKIGFF----WEXE-----SGRCFRIERRAFPvgpyTFXRQATEV-----GGKI-SFVFYVKVSndpesdpiplqsrdytalagrdnaptnlgkpyptlakdldypkkrd

d2fr2a1 ----------------------------------------------------------------------------------------------------------------------------------------------------dlapALQAlSPLLGSWAG-RGAG---------------kypTIRP------feyLEEVVFAHvgkPFLTYTQQtravadgkPLHS--eTGYLRVcrpgCVELVLAHPSGITEIEVGTYSVtgdvIELELSTradgSIGLaptakEVTALDRSYRIDG----DELSYSLQMravgqPLQDhLAAVLHRQR----------------------------------------------

d1vpra1 ekgfeagdnklggalnakhvekygdnfkngxhkpefhedglhkpxevggkkfesgfhyllechelggknasggyggplcedpygsevqaxtekllkeadsdrtlcfnnfqdpcpqltkeqvaxckgfdygdktlklpcgplpwpaglpepgyvpktNPLHGRWITVSGgqaafikeaiksgmlgaaeankivadtdhhqtgGXYLRINQ-fgdVCTVDASvakfarAKRTWK----SGHYFYEPLvsggnlLGVWVLPEEYRKIGFF-WEXESGRCFRIERRAFPvgpYTFXRQATEV------GGKISFVFYVKVsndpesdpiplqsrdytalagrdnaptnlgkpyptlakdldypkkrd

d2o62a1 -----------------------------------------------------------------------------------------------------------------------------------------------------erpllqiNDLLGEWRGQAV---------------------tiyrdlrppdiySTTLKIQLddagRLXQSTS-fgertITSTATikgsIVLFDQDPE-----kQVQVLLLPDGASATSPlKVQLRQPLFLEAGWLIQ---SDLRQRXIRSyndkgeWVSLTLVTEERV-----------------------------------------------

d1xcaa_ pnfsgnwkiirSENFeellkvlgvnvmlrkiavaaaskpaveiKQEG-dtfYIKTS-TTVRT---teinFKVG-------eefeeqtVDGR--PCKSLVKW-ESENKMVCEQKLLkgEGPKTSWTMELTND-----GELILTMTAD-DVVCTRVYV--------------re

d1avgi_ -----------AEGD-----------------dcsiekamgdfKPEEffngTWYLAhGPGVTspavcqkFTTSgskgftqiveigynKFESnvKFQCNQVDnKNGEQYSFKCKSS--DNTEFEADFTFISVsydnfALVCRSITFTsQPKEDRYLVfertksdtdpdakeic

d1xcaa_ --------------------------pNFSGNWKIIR------------senfeellkvlgvnvmlrkiavaaaSKPAVEIKQegdtFYIKTSTTVRTTEINFKVgeefeeqtvdgrpckslVKWES--------eNKMVCEqkllkgegpkTSWTMELTN-----dGELILTMTAD-------dvVCTRVYVRE--------------

d1oeja_ gkplteveqkaangvfddanvqnrtlsDWDGVWQSVYpllqsgkldpvfqkkadadktktfaeikdyyhkgyatDIEMIGIED----GIVEFHRNNETTSCKYDY-----------------DGYKIltyksgkkgVRYLFE-ckdpeskapKYIQFSDHIiaprksSHFHIFMGNDsqqsllnemENWPTYYPYqlsseevveemmsh

d1xcaa_ ------------------------------------------pNFSGNWKIirsenfeellkvlgvnvmlrkiaVAAAskPAVEIKQEGDTFYIKTSTTvRTTEINFKVGEEFEEQ---tvDGRPCKSLV-KWESE-------NKMVCEQKLLkgegpkTSWTMELTNDgeliltmtaddvvctrvyvre

d1r0ua_ gfqsnamkqetpitlhvksvieddgnqeviefrttgfyyvkqnKVYLSYYE---------------------ehDLGK--VKTIVKVSEGEVLVMRSGA-VKMNQRFVTGASTIAKykmsfGELELKTSTkSIQSDldeekgrISIAYDMHVG------HLHNMTITYE------------------ggt

d1xcaa_ ------------------------------------------------------------------------------------------------------------------------------------------------------------PNFSGNWKIIRS-----enfeellkvlgvNVMLRK---iavaaasKPAVEIKQEGDTFYIKTSTT-vrtTEINFKVGEEFEEQTVDGRPCKSLVKWESENKMVCEQKLLkGEGPKTSWTMELTND---GELILTMTADDVVCTRVYVRE-----------------------------------------------

d1vpra1 ekgfeagdnklggalnakhvekygdnfkngxhkpefhedglhkpxevggkkfesgfhyllechelggknasggyggplcedpygsevqaxtekllkeadsdrtlcfnnfqdpcpqltkeqvaxckgfdygdktlklpcgplpwpaglpepgyvpktNPLHGRWITVSGgqaafikeaiksgmlgaAEANKIvadtdhhqtgGXYLRINQFGDVCTVDASVAkfarAKRTWKSGHYFYEPLVSGGNLLGVWVLPEEYRKIGFFWEX-ESGRCFRIERRAFPVgpyTFXRQATEVGGKISFVFYVKVsndpesdpiplqsrdytalagrdnaptnlgkpyptlakdldypkkrd

d1xcaa_ ----------PNFSGNWKIiRSENfeeLLKVlgvnvmlrkiavaaaSKPAVEIKQE-GDTFYIKTST-------TVRTTEINFKV----GEEFEEQtvDGRPCKSLVKWES-ENKMVCEQ-KLLKGE-GPKTSWTMELTnDGELILTMTA------DDVVCTRVYVRE

d2a13a1 ppvhpfvaplSYLLGTWRG-QGEG---EYPT----------ipsfrYGEEIRFSHSgKPVIAYTQKTwklesgaPXHAESGYFRPrpdgSIEVVIAqsTGLVEVQKGTYNVdEQSIKLKSdLVGNASkVKEISREFELV-DGKLSYVVRXstttnpLQPHLKAILDKL

d1xcaa_ PNFSGNWKIIRSENFEELLKVLGVNVMLRKIAVAaaSKPAVEIKQEGDTFYIKTSttvRTTEINFKVGEEFEEQTVDGRPCKSLVKWESENKMVCEQKllkgegpKTSWTMELTNdGELILTMTADDVVCTRVYVRE

d2f73a1 MSFSGKYQLQSQENFEAFMKAIGLPEELIQKGKD--IKGVSEIVQNGKHFKFTITagsKVIQNEFTVGEECELETMTGEKVKTVVQLEGDNKLVTTFK-------NIKSVTELNG-DIITNTMTLGDIVFKRISKRI

d1xcaa_ ---------PNFSGNWKIiRSENfeeLLKVlgvnvmlrkiavaaaSKPAVEIKQE-GDTFYIKTST-------TVRTTEINFKV----GEEFEEQtvDGRPCKSLVKWESE---NKMVCEQ----KLLK----gEGPKTSWTMELTNdGELILTMTA------DDVVCTRVYVRE-

d2fr2a1 dlapalqalSPLLGSWAG-RGAG---KYPT----------irpfeYLEEVVFAHVgKPFLTYTQQTravadgkPLHSETGYLRVcrpgCVELVLAhpSGITEIEVGTYSVTgdvIELELSTradgSIGLaptakEVTALDRSYRIDG-DELSYSLQMravgqpLQDHLAAVLHRQr

d1xcaa_ -----------pNFSGNWKIIRsenfeellkvlgvnvmlrkiavaaasKPAVEIKQEGD-TFYIKTST-tvRTTEINFKVgeefeeqtvdgrpckslVKWESE----NKMVCEQKllkgegpKTSWTMELT-NDGELILTMTAD--------------------------------DVVCTRVYVRE----------------------------------------

d2gc9a1 xtktfktlddflGTHFIYTYDN--------------------------GWEYEWYAKNDhTVDYRIHGgxvAGRWVTDQK-----------------ADIVXLtegiYKISWTEP------tGTDVALDFXpNEKKLHGTIFFPkwveehpeitvtyqnehidlxeqsrekyatypKLVVPEFANITyxgdagqnnedviseapykexpndirngkyfdqnyhrlnk

d1xcaa_ -------PNFSGNWKIiRSENFEEllkvlgvnvmlrkiavaaaSKPAVEIKQE-gdTFYIKTSTTVRTTEINFK----VGEEFEEQtvdgrpcKSLVKWESENKMVCEQKLLKGEGPKTSWTMELTNDGELILTMTAD------DVVCTRVYVRE

d2o62a1 erpllqiNDLLGEWRG-QAVTIYR------------dlrppdiYSTTLKIQLDdagRLXQSTSFGERTITSTATikgsIVLFDQDP-----ekQVQVLLLPDGASATSPLKVQLRQPLFLEAGWLIQSDLRQRXIRSYndkgewVSLTLVTEERV

d1xkia_ ------------------DVSG-TWYLKAmtvnlesvtpmtlTTLEG----gnleakvtmsgrCQEVKAVLEK-TDEPgKYTAD-------ggKHVAYIIRSHVKDHYIFYSEGE---GKPVRGVKLVGRDPKnnlealedfekaagarglstesiliPRQS------

d1avgi_ aegddcsiekamgdfkpeEFFNgTWYLAHgpgvtspavcqkfTTSGSkgftqiveigynkfesNVKFQCNQVDnKNGE-QYSFKckssdntefEADFTFISVSYDNFALVCRSITftsQPKEDRYLVFERTKS-------------------------DTDPdakeic

d1xkia_ -DVSGTWYLKAMTV-------------------NLESVTPMTLTTLEgGNLEAKVTMsgrCQEVKAVLEK-----------------TDEP----GKYT-----adggKHVAYIIRShvKDHYIFYSEGEgkPVRGVKLVGRDpknnlealedfekaagarglstesiliprqs

d1cbia_ pNFAGTWKMRSSENfdellkalgvnamlrkvavAAASKPHVEIRQDG-DQFYIKTST--tVRTTEINFKVgegfeeetvdgrkcrslPTWEnenkIHCTqtllegdgpKTYWTRELA--NDELILTFGAD--DVVCTRIYVRE-------------------------------

d1xkia_ --DVSG-TWYLKAMTV-----------------NLESVTPMTLTTLEGGNLEAKVTMsgrCQEVKAV------------------lEKTDEP---GKYTA-dggKHVAYIIRShvKDHYIFYSEGEgkPVRGVKLVGRDpknnlealedfekaagarglstesiliprqs

d1ftpa_ vkEFAGiKYKLDSQTNfeeymkaigvgaierkaGLALSPVIELEILDGDKFKLTSKT--aIKNTEFTfklgeefdeetldgrkvksTITQDGpnkLVHEQkgdhPTIIIREFS--KEQCVITIKLG--DLVATRIYKAQ-------------------------------

d1xkia_ --DVSGTWYLKAMTV-----------------NLESVTPMTLTTLEGGnLEAKVTMsgrCQEVKAV--------------------lEKTDEP--GKYTA--dggKHVAYIIRShvKDHYIFYSEGEgkPVRGVKLVGRDpknnlealedfekaagarglstesiliprqs

d1ggla_ ppNLTGYYRFVSQKNmedylqalnislavrkiALLLKPDKEIEHQGNH-MTVRTLS--tFRNYTVQfdvgvefeedlrsvdgrkcqtIVTWEEehLVCVQkgevpNRGWRHWLE--GEMLYLELTAR--DAVCEQVFRKV------------------------------h

d1xkia_ --DVSGTWYLKAMT-----------------vnlesVTPMTLTTLeGGNLEAKVTMsgrCQEVKAVLEK--------------------TDEP-GKYT---adggkhvAYIIRShvKDHYIFYSEGEgkPVRGVKLVGRDPknnlealedfekaagarglstesiliprqs

d1kqxa_ paDFNGTWEMLSNDnfedvmkaldidfatrkiavhlKQTKVIVQN-GDKFETKTLS--tFRNYEVNFVIgeefdeqtkgldnrtvktlvKWDGdKLVCvqkgekenrgWKQWIE--GDLLHLEIHCQ--DKVCHQVFKKKN------------------------------

d1xkia_ --DVSGTWYLKAMTV-----------------NLESVTPMTLTTLEGGnLEAKVTMsgrCQEVKAV------------------lEKTDEP--GKYTADG--gKHVAYIIRShvKDHYIFYSEGEgkPVRGVKLVGRDpknnlealedfekaagarglstesiliprqs

d1liba_ cdAFVGTWKLVSSENfddymkevgvgfatrkvAGMAKPNMIISVNGDL-VTIRSES--tFKNTEISfklgvefdeitaddrkvksIITLDGgaLVQVQKWdgkSTTIKRKRD--GDKLVVECVMK--GVTSTRVYERA-------------------------------

d1xkia_ --DVSGTWYLKAMTV-----------------NLESVTPMTLTTLEgGNLEAKVTMsgrCQEVKAV--------------------lEKTDEP--GKYTADG--gKHVAYIIRShvKDHYIFYSEGEgkPVRGVKLVGRDpknnlealedfekaagarglstesiliprqs

d1lpja_ paDLSGTWTLLSSDNfegymlalgidfatrkiAKLLKPQKVIEQNG-DSFTIHTNS--sLRNYFVKfkvgeefdednrgldnrkcksLVIWDNdrLTCIQKGekkNRGWTHWIE--GDKLHLEMFCE--GQVCKQTFQRA-------------------------------

d1xkia_ ---------------------------DVSGTWYLKA-------------------------------mtvnlESVTPMTLTTleggnLEAKVTMsgrCQEVKAVLEKTDEP------------GKYTADG----gkHVAYIIRS----hvkDHYIFYSEGE-----gkpvrGVKLVGR-DPKN--NLEALedfekaagarglstesiliprqs

d1oeja_ gkplteveqkaangvfddanvqnrtlsDWDGVWQSVYpllqsgkldpvfqkkadadktktfaeikdyyhkgyaTDIEMIGIED-----GIVEFHR--nNETTSCKYDYDGYKiltyksgkkgvrYLFECKDpeskapKYIQFSDHiiaprksSHFHIFMGNDsqqsllnemeNWPTYYPyQLSSeeVVEEM--------------------msh

d1xkia_ --DVSGTWYLKAMTV-----------------NLESVTPMTLTTLEgGNLEAKVTMsgrCQEVKAV--------------------lEKTDEP--GKYTA--dggKHVAYIIRShvKDHYIFYSEGEgkPVRGVKLVGRDpknnlealedfekaagarglstesiliprqs

d1opaa_ tkDQNGTWEMESNENfegymkaldidfatrkiAVRLTQTKIIVQDG-DNFKTKTNS--tFRNYDLDftvgvefdehtkgldgrnvktLVTWEGntLVCVQkgekeNRGWKQWVE--GDKLYLELTCG--DQVCRQVFKKK-------------------------------

d1xkia_ DVSGTWYLKAMTV-----------------NLESVTPMTLTTLEgGNLEAKVTMsgrCQEVKAVLEK-----------------TDEP---GKYTadgGKHVAYIIRShvKDHYIFYSEGEgkPVRGVKLVGRDpknnlealedfekaagarglstesiliprqs

d1p6pa_ AFNGTWNVYAQENyenflrtvglpediikvAKDVNPVIEIEQNG-NEFVVTSKT--pKQTHSNSFTVgkeseitsmdgkkikvtVQLEggkLICK--sDKFSHIQEVN--GDEMVEKITIG--SSTLTRKSKRV-------------------------------

d1xkia_ -------dvSGTWYL---------kamtVNLEsVTPMTLTTLEgGNLEAKVTM--SGRCQEVKAVLEKtdepgKYTA----------DGGK----------------------hVAYIIrshvkDHYIFYSEGEG---KPVRGVKLVGRDPknnlealedfekaagarglstesiliprqs

d1r0ua_ gfqsnamkqETPITLhvksvieddgnqeVIEF-RTTGFYYVKQ-NKVYLSYYEehDLGKVKTIVKVSE-----GEVLvmrsgavkmnQRFVtgastiakykmsfgelelktstkSIQSD--ldeEKGRISIAYDMhvgHLHNMTITYEGGT------------------------------

d1xkia_ DVSGTWYLKAMTV-----------------NLESVTPMTLTTLEgGNLEAKVTMsgrCQEVKAVLEK-----------------TDEP---GKYTadgGKHVAYIIRShvKDHYIFYSEGEgkPVRGVKLVGRDpknnlealedfekaagarglstesiliprqs

d1tvqa_ AFSGTWQVYAQENyeeflkalalpedlikmARDIKPIVEIQQKG-DDFVVTSKT--pRQTVTNSFTLgkeadittmdgkklkctVHLAngkLVTK--sEKFSHEQEVK--GNEMVETITFG--GVTLIRRSKRV-------------------------------

d1xkia_ -------------------------------------------------------------------------------------------------------------------------------------------------------------DVSGTWYLKAM---------------------------tvnlESVTPMTLTTLEgGNLEAKVTMsgrcQEVKAVL------------------eKTDEP---GKYTADG----gKHVAYIIRSHV--KDHYIFYSEGEgkPVRGVKLVGRD----------------pknnlealedfekaagarglstesiliprqs

d1vpra1 ekgfeagdnklggalnakhvekygdnfkngxhkpefhedglhkpxevggkkfesgfhyllechelggknasggyggplcedpygsevqaxtekllkeadsdrtlcfnnfqdpcpqltkeqvaxckgfdygdktlklpcgplpwpaglpepgyvpktnPLHGRWITVSGgqaafikeaiksgmlgaaeankivadtdhhqTGGXYLRINQFG-DVCTVDASV-akfARAKRTWksghyfyeplvsggnllgvWVLPEeyrKIGFFWExesgrCFRIERRAFPVgpYTFXRQATEVG--GKISFVFYVKVsndpesdpiplqsrdytalagrdnaptnlgkpyptlakdldypkkrd

d1xkia_ -DVSGTWYLKAMTV-------------------NLESVTPMTLTTLEgGNLEAKVTMsgrCQEVKAVLEK-----------------TDEP----GKYT-----adggKHVAYIIRSHVkDHYIFYSEGEgkPVRGVKLVGRDpknnlealedfekaagarglstesiliprqs

d1xcaa_ pNFSGNWKIIRSENfeellkvlgvnvmlrkiavAAASKPAVEIKQEG-DTFYIKTST--tVRTTEINFKVgeefeeqtvdgrpckslVKWEsenkMVCEqkllkgegpKTSWTMELTND-GELILTMTAD--DVVCTRVYVRE-------------------------------

d1xkia_ -------------DVSGTWYLKAMTV----------NLESVTPMTLTTLEGGNLEAKVTM--SGRCQEVKAVLEKTDEPGKYTAD-ggKHVAYIIRSHVKDHYIFYSEGEGKP--VRGVKLVGRDPKNNLEALEDFEKAAGARGLstESILIPRQ--------s

d1yupa1 iivtqtmkdldvqKVAGTWYSLAMAAsdislldaqsAPLRVYVEELKPTPGGDLEILLQKweNGKCAQKKIIAEKTEIPAVFKIDalnENKVLVLDTDYKKYLLFCMENSAEPeqSLACQCLVRTPEVDDEAMEKFDKALKALPM--HIRLSFNPtqleeqcrv

d1xkia_ -----------DVSGTWYLkAMTV------nleSVTPMTLTTLEGGNLEAKVT-----msgrCQEVKAVLEKTDEP--------gkytaDGGK-------------------------HVAYIIRShvKDHYIFYSEGE---GKPVR-GVKLVGRDpknnlealedfekaagarglstesiliprqs

d2a13a1 ppvhpfvaplsYLLGTWRG-QGEGeyptipsfrYGEEIRFSHSGKPVIAYTQKtwklesgapXHAESGYFRPRPDGsievviaqstglvEVQKgtynvdeqsiklksdlvgnaskvkeISREFELV--DGKLSYVVRXStttNPLQPhLKAILDKL-------------------------------

d1xkia_ -DVSGTWYLKAMT-----------------vnlesVTPMTLTTLEgGNLEAKVTmsgrCQEVKAVLEK-----------------TDEP----GKYTadgGKHVAYIIRShvKDHYIFYSEGEgkPVRGVKLVGRDpknnlealedfekaagarglstesiliprqs

d2f73a1 mSFSGKYQLQSQEnfeafmkaiglpeeliqkgkdiKGVSEIVQNG-KHFKFTIT--agSKVIQNEFTVgeeceletmtgekvktvVQLEgdnkLVTT--fKNIKSVTELN--GDIITNTMTLG--DIVFKRISKRI-------------------------------

d1xkia_ ----------DVSGT-----wylkamtvnlesvtpMTLTTLEGGNLEAKVTM-----SGRCQEVKAVLEKTdEPGKYTAD-------ggKHVAYIIRShvKDHYIFYSEGE-------------GKPVRGVKLVGRDPknnlealedfekaagarglsTESILIP--rqs

d2fr2a1 dlapalqalsPLLGSwagrgagkyptirpfeyleeVVFAHVGKPFLTYTQQTravadGKPLHSETGYLRVC-RPGCVELVlahpsgiteIEVGTYSVT--GDVIELELSTRadgsiglaptakeVTALDRSYRIDGDE------lsyslqmravgqplQDHLAAVlhrqr

d1xkia_ ------------dvSGTWYLKamtvnleSVTPMTLTTLEGGNLEAKVTmsgrcqEVKAV-LEKT-------depGKYTAdGGKHVAYIIRShVKDHYIFYSEGE------------------------------gkPVRGVKLVGRD---------pknnlealedfekaagarglstesiliprqs

d2gc9a1 xtktfktlddflgtHFIYTYD-------NGWEYEWYAKNDHTVDYRIH-ggxvaGRWVTdQKADivxltegiykISWTE-PTGTDVALDFXpNEKKLHGTIFFPkwveehpeitvtyqnehidlxeqsrekyatypKLVVPEFANITyxgdagqnnedviseapykexpndirngkyfdqnyhrlnk

d1xkia_ --------DVSGTWY------lkamtvnlesvtPMTLTTLEGGNLEAKVTMsgrcQEVKAVLEKTdePGKYTADG---gKHVAYIIRShvkdhYIFYSEGE-gkpvrgvklvgrdpknnlealedfekaagarglstesiliprqs

d2o62a1 erpllqinDLLGEWRgqavtiyrdlrppdiystTLKIQLDDAGRLXQSTSF--geRTITSTATIK--GSIVLFDQdpekQVQVLLLPD-----GASATSPLkvqlrqplfleagwliqsdlrqrxirsyndkgewvsltlvteerv

d1xkia_ ---------------DVSG--TWYLKAMTV------nleSVTPMTLTTLEGgNLEAKVT----mSGRCQEVKAVLEKTdEPGKYTAD----------------ggKHVAYIIRShVKDHYIFYSEGE--GKPV-RGVKLVGRDpKNNLeALEDFEKAAGARGLStESILIPRQ---------------s

d2ofmx1 actknaiaqtgfnkdKYFNgdVWYVTDYLDlepddvpkrYCAALAAGTASG-KLKEALYhydpkTQDTFYDVSELQVE-SLGKYTANfkkvdkngnvkvavtagnYYTFTVMYA-DDSSALIHTCLHkgNKDLgDLYAVLNRN-KDAA-AGDKVKSAVSAATLEfSKFISTKEnncaydndslkslltk

d1xkia_ -------------DVSGTWYLKAMTV---------NLESVTPMTLTTLeGGNLEAKVTM--SGRCQEVKAVLEKTDEPGKYTAD-ggKHVAYIIRSHVKDHYIFYSEGE--GKPVRGVKLVGRDPkNNLE-ALEDFEKAAGARGLSTESILI-PRQS--

d2ozqa1 eeasstgrnfnveKINGEWHTIILASdkrekiednGNFRLFLEQIHVL-EKSLVLKFHTvrDEECSELSMVADKTEKAGEYSVTydgFNTFTIPKTDYDNFLMAHLINEkdGETFQLMGLYGREP-DLSSdIKERFAQLCEEHGILRENIIDlSNANrc

d1xkia_ ---------------DVSG--TWYLKAMTV------nleSVTPMTLTTLEGgNLEAKVTM----SGRCQEVKAVLEKTdEPGKYTAD----------------GGKHVAYIIRShVKDHYIFYSEGE---GKPVRGVKLVGRDpKNNLeALEDFEKAAGARGLStESILIPRQ---------------s

d3np1a_ kctknalaqtgfnkdKYFNgdVWYVTDYLDlepddvpkrYCAALAAGTASG-KLKEALYHydpkTQDTFYDVSELQEE-SPGKYTANfkkvekngnvkvdvtsGNYYTFTVMYA-DDSSALIHTCLHkgnKDLGDLYAVLNRN-KDTN-AGDKVKGAVTAASLKfSDFISTKDnkceydnvslkslltk

d1yupa1 -----iIVTQTMKDLDVQKV-AGTWYSLAmaasdislldaqsaplrvyveelkPTPG-----gdleillqKWENgkcAQKKIIAEK-TEIPaVFKIDA------LNENKVLVLDTDYKKYLLFCMENS-aepeQSLACQCLVRTPEvddeamekfdkalkalpmhirlsfnPTQLeEQCR-v

d1avgi_ aegddcSIEKAMGDFKPEEFfNGTWYLAH----------gpgvtspavcqkftTSGSkgftqiveigynkFESN---VKFQCNQVDnKNGE-QYSFKCkssdntEFEADFTFISVSYDNFALVCRSITftsqpKEDRYLVFERTKS-------------------------DTDP-DAKEic

d1yupa1 iivtqtmkdldvqKVAGTWYSLAMAA--SDIS------llDAQSAPLRV-YVEELKPTPgGDLEILLQkwengKCAQKKIIAEK----------------TEIP-----AVFK----idalneNKVLVLDTDykKYLLFCMEnsaePEQSLACQCLVRTpevddeamekfdkalkalpmhirlsfnptqleeqcrv

d1cbia_ ------------pNFAGTWKMRSSENfdELLKalgvnamlRKVAVAAASkPHVEIRQDG-DQFYIKTS----tTVRTTEINFKVgegfeeetvdgrkcrsLPTWenenkIHCTqtllegdgpkTYWTRELAN--DELILTFG----ADDVVCTRIYVRE-------------------------------------

d1yupa1 iivtqtmkdlDVQKVAGTWYSLAMAA--SDIS----lldAQSAPLRVYVEELKPTP--GGDLEILLQkwengkCAQKKIIAE------------------kTEIP----AVFKidalneNKVLVlDTDYkKYLLFCMEnsaePEQSLACQCLVRTpevddeamekfdkalkalpmhirlsfnptqleeqcrv

d1ftpa_ ----------VKEFAGIKYKLDSQTNfeEYMKaigvgaiERKAGLALSPVIELEILdgDKFKLTSKT------AIKNTEFTFklgeefdeetldgrkvkstITQDgpnkLVHEqkgdhpTIIIR-EFSK-EQCVITIK----LGDLVATRIYKAQ-------------------------------------

d1yupa1 iivtqtmkdldvQKVAGTWYSLAMAA--SDIS------llDAQSAplRVYVEELKPTPGgDLEILLQKWengkCAQKKII--------------------aeKTEI--PAVFK-idalneNKVLVlDTDYkKYLLFCMENsaepEQSLACQCLVRTpevddeamekfdkalkalpmhirlsfnptqleeqcrv

d1ggla_ -----------pPNLTGYYRFVSQKNmeDYLQalnislavRKIAL-lLKPDKEIEHQGN-HMTVRTLST----FRNYTVQfdvgvefeedlrsvdgrkcqtiVTWEeeHLVCVqkgevpnRGWRH-WLEG-EMLYLELTA----RDAVCEQVFRKV------------------------------------h

d1yupa1 iivtqtmkdldvQKVAGTWYSLAMAA--SDIS------llDAQSAplRVYVEELKPTPgGDLEILLQKWengkCAQKKIIAEK-------------------TEIP---AVFK-idalneNKVLVlDTDYkKYLLFCMEnsaePEQSLACQCLVRTPevddeamekfdkalkalpmhirlsfnptqleeqcrv

d1kqxa_ -----------pADFNGTWEMLSNDNfeDVMKaldidfatRKIAV-hLKQTKVIVQNG-DKFETKTLST----FRNYEVNFVIgeefdeqtkgldnrtvktlVKWDgdkLVCVqkgekenRGWKQ-WIEG-DLLHLEIH----CQDKVCHQVFKKKN------------------------------------

d1yupa1 iivtqtmkdldVQKVAGTWYSLAMAA--SDIS-----lldAQSAPlRVYVEELKPTPGgdLEILLQKWengkCAQKKII------------------aeKTEIP--AVFK-idalneNKVLVLDTDykKYLLFCMEnsaePEQSLACQCLVRTpevddeamekfdkalkalpmhirlsfnptqleeqcrv

d1liba_ -----------CDAFVGTWKLVSSENfdDYMKevgvgfatRKVAGmAKPNMIISVNGD-lVTIRSEST----FKNTEISfklgvefdeitaddrkvksiITLDGgaLVQVqkwdgksTTIKRKRDG--DKLVVECV----MKGVTSTRVYERA-------------------------------------

d1yupa1 iivtqtmkdldvQKVAGTWYSLAMAASD--------isllDAQSAplRVYVEELKPTPgGDLEILLQKWEngkcAQKKIIAEK------------------TEIP----AVFK-idalneNKVLVlDTDYkKYLLFCMENSAepeqSLACQCLVRTpevddeamekfdkalkalpmhirlsfnptqleeqcrv

d1lpja_ -----------pADLSGTWTLLSSDNFEgymlalgidfatRKIAK-lLKPQKVIEQNG-DSFTIHTNSSL----RNYFVKFKVgeefdednrgldnrkcksLVIWdndrLTCIqkgekknRGWTH-WIEG-DKLHLEMFCEG----QVCKQTFQRA-------------------------------------

d1yupa1 --------------iivtqtmKDLDVQKVAGTWYSLA---------------------maasdislldaqsapLRVYVEELKPtpggdLEILLQKWengkCAQKKIIAEKTEI------------pAVFKID---alnENKVLVLD----tdYKKYLLFCMENSA--EPEQ-slACQCLVR-TPEV--DDEAMEkfdkalkalpmhirlsfnptqleeqcrv

d1oeja_ gkplteveqkaangvfddanvQNRTLSDWDGVWQSVYpllqsgkldpvfqkkadadktktfaeikdyyhkgyaTDIEMIGIED-----GIVEFHRN----NETTSCKYDYDGYkiltyksgkkgvrYLFECKdpeskaPKYIQFSDhiiaprKSSHFHIFMGNDSqqSLLNemeNWPTYYPyQLSSeeVVEEMM--------------------------sh

d1yupa1 iivtqtmkdldvQKVAGTWYSLAMAA--SDIS-----lldAQSAPlrvYVEELKPTPgGDLEILLQKWengkCAQKKII-------------------aeKTEIP---AVFK-idalneNKVLVlDTDYkKYLLFCMENsaepEQSLACQCLVRTpevddeamekfdkalkalpmhirlsfnptqleeqcrv

d1opaa_ -----------tKDQNGTWEMESNENfeGYMKaldidfatRKIAVrltQTKIIVQDG-DNFKTKTNST----FRNYDLDftvgvefdehtkgldgrnvktLVTWEgntLVCVqkgekenRGWKQ-WVEG-DKLYLELTC----GDQVCRQVFKKK-------------------------------------

d1yupa1 iivtqtmkdldvqKVAGTWYSLAMAA--SDIS-----lldAQSAPlRVYVEELKPTPgGDLEILLQKWengkCAQKKIIAEK----------------TEIP----AVFKidalneNKVLVlDTDYkKYLLFCMENsaepEQSLACQCLVRTpevddeamekfdkalkalpmhirlsfnptqleeqcrv

d1p6pa_ -------------AFNGTWNVYAQENyeNFLRtvglpediIKVAKdVNPVIEIEQNG-NEFVVTSKTP----KQTHSNSFTVgkeseitsmdgkkikvTVQLeggkLICK---sdkFSHIQ-EVNG-DEMVEKITI----GSSTLTRKSKRV-------------------------------------

d1yupa1 iivtqtmkdldvqkvAGTWYSlAMAASD------ISLLdaqsaplRVYVEELKPTPgGDLEILLQKWENGKCAQKKIIAEKteipavFKIDA--------LNEN------------------------KVLVlDTDYkKYLLFCMENSA-epEQSLACQCLVRTPevddeamekfdkalkalpmhirlsfnptqleeqcrv

d1r0ua_ ------gfqsnamkqETPITL-HVKSVIeddgnqEVIE-------FRTTGFYYVKQ-NKVYLSYYEEHDLGKVKTIVKVSE------GEVLVmrsgavkmNQRFvtgastiakykmsfgelelktstkSIQS-DLDE-EKGRISIAYDMhvgHLHNMTITYEGGT------------------------------------

d1yupa1 iivtqtmkdldvqKVAGTWYSLAMAA--SDIS------llDAQSAplRVYVEELKPTPgGDLEILLQKWengkCAQKKIIAEK----------------TEIP----AVFKidalneNKVLVlDTDYkKYLLFCMEnsaePEQSLACQCLVRTpevddeamekfdkalkalpmhirlsfnptqleeqcrv

d1tvqa_ -------------AFSGTWQVYAQENyeEFLKalalpedlIKMAR-dIKPIVEIQQKG-DDFVVTSKTP----RQTVTNSFTLgkeadittmdgkklkcTVHLangkLVTK---sekFSHEQ-EVKG-NEMVETIT----FGGVTLIRRSKRV-------------------------------------

d1yupa1 ---------------------------------------------------------------------------------------------------IIVTQ---------------------------------------------tmkdldvQKVAGTWYSLAM-------------------aasdISLLdaqsapLRVY-VEELKPTPgGDLEILLQKWengkCAQKK-------------------iiaEKTEIP-AVFKID-----alNENKvLVLDTD--yKKYLLFCMENsaepEQSLACQCLVRT----------pevddeamekfdkalkalpmhirlsfnptqleeqcrv

d1vpra1 ekgfeagdnklggalnakhvekygdnfkngxhkpefhedglhkpxevggkkfesgfhyllechelggknasggyggplcedpygsevqaxtekllkeadSDRTLcfnnfqdpcpqltkeqvaxckgfdygdktlklpcgplpwpaglpepgyvpktNPLHGRWITVSGgqaafikeaiksgmlgaaeankiVADT---dhhQTGGxYLRINQFG-DVCTVDASVA---kFARAKrtwksghyfyeplvsggnllgvWVLPEEyRKIGFFwexesgrCFRI-ERRAFPvgpYTFXRQATEV----GGKISFVFYVKVsndpesdpiplqsrdytalagrdnaptnlgkpyptlakdldypkkrd

d1yupa1 iivtqtmkdldvQKVAGTWYSLAMAA--SDIS------llDAQSA-plRVYVEELKPTPgGDLEILLQKWengkCAQKKII------------------aeKTEIP---AVFK----idalnenKVLVlDTDYKKYLLFCMENSAepeqSLACQCLVRTpevddeamekfdkalkalpmhirlsfnptqleeqcrv

d1xcaa_ ------------PNFSGNWKIIRSENfeELLKvlgvnvmlRKIAVaaaSKPAVEIKQEG-DTFYIKTSTT----VRTTEINfkvgeefeeqtvdgrpckslVKWESenkMVCEqkllkgegpktSWTM-ELTNDGELILTMTADD----VVCTRVYVRE-------------------------------------

d1yupa1 -iivtqtmkdLDVQkvAGTWYSlaMAASD-ISLLdaqsaplRVYVeELKPTP----GGDLEILLQKWEngKCAQ-KKIIAEKTE---ipavFKIDA------------------------------lnenkvLVLDTDykKYLLFCMENS----AEPEqsLACQCLVRTpevddeamekfdkalkalpmhirlsfnptqleeqcrv

d2a13a1 ppvhpfvaplSYLL--GTWRGQ-gEGEYPtIPSF-------RYGE-EIRFSHsgkpVIAYTQKTWKLE--SGAPxHAESGYFRPrpdgsieVVIAQstglvevqkgtynvdeqsiklksdlvgnaskvkeisREFELV-dGKLSYVVRXStttnPLQP--HLKAILDKL-------------------------------------

d1yupa1 iivtqtmkdldvQKVAGTWYSLAMAAsdISLL---------DAQSAplrvYVEELKPTPgGDLEILLQKWengkCAQKKIIAEK-----------------TEIP----AVFKidalneNKVLVlDTDYkKYLLFCMENsaepEQSLACQCLVRTpevddeamekfdkalkalpmhirlsfnptqleeqcrv

d2f73a1 ------------MSFSGKYQLQSQEN-fEAFMkaiglpeelIQKGK-dikGVSEIVQNG-KHFKFTITAG----SKVIQNEFTVgeeceletmtgekvktvVQLEgdnkLVTT---fknIKSVT-ELNG-DIITNTMTL----GDIVFKRISKRI-------------------------------------

d1yupa1 iivtqtmkdLDVQKVAGTWyslamaasdisllDAQSaplrvyvEELKPTPGGDLEILLQKWE---NGKCAQKKIIAEKTeIPAVFKIDA------LNENKVLvLDTDyKKYLLFCMENSA-----------epEQSLACQCLVRTPevddeamekfdkalkalPMHIRLSFnptqleeqcrv

d2fr2a1 ---dlapalQALSPLLGSW-agrgagkyptirPFEY----leeVVFAHVGKPFLTYTQQTRAvadGKPLHSETGYLRVC-RPGCVELVLahpsgiTEIEVGT-YSVT-GDVIELELSTRAdgsiglaptakevTALDRSYRIDGDE----lsyslqmravgqpLQDHLAAV------lhrqr

d1yupa1 iivtqtmkdLDVQKV-AGTWYSlAMAAsdislldaqsaplRVYVEELKPTpggdLEILLQK-wengkCAQKK-IIAEKTEI---pAVFKIDA--lNENKVLVLDTdykkyLLFCMENSA--------------------------------epEQSLACQCLVRtpevddeamekfdkalkalpmhirlsfnpTQLEE------------qcrv

d2gc9a1 ----xtktfKTLDDFlGTHFIY-TYDN------------gWEYEWYAKND----HTVDYRIhggxvaGRWVTdQKADIVXLtegiYKISWTEptgTDVALDFXPN----eKKLHGTIFFpkwveehpeitvtyqnehidlxeqsrekyatypkLVVPEFANITY-----------xgdagqnnedviseapykEXPNDirngkyfdqnyhrlnk

d1yupa1 iivtqtmKDLDVQKVAGTWYSlAMAASD---ISLLdaqsaplRVYVEELKPTP--ggdlEILLQKwengkcAQKKII-----------------aEKTEIP----AVFK----idalNENKVLVlDTDYKKYLLFCMENSA--epEQSLACQCLVRTpevddeamekfdkalkalpmhirlsfnptqleeqcrv

d2o62a1 -----erPLLQINDLLGEWRG-QAVTIYrdlRPPD-------IYSTTLKIQLDdagrlxQSTSFG------ERTITStatikgsivlfdqdpekqVQVLLLpdgaSATSplkvqlrqPLFLEAG-WLIQSDLRQRXIRSYNdkgeWVSLTLVTEERV-------------------------------------

d1yupa1 --IIVTQTMKDLDVQKVA--GTWYSLAMAASDISLLDAQsaplrvYVEELKPTPGgDLEILLQKW--eNGKCAQKKIIAEKTeIPAVFKIDA---------------lNENKVLVLDTDyKKYLLFCMENS--AEPEqSLACQCLVRTPE-VDDE-AMEKFDKALKalpmhIRLSFNpTQLEEQ--------crv

d2ofmx1 acTKNAIAQTGFNKDKYFngDVWYVTDYLDLEPDDVPKR----ycAALAAGTASG-KLKEALYHYdpkTQDTFYDVSELQVE-SLGKYTANFkkvdkngnvkvavtagNYYTFTVMYAD-DSSALIHTCLHkgNKDL-GDLYAVLNRNKDaAAGDkVKSAVSAATL--efsKFISTK-ENNCAYdndslkslltk

d1yupa1 iivtqTMKD--LDVQKVAGTWYSLAMAASDISLLDaQSAPLRVYVEELKPTpGGDLEILLQKWENGKCAQKKIIAEKTEIPAVFKIDALNENKVLVLDTDYKKYLLFCMENS--AEPEqsLACQCLVRTPEVDDEAMEKFDKALKALPMH--IRLSFNPtQLEEqcrv

d2ozqa1 --eeaSSTGrnFNVEKINGEWHTIILASDKREKIE-DNGNFRLFLEQIHVL-EKSLVLKFHTVRDEECSELSMVADKTEKAGEYSVTYDGFNTFTIPKTDYDNFLMAHLINEkdGETF--QLMGLYGREPDLSSDIKERFAQLCEEHGILreNIIDLSN-ANRC----

d1yupa1 --iIVTQTMKDLDVQKVA--GTWYSLAMAASDISllDAQSaplrvYVEELKPTPGgDLEILLQKW--eNGKCAQKKIIAEKTeIPAVFKIDA---------------lNENKVLVLDTDyKKYLLFCMENS---AEPEqsLACQCLVRTPE-VDDE-AMEKFDKALKalpmhIRLSFNPTQlEEQC--------rv

d3np1a_ kctKNALAQTGFNKDKYFngDVWYVTDYLDLEPDdvPKRY----cAALAAGTASG-KLKEALYHYdpkTQDTFYDVSELQEE-SPGKYTANFkkvekngnvkvdvtsgNYYTFTVMYAD-DSSALIHTCLHkgnKDLG--DLYAVLNRNKDtNAGDkVKGAVTAASL--kfsDFISTKDNK-CEYDnvslkslltk

d2a13a1 pPVHPFVAPLSYLLGTWRGQGEGEYPTIPSFRYGEEIRFSHSGKPVIAYTQKTWKLESGAPXHAESGYFRPRPDGSIEVVIAQSTGLVEVQKGTYNVD--EQSIKLKS---DLVGN---ASKVKEISREFELVDGKLSYVVRXSTTTNPLQPHLKAILDKL-

d2fr2a1 -DLAPALQALSPLLGSWAGRGAGKYPTIRPFEYLEEVVFAHVGKPFLTYTQQTRAVADGKPLHSETGYLRVCRPGCVELVLAHPSGITEIEVGTYSVTgdVIELELSTradGSIGLaptAKEVTALDRSYRIDGDELSYSLQMRAVGQPLQDHLAAVLHRQr

d2a13a1 ppvhpfvaPLSYLLGTWRGQGEGEYPT-IPSFRYGEEIRFSHSGKPVIAYTQKTWklesgapXHAESGYFRPRPdGSIEVViaqstgLVEVQKGTYNvDEQSIKLKSDLVgnASKVKEISREFELVDG-KLSYVVRXSTTTNPLQPHLKAILDKL

d2o62a1 ---erpllQINDLLGEWRGQAVTIYRDlRPPDIYSTTLKIQLDDAGRLXQSTSFG-------ERTITSTATIKG-SIVLFD----qdPEKQVQVLLL-PDGASATSPLKV--QLRQPLFLEAGWLIQSdLRQRXIRSYNDKGEWVSLTLVTEERV

d2f73a1 --------------MSFS------GKYQLqsqenfeafmkaiglpeeliqkgkdikgvseivqngkhfkftitagskviqneftvgeeceletmtgekvkTVVQ-LEGDnKLVTTFK-----NIKSVTELNG------DIITNTMTLG-DIVFKRISKRI--------------

d1avgi_ aegddcsiekamgdFKPEeffngtWYLAH-------------------------------gpgvtspavcqkfttsgskgftqiveigynkfesnvkfqcNQVDnKNGE-QYSFKCKssdntEFEADFTFISvsydnfALVCRSITFTsQPKEDRYLVFErtksdtdpdakeic

d2f73a1 --------------------------mSFSGKYQLQS--------------qenfeafmkaiglpeeliqkgkdikGVSEIVQngkhFKFTITAGSKVIQNEFTvgeeceletmtgekvktvVQLEG----------DNKLVT----tfkniKSVTELN-------GDIITNTMT-------lgdivfKRISKRI--------------

d1oeja_ gkplteveqkaangvfddanvqnrtlsDWDGVWQSVYpllqsgkldpvfqkkadadktktfaeikdyyhkgyatdiEMIGIED----GIVEFHRNNETTSCKYD------------------YDGYKiltyksgkkgVRYLFEckdpeskapKYIQFSDhiiaprkSSHFHIFMGndsqqsllnemenWPTYYPYqlsseevveemmsh

d2f73a1 msfsgkyqlqsqenfeafmkaiglpeeliqkgkdikgvseivqngkhfkftitagskviqneftvgeeceletmtgekvktVVQLEGD---NKLVTTFK----NIKSVTELNGDIITNTMTLGdIVFKRISKRI--------------------------------------------------------

d1r0ua_ -----------------------------------------------gfqsnamkqetpitlhvksvieddgnqeviefrtTGFYYVKqnkVYLSYYEEhdlgKVKTIVKVSEGEVLVMRSGA-VKMNQRFVTGastiakykmsfgelelktstksiqsdldeekgrisiaydmhvghlhnmtityeggt

d2f73a1 ------------------------------------------------------------------------------------------------------------------------------------------------------------MSFSGKYQLQSQ----------enfeafmkaiglpeeliqkgkdiKGVSEIVQNGKHFKFTITaGSKV--IQNEFTVGEECELETmTGEKVKTVVQLEGDNKLVTTFK------NIKSVTELNG----DIITNTMTLGDIVFKRISKRI-----------------------------------------------

d1vpra1 ekgfeagdnklggalnakhvekygdnfkngxhkpefhedglhkpxevggkkfesgfhyllechelggknasggyggplcedpygsevqaxtekllkeadsdrtlcfnnfqdpcpqltkeqvaxckgfdygdktlklpcgplpwpaglpepgyvpktNPLHGRWITVSGgqaafikeaiksgmlgaaeankivadtdhhqtgGXYLRINQFGDVCTVDAS-VAKFarAKRTWKSGHYFYEPLvSGGNLLGVWVLPEEYRKIGFFWexesgrCFRIERRAFPvgpyTFXRQATEVGGKISFVFYVKVsndpesdpiplqsrdytalagrdnaptnlgkpyptlakdldypkkrd

d2f73a1 ----------MSFSGKYQLqSQENFEAfmkaiglpeeliqkgKDIKGVSEIVQN-GKHFKFTITAG-------SKVIQNEFTV----GEECELETMTGEKVKTVVQLEG-DNKLVTTF---------KNIKSVTELNGDIITNTMTL------GDIVFKRISKRI

d2a13a1 ppvhpfvaplSYLLGTWRG-QGEGEYP-----------tipsFRYGEEIRFSHSgKPVIAYTQKTWklesgapXHAESGYFRPrpdgSIEVVIAQSTGLVEVQKGTYNVdEQSIKLKSdlvgnaskvKEISREFELVDGKLSYVVRXstttnpLQPHLKAILDKL

d2f73a1 ---------MSFSGKYQLqSQENFEAfmkaiglpeeliqkgKDIKGVSEIVQN-GKHFKFTITAG-------SKVIQNEFTV----GEECELETMTGEKVKTVVQLEGD---NKLVTTFK---------------NIKSVTELNGDIITNTMTL------GDIVFKRISKRI-

d2fr2a1 dlapalqalSPLLGSWAG-RGAGKYP-----------tirpFEYLEEVVFAHVgKPFLTYTQQTRavadgkpLHSETGYLRVcrpgCVELVLAHPSGITEIEVGTYSVTgdvIELELSTRadgsiglaptakevtALDRSYRIDGDELSYSLQMravgqpLQDHLAAVLHRQr

d2f73a1 -----------mSFSGKYQLQSqenfeafmkaiglpeeliqkgkdiKGVSEIVQNGK-HFKFTITAGS-KVIQNEFtvgeeceletmtgekvktVVQLEGD----NKLVTTF-KNIKSVTELNG--DIITNTMTLG--------------------------------DIVFKRISKRI----------------------------------------

d2gc9a1 xtktfktlddflGTHFIYTYDN------------------------GWEYEWYAKNDhTVDYRIHGGXvAGRWVTD-----------------qKADIVXLtegiYKISWTEpTGTDVALDFXPneKKLHGTIFFPkwveehpeitvtyqnehidlxeqsrekyatypKLVVPEFANITyxgdagqnnedviseapykexpndirngkyfdqnyhrlnk

d2f73a1 -------MSFSGKYQLqSQENFEAfmkaiglpeeliqkgKDIKGVSEIVQN-gkHFKFTITAGSKVIQNEFTV---GEECELEtmtgEKVKTVVQLEGDNKLVTTF-------KNIKSVTELN-GDIITNTMTLG------DIVFKRISKRI

d2o62a1 erpllqiNDLLGEWRG-QAVTIYR----------dlrppDIYSTTLKIQLDdagRLXQSTSFGERTITSTATIkgsIVLFDQD----PEKQVQVLLLPDGASATSPlkvqlrqPLFLEAGWLIqSDLRQRXIRSYndkgewVSLTLVTEERV

d2fr2a1 dlapalqALSPLLGSWAGRGAGKYPTI-RPFEYLEEVVFAHVGKPFLTYTQQTRavadgkpLHSETGYLRVCrPGCVELVLAhpsgITEIEVGTYSVTgdvIELELSTradGSIGlaptakEVTALDRSYRID-GDELSYSLQMRAVGQPLQDHLAAVLHRQr

d2o62a1 --erpllQINDLLGEWRGQAVTIYRDLrPPDIYSTTLKIQLDDAGRLXQSTSFG-------ERTITSTATIK-GSIVLFDQD----PEKQVQVLLLPD---GASATSP---LKVQ-----lRQPLFLEAGWLIqSDLRQRXIRSYNDKGEWVSLTLVTEERV-

d2gc9a1 --------------------------------------------------------------------------------------------------------------------------------------------------xtktfktLDDFlgtHFIYTYDN----------------------------------GWEYEWYAKNDhTVDYRIHggXVAG-RWVTDQK-----------------ADIVXLtegiYKISWTEPT-----GTDVALDFXPN--EKKLHGTIFFPkwveehpeitvtyqnehidlxeqsrekyatypKLVVPEFANITYXGdagqnnedVISE------------apykexpndirngkyfdqnyhrlnk

d1vpra1 ekgfeagdnklggalnakhvekygdnfkngxhkpefhedglhkpxevggkkfesgfhyllechelggknasggyggplcedpygsevqaxtekllkeadsdrtlcfnnfqdpcpqltkeqvaxckgfdygdktlklpcgplpwpaglpepgyvPKTN-plHGRWITVSggqaafikeaiksgmlgaaeankivadtdhhqtgGXYLRINQFGD-VCTVDAS-vAKFArAKRTWKSghyfyeplvsggnllgvWVLPEE----YRKIGFFWExesgrCFRIERRAFPVgpYTFXRQATEVG--------------------------------GKISFVFYVKVSND-----pesDPIPlqsrdytalagrdnaptnlgkpyptlakdldypkkrd

d2gc9a1 xtktfktlDDFLGT-HFIYTYDN------------GWEYEWYakndhTVDYRIHG------gxvAGRWVTD---------------------qkADIVXLtegIYKISWTEPT--------GTDVALDFXpnEKKLHGTIFFPKwveehpeitvtyqnehidlxeqsrekYATYpKLVVPEFANITyxgdagqnnedviseapykexpndirngkyfdqnyhrlnk

d2a13a1 --ppvhpfVAPLSYlLGTWRGQGegeyptipsfryGEEIRFShsgkpVIAYTQKTwklesgapxHAESGYFrprpdgsievviaqstglvevqkGTYNVD---EQSIKLKSDLvgnaskvkEISREFELV--DGKLSYVVRXST--------------------------TTNPlQPHLKAILDKL----------------------------------------

d2gc9a1 xtktfktlDDFLGT-HFIYTYDN------------GWEYEWYakndhTVDYRIHGG------xvAGRWVTDQ---------------------kADIVXlTEGIYKISWTEP--------------tGTDVALDFXPneKKLHGTIFFPKwveehpeitvtyqnehidlxeqsrekYATYpKLVVPEFANITYxgdagqnnedviseapykexpndirngkyfdqnyhrlnk

d2fr2a1 ---dlapaLQALSPlLGSWAGRGagkyptirpfeyLEEVVFAhvgkpFLTYTQQTRavadgkplHSETGYLRvcrpgcvelvlahpsgiteievGTYSV-TGDVIELELSTRadgsiglaptakevtALDRSYRIDG--DELSYSLQMRA--------------------------VGQPlQDHLAAVLHRQR---------------------------------------

d2gc9a1 xtktFKTLddFLGT-HFIYTYDN-------------GWEYEWYakndhtVDYRIHGGXvAGRWVTD----------------QKADIVXLtegiYKISWTE------ptGTDVALDFXPnEKKLHGTIFFPKWveehpeitvtyqnehidlxeqsrekyATYPKLVVPEFANITyxgdagqnnedviseapykexpndirngkyfdqnyhrlnk

d2o62a1 ----ERPL-lQINDlLGEWRGQAvtiyrdlrppdiySTTLKIQlddagrLXQSTSFGE-RTITSTAtikgsivlfdqdpekqVQVLLLPD----GASATSPlkvqlrqpLFLEAGWLIQ-SDLRQRXIRSYND--------------------------KGEWVSLTLVTEERV----------------------------------------

d2ofmx1 ---actKNAIAQTGFNKDKYFNgDVWYVTdyldlepddvpkrycaalaagtasgklkeaLYHYdpktqdtFYDVSEL-QVESlGKYTANFKkvdkngnvkVAVTagNYYTFTVMYAD-DSSALIHTCLHKGNKDLGDLYAVLNRNKDaaagdkvksavsaatlefskfistkennCAYDNDSLKslltk

d1avgi_ aegddcSIEKAMGDFKPEEFFN-GTWYLA-----hgpgvtspavcqkfttsgskgftqiVEIGynkfesnVKFQCNQvDNKNgEQYSFKCK--------sSDNT-eFEADFTFISVSyDNFALVCRSITFTSQPKEDRYLVFERTKS---------------------------dTDPDAKEIC-----

d2ofmx1 actknaiaqtgfnkdkyfngdVWYVTDYLDLE------------------PDDVPkRYCAALAAGTasgkLKEALYHYDPktQDTFYDVSE------------------LQVESLGKYTANfkkvdkngnvkvavtaGNYYTFTVMYaddssALIHTCLHKGNKDlGDLYAVLNrnkdaaagdkvksavsaatlefskfistkenncaydndslkslltk

d1cbia_ ------------------pnfAGTWKMRSSENfdellkalgvnamlrkvaVAAAS-KPHVEIRQDG----DQFYIKTSTT--VRTTEINFKvgegfeeetvdgrkcrslPTWENENKIHCT-------qtllegdgpKTYWTRELAN-----DELILTFGADDVV-CTRIYVRE----------------------------------------------

d2ofmx1 actknaiaqtgfnKDKYfNGDVWYVTDYLD--LEPD---------dvPKRYCAALAAGTAS-GKLKEALYHYdpktqdTFYDVSE------------------lQVESL----GKYTanfkkvdkngnvkvavtAGNYYTFTVmYADDSSALIHTCLhkgnkDLGDLYAVLNRNkdaaagdkvksavsaatlefskfistkenncaydndslkslltk

d1ftpa_ -------------VKEF-AGIKYKLDSQTNfeEYMKaigvgaierkaGLALSPVIELEILDgDKFKLTSKTA------IKNTEFTfklgeefdeetldgrkvksTITQDgpnkLVHE---------------qkGDHPTIIIR-EFSKEQCVITIKL-----GDLVATRIYKAQ--------------------------------------------

d2ofmx1 actknaiaqtgfnKDKYfnGDVWYVTDYLDLEP-----------ddvPKRYCAALAAGTASGKLKEALYHYdpktqdTFYDVSE-------------------lQVESL----GKYTanfkkvdkngnvkvavtAGNYYTFTVmYADDSSALIHTCLHkgnkdlGDLYAVLNRNkdaaagdkvksavsaatlefskfistkenncaydndslkslltk

d1ggla_ -------------PPNL--TGYYRFVSQKNMEDylqalnislavrkiALLLKPDKEIEHQGNHMTVRTLST------FRNYTVQfdvgvefeedlrsvdgrkcqTIVTWeeehLVCV--------------qkgEVPNRGWRH-WLEGEMLYLELTAR-----dAVCEQVFRKV-------------------------------------------h

d2ofmx1 actknaiaqtgfnkDKYFngDVWYVTDYL-----------dlepddvpkryCAALAAGTASGKLKEALYHYdpktqdTFYDVSEL--------------------QVESL---GKYTanfkkvdkngnvkvavtagnyyTFTVmYADDSSALIHTCLhkgnkDLGDLYAVLNRNkdaaagdkvksavsaatlefskfistkenncaydndslkslltk

d1kqxa_ -------------pADFN--GTWEMLSNDnfedvmkaldidfatrkiavhlKQTKVIVQNGDKFETKTLST------FRNYEVNFvigeefdeqtkgldnrtvktLVKWDgdkLVCV--------------qkgekenrGWKQ-WIEGDLLHLEIHC-----QDKVCHQVFKKK-------------------------------------------n

d2ofmx1 actknaiaqtgfnKDKYFngDVWYVTDYLD--LEPD---------dvPKRYCAALAAGTASGKLKEALYHYdpktqdTFYDVSE-----------------lQVESL----GKYTanfkkvdkngnvkvavTAGNyYTFTVmYADDSSALIHTCLhkgnkDLGDLYAVLNRNkdaaagdkvksavsaatlefskfistkenncaydndslkslltk

d1liba_ -------------CDAFV--GTWKLVSSENfdDYMKevgvgfatrkvAGMAKPNMIISVNGDLVTIRSEST------FKNTEISfklgvefdeitaddrkvkSIITLdggaLVQV-------------qkwDGKS-TTIKR-KRDGDKLVVECVM-----KGVTSTRVYERA--------------------------------------------

d2ofmx1 actknaiaqtgfnkdkyfngdVWYVTDYLDLEPD--------------dvPKRYC-AALAAGTAsgklkEALYHYDPktQDTFYD--------------------vSELQVESlGKYTANFkkvdkngnvkvavTAGNYYTFTVMYaddssALIHTCLHKGNKDLgDLYAVLNrnkdaaagdkvksavsaatlefskfistkenncaydndslkslltk

d1lpja_ -----------------padlSGTWTLLSSDNFEgymlalgidfatrkiaKLLKPqKVIEQNGD----sFTIHTNSS--LRNYFVkfkvgeefdednrgldnrkckSLVIWDN-DRLTCIQ----------kgeKKNRGWTHWIEG-----DKLHLEMFCEGQVC-KQTFQRA----------------------------------------------

d2ofmx1 ------------actknaiaqTGFNKDKYFngDVWYVTD-------------------------yldlepddvpkrYCAALAAGTasgkLKEALYHYdpktqdTFYDVSELQVESL-------------GKYTANfkkvdkngnvkvavtagnYYTFTVMY-----aDDSSALIHTCLH--kgnkdlgDLYAVLNRNKDA--AAGDkvksavsaatlefskfistkenncaydndslkslltk

d1oeja_ gkplteveqkaangvfddanvQNRTLSDWD--GVWQSVYpllqsgkldpvfqkkadadktktfaeikdyyhkgyatDIEMIGIED----GIVEFHRN------NETTSCKYDYDGYkiltyksgkkgvrYLFECK------------dpeskaPKYIQFSDhiiaprKSSHFHIFMGNDsqqsllnemENWPTYYPYQLSseEVVE--------------------------------emmsh

d2ofmx1 actknaiaqtgfnkdkyfngdVWYVTDYLDLEPD---------------dVPKRYCAALAAGTasgkLKEALYHYDPktQDTFYDVSEL--------------------QVESlGKYTANfkkvdkngnvkvavtaGNYYTFTVMYaddssALIHTCLHKGNKDlGDLYAVLNrnkdaaagdkvksavsaatlefskfistkenncaydndslkslltk

d1opaa_ -----------------tkdqNGTWEMESNENFEgymkaldidfatrkiaVRLTQTKIIVQDG----DNFKTKTNST--FRNYDLDFTVgvefdehtkgldgrnvktlvTWEG-NTLVCV----------qkgekeNRGWKQWVEG-----DKLYLELTCGDQV-CRQVFKKK----------------------------------------------

d2ofmx1 actknaiaqtgfnkdKYFNgdVWYVTDYLD--LEPDD---------vPKRYCAALAAGTASGKLKEALYHYdpktqdTFYDVSEL-----------------QVESL----GKYTanfkkvdkngnvkvavtagnyYTFTVmYADDSSALIHTCLhkgnkDLGDLYAVLNRNkdaaagdkvksavsaatlefskfistkenncaydndslkslltk

d1p6pa_ ---------------AFNG--TWNVYAQENyeNFLRTvglpediikvAKDVNPVIEIEQNGNEFVVTSKTP------KQTHSNSFtvgkeseitsmdgkkikVTVQLeggkLICK------------------sdkFSHIQ-EVNGDEMVEKITI-----GSSTLTRKSKRV--------------------------------------------

d2ofmx1 actknaiaqtgfnkdkyfNGDVWYVtDYLDLE-----pDDVPkrYCAALAAGTASGKLKEALYHYdpkTQDTFYDVSELQVeslgkYTANFKKVDkngnvKVAVtagNYYT------------------------FTVMYaddSSALIHTCLhkgnkdlGDLYAVLNRNKdaaagdkvksavsaatlefskfistkenncaydndslkslltk

d1r0ua_ ----------gfqsnamkQETPITL-HVKSVIeddgnqEVIE--FRTTGFYYVKQNKVYLSYYEE--hDLGKVKTIVKVSE-----GEVLVMRSG----aVKMN---QRFVtgastiakykmsfgelelktstksIQSDL-deEKGRISIAYdmhvghlHNMTITYEGGT-------------------------------------------

d2ofmx1 actknaiaqtgfnkdKYFNgdVWYVTDYLD--LEPDD---------vPKRYCAALAAGTASGKLKEALYHYdpktqdTFYDVSEL-----------------QVESL----GKYTanfkkvdkngnvkvavtagnyYTFTVmYADDSSALIHTCLhkgnkDLGDLYAVLNRNkdaaagdkvksavsaatlefskfistkenncaydndslkslltk

d1tvqa_ ---------------AFSG--TWQVYAQENyeEFLKAlalpedlikmARDIKPIVEIQQKGDDFVVTSKTP------RQTVTNSFtlgkeadittmdgkklkCTVHLangkLVTK------------------sekFSHEQ-EVKGNEMVETITF-----GGVTLIRRSKRV--------------------------------------------

d2ofmx1 ---------------------------------------------------------------------------------------------------aCTKN-----------------------------------------aiaqtgfnKDKYfngdVWYVTDYLDlepddVPKR------------------------------YCAALAAGTasgkLKEALYHYDPktQDTFYDVSE------------------LQVESLGKYTANfkkvdkngnvkvavtAGNYYTFTVMYaddsSALIHTCLHKGNKDLgDLYAVLNRNkdaaagdkvksavsaatlefskfistkenncaydNDSLKS---------------------------------lltk

d1vpra1 ekgfeagdnklggalnakhvekygdnfkngxhkpefhedglhkpxevggkkfesgfhyllechelggknasggyggplcedpygsevqaxtekllkeadsDRTLcfnnfqdpcpqltkeqvaxckgfdygdktlklpcgplpwpaglpepgyvPKTN--plHGRWITVSG-----GQAAfikeaiksgmlgaaeankivadtdhhqtggXYLRINQFG----DVCTVDASVA-kFARAKRTWKsghyfyeplvsggnllgvWVLPEEYRKIGF--------fwexesgRCFRIERRAFP-vgpYTFXRQATEVGGKIS-FVFYVKVSN--------------------------------dpESDPIPlqsrdytalagrdnaptnlgkpyptlakdldypkkrd

d2ofmx1 actknaiaqtgfnkdkyfngdVWYVTDYLDLEPD-----------------dvPKRYCAALAAGTAsgklkealyHYDPktQDTFYDV------------------SELQVESLGKYTANFKkvdkngnvkvavtaGNYYTFTVMYAddssALIHTCLHKGNKDlGDLYAVLNrnkdaaagdkvksavsaatlefskfistkenncaydndslkslltk

d1xcaa_ ------------------pnfSGNWKIIRSENFEellkvlgvnvmlrkiavaaASKPAVEIKQEGD----tfyikTSTT--VRTTEINfkvgeefeeqtvdgrpckSLVKWESENKMVCEQK-------llkgegpKTSWTMELTND----GELILTMTADDVV-CTRVYVRE----------------------------------------------

d2ofmx1 actknaiaqtgfNKDKYfnGDVWYVtDYLDLE--PDDVpkRYCAALAAGTA-SGKLKEALYHYDPKTQDT-FYDVSEL---------------------QVESL------GKYTanfkkvdkngnvkvAVTA-GNYYTFTVmYADDSSALIHTCLHKGNK-DLGDLYAVLNRNkdaaagdkvksavsaatlefskfistkenncaydndslkslltk

d2a13a1 ----ppvhpfvaPLSYL--LGTWRG-QGEGEYptIPSF--RYGEEIRFSHSgKPVIAYTQKTWKLESGAPxHAESGYFrprpdgsievviaqstglvevQKGTYnvdeqsIKLK----------sdlvGNASkVKEISREF-ELVDGKLSYVVRXSTTTNpLQPHLKAILDKL--------------------------------------------

d2ofmx1 actknaiaqtgfnkDKYFngDVWYVTDYLDlePDDV------------pKRYCAALAAGTASGKLKEALYHYdpktqdTFYDVSEL-----------------qVESL-----GKYTanfkkvdkngnvkvavtagnyYTFTVmYADDSSALIHTCLhkgnkDLGDLYAVLNRNkdaaagdkvksavsaatlefskfistkenncaydndslkslltk

d2f73a1 --------------MSFS--GKYQLQSQEN-fEAFMkaiglpeeliqkgKDIKGVSEIVQNGKHFKFTITAG------SKVIQNEFtvgeeceletmtgekvktVVQLegdnkLVTT------------------fknIKSVT-ELNGDIITNTMTL-----GDIVFKRISKRI--------------------------------------------

d2ofmx1 actknaiaqtgfNKDKYFngDVWYVtDYLDL--ePDDVpkRYCAALAAGTASG-KLKEALYHYDPKTQDT-FYDVSELQVESlgkyTANFKKVdkngnVKVAV----------------------TAGN---------yyTFTVmYADDSSALIHTCLH-kgNKDLGDLYAVLNRNkdaaagdkvksavsaatlefskfistkenncaydndslkslltk

d2fr2a1 -----dlapalqALSPLL--GSWAG-RGAGKyptIRPF--EYLEEVVFAHVGKpFLTYTQQTRAVADGKPlHSETGYLRVCRpgcvELVLAHP-----SGITEievgtysvtgdvielelstradGSIGlaptakevtalDRSY-RIDGDELSYSLQMRavgQPLQDHLAAVLHRQ-------------------------------------------r

d2ofmx1 actknaiaqTGFNKDKYFnGDVWYVtDYLDlepddvpkrYCAALAAGTasgKLKEALYHYDpktqdTFYDV-SELQVESL----GKYTANFkkvdkngnvkvavTAGNYYTFTVMYAddssALIHTCLHKG--------------------------------NKDLgDLYAVLNRnkdaaagdkvksavsaatlefskfistkenNCAY-------dndslkslltk

d2gc9a1 ------xtkTFKTLDDFL-GTHFIY-TYDN--------gWEYEWYAKN---DHTVDYRIHG-gxvaGRWVTdQKADIVXLtegiYKISWTE-------------PTGTDVALDFXPN---eKKLHGTIFFPkwveehpeitvtyqnehidlxeqsrekyatypKLVV-PEFANITY-------------xgdagqnnedviseapyKEXPndirngkyfdqnyhrlnk

d2ofmx1 actknaiaqTGFNKDKYFngDVWYVtDYLDLE---pDDVPkrYCAALAAGTAS-GKLKEALYHYdpktqdTFYDVSE----------------lQVESL-----GKYTanfkkvdkngnvkvavTAGNYYTFTVMYADDSSALIHTCLHKG-NKDLGDLYAVLNRNkdaaagdkvksavsaatlefskfistkenncaydndslkslltk

d2o62a1 -------erPLLQINDLL--GEWRG-QAVTIYrdlrPPDI--YSTTLKIQLDDaGRLXQSTSFG------ERTITSTatikgsivlfdqdpekqVQVLLlpdgaSATS-----------plkvqLRQPLFLEAGWLIQSDLRQRXIRSYNDkGEWVSLTLVTEERV--------------------------------------------

d2ofmx1 actknaIAQT-GFNKDKYFngDVWYVTDYLDLEPDDVPKR---YCAALAAGTAsGKLKEALYHYDPKTQDTFYDVSeLQVE-SLGKYTANFkkvdkngnvkvavtaGNYYTFTVMYAD-DSSALIHTCLHKGNKDlGDLYAVLNRNKDAAaGDKVKSAVSAATL---EFSKFISTKENNCAydndslkslltk

d2ozqa1 ---eeaSSTGrNFNVEKIN--GEWHTIILASDKREKIEDNgnfRLFLEQIHVL-EKSLVLKFHTVRDEECSELSMV-ADKTeKAGEYSVTY---------------DGFNTFTIPKTDyDNFLMAHLINEKDGET-FQLMGLYGREPDLS-SDIKERFAQLCEEhgiLRENIIDLSNANRC------------

d2ofmx1 ACTKNAIAQTGFNKDKYFNGDVWYVTDYLDLEPDdvPKRYCAALAAGTASGKLKEALYHYDPKTQDTFYDVSELQVESLGKYTANFKKVDKNGNVKVAVTAGNYYTFTVMYADDSSALIHTCLHKGNKDLGDLYAVLNRNKDAAAGDKVKSAVSAATLEFSKFISTKENNCAYDNDSLKSLLTK

d3np1a_ KCTKNALAQTGFNKDKYFNGDVWYVTDYLDLEPDdvPKRYCAALAAGTASGKLKEALYHYDPKTQDTFYDVSELQEESPGKYTANFKKVEKNGNVKVDVTSGNYYTFTVMYADDSSALIHTCLHKGNKDLGDLYAVLNRNKDTNAGDKVKGAVTAASLKFSDFISTKDNKCEYDNVSLKSLLTK

d2ozqa1 ------eeasSTGRnFNVEKI-NGEWHTII-----------------------lasdkREKIEDNGnfrlfleqihvlekslvlkfhtvrdeecselsmvadkTEKAgEYSVTY------DGFNTFTIPKTDYDNFLMAHLINEK-DGETFQLMGLYGREPdlssdikerfaqlceehgilreniidlSNANRC-----

d1avgi_ aegddcsiekAMGD-FKPEEFfNGTWYLAHgpgvtspavcqkfttsgskgftqiveigYNKFESNV----------------------------kfqcnqvdnKNGE-QYSFKCkssdntEFEADFTFISVSYDNFALVCRSITFtSQPKEDRYLVFERTK---------------------------SDTDPDakeic

d2ozqa1 eeasstgrnfnvekinGEWHTiILASDKRE--------------kiEDNGnFRLFLeQIHVLEKSLVLKFHTVrdeeCSELSMV-------------------aDKTEKAGEYSVT--------ydGFNTFTIPKTdydnflMAHLINEKDGETFQLMGLYGrepdlssdikerfaqlceehgilreniidlsnanrc

d1cbia_ -------------pnfAGTWK-MRSSENFDellkalgvnamlrkvaVAAA-SKPHV-EIRQDGDQFYIKTSTT----VRTTEINfkvgegfeeetvdgrkcrslPTWENENKIHCTqtllegdgpkTYWTRELAND------ELILTFGADDVVCTRIYVRE------------------------------------

d2ozqa1 eeasstgrnfnVEKIN-GEWHTIILAS--DKRE------kiEDNGNfrlFLEQIHVLE---KSLVLKFHtvrdeeCSELSMV------------------adKTEKA----GEYSVtydGFNTFTIpKTDYdNFLMAHLINEkdgeTFQLMGLYGREpdlssdikerfaqlceehgilreniidlsnanrc

d1ftpa_ -----------VKEFAgIKYKLDSQTNfeEYMKaigvgaieRKAGL-alSPVIELEILdgdKFKLTSKT------AIKNTEFtfklgeefdeetldgrkvksTITQDgpnkLVHEQkgdHPTIIIR-EFSK-EQCVITIKLG----DLVATRIYKAQ----------------------------------

d2ozqa1 eeasstgrnfnVEKINGEWHTIILAS--DKRE------kiEDNGNfRLFLEQIHVLEKSLVLKFHTVrdeeCSELSMV--------------------aDKTEKA--GEYSV-tydGFNTFTIpKTDYdNFLMAHLINEkdgeTFQLMGLYGREpdlssdikerfaqlceehgilreniidlsnanrc

d1ggla_ -----------PPNLTGYYRFVSQKNmeDYLQalnislavRKIALlLKPDKEIEHQGNHMTVRTLST----FRNYTVQfdvgvefeedlrsvdgrkcqtIVTWEEehLVCVQkgevPNRGWRH-WLEG-EMLYLELTAR----DAVCEQVFRKV---------------------------------h

d2ozqa1 eeasstgrnfnVEKINGEWHTIILA--------sdkrekiedngnfrLFLEQIHVLEKSLVLKFHTVrdeeCSELSMVADK-------------------TEKA--GEYS--vtydgfntFTIPkTDYDnFLMAHLINEkdgeTFQLMGLYGREpdlssdikerfaqlceehgilreniidlsnanrc

d1kqxa_ -----------PADFNGTWEMLSNDnfedvmkaldidfatrkiavhlKQTKVIVQNGDKFETKTLST----FRNYEVNFVIgeefdeqtkgldnrtvktlVKWDgdKLVCvqkgekenrgWKQW-IEGD-LLHLEIHCQ----DKVCHQVFKKK---------------------------------n

d2ozqa1 eeasstgrnfnVEKINGEWHTIILA--------sdkrekiedngnfrLFLEQIHVLEKSLVLKFHTVrdeeCSELSMVAD---------------------ktekageysvtydgfntfTIPKTDydNFLMAHLINEkdgeTFQLMGLYGREpdlssdikerfaqlceehgilreniidlsnanrc

d1liba_ -----------CDAFVGTWKLVSSEnfddymkevgvgfatrkvagmaKPNMIISVNGDLVTIRSEST----FKNTEISFKlgvefdeitaddrkvksiitldggalvqvqkwdgksttiKRKRDG--DKLVVECVMK----GVTSTRVYERA----------------------------------

d2ozqa1 eeasstgrnfnvEKINGEWHTIILAS--DKRE------kiEDNGNfRLFLEQIHVLEKSLVLKFHTVrdeeCSELSMVADK-------------------TEKA---GEYS-vtydgfnTFTIpKTDYdNFLMAHLINEkdgeTFQLMGLYGREpdlssdikerfaqlceehgilreniidlsnanrc

d1lpja_ -----------pADLSGTWTLLSSDNfeGYMLalgidfatRKIAKlLKPQKVIEQNGDSFTIHTNSS----LRNYFVKFKVgeefdednrgldnrkckslVIWDndrLTCIqkgekknrGWTH-WIEG-DKLHLEMFCE----GQVCKQTFQRA----------------------------------

d2ozqa1 -------------eeasstgRNFNV-EKINGEWHTII----------------------lasdkrekiedngnFRLFLEQIHVleksLVLKFHTVrdeeCSELSMVADKTEKA------------GEYSVTY---dgfNTFTIPK----tdYDNFLMAHLINE---kdgetfqLMGLYGREPD---lSSDIKERfaqlceehgilreniidlsnanrc

d1oeja_ gkplteveqkaangvfddanVQNRTlSDWDGVWQSVYpllqsgkldpvfqkkadadktktfaeikdyyhkgyaTDIEMIGIED----GIVEFHRN----NETTSCKYDYDGYKiltyksgkkgvrYLFECKDpeskapKYIQFSDhiiaprKSSHFHIFMGNDsqqsllnemeNWPTYYPYQLsseeVVEEMMS-----------------------h

d2ozqa1 eeasstgrnfnvEKINGEWHTIILAS--DKRE------kiEDNGNFRlFLEQIHVLEKSLVLKFHTVrdeeCSELSMV--------------------adKTEKA--GEYS-vtydgFNTFTIpKTDYdNFLMAHLINEkdgeTFQLMGLYGREpdlssdikerfaqlceehgilreniidlsnanrc

d1opaa_ -----------tKDQNGTWEMESNENfeGYMKaldidfatRKIAVRLtQTKIIVQDGDNFKTKTNST----FRNYDLDftvgvefdehtkgldgrnvktlVTWEGntLVCVqkgekeNRGWKQ-WVEG-DKLYLELTCG----DQVCRQVFKKK----------------------------------

d2ozqa1 eeasstgrnfnveKINGeWHTIILASDKRE------------kiEDNGnfRLFLeQIHVLEKSLVLKFHTVrdeeCSELSMV------------------adKTEKA-GEYSVTydGFNTFTIPKTdydnFLMAHLINEKdgETFQLMGLYGRepdlssdikerfaqlceehgilreniidlsnanrc

d1p6pa_ -------------AFNG-TWNVYAQENYENflrtvglpediikvAKDV--NPVI-EIEQNGNEFVVTSKTP----KQTHSNSftvgkeseitsmdgkkikvtVQLEGgKLICKS-dKFSHIQEVNG----DEMVEKITIG--SSTLTRKSKRV-----------------------------------

d2ozqa1 eeasstgrnfnvekINGEWHTiILASDK------REKIedngnfRLFLEQIHVLEKSLVLKFHTVRDEECSELSMVADktekaGEYSV------------------------------tyDGFN-tFTIPktdYDNFLMAHLINEKDG-ETFQLMGLYGREPdlssdikerfaqlceehgilreniidlsnanrc

d1r0ua_ ------gfqsnamkQETPITL-HVKSVIeddgnqEVIE------FRTTGFYYVKQNKVYLSYYEEHDLGKVKTIVKVS-----EGEVLvmrsgavkmnqrfvtgastiakykmsfgelelKTSTksIQSD--lDEEKGRISIAYDMHVgHLHNMTITYEGGT---------------------------------

d2ozqa1 eeasstgrnfnveKINGEWHTIILAS--DKRE------kiEDNGNfRLFLEQIHVLEKSLVLKFHTVrdeeCSELSMVA------------------dKTEKA--GEYSvtydgFNTFTIpKTDYdNFLMAHLINEkdgeTFQLMGLYGREpdlssdikerfaqlceehgilreniidlsnanrc

d1tvqa_ -------------AFSGTWQVYAQENyeEFLKalalpedlIKMARdIKPIVEIQQKGDDFVVTSKTP----RQTVTNSFtlgkeadittmdgkklkctVHLANgkLVTK---seKFSHEQ-EVKG-NEMVETITFG----GVTLIRRSKRV----------------------------------

d2ozqa1 -------------------------------------------------------------------------------------------------------------------------------------------------eeasstgrnFNVEkinGEWHTiILASdkrekIEDN-------------------------gnFRLFLeQIHVLEKSLVLKFHTVrdeecSELSMV--------------------ADKTeKAGEYSVTY-------dGFNTFTIPKTDydnFLMAHLINEKDGETFQLMGLYGR-----------epdlssdikerfaqlceehgilreniidlsnanrc

d1vpra1 ekgfeagdnklggalnakhvekygdnfkngxhkpefhedglhkpxevggkkfesgfhyllechelggknasggyggplcedpygsevqaxtekllkeadsdrtlcfnnfqdpcpqltkeqvaxckgfdygdktlklpcgplpwpaglpepgyvpKTNP--lHGRWI-TVSG----gQAAFikeaiksgmlgaaeankivadtdhhqtGGXYL-RINQFGDVCTVDASVA---kfARAKRTwksghyfyeplvsggnllgvWVLP-EEYRKIGFFwexesgrcFRIERRAFPVG--pYTFXRQATEVGGKISFVFYVKVSndpesdpiplqsrdytalagrdnaptnlgkpyptlakdldypkkrd

d2ozqa1 eeasstgrnfnvEKINGEWHTIILAS--DKRE-------kiEDNG-nfRLFLEQIHVLEKSLVLKFHTVrdeeCSELSMV------------------adKTEKA--GEYSVT------ydGFNTFTIpkTDYDNFLMAHLINEkdgeTFQLMGLYGREpdlssdikerfaqlceehgilreniidlsnanrc

d1xcaa_ ------------PNFSGNWKIIRSENfeELLKvlgvnvmlrKIAVaaaSKPAVEIKQEGDTFYIKTSTT----VRTTEINfkvgeefeeqtvdgrpckslVKWESenKMVCEQkllkgegpKTSWTME--LTNDGELILTMTAD----DVVCTRVYVRE----------------------------------

d2ozqa1 eeasstgrnfNVEKINGEWHtiilasdkrekIEDN------gnfrLFLEqIHVL-EKSLVLKFHTVR---DEECSELSMVADKTeKAGEYSVTY------DGFNTFTIPKTdydnfLMAHLIN-EKDG----ETFQLMGLYGrepdlssdikerfaqlceehgilreNIIDLSnanrc

d2a13a1 ---ppvhpfvAPLSYLLGTW--------rgqGEGEyptipsfrygEEIR-FSHSgKPVIAYTQKTWKlesGAPXHAESGYFRPR-PDGSIEVVIaqstglVEVQKGTYNVD----eQSIKLKSdLVGNaskvKEISREFELV-----dgklsyvvrxstttnplqphLKAILD---kl

d2ozqa1 eeasstgrnfnvekINGEWHTiiLASDKRE-----------kieDNGNfRLFLeQIHVLEKSLVLKFHTVrdeeCSELSMV-------------------adKTEKAGEYSVT-ydGFNTFTIPKtdydnfLMAHLINEKDGETFQLMGLYGrepdlssdikerfaqlceehgilreniidlsnanrc

d2f73a1 -------------mSFSGKYQ-lQSQENFEafmkaiglpeeliqKGKDiKGVS-EIVQNGKHFKFTITAG----SKVIQNEftvgeeceletmtgekvktvvQLEGDNKLVTTfknIKSVTELNG------DIITNTMTLGDIVFKRISKRI------------------------------------

d2ozqa1 eeasstgrnFNVEKINGEWhtiilasdkrekIEDN-----gnfrlflEQIHVL-EKSLVLKFHTVR---DEECSELSMVADKTeKAGEYSVTY------DGFNTFTIpKTDYdNFLMAHLIN----EKDG-------ETFQLMGLYGREPdlssdikerfaqlceehgilreniIDLSnanrc

d2fr2a1 ---dlapalQALSPLLGSW---------agrGAGKyptirpfeyleeVVFAHVgKPFLTYTQQTRAvadGKPLHSETGYLRVC-RPGCVELVLahpsgiTEIEVGTY-SVTG-DVIELELSTradgSIGLaptakevTALDRSYRIDGDE------lsyslqmravgqplqdhlAAVL-hrqr

d2ozqa1 eeasstgrNFNVEkiNGEWHTIILasdkrekiedngnfRLFLEqIHVLE-KSLVLKFHTvrdeecSELSMV-ADKTE-------kaGEYSVTYdgFNTFTIpKTDY-DNFLMAHLINEK----------------------------DGETFQLMGLYGREpdlssdikerfaqlceehgilreniidlsnANRC-----------------

d2gc9a1 -xtktfktLDDFL-gTHFIYTYDN--------------GWEYE-WYAKNdHTVDYRIHG---gxvAGRWVTdQKADIvxltegiykISWTEPT--GTDVAL-DFXPnEKKLHGTIFFPKwveehpeitvtyqnehidlxeqsrekyaTYPKLVVPEFANIT-----------yxgdagqnnedviseapykEXPNdirngkyfdqnyhrlnk

d2ozqa1 eeasstgRNFNVEKINGEWHTiILASDK----REKIedngnfRLFLEQIHVL-eksLVLKFHTvrdeeCSELSMV----------------aDKTE----kaGEYS----vtydGFNTFTIpKTDYDNFLMAHLINEK-DGET-FQLMGLYGREpdlssdikerfaqlceehgilreniidlsnanrc

d2o62a1 -----erPLLQINDLLGEWRG-QAVTIYrdlrPPDI------YSTTLKIQLDdagrLXQSTSF----gERTITSTatikgsivlfdqdpekqVQVLllpdgaSATSplkvqlrqPLFLEAG-WLIQSDLRQRXIRSYNdKGEWvSLTLVTEERV----------------------------------

d2ozqa1 ---eeaSSTGrNFNVEKI--NGEWHTIILASDKREkiEDNGnfRLFLeQIHVLE--KSLVLKFHTVRDEECSELSMV-ADKTeKAGEYSVTY---------------dGFNTFTIPKTDyDNFLMAHLINEKDGET-FQLMGLYGREPDLSsdIKERFAQLCEEHGILRENIIDLSNANRC------------

d3np1a_ kctknaLAQT-GFNKDKYfnGDVWYVTDYLDLEPD-dVPKR--YCAA-LAAGTAsgKLKEALYHYDPKTQDTFYDVSeLQEE-SPGKYTANFkkvekngnvkvdvtsgNYYTFTVMYAD-DSSALIHTCLHKGNKDlGDLYAVLNRNKDTN--AGDKVKGAVTAASLKFSDFISTKDNKCEydnvslkslltk

d3np1a_ --KCTK-NALAQTGFNKDKYFNgDVWYVTDyldlepddvpKRYC--aalaagTASGK-lkealyhydpktqDTFYDVSELQ--EESPgKYTANFKKVekngnvKVDVtsgNYYTFTVMYAD-DSSALIHTCLHKGNKDLGDLYAVLNRNKDtnagdkvkgavtaaslkfsdfistkdnKCEYDNVSLkslltk

d1avgi_ aeGDDCsIEKAMGDFKPEEFFN-GTWYLAH--------gpGVTSpavcqkftTSGSKgftqiveigynkfeSNVKFQCNQVdnKNGE-QYSFKCKSS------DNTE---FEADFTFISVSyDNFALVCRSITFTSQPKEDRYLVFERTKS--------------------------dTDPDAKEIC------

d3np1a_ kctknalaqtgfnkdkyfngdVWYVTDYLDL-----------------epddvpkRYCAALAAGTasgkLKEALYHYDPktQDTFYDV------------------sELQEESPGKYTANfkkvekngnvkvdvtSGNYYTFTVMYaddssALIHTCLHKGNKDlGDLYAVLNrnkdtnagdkvkgavtaaslkfsdfistkdnkceydnvslkslltk

d1cbia_ ------------------pnfAGTWKMRSSEnfdellkalgvnamlrkvavaaasKPHVEIRQDG----DQFYIKTSTT--VRTTEINfkvgegfeeetvdgrkcrsLPTWENENKIHCT-------qtllegdgPKTYWTRELAN-----DELILTFGADDVV-CTRIYVRE----------------------------------------------

d3np1a_ kctknalaqtgfnKDKYfNGDVWYVTDYLD--LEPD-----DVPK----RYCAALAAGTAS-GKLKEALYHYdpktqdTFYDVSEL------------------QEESP----GKYTanfkkvekngnvkvdvtSGNYYTFTVmYADDSSALIHTCLhkgnkDLGDLYAVLNRNkdtnagdkvkgavtaaslkfsdfistkdnkceydnvslkslltk

d1ftpa_ -------------VKEF-AGIKYKLDSQTNfeEYMKaigvgAIERkaglALSPVIELEILDgDKFKLTSKTA------IKNTEFTFklgeefdeetldgrkvksTITQDgpnkLVHE---------------qkGDHPTIIIR-EFSKEQCVITIKL-----GDLVATRIYKAQ--------------------------------------------

d3np1a_ kctknalaqtgfnKDKYfnGDVWYVTDYLDLEP-------dDVPK----RYCAALAAGTASGKLKEALYHYdpktqdTFYDVSEL-------------------qEESP----GKYTanfkkvekngnvkvdvtsgnyYTFTVmYADDSSALIHTCLhkgnkDLGDLYAVLNRNkdtnagdkvkgavtaaslkfsdfistkdnkceydnvslkslltk

d1ggla_ -------------PPNL--TGYYRFVSQKNMEDylqalnisLAVRkialLLKPDKEIEHQGNHMTVRTLST------FRNYTVQFdvgvefeedlrsvdgrkcqtIVTWeeehLVCV--------------qkgevpnRGWRH-WLEGEMLYLELTA-----RDAVCEQVFRKV-------------------------------------------h

d3np1a_ kctknalaqtgfnKDKYfnGDVWYVTDYLD--LEPD-----DVPK----RYCAALAAGTASGKLKEALYHYdpktqdTFYDVSEL--------------------QEESP---GKYTanfkkvekngnvkvdvtsgnyYTFTVmYADDSSALIHTCLhkgnkDLGDLYAVLNRNkdtnagdkvkgavtaaslkfsdfistkdnkceydnvslkslltk

d1kqxa_ -------------PADF--NGTWEMLSNDNfeDVMKaldidFATRkiavHLKQTKVIVQNGDKFETKTLST------FRNYEVNFvigeefdeqtkgldnrtvktLVKWDgdkLVCV--------------qkgekenRGWKQ-WIEGDLLHLEIHC-----QDKVCHQVFKKK-------------------------------------------n

d3np1a_ kctknalaqtgfnKDKYFngDVWYVTDYLD--LEPD----DVPK-----RYCAALAAGTASGKLKEALYHYdpktqdTFYDVSE------------------lQEESP---GKYTAnfkkvekngnvkvdvtSGNYYTFTVmYADDSSALIHTCLhkgnkDLGDLYAVLNRNkdtnagdkvkgavtaaslkfsdfistkdnkceydnvslkslltk

d1liba_ -------------CDAFV--GTWKLVSSENfdDYMKevgvGFATrkvagMAKPNMIISVNGDLVTIRSEST------FKNTEISfklgvefdeitaddrkvksIITLDggaLVQVQ--------------kwDGKSTTIKR-KRDGDKLVVECVM-----KGVTSTRVYERA--------------------------------------------

d3np1a_ kctknalaqtgfnkdkyfngdVWYVTDYLDLEPD------------DVPK---RYCAALAAGTasgkLKEALYHYDPktQDTFYDVSE--------------------LQEESpGKYTANfkkvekngnvkvdvTSGNYYTFTVMYaddssALIHTCLHKgNKDLGDLYAVLNrnkdtnagdkvkgavtaaslkfsdfistkdnkceydnvslkslltk

d1lpja_ -----------------padlSGTWTLLSSDNFEgymlalgidfatRKIAkllKPQKVIEQNG----DSFTIHTNSS--LRNYFVKFKvgeefdednrgldnrkckslVIWDN-DRLTCI----------qkgeKKNRGWTHWIEG-----DKLHLEMFC-EGQVCKQTFQRA----------------------------------------------

d3np1a_ ------------kctknalaqTGFNKDKYFngDVWYVTD-------------------------yldlepddvpkrycAALAAGTasgkLKEALYHYdpktqdTFYDVSELQEESP-------------GKYTANfkkvekngnvkvdvtsgnYYTFTVMY-----adDSSALIHTCLH--kgnkdlgDLYAVLNRNKDTN--AGDKvkgavtaaslkfsdfistkdnkceydnvslkslltk

d1oeja_ gkplteveqkaangvfddanvQNRTLSDWD--GVWQSVYpllqsgkldpvfqkkadadktktfaeikdyyhkgyatdiEMIGIED----GIVEFHRN------NETTSCKYDYDGYkiltyksgkkgvrYLFECK------------dpeskaPKYIQFSDhiiaprkSSHFHIFMGNDsqqsllnemENWPTYYPYQLSSeeVVEE--------------------------------mmsh

d3np1a_ kctknalaqtgfnKDKYfnGDVWYVTDYLDLEP------DDVP---KRYC--AALAAGTASGKLKEALYHYdpktqdTFYDVSEL--------------------QEESP---GKYTanfkkvekngnvkvdvTSGN-yyTFTVmYADDSSALIHTCLhkgnkDLGDLYAVLNRNkdtnagdkvkgavtaaslkfsdfistkdnkceydnvslkslltk

d1opaa_ -------------TKDQ--NGTWEMESNENFEGymkaldIDFAtrkIAVRltQTKIIVQDGDNFKTKTNST------FRNYDLDFtvgvefdehtkgldgrnvktLVTWEgntLVCV---------------qKGEKenrGWKQ-WVEGDKLYLELTC-----GDQVCRQVFKKK--------------------------------------------

d3np1a_ kctknalaqtgfnkdKYFNgdVWYVTDYLD--LEPD-------DVPK--RYCAALAAGTASGKLKEALYHYdpktqdTFYDVSELQ-----------------EESP----GKYTanfkkvekngnvkvdvtsgnyYTFTVmYADDSSALIHTCLhkgnkDLGDLYAVLNRNkdtnagdkvkgavtaaslkfsdfistkdnkceydnvslkslltk

d1p6pa_ ---------------AFNG--TWNVYAQENyeNFLRtvglpedIIKVakDVNPVIEIEQNGNEFVVTSKTP------KQTHSNSFTvgkeseitsmdgkkikvTVQLeggkLICK------------------sdkFSHIQ-EVNGDEMVEKITI-----GSSTLTRKSKRV--------------------------------------------

d3np1a_ kctknalaqtgfnkdkyfngdvwyvtdyldlepddvpkrycaalaagtasgKLKEALYHYDPK--TQDTFYDVSELQEESP---GKYTANFkkvekngnvkvdvtSGNYYTFTVMYaddssALIHTCLHKGnkDLGDLYAVlnrnkdtnagdkvkgavtaaslkfSDFI-----------------------------------STKDnKCEYdnvslkslltk

d1r0ua_ ----------------------------------------gfqsnamkqetPITLHVKSVIEDdgNQEVIEFRTTGFYYVKqnkVYLSYYE----------ehdlGKVKTIVKVSE-----GEVLVMRSGA--VKMNQRFV-----------------------tGASTiakykmsfgelelktstksiqsdldeekgrisiayDMHVgHLHN--mtityeggt

d3np1a_ kctknalaqtgfnkdKYFNgdVWYVTDYLD--LEPD-------dVPKR--YCAALAAGTASGKLKEALYHYdpktqdTFYDVSEL------------------QEESP---GKYTanfkkvekngnvkvdvtsgnyYTFTVmYADDSSALIHTCLhkgnkDLGDLYAVLNRNkdtnagdkvkgavtaaslkfsdfistkdnkceydnvslkslltk

d1tvqa_ ---------------AFSG--TWQVYAQENyeEFLKalalpedlIKMArdIKPIVEIQQKGDDFVVTSKTP------RQTVTNSFtlgkeadittmdgkklkcTVHLAngkLVTK------------------sekFSHEQ-EVKGNEMVETITF-----GGVTLIRRSKRV--------------------------------------------

d3np1a_ ---------------------------------------------------------------------------------------------------kCTKN-----------------------------------------alaqtgfnKDKYfngdVWYVTDYLD-lePDDVP------------------------krYCAALAAGTasgkLKEALYHYDPktqDTFYDVSE------------------LQEESPGKYTANfkkvekngnvkvdvtSGNYYTFTVMYaddsSALIHTCLHKGNKDlGDLYAVLNRNkdtnagdkvkgavtaaslkfsdfistkdnkceyDNVSlkSLLTK----------------------------------

d1vpra1 ekgfeagdnklggalnakhvekygdnfkngxhkpefhedglhkpxevggkkfesgfhyllechelggknasggyggplcedpygsevqaxtekllkeadsDRTLcfnnfqdpcpqltkeqvaxckgfdygdktlklpcgplpwpaglpepgyvPKTN--plHGRWITVSGgqaAFIKEaiksgmlgaaeankivadtdhhqtggXYLRINQFG----DVCTVDASVA-kfARAKRTWKsghyfyeplvsggnllgvWVLPEEYRKIGF--------fwexesgRCFRIERRAFP-vgpYTFXRQATEVGGKI-SFVFYVKVSN-------------------------------dpESDP--IPLQSrdytalagrdnaptnlgkpyptlakdldypkkrd

d3np1a_ kctknalaqtgfnkDKYFngDVWYVTDYLD--LEPD--------dVPKR---YCAALAAGTASGKLKEALYHYdpktqdTFYDVSELQEEspgkytanfkkvekngnvkvdvTSGNY-----------------------yTFTVmYADD-SSALIHTCLhkgnkDLGDLYAVLNRNkdtnagdkvkgavtaaslkfsdfistkdnkceydnvslkslltk

d1xcaa_ --------------PNFS--GNWKIIRSENfeELLKvlgvnvmlrKIAVaaaSKPAVEIKQEGDTFYIKTSTT------VRTTEINFKVG------------eefeeqtvdgRPCKSlvkwesenkmvceqkllkgegpktSWTM-ELTNdGELILTMTA-----DDVVCTRVYVRE--------------------------------------------

d3np1a_ kctknalaqtgfnKDKYFngDVWYVTdYLDLEPDDVpkrYCAALAAGTASG-KLKEALYHYDPKTQDT-FYDVSELQEespgkytANFKKVekngnvKVDV------------------TSGN------yytFTVMyADDSSALIHTC-lhkgnkDLGDLYAVLNRNkdtnagdkvkgavtaaslkfsdfistkdnkceydnvslkslltk

d2a13a1 ----ppvhpfvapLSYLL--GTWRGQ-GEGEYPTIPsfrYGEEIRFSHSGKpVIAYTQKTWKLESGAPxHAESGYFRPrpdgsieVVIAQS------TGLVevqkgtynvdeqsiklksDLVGnaskvkeisREFE-LVDGKLSYVVRxstttnpLQPHLKAILDKL--------------------------------------------

d3np1a_ kctknalaqtgfnkDKYFngDVWYVTDYLD--LEPD----DVPK-----RYCAALAAGTASGKLKEALYHYdpktqdTFYDVSEL------------------QEESP----GKYTanfkkvekngnvkvdvtsgnyYTFTVmYADDSSALIHTCLhkgnkDLGDLYAVLNRNkdtnagdkvkgavtaaslkfsdfistkdnkceydnvslkslltk

d2f73a1 --------------MSFS--GKYQLQSQENfeAFMKaiglPEELiqkgkDIKGVSEIVQNGKHFKFTITAG------SKVIQNEFtvgeeceletmtgekvktVVQLEgdnkLVTT------------------fknIKSVT-ELNGDIITNTMTL-----GDIVFKRISKRI--------------------------------------------

d3np1a_ kctknalaqtgfnKDKYF-NGDVwyvtdyldlepdDVPKRYCAALAaGTASGKLKEALYHYDP---KTQDTFYDVSeLQEESPGKYTANFKKVekngnvKVDVtsgNYYTFTVmYADDSSALIHTC----LHKG------nKDLGDLYAVLNRNKdtnagdkvkgavtaaslkfsdfISTKdnkceydnvslkslltk

d2fr2a1 --------dlapaLQALSpLLGSwagrgagkyptiRPFEYLEEVVF-AHVGKPFLTYTQQTRAvadGKPLHSETGY-LRVCRPGCVELVLAHP------SGIT---EIEVGTY-SVTGDVIELELStradGSIGlaptakeVTALDRSYRIDGDE---lsyslqmravgqplqdhlaAVLH--------------rqr

d3np1a_ kctknalaqtGFNKDKYFnGDVWYVtDYLDlepddvpkrYCAALAAGTasgKLKEALYHYdpktqdTFYDV-SELQEESP----GKYTANFkkvekngnvkvdvTSGNYYTFTVMYAddssALIHTCLHKGN-------------------------------KDLGDLYAVLNRNkdtnagdkvkgavtaaslkfsdfistkdnkceydnVSLKS----------------------lltk

d2gc9a1 ------xtktFKTLDDFL-GTHFIY-TYDN--------gWEYEWYAKN---DHTVDYRIH-ggxvaGRWVTdQKADIVXLtegiYKISWTE-------------PTGTDVALDFXPN---eKKLHGTIFFPKwveehpeitvtyqnehidlxeqsrekyatypKLVVPEFANITYX----------------------------gdagqnnEDVISeapykexpndirngkyfdqnyhrlnk

d3np1a_ kctknalaqTGFNKDKYFngDVWYVtDYLDLE-pDDVPkrYCAALAAGTAS-GKLKEALYHYdpktqdTFYDVSE----------------lQEESP-----GKYTanfkkvekngnvkvdvTSGNYYTFTVMYADDSSALIHTCLHKG-NKDLGDLYAVLNRNkdtnagdkvkgavtaaslkfsdfistkdnkceydnvslkslltk

d2o62a1 -------erPLLQINDLL--GEWRG-QAVTIYrdLRPPdiYSTTLKIQLDDaGRLXQSTSFG------ERTITSTatikgsivlfdqdpekqVQVLLlpdgaSATS-----------plkvqLRQPLFLEAGWLIQSDLRQRXIRSYNDkGEWVSLTLVTEERV--------------------------------------------
