# Supplementary material for: Structure-Based Phylogenetic Analysis of the Lipocalin Superfamily
Source: PLoS One. 2015 Aug 11;10(8):e0135507. doi: 10.1371/journal.pone.0135507 (PMC4532494; doi:10.1371/journal.pone.0135507)
Supplement: S2 Text — (DOC) [file pone.0135507.s003.doc]

**S2 Text:** Multiple sequence alignment for 39 lipocalin domains using clustalW2.

d1bj7a_ ------------------------------------------------------------

d1e5pa_ ------------------------------------------------------------

d1a3ya_ ------------------------------------------------------------

d1ew3a_ ------------------------------------------------------------

d1gm6a_ ------------------------------------------------------------

d2ozqa1 ------------------------------------------------------------

d1beba_ ------------------------------------------------------------

d1yupa1 ------------------------------------------------------------

d1exsa_ ------------------------------------------------------------

d1brqa_ ------------------------------------------------------------

d1jyda_ ------------------------------------------------------------

d1hbqa_ ------------------------------------------------------------

d2ofmx1 ------------------------------------------------------------

d3np1a_ ------------------------------------------------------------

d1euoa_ ------------------------------------------------------------

d2a13a1 ------------------------------------------------------------

d2fr2a1 ------------------------------------------------------------

d1cbia_ ------------------------------------------------------------

d1xcaa_ ------------------------------------------------------------

d1kqxa_ ------------------------------------------------------------

d1opaa_ ------------------------------------------------------------

d1lpja_ ------------------------------------------------------------

d1ggla_ ------------------------------------------------------------

d1ftpa_ ------------------------------------------------------------

d1liba_ ------------------------------------------------------------

d1p6pa_ ------------------------------------------------------------

d1tvqa_ ------------------------------------------------------------

d2f73a1 ------------------------------------------------------------

d1epaa_ ------------------------------------------------------------

d1xkia_ ------------------------------------------------------------

d1iw2a_ ------------------------------------------------------------

d1vpra1 EKGFEAGDNKLGGALNAKHVEKYGDNFKNGMHKPEFHEDGLHKPMEVGGKKFESGFHYLL 60

d1obqa_ ------------------------------------------------------------

d1qwda_ ------------------------------------------------------------

d1oeja_ ------------------------------------------------------------

d2o62a1 ------------------------------------------------------------

d1avgi_ ------------------------------------------------------------

d2gc9a1 ------------------------------------------------------------

d1r0ua_ ------------------------------------------------------------

d1bj7a_ ------------------------------------------------------------

d1e5pa_ ------------------------------------------------------------

d1a3ya_ ------------------------------------------------------------

d1ew3a_ ------------------------------------------------------------

d1gm6a_ ------------------------------------------------------------

d2ozqa1 ------------------------------------------------------------

d1beba_ ------------------------------------------------------------

d1yupa1 ------------------------------------------------------------

d1exsa_ ------------------------------------------------------------

d1brqa_ ------------------------------------------------------------

d1jyda_ ------------------------------------------------------------

d1hbqa_ ------------------------------------------------------------

d2ofmx1 ------------------------------------------------------------

d3np1a_ ------------------------------------------------------------

d1euoa_ ------------------------------------------------------------

d2a13a1 ------------------------------------------------------------

d2fr2a1 ------------------------------------------------------------

d1cbia_ ------------------------------------------------------------

d1xcaa_ ------------------------------------------------------------

d1kqxa_ ------------------------------------------------------------

d1opaa_ ------------------------------------------------------------

d1lpja_ ------------------------------------------------------------

d1ggla_ ------------------------------------------------------------

d1ftpa_ ------------------------------------------------------------

d1liba_ ------------------------------------------------------------

d1p6pa_ ------------------------------------------------------------

d1tvqa_ ------------------------------------------------------------

d2f73a1 ------------------------------------------------------------

d1epaa_ ------------------------------------------------------------

d1xkia_ ------------------------------------------------------------

d1iw2a_ ------------------------------------------------------------

d1vpra1 ECHELGGKNASGGYGGPLCEDPYGSEVQAMTEKLLKEADSDRTLCFNNFQDPCPQLTKEQ 120

d1obqa_ ------------------------------------------------------------

d1qwda_ ------------------------------------------------------------

d1oeja_ ------------------------------------------------------------

d2o62a1 ------------------------------------------------------------

d1avgi_ ------------------------------------------------------------

d2gc9a1 ------------------------------------------------------------

d1r0ua_ ------------------------------------------------------------

d1bj7a_ ---------------------------------IDPSKIPG-EWRIIYAAADNKDKIVEG 26

d1e5pa_ -----------------------------------FAELQG-KWYTIVIAADNLEKIEEG 24

d1a3ya_ -----------------------------------PFELSG-KWITSYIGSSDLEKIGEN 24

d1ew3a_ ----------------------------VAIRNFDISKISG-EWYSIFLASDVKEKIEEN 31

d1gm6a_ ----------------------------VVTSNFDASKIAG-EWYSILLASDAKENIEEN 31

d2ozqa1 ------------------------EEASSTGRNFNVEKING-EWHTIILASDKREKIEDN 35

d1beba_ ----------------------------QTMKGLDIQKVAG-TWYSLAMAASD-ISLLDA 30

d1yupa1 ------------------------IIVTQTMKDLDVQKVAG-TWYSLAMAASD-ISLLDA 34

d1exsa_ ------------------------------MTELDTQKVAG-TWHTVAMAVSD-VSLLDA 28

d1brqa_ -------------------ERDCRVSSFRVKENFDKARFSG-TWYAMAKKDPEGLFLQDN 40

d1jyda_ -------------------ERDCRVSSFRVKENFDKARFSG-TWYAMAKKDPEGLFLQDN 40

d1hbqa_ -------------------ERDCRVSSFRVKENFDKARFAG-TWYAMAKKDPEGLFLQDN 40

d2ofmx1 ---------------------ACTKNAIAQTGFNKDKYFNGDVWYVTDYLDLEPDDVPKR 39

d3np1a_ ---------------------KCTKNALAQTGFNKDKYFNGDVWYVTDYLDLEPDDVPKR 39

d1euoa_ --------------------MDCSTNISPKQGLDKAKYFSG-KWYVTHFLDKDPQ-VTDQ 38

d2a13a1 --------------------------PPVHPFVAPLSYLLG-TWRGQGEGEYPTIPSFRY 33

d2fr2a1 ---------------------------DLAPALQALSPLLG-SWAGRGAGKYPTIRPFEY 32

d1cbia_ ------------------------------------PNFAG-TWKMRSSENFDELLKALG 23

d1xcaa_ ------------------------------------PNFSG-NWKIIRSENFEELLKVLG 23

d1kqxa_ -----------------------------------PADFNG-TWEMLSNDNFEDVMKALD 24

d1opaa_ -----------------------------------TKDQNG-TWEMESNENFEGYMKALD 24

d1lpja_ -----------------------------------PADLSG-TWTLLSSDNFEGYMLALG 24

d1ggla_ -----------------------------------PPNLTG-YYRFVSQKNMEDYLQALN 24

d1ftpa_ -----------------------------------VKEFAGIKYKLDSQTNFEEYMKAIG 25

d1liba_ -----------------------------------CDAFVG-TWKLVSSENFDDYMKEVG 24

d1p6pa_ -------------------------------------AFNG-TWNVYAQENYENFLRTVG 22

d1tvqa_ -------------------------------------AFSG-TWQVYAQENYEEFLKALA 22

d2f73a1 ------------------------------------MSFSG-KYQLQSQENFEAFMKAIG 23

d1epaa_ ------------------------------VKDFDISKFLG-FWYEIAFASKMGTPGLAH 29

d1xkia_ -------------------------------------DVSG-TWYLKAMTVDREFPEMNL 22

d1iw2a_ ---------------------ASPISTIQPKANFDAQQFAG-TWLLVAVGSAGRFLQEQG 38

d1vpra1 VAMCKGFDYGDKTLKLPCGPLPWPAGLPEPGYVPKTNPLHG-RWITVSGGQAAFIKEAIK 179

d1obqa_ --------DKIPDFVVPGKCASVDRNKLWAEQTPNRNSYAG-VWYQFALTNNPYQLIEKC 51

d1qwda_ ---------HLESTSLYKKSSSTPPRGVTVVNNFDAKRYLG-TWYEIARFDHRFERGLEK 50

d1oeja_ ----------GKPLTEVEQKAANGVFDDANVQNRTLSDWDG-VWQSVYPLLQSGKLDPVF 49

d2o62a1 -----------------------------ERPLLQINDLLG-EWRGQAVTIYRDLRPPDI 30

d1avgi_ ----------------------------AEGDDCSIEKAMG-DFKPEEFFNGTWYLAHGP 31

d2gc9a1 ----------------------------MTKTFKTLDDFLGTHFIYTYDNGWEYEWYAKN 32

d1r0ua_ -----------------------------GFQSNAMKQETPITLHVKSVIEDDGNQEVIE 31

d1bj7a_ --GPLRNYY------RRIECINDCESLSITFYLKDQGTCLLLTEVAKRQE-GYVYVLEFY 77

d1e5pa_ --GPLRFYF------RHIDCYKNCSEMEITFYVITNNQCSKTTVIGYLKG-NGTYETQFE 75

d1a3ya_ --APFQVFM------RSIEFDDKESKVYLNFFSKENGICEEFSLIGTKQE-GNTYDVNYA 75

d1ew3a_ --GSMRVFV------DVIRALDNSS-LYAEYQTKVNGECTEFPMVFDKTEEDGVYSLNYD 82

d1gm6a_ --GSMRVFV------EHIRVLDNSS-LAFKFQRKVNGECTDFYAVCDKVG-DGVYTVAYY 81

d2ozqa1 --GNFRLFL------EQIHVLEKS--LVLKFHTVRDEECSELSMVADKTEKAGEYSVTYD 85

d1beba_ QSAPLRVYV------EELKPTPEGD-LEILLQKWENGECAQKKIIAEKTKIPAVFKID-- 81

d1yupa1 QSAPLRVYV------EELKPTPGGD-LEILLQKWENGKCAQKKIIAEKTEIPAVFKID-- 85

d1exsa_ KSSPLKAYV------EGLKPTPEGD-LEILLQKRENDKCAQEVLLAKKTDIPAVFKIN-- 79

d1brqa_ IVAEFSVDE------TGQMSATAKG-RVRLLNNWD--VCADMVGTFTDTEDPAKFKMKYW 91

d1jyda_ IVAEFSVDE------TGQMSATAKG-RVRLLNNWD--VCADMVGTFTDTEDPAKFKMKYW 91

d1hbqa_ IVAEFSVDE------NGQMSATAKG-RVRLLNNWD--VCADMVGTFTDTEDPAKFKMKYW 91

d2ofmx1 YCAALAAGTASGKLKEALYHYDPKTQDTFYDVSELQVESLG-KYTANFKKVDKNGNVKVA 98

d3np1a_ YCAALAAGTASGKLKEALYHYDPKTQDTFYDVSELQEESPG-KYTANFKKVEKNGNVKVD 98

d1euoa_ YCSSFTPRESDGTVKEALYHYNANKKTSFYNIGEGKLESSGLQYTAKYKTVDKKKAVLKE 98

d2a13a1 GEEIRFSHSG-----KPVIAYTQKT----WKLESGAPMHAESGYFRPRPDGSIEVVIAQS 84

d2fr2a1 LEEVVFAHVG-----KPFLTYTQQT----RAVADGKPLHSETGYLRVCRPGCVELVLAHP 83

d1cbia_ VNAMLRKVA------------VAAASKPHVEIRQD-GDQFYIKTSTTVRTTEINFKVGEG 70

d1xcaa_ VNVMLRKIA------------VAAASKPAVEIKQE-GDTFYIKTSTTVRTTEINFKVGEE 70

d1kqxa_ IDFATRKIA------------VHLK--QTKVIVQN-GDKFETKTLSTFRNYEVNFVIGEE 69

d1opaa_ IDFATRKIA------------VRLT--QTKIIVQD-GDNFKTKTNSTFRNYDLDFTVGVE 69

d1lpja_ IDFATRKIA------------KLLK--PQKVIEQN-GDSFTIHTNSSLRNYFVKFKVGEE 69

d1ggla_ ISLAVRKIA------------LLLK--PDKEIEHQ-GNHMTVRTLSTFRNYTVQFDVGVE 69

d1ftpa_ VGAIERKAG------------LALS--PVIELEILDGDKFKLTSKTAIKNTEFTFKLGEE 71

d1liba_ VGFATRKVA------------GMAK--PNMIISVN-GDLVTIRSESTFKNTEISFKLGVE 69

d1p6pa_ LPEDIIKVA------------KDVN--PVIEIEQN-GNEFVVTSKTPKQTHSNSFTVGKE 67

d1tvqa_ LPEDLIKMA------------RDIK--PIVEIQQK-GDDFVVTSKTPRQTVTNSFTLGKE 67

d2f73a1 LPEELIQKG------------KDIK--GVSEIVQN-GKHFKFTITAGSKVIQNEFTVGEE 68

d1epaa_ KEEKMGAMV----------VELKENLLALTTTYYSEDHCVLEKVTATEGDGPAKFQVTRL 79

d1xkia_ ESVTPMTLT-----------TLEGGNLEAKVTMLISGRCQEVKAVLEKTDEPGKYTADGG 71

d1iw2a_ HRAEATTLH----------VAPQGTAMAVSTFRKLDGICWQVRQLYGDTGVLGRFLLQAR 88

d1vpra1 SGMLGAAEAN----KIVADTDHHQTGGMYLRINQFGDVCTVDASVAKFARAKRTWKSGHY 235

d1obqa_ VRNEYSFDG-------------KQFVIESTGIAYDGNLLKRNGKLYPNPFGEPHLSIDYE 98

d1qwda_ VTATYSLRD------------DGGLNVINKGYNPDRGMWQQSEGKAYFTGAPTRAALKVS 98

d1oeja_ QKKADADKTKTFAEIKDYYHKGYATDIEMIGIEDGIVEFHRNNETTSCKYDYDGYKILTY 109

d2o62a1 YSTTLKIQL------------DDAG----RLMQSTSFGERTITSTATIKGSIVLFDQDPE 74

d1avgi_ GVTSPAVCQ-------------------KFTTSGSKGFTQIVEIGYNKFESNVKFQCN-- 70

d2gc9a1 DHTVDYRIHG------GMVAGRWVTDQKADIVMLTEGIYKISWTEPTGTDVALDFMPNEK 86

d1r0ua_ FRTTGFYYVK---------------QNKVYLSYYEEHDLGKVKTIVKVSEGEVLVMRSG- 75

d1bj7a_ ---------------------------------GTNTLEVIHVSENMLVTYVENYDGE-R 103

d1e5pa_ ---------------------------------GNNIFQPLYITSDKIFFTNKNMDRAGQ 102

d1a3ya_ ---------------------------------GNNKFVVSYASETALIISNINVDEEGD 102

d1ew3a_ ---------------------------------GYNVFRISEFENDEHIILYLVNFDKDR 109

d1gm6a_ ---------------------------------GENKFRLLEVNYSDYVILHLVDVNGDK 108

d2ozqa1 ---------------------------------GFNTFTIPKTDYDNFLMAHLINEKDGE 112

d1beba_ -------------------------------ALNENKVLVLDTDYKKYLLFCMENSAEPE 110

d1yupa1 -------------------------------ALNENKVLVLDTDYKKYLLFCMENSAEPE 114

d1exsa_ -------------------------------ALDENQLFLLDTDYDSHLLLCMENSASPE 108

d1brqa_ GVASFL-------------------------QKGNDDHWIVDTDYDTYAVQYSCRLLNLD 126

d1jyda_ GVASFL-------------------------QKGNDDHWIVDTDYDTYAVQYSCRLLNLD 126

d1hbqa_ GVASFL-------------------------QKGNDDHWIIDTDYETFAVQYSCRLLNLD 126

d2ofmx1 VT-----------------------------AGNYYTFTVMYADDSSALIHTCLHKGNKD 129

d3np1a_ VT-----------------------------SGNYYTFTVMYADDSSALIHTCLHKGNKD 129

d1euoa_ AD-----------------------------EKNSYTLTVLEADDSSALVHICLREGSKD 129

d2a13a1 --------------------------------TGLVEVQKGTYNVD----EQSIKLKSD- 107

d2fr2a1 --------------------------------SGITEIEVGTYSVTGDVIELELSTRADG 111

d1cbia_ -------------------------------------FEEET--VDGRKCRSLPTWENEN 91

d1xcaa_ -------------------------------------FEEQT--VDGRPCKSLVKWESEN 91

d1kqxa_ -------------------------------------FDEQTKGLDNRTVKTLVKWD-GD 91

d1opaa_ -------------------------------------FDEHTKGLDGRNVKTLVTWE-GN 91

d1lpja_ -------------------------------------FDEDNRGLDNRKCKSLVIWD-ND 91

d1ggla_ -------------------------------------FEEDLRSVDGRKCQTIVTWE-EE 91

d1ftpa_ -------------------------------------FDEET--LDGRKVKSTITQDGPN 92

d1liba_ -------------------------------------FDEIT--ADDRKVKSIITLDG-G 89

d1p6pa_ -------------------------------------SEITS--MDGKKIKVTVQLEG-G 87

d1tvqa_ -------------------------------------ADITT--MDGKKLKCTVHLAN-G 87

d2f73a1 -------------------------------------CELET--MTGEKVKTVVQLEGDN 89

d1epaa_ S--------------------------------GKKEVVVEATDYLTYAIIDITSLVAGA 107

d1xkia_ ----------------------------------KHVAYIIRSHVKDHYIFYSEGELHGK 97

d1iw2a_ G------------------------------ARGAVHVVVAETDYQSFAVLYLERAG--- 115

d1vpra1 FYEPLVSGGNLLGVWVLPEEYRKIGFFWEMESGRCFRIERRAFPVGPYTFMRQATEVGGK 295

d1obqa_ N------------------------------SFAAPLVILETDYSNYACLYSCIDYNFGY 128

d1qwda_ ----------------------------------FFGPFYGGYNVIALDREYRHALVCGP 124

d1oeja_ KSGK---------------------------KGVRYLFECKDPESKAPKYIQFSDHIIAP 142

d2o62a1 ------------------------------------KQVQVLLLPDGASATSPLKVQLRQ 98

d1avgi_ ------------------------------------QVDNKNGEQYSFKCKSSDNTEFEA 94

d2gc9a1 KLHG--------------------TIFFPKWVEEHPEITVTYQNEHIDLMEQSREKYATY 126

d1r0ua_ ------------------------------------AVKMNQRFVTGASTIAKYKMSFGE 99

d1bj7a_ ITKMTEGLAKGTSFTPEELEKYQQLNSERGVPNENIENLIKTDNCPP---------- 150

d1e5pa_ ETNMIVVAGKGNALTPEENEILVQFAHEKKIPVENILNILATDTCPE---------- 149

d1a3ya_ KTIMTGLLGKGTDIEDQDLEKFKEVTRENGIPEENIVNIIERDDCPA---------- 149

d1ew3a_ PFQLFEFYAREPDVSPEIKEEFVKIVQKRGIVKENIIDLTKIDRCFQLRG------- 159

d1gm6a_ TFQLMEFYGRKPDVEPKLKDKFVEICQQYGIIKENIIDLTKIDRCFQLRG------- 158

d2ozqa1 TFQLMGLYGREPDLSSDIKERFAQLCEEHGILRENIIDLSNANRC------------ 157

d1beba_ QSLVCQCLVRTPEVDDEALEKFDKALKALPMHIRLSFNPTQLEEQC----------- 156

d1yupa1 QSLACQCLVRTPEVDDEAMEKFDKALKALPMHIRLSFNPTQLEEQCRV--------- 162

d1exsa_ HSLVCQSLARTLEVDDQIREKFEDALKTLSVPMRI--LPAQLEEQCRV--------- 154

d1brqa_ GTCADSYSFVFSRDPNGLPPEAQKIVRQRQEELCLARQYRLIVHNGYC--------- 174

d1jyda_ GTCADSYSFVFSRDPNGLPPEAQKIVRQRQEELCLARQYRLIVHNGYC--------- 174

d1hbqa_ GTCADSYSFVFARDPSGFSPEVQKIVRQRQEELCLARQYRLIPHNGYCNG------- 176

d2ofmx1 LGDLYAVLNRNKDAAAG--DKVKSAVSAATLEFSKFISTKENNCAYDNDSLKSLLTK 184

d3np1a_ LGDLYAVLNRNKDTNAG--DKVKGAVTAASLKFSDFISTKDNKCEYDNVSLKSLLTK 184

d1euoa_ LGDLYTVLTHQKDAEPS--AKVKSAVTQAGLQLSQFVGTKDLGCQYDD-QFTSL--- 180

d2a13a1 ---LVGNASKVKEISREFELVDGKLSYVVRMSTTTNPLQPHLKAILDKL-------- 153

d2fr2a1 SIGLAPTAKEVTALDRSYRIDGDELSYSLQMRAVGQPLQDHLAAVLHRQR------- 161

d1cbia_ KIHCTQTLLEGDGPKTYWTRELAND-ELILTFGADDVVCTRIYVRE----------- 136

d1xcaa_ KMVCEQKLLKGEGPKTSWTMELTNDGELILTMTADDVVCTRVYVRE----------- 137

d1kqxa_ KLVCVQ---KGEKENRGWKQWIEGD-LLHLEIHCQDKVCHQVFKKKN---------- 134

d1opaa_ TLVCVQ---KGEKENRGWKQWVEGD-KLYLELTCGDQVCRQVFKKK----------- 133

d1lpja_ RLTCIQ---KGEKKNRGWTHWIEGD-KLHLEMFCEGQVCKQTFQRA----------- 133

d1ggla_ HLVCVQ---KGEVPNRGWRHWLEGE-MLYLELTARDAVCEQVFRKVH---------- 134

d1ftpa_ KLVHEQ---KGD-HPTIIIREFSKE-QCVITIKLGDLVATRIYKAQ----------- 133

d1liba_ ALVQVQ---KWDGKSTTIKRKRDGD-KLVVECVMKGVTSTRVYERA----------- 131

d1p6pa_ KLICKS-------DKFSHIQEVNGD-EMVEKITIGSSTLTRKSKRV----------- 125

d1tvqa_ KLVTKS-------EKFSHEQEVKGN-EMVETITFGGVTLIRRSKRV----------- 125

d2f73a1 KLVTTF-------KNIKSVTELNGD-IITNTMTLGDIVFKRISKRI----------- 127

d1epaa_ VHRTMKLYSRSLDDNGEALYNFRKITSDHGFSETDLYILKHDLTCVKVLQSAA---- 160

d1xkia_ PVRGVKLVGRDPKNNLEALEDFEKAAGARGLSTESILIPRQS--------------- 139

d1iw2a_ -QLSVKLYARSLPVSDSVLSGFEQRVQEAHLTEDQIFYFPKYGFCEAADQFHVLDEV 171

d1vpra1 ISFVFYVKVSNDPESDPIPLQSRDYTALAGRDNAPTNLGKPYPTLAKDLDYPKKRD- 351

d1obqa_ HSDFSFIFSRSANLADQYVKKCEAAFKNINVDTTRFVKTVQGSSCPYDTQKTL---- 181

d1qwda_ DRDYLWILSRTPTISDEVKQEMLAVATREGFDVSKFIWVQQPG-------------- 167

d1oeja_ RKSSHFHIFMGNDSQQSLLNEMENWPTYYPYQLSSEEVVEEMMSH------------ 187

d2o62a1 PLFLEAGWLIQSDLRQRMIRSYNDKGEWVSLTLVTEERV------------------ 137

d1avgi_ DFTFISVSYDNFALVCRSITFTSQPKEDRYLVFERTKSDTDPDAKEIC--------- 142

d2gc9a1 PKLVVPEFANITYMGDAGQNNEDVISEAPYKEMPNDIRNGKYFDQNYHRLNK----- 178

d1r0ua_ LELKTSTKSIQSDLDEEKGRISIAYDMHVGDEQEHLHNMTITYEGGT---------- 146
